# Optimization data on total cost of ownership for conventional and battery electric heavy vehicles driven by humans and by automated driving systems

**DOI:** 10.1016/j.dib.2020.105566

**Published:** 2020-04-18

**Authors:** Toheed Ghandriz, Bengt Jacobson, Leo Laine, Jonas Hellgren

**Affiliations:** a Division of Vehicle Engineering and Autonomous Systems, Department of Mechanics and Maritime Sciences, Chalmers University of Technology, Gothenburg SE-41296, Sweden; b Volvo Group Trucks Technology, Gothenburg SE-40508, Sweden

**Keywords:** Transportation, Total cost of ownership data, Automated driving systems, Heavy vehicles, Electrified propulsion

## Abstract

In road freight transport, the emerging technologies such as automated driving systems improve the mobility, productivity and fuel efficiency. However, the improved efficiency is not enough to meet environmental goals due to growing demands of transportation. Combining automated driving systems and electrified propulsion can substantially improve the road freight transport efficiency. However, the high cost of the battery electric heavy vehicles is a barrier hindering their adoption by the transportation companies. Automated driving systems, requiring no human driver on-board, make the battery electric heavy vehicles competitive to their conventional counterparts in a wider range of transportation tasks and use cases compared to the vehicles with human drivers. The presented data identify transportation tasks where the battery electric heavy vehicles driven by humans or by automated driving systems have lower cost of ownership than their conventional counterparts. The data were produced by optimizing the vehicle propulsion system together with the loading/unloading schemes and charging powers, with the objective of minimizing the total cost of ownership on 3072 different transportation scenarios, according to research article “Impact of automated driving systems on road freight transport and electrified propulsion of heavy vehicles” (Ghandriz et al., 2020) [2]. The data help understanding the effects of traveled distance, road hilliness and vehicle size on the total cost of ownership of the vehicles with different propulsion and driving systems. Data also include sensitivity tests on the uncertain parameters.

Specifications Table

**Table:** 

Subject	Engineering
Specific subject area	Transportation, Energy, Automotive Engineering and mathematical optimization
Type of data	Chart and Graph
How data were acquired	By implementing mathematical simulation and optimization using models of roads and vehicle dynamics
Data format	Raw and Analyzed
Parameters for data collection	Roads of different lengths and hillinesses and vehicles of different size and driving system, i.e., human-driven or driven by automated driving systems.
Description of data collection	The optimum Heavy vehicle propulsion system and corresponding infrastructure with the lowest total cost of ownership was found in terms of size of the internal combustion engine, the type and number of battery packs, type and number of electric motors, charging powers from charging stations and loading/unloading (LU) scheme. The optimization was repeated for roads of different lengths and hillinesses and vehicles of different size and driving system, i.e., human-driven or driven by automated driving systems.
Data source location	Sweden
Data accessibility	With the article
Related research article	Ghandriz, Toheed and Jacobson, Bengt and Laine, Leo and Hellgren, Jonas, Impact of Automated Driving Systems on Road Freight Transport and Electrified Propulsion of Heavy Vehicles, Journal of Transportation Research Part C: Emerging Technologies, In Press

## Value of the data

• These data provide the total cost of ownership and cost components of deploying different heavy vehicles in the various transportation scenarios and help to perform a comparative assessment between different road freight transport solutions.
• These data provide valuable information for the practitioners, vehicle manufactures and transportation companies with regard to competitiveness of the battery electric heavy vehicles and automated driving systems (i.e., high and full driving automation) against the conventional heavy vehicles and vehicles driven by human drivers.
• These data help further research in adoption of the automated driving systems and battery electric heavy vehicles in road freight transport.
• These data identify those transportation scenarios, where the battery electric heavy vehicles become competitive to the conventional combustion-powered heavy vehicles.
• Data provide sensitivity of the total cost of ownership to the different parameters such as the utilization level and fuel efficiency covering a wide range of transportation scenarios in the different geographical regions.

## 1 Data description

Three data files were provided.

1. mainDataOptTCO.mat, that includes the total cost of ownership (TCO), cost components of TCO, duration time of a round-trip including charging and LU, and the optimum setup of vehicle propulsion and infrastructure of all the 3072 transportation scenarios. The content of the file and the variable names are explained in Table 1. Each variable is a 4-dimensional cell array corresponding to the road hilliness, road length, vehicle size and average trip speed. The data were produced assuming 100% utilization rate (i.e. vehicle-time on operation), and using the nominal values of vehicles and cost parameters provided in [2], when there was a single vehicle in the fleet.

**Table 1:** Content of file “mainDataOptTCO.mat”

Variable name	Description
adsBEHV_MissionTimes⁎	A 4-dimensional cell array containing the mission times of the automated driving system-dedicated battery electric heavy vehicles (ADS BEHVs)
adsCHV_optPropInfra⁎	A 4-dimensional cell array containing the propulsion hardware, loading-unloading and charging powers, explained in Table 2, belonging to the automated driving system-dedicated conventional combustion-powered heavy vehicles (ADS CHVs)
hdBEHV_TCOcomponents⁎	A 4-dimensional cell array containing the components of TCO belonging to human-driven battery electric heavy vehicles (HD BEHVs)
hdCHV_TCOPerYearPerTon⁎	A 4-dimensional cell array of the optimum TCO belonging to human-driven conventional combustion-powered heavy vehicles (HD CHVs)

⁎ A similar variable exists for the other vehicles and driving systems.
2. sensitivityData.mat, that includes the sensitivity tests of TCO and its cost components with respect to the fuel price, vehicle utilization, battery price, fuel efficiency, economic life time, discount rate, automation-specific hardware price and electric energy price. Sensitivity tests were done for optimum average speed and for all roads, vehicles and driving systems, i.e., human-driven or driven by the automated driving systems, when there was a single vehicle in the fleet.
3. roadData.mat, that includes topographic data, i.e., elevation and road grade versus distance of all road types.

The data were analyzed and related figures were provided in this article. Furthermore, Matlab codes used for generating the figures from the raw data were provided in files “optimumTCOgraphs.m” and “sensitivityFigures.m”. File “optimumTCOgraphs.m” also provides the optimum propulsion system and infrastructure of all vehicles, that drive in an optimum average speed, as an output, given a road length and hilliness. Moreover, the number of vehicles of the same type in the fleet can be selected.

## 2 Experimental design, materials, and methods

A transportation scenario is defined by the following parameters.

• Road length, i.e., the distance between pickup an delivery points or charging stations that can be a choice from 10, 20, 40, 80, 160, and 320 km.
• Road hilliness, i.e., a choice from flat road (FR), predominantly flat road (PFR), hilly road (HR), and very hilly road (VHR).
• Average reference speed, that can be a choice from 20, 30, 40, 50, 60, 70, 80, and 90 km/h.
• Type of the vehicle regarding its propulsion system, that can be either a battery electric heavy vehicle (BEHV) or a conventional combustion-powered heavy vehicle (CHV).
• Vehicle size, that can be one of the followings: rigid truck (RT), tractor-semitrailer (TS), Nordic combination (NC), and A-double (AD), with the gross mass of 25 ton, 40 ton, 60 ton, and 80 ton, respectively.
• Driving system, i.e., human-driven (HD) or driven by the automated driving systems with no human driver on-board. According to [1] J3016, the latter is called automated driving systems-dedicated vehicle (ADS-DV).

The combination of all the parameter choices above resulted in the 3072 different transportation scenarios. For generating the data, on each of the transportation scenarios, the following optimization problem was solved to optimize the vehicle propulsion system and infrastructure including the LU scheme and charging powers, with the objective of minimizing the TCO.

(1) $$\begin{array}{cccc} & \text{Find} & & \mathrm{size}\;\mathrm{of}\;\mathrm{internal}\;\mathrm{combustion}\;\mathrm{engine}\;\mathrm{(ICE),}\;\mathrm{type}\;\mathrm{of}\;\mathrm{electric}\;\mathrm{motor}\;\mathrm{(EM),}\;\mathrm{number}\\ & & & \mathrm{of}\;\mathrm{EMs,}\;\mathrm{type}\;\mathrm{of}\;\mathrm{battery}\;\mathrm{packs,}\;\mathrm{number}\;\mathrm{of}\;\mathrm{battery}\;\mathrm{packs,}\;\mathrm{LU}\;\mathrm{scheme}\;\mathrm{of}\;\mathrm{the}\;\mathrm{first}\\ & & & \mathrm{container/semi-trailer,}\;\mathrm{LU}\;\mathrm{scheme}\;\mathrm{of}\;\mathrm{the}\;\mathrm{second}\;\mathrm{container/semi-trailer,}\\ & & & \mathrm{charging}\;\mathrm{power}\;\mathrm{at}\;\mathrm{the}\;\mathrm{start}\;\mathrm{node,}\;\mathrm{charging}\;\mathrm{power}\;\mathrm{at}\;\mathrm{the}\;\mathrm{end}\;\mathrm{node}\\ & \text{to}\;\text{minimize} & & \mathrm{fleet}\;\mathrm{total}\;\mathrm{cost}\;\mathrm{of}\;\mathrm{ownership}\;\mathrm{per}\;\mathrm{unit}\;\mathrm{freight}\;\mathrm{transported}\\ & \text{subject}\;\text{to} & & \mathrm{vehicle}\;\mathrm{dynamic}\;\mathrm{model}\;\mathrm{constraints,}\\ & & & \mathrm{performance}\;\mathrm{constraints,}\\ & & & \mathrm{transportation}\;\mathrm{task}\;\mathrm{constraints} \end{array}$$

The design variables and their discrete ranges are according to Table 2.

**Table 2:** Propulsion hardware-infrastructure optimization design variables⁎

Design variable	Discrete range of the design variable
Size of ICE	4 l, 6 l, 8 l, 11 l, 13 l, 16 l
Type of EM	221 Nm, 266 Nm, 400 Nm
Number of EMs	1,2, …, 22
Type of battery packs	power-optimized, energy-optimized
Number of battery packs	1,2, …, 60
LU scheme of the first container/semi-trailer	on-board waiting, straddle carrier, additional semitrailer, on-board lift
LU scheme of the second container/semi-trailer (if any)	on-board waiting, straddle carrier, additional semitrailer, on-board lift
Charging power at the start node	10, 20, …, 300 kW
Charging power at the end node	10, 20, …, 300 kW

⁎ For detailed specification refer to [2].

As an objective function, the TCO included the operational costs and depreciation of purchase cost. The purchase cost included the cost of chassis, driver cabin, transmission, automated driving systems hardware, ICE, battery packs, EMs, LU, charging stations, and the investment on transportation mission management system (TMMS) needed for managing ADS-DVs. The operational costs included the cost of driver, diesel fuel, electric energy, maintenance, tax, insurance, and the operational costs related to TMMS. In calculating the TCO, the details such as the driver rest time and battery degradation and replacement were considered. Moreover, the difference in the rest value of the battery and other vehicle hardware was taken into account, depending on the battery state of health when vehicle service life ends. Furthermore, the optimization constraints ensure the vehicle proper operation on each of the transportation scenarios using models of vehicle dynamics. More details were provided in [2].

**Fig. 1:**
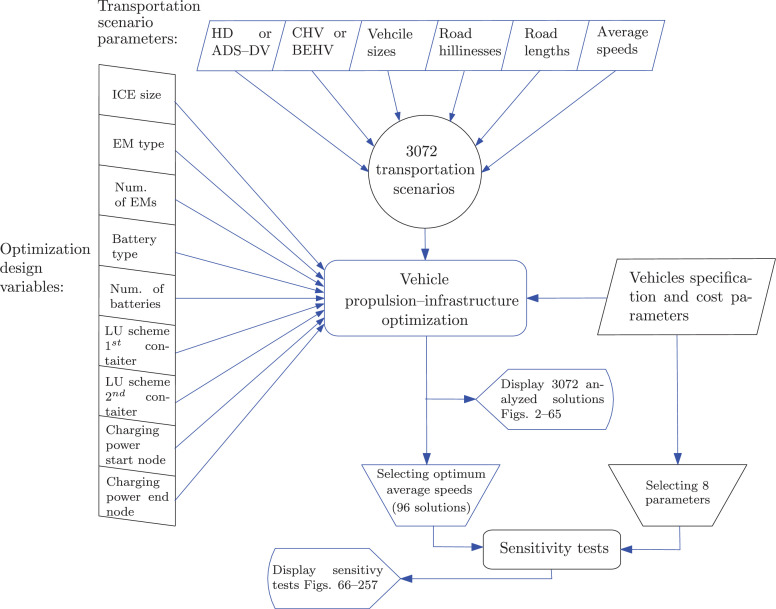
The process of data generation and presentation. Please refer to [2] for the nominal values of the vehicles specification and cost parameters. The selected 8 parameters used for sensitivity tests include the fuel price, vehicle utilization, battery price, fuel efficiency, economic life time, discount rate, automation-specific hardware price and electric energy price..

Finally, after solving the optimization problems, the sensitivity tests were performed on all the roads and for all the vehicles but only for an average reference speed that yielded the lowest TCO. Fig. 1 depicts the process of data generation and presentation.

### 2.1 Particle swarm optimization algorithm

The defined optimization problem (1) is nonlinear and non-smooth. The stochastic optimization methods and in particular the particle swarm optimization (PSO) showed to be effective methods for solving such problems, according to [3], [5] and [4]; however, the global optimum may not be found unless solving the optimization problem is repeated many times. The presented data include the best attained solution among 20 optimization runs which took about 12 days to run on a computer with 32 cores and 92 GB of RAM.

PSO stochastically and iteratively moves a population of particles, i.e., points in the search space, closer to an optimum solution. PSO uses the speed and direction of particles’ motion relative to their own positions or the position of the best particle that is found in the previous iteration. PSO algorithm according to [5] is described as follows.

1. Determine the range of design variables *a_k_* in Table 2, i.e. the search space, and their minimum and maximum values; or alternatively their indices *k*, since the variables may belong to discrete and/or non-numeric sets. In that case, the design variables must be arranged in an increasing order if applicable.
  $k_{min}=\left\{1,1,1,1,1,1,1,1,1\right\}$, $k_{max}=\left\{6,3,11,2,60,4,4,30,30\right\}$
2. Initialize the swarm, i.e., the position of particles *k* and their speeds *v*, randomly, and the cost function *C_t_*.
  $k_{ij}=\left\lfloor k_{min,ij}+r_{s,ij}\left(k_{max,ij}-k_{min,ij}\right)\right\rceil ,$$v_{ij}=r_{v,ij}\left(k_{max,ij}-k_{min,ij}\right)-0.5\left(k_{max,ij}-k_{min,ij}\right),$$C_{t}\left(a_{k_{i}^{p}}\right)=10^{16}$,$C_{t}\left(a_{k^{gp}}\right)=10^{16}$,i=1,…,N,j=1,…,n,
  where ⌊ · ⌉ is the closest integer function, *r*_*s,ij*_ and *r*_*v, ij*_ denote random numbers in [0,1], *C_t_* denotes total cost of ownership per unit freight, $k_{i}^{p}$ is the previous position of the $i^{\text{th}}$ particle, *k^gp^* denotes previous global best position, *N* denotes the number of particles in the swarm, and *n* denotes the number of dimensions of the search space, or the number of design variables (n=9).
3. Evaluate the cost function $C_{t}\left(a_{k_{i}}\right),$ for i=1,…,N, according to [2].
4. Update the best global position and position of each particle, for i=1,…,N, if its current position is better than previous position.
  $k_{i}^{p}\leftarrow k_{i},$ if $C_{t}\left(a_{k_{i}}\right)<C_{t}\left(a_{k_{i}^{p}}\right)$*, k^gp^* ← *k_i_*, if $C_{t}\left(a_{k_{i}}\right)<C_{t}\left(a_{k^{gp}}\right)$
5. Update velocities and positions, for i=1,…,N,j=1,…,n.
  $v_{ij}\leftarrow wv_{ij}+2q\left(k_{ij}^{p}-k_{ij}\right)/\Delta t+2r\left(k^{gp}-k_{ij}\right)/\Delta t$*, v_min_* ≤ *v_ij_* ≤ *v_max,_*$k_{ij}\leftarrow k_{ij}+\Delta tv_{ij}$
  where, *q* and *r* denote random numbers in [0,1], Δt=1 is the time step, and *w* is a number (usually in [0,1]) that determines the influence of the previous velocity on the current velocity.
6. Return to step 3 if termination criteria is not satisfied.

## 3 Analyzed data

The data can be used for choosing the right vehicle in terms of the vehicle size, propulsion system, and the driving system (i.e., human-driven or driven by the automated driving systems), knowing the properties of the transportation scenario such as the road length and road hilliness. Provided figures handle multidimensionality of the data and facilitate data interpretation and comparative analysis. Table 3, Table 4, Table 5, Table 6 explain the content of figures. The figures presented in Table 3, Table 4, Table 5, were produced assuming 100% utilization rate (i.e. vehicle-time on operation), and using the nominal values of vehicles specification and cost parameters provided in [2].

**Table 3:** The optimized TCO versus average speed is shown for the different road lengths, propulsion and driving systems. The vehicle size and road hilliness are fixed while other transportation scenario parameters vary in the figures. The variation of vehicle size and road hilliness between figures is according to this table.

Fixed parameter	Flat road	Predominantly flat road	Hilly road	Very hilly road
Rigid truck	Fig. 2	Fig. 3	Fig. 4	Fig. 5
Tractor-semitrailer	Fig. 6	Fig. 7	Fig. 8	Fig. 9
Nordic combination	Fig. 10	Fig. 11	Fig. 12	Fig. 13
A-double	Fig. 14	Fig. 15	Fig. 16	Fig. 17

**Table 4:** The optimized TCO versus the average speed is shown for the different vehicle sizes, propulsion and driving systems. The road hilliness and road length are fixed while other transportation scenario parameters vary in the figures. The variation of the road hilliness and road length between figures is according to this table.

Fixed parameter	Flat road	Predominantly flat road	Hilly road	Very hilly road
10 km road	Fig. 18	Fig. 24	Fig. 30	Fig. 36
20 km road	Fig. 19	Fig. 25	Fig. 31	Fig. 37
40 km road	Fig. 20	Fig. 26	Fig. 32	Fig. 38
80 km road	Fig. 21	Fig. 27	Fig. 33	Fig. 39
160 km road	Fig. 22	Fig. 28	Fig. 34	Fig. 40
320 km road	Fig. 23	Fig. 29	Fig. 35	Fig. 41

**Table 5:** The optimized TCO components are shown for the optimum average speed of the corresponding transportation scenario and the different vehicle sizes, propulsion and driving systems. The road hilliness and road length are fixed while other transportation scenario parameters vary in the figures. The variation of the road hilliness and road length between figures is according to this table.

Fixed parameter	Flat road	Predominantly flat road	Hilly road	Very hilly road
10 km road	Fig. 42	Fig. 48	Fig. 54	Fig. 60
20 km road	Fig. 43	Fig. 49	Fig. 55	Fig. 61
40 km road	Fig. 44	Fig. 50	Fig. 56	Fig. 62
80 km road	Fig. 45	Fig. 51	Fig. 57	Fig. 63
160 km road	Fig. 46	Fig. 52	Fig. 58	Fig. 64
320 km road	Fig. 47	Fig. 53	Fig. 59	Fig. 65

**Table 6:** The sensitivity tests of the optimized TCO and its components to the different parameters are shown for the different vehicles and driving systems, and for the optimum speed of the transportation scenario. The vehicle size, road hilliness and road length are fixed in a figure, but they vary between figures according to this table.

Fixed parameter	10 km flat road	10 km predominantly flat road	10 km hilly road	10 km very hilly road
Rigid truck	Figs. 66 and 67	Figs. 74 and 75	Figs. 82 and 83	Figs. 90 and 91
Tractor-semitrailer	Figs. 68 and 69	Figs. 76 and 77	Figs. 84 and 85	Figs. 92 and 93
Nordic combination	Figs. 70 and 71	Figs. 78 and 79	Figs. 86 and 87	Figs. 94 and 95
A-double	Figs. 72 and 73	Figs. 80 and 81	Figs. 88 and 89	Figs. 96 and 97

Fixed parameter	20 km flat road	20 km predominantly flat road	20 km hilly road	20 km very hilly road

Rigid truck	Figs. 98 and 99	Figs. 106 and 107	Figs. 114 and 115	Figs. 122 and 123
Tractor-semitrailer	Figs. 100 and 101	Figs. 108 and 109	Figs. 116 and 117	Figs. 124 and 125
Nordic combination	Figs. 102 and 103	Figs. 110 and 111	Figs. 118 and 119	Figs. 126 and 127
A-double	Figs. 104 and 105	Figs. 112 and 113	Figs. 120 and 121	Figs. 128 and 129

Fixed parameter	40 km flat road	40 km predominantly flat road	40 km hilly road	40 km very hilly road

Rigid truck	Figs. 130 and 131	Figs. 138 and 139	Figs. 146 and 147	Figs. 154 and 155
Tractor-semitrailer	Figs. 132 and 133	Figs. 140 and 141	Figs. 148 and 149	Figs. 156 and 157
Nordic combination	Figs. 134 and 135	Figs. 142 and 143	Figs. 150 and 151	Figs. 158 and 159
A-double	Figs. 136 and 137	Figs. 144 and 145	Figs. 152 and 153	Figs. 160 and 161

Fixed parameter	80 km flat road	80 km predominantly flat road	80 km hilly road	80 km very hilly road

Rigid truck	Figs. 162 and 163	Figs. 170 and 171	Figs. 178 and 179	Figs. 186 and 187
Tractor-semitrailer	Figs. 164 and 165	Figs. 172 and 173	Figs. 180 and 181	Figs. 188 and 189
Nordic combination	Figs. 166 and 167	Figs. 174 and 175	Figs. 182 and 183	Figs. 190 and 191
A-double	Figs. 168 and 169	Figs. 176 and 177	Figs. 184 and 185	Figs. 192 and 193

Fixed parameter	160 km flat road	160 km predominantly flat road	160 km hilly road	160 km very hilly road

Rigid truck	Figs. 194 and 195	Figs. 202 and 203	Figs. 210 and 211	Figs. 218 and 219
Tractor-semitrailer	Figs. 196 and 197	Figs. 204 and 205	Figs. 212 and 213	Figs. 220 and 221
Nordic combination	Figs. 198 and 199	Figs. 206 and 207	Figs. 214 and 215	Figs. 222 and 223
A-double	Figs. 200 and 201	Figs. 208 and 209	Figs. 216 and 217	Figs. 224 and 225

Fixed parameter	320 km flat road	320 km predominantly flat road	320 km hilly road	320 km very hilly road

Rigid truck	Figs. 226 and 227	Figs. 234 and 235	Figs. 242 and 243	Figs. 250 and 251
Tractor-semitrailer	Figs. 228 and 229	Figs. 236 and 237	Figs. 244 and 245	Figs. 252 and 253
Nordic combination	Figs. 230 and 231	Figs. 238 and 239	Figs. 246 and 247	Figs. 254 and 255
A-double	Figs. 232 and 233	Figs. 240 and 241	Figs. 248 and 249	Figs. 256 and 257

In figures, the chassis cost includes also the cost of driver cabin, transmission and automated driving systems hardware.

The TCO optimizations were done while there was a single vehicle in the fleet. However, file “optimumTCOgraphs.m” provides the possibility to regenerate the data (other than sensitivity data) for more number of vehicles than one, with a fixed propulsion system and infrastructure, assuming that the optimum propulsion system and infrastructure are not influenced by the number of fleet vehicles. However, for the sensitivity figures, if the number of vehicles in a transportation scenario is more that one, the cost components relating to charging station (and possibly LU) must be divided by the number of vehicles. Furthermore, if the cost of the charging infrastructure is intended to be excluded from the TCO, for example, if it is provided by a third party, then it must be disregarded in the figures.

Refer to [2] for better interpretation of data presented in the figures.

**Fig. 2:**
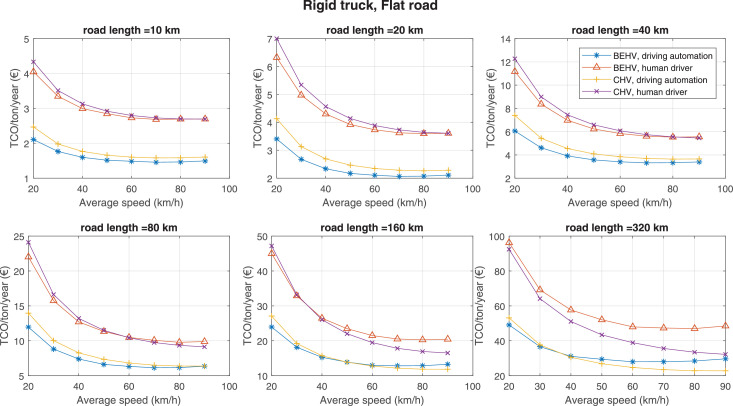
Rigid truck and flat road; the vehicle size and road hilliness are fixed while other transportation scenario parameters vary in the figure. Each dot in the plots corresponds to a vehicle with an optimized propulsion hardware and infrastructure. See Table 3 for the other vehicle sizes and road hillinesses.

**Fig. 3:**
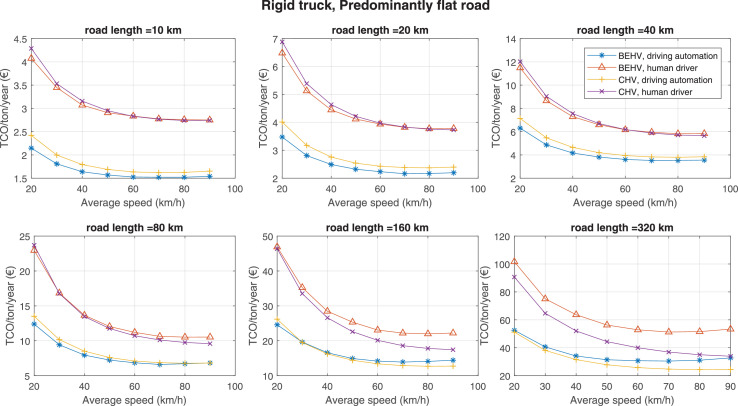
Rigid truck and predominantly flat road; the vehicle size and road hilliness are fixed while other transportation scenario parameters vary in the figure. Each dot in the plots corresponds to a vehicle with an optimized propulsion hardware and infrastructure. See Table 3 for the other vehicle sizes and road hillinesses.

**Fig. 4:**
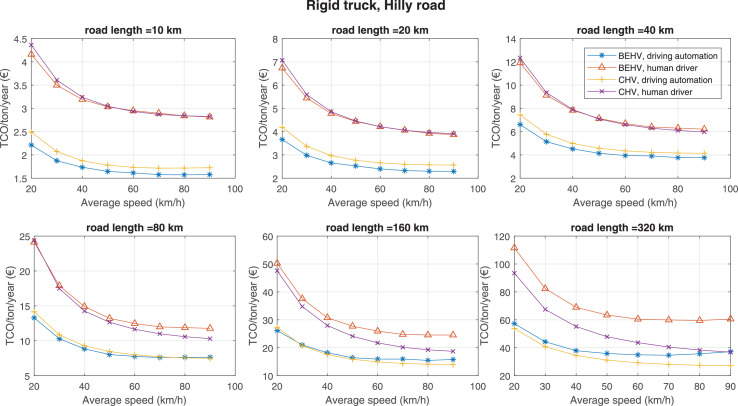
Rigid truck and hilly road; the vehicle size and road hilliness are fixed while other transportation scenario parameters vary in the figure. Each dot in the plots corresponds to a vehicle with an optimized propulsion hardware and infrastructure. See Table 3 for the other vehicle sizes and road hillinesses.

**Fig. 5:**
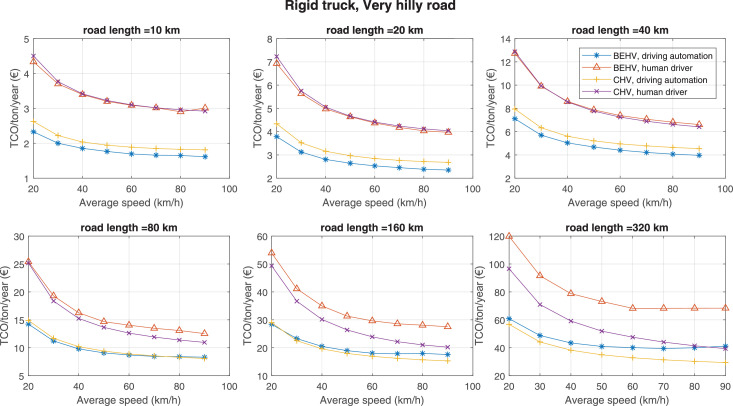
Rigid truck and very hilly road; the vehicle size and road hilliness are fixed while other transportation scenario parameters vary in the figure. Each dot in the plots corresponds to a vehicle with an optimized propulsion hardware and infrastructure. See Table 3 for the other vehicle sizes and road hillinesses.

**Fig. 6:**
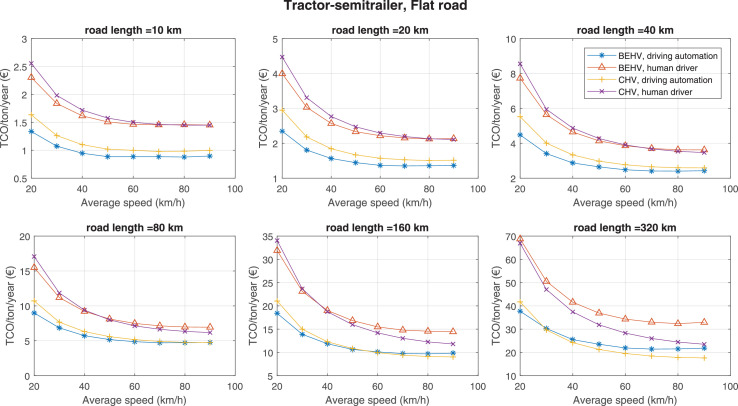
Tractor-semitrailer and flat road; the vehicle size and road hilliness are fixed while other transportation scenario parameters vary in the figure. Each dot in the plots corresponds to a vehicle with an optimized propulsion hardware and infrastructure. See Table 3 for the other vehicle sizes and road hillinesses.

**Fig. 7:**
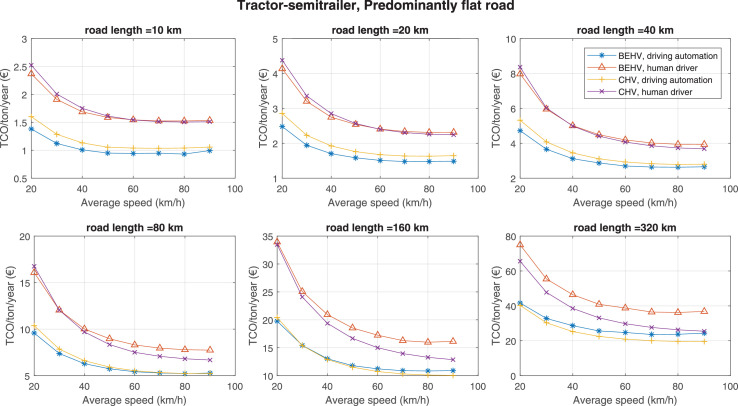
Tractor-semitrailer and predominantly flat road; the vehicle size and road hilliness are fixed while other transportation scenario parameters vary in the figure. Each dot in the plots corresponds to a vehicle with an optimized propulsion hardware and infrastructure. See Table 3 for the other vehicle sizes and road hillinesses.

**Fig. 8:**
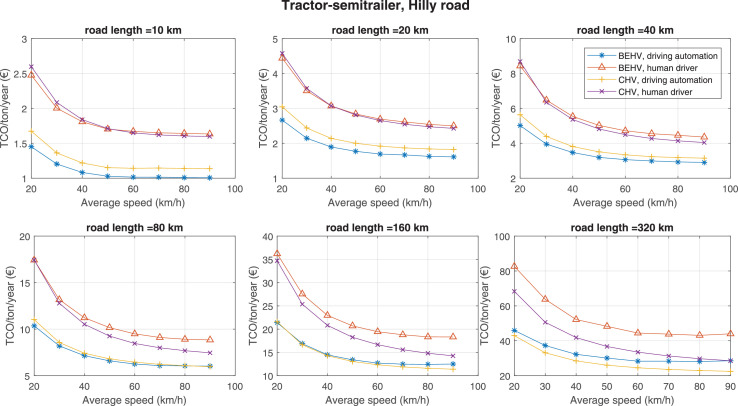
Tractor-semitrailer and hilly road; the vehicle size and road hilliness are fixed while other transportation scenario parameters vary in the figure. Each dot in the plots corresponds to a vehicle with an optimized propulsion hardware and infrastructure. See Table 3 for the other vehicle sizes and road hillinesses.

**Fig. 9:**
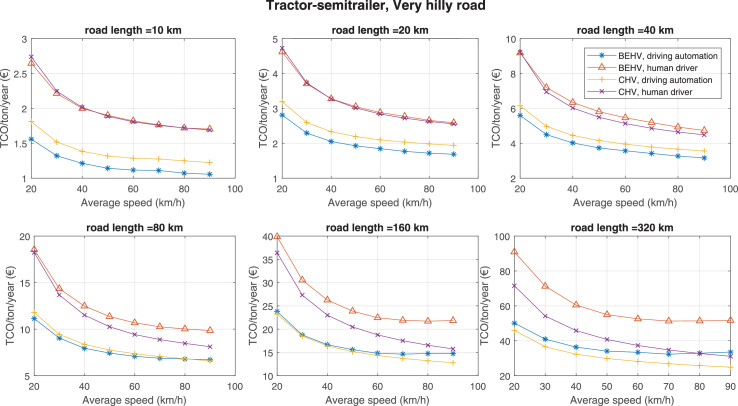
Tractor-semitrailer and very hilly road; the vehicle size and road hilliness are fixed while other transportation scenario parameters vary in the figure. Each dot in the plots corresponds to a vehicle with an optimized propulsion hardware and infrastructure. See Table 3 for the other vehicle sizes and road hillinesses.

**Fig. 10:**
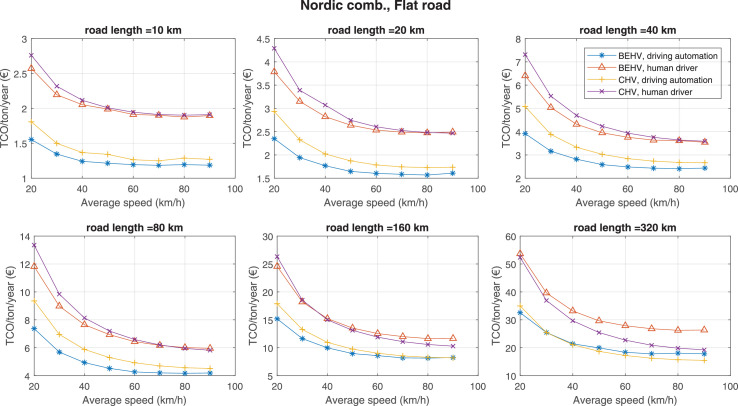
Nordic combination and flat road; the vehicle size and road hilliness are fixed while other transportation scenario parameters vary in the figure. Each dot in the plots corresponds to a vehicle with an optimized propulsion hardware and infrastructure. See Table 3 for the other vehicle sizes and road hillinesses.

**Fig. 11:**
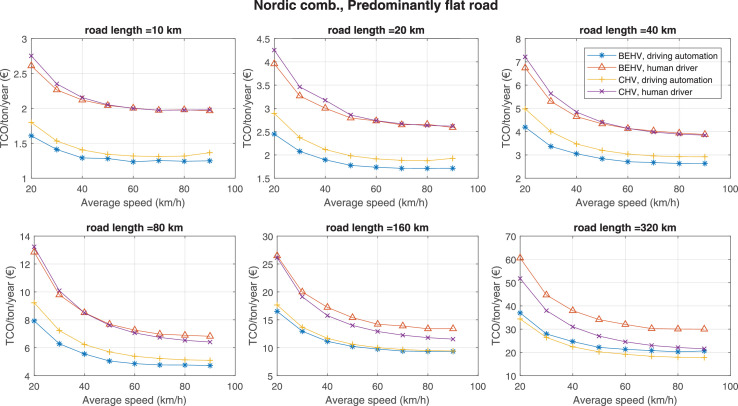
Nordic combination and predominantly flat road; the vehicle size and road hilliness are fixed while other transportation scenario parameters vary in the figure. Each dot in the plots corresponds to a vehicle with an optimized propulsion hardware and infrastructure. See Table 3 for the other vehicle sizes and road hillinesses.

**Fig. 12:**
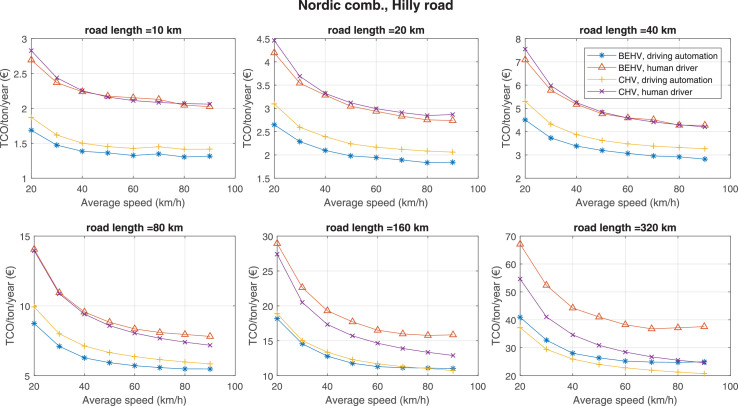
Nordic combination and hilly road; the vehicle size and road hilliness are fixed while other transportation scenario parameters vary in the figure. Each dot in the plots corresponds to a vehicle with an optimized propulsion hardware and infrastructure. See Table 3 for the other vehicle sizes and road hillinesses.

**Fig. 13:**
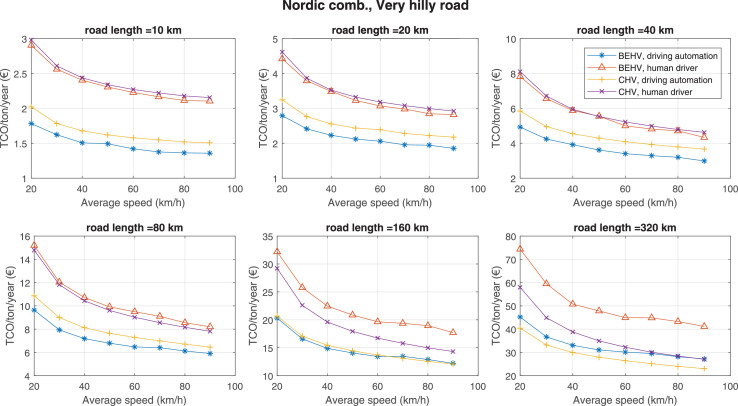
Nordic combination and very hilly road; the vehicle size and road hilliness are fixed while other transportation scenario parameters vary in the figure. Each dot in the plots corresponds to a vehicle with an optimized propulsion hardware and infrastructure. See Table 3 for the other vehicle sizes and road hillinesses.

**Fig. 14:**
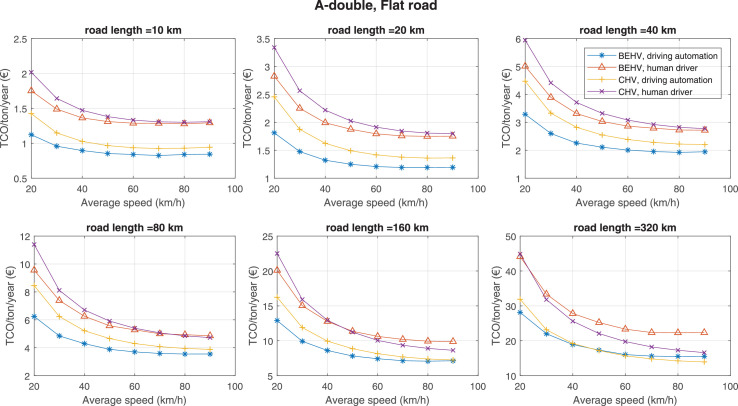
A-double and flat road; the vehicle size and road hilliness are fixed while other transportation scenario parameters vary in the figure. Each dot in the plots corresponds to a vehicle with an optimized propulsion hardware and infrastructure. See Table 3 for the other vehicle sizes and road hillinesses.

**Fig. 15:**
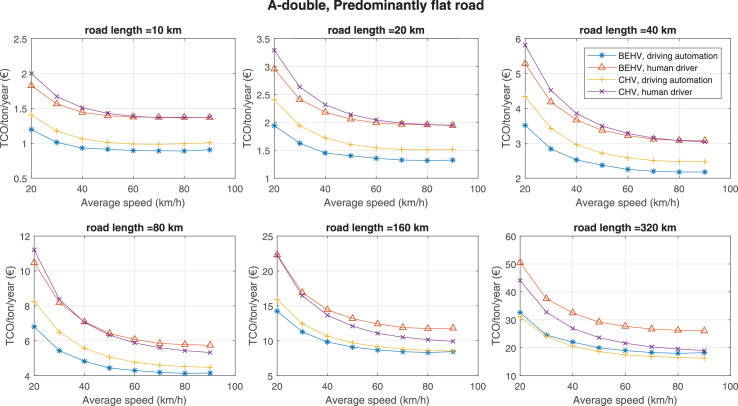
A-double and predominantly flat road; the vehicle size and road hilliness are fixed while other transportation scenario parameters vary in the figure. Each dot in the plots corresponds to a vehicle with an optimized propulsion hardware and infrastructure. See Table 3 for the other vehicle sizes and road hillinesses.

**Fig. 16:**
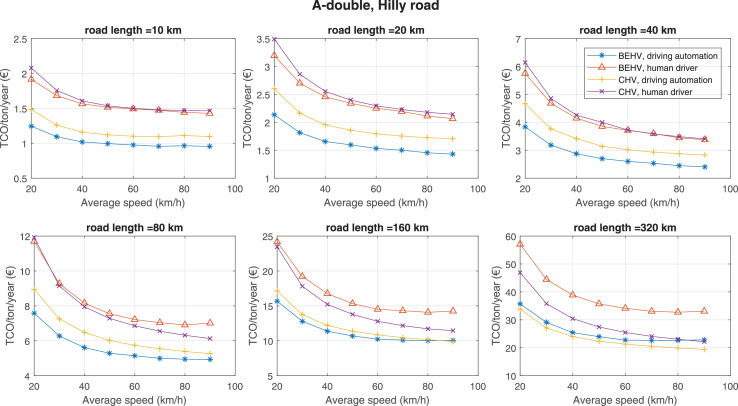
A-double and hilly road; the vehicle size and road hilliness are fixed while other transportation scenario parameters vary in the figure. Each dot in the plots corresponds to a vehicle with an optimized propulsion hardware and infrastructure. See Table 3 for the other vehicle sizes and road hillinesses.

**Fig. 17:**
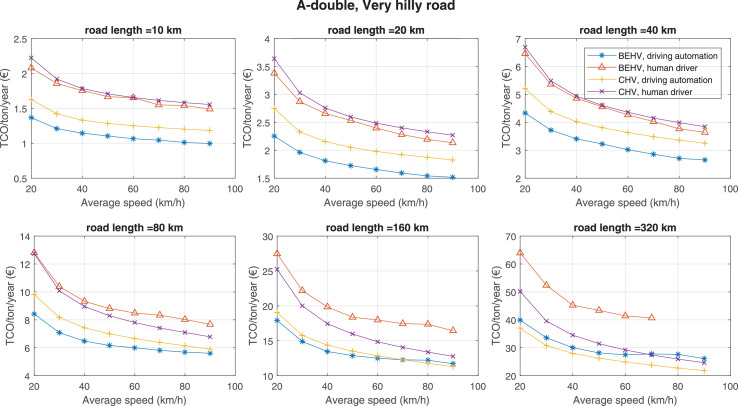
A-double and very hilly road; the vehicle size and road hilliness are fixed while other transportation scenario parameters vary in the figure. Each dot in the plots corresponds to a vehicle with an optimized propulsion hardware and infrastructure. No TCO is available for infeasible solutions in lower-right figure. See Table 3 for the other vehicle sizes and road hillinesses.

**Fig. 18:**
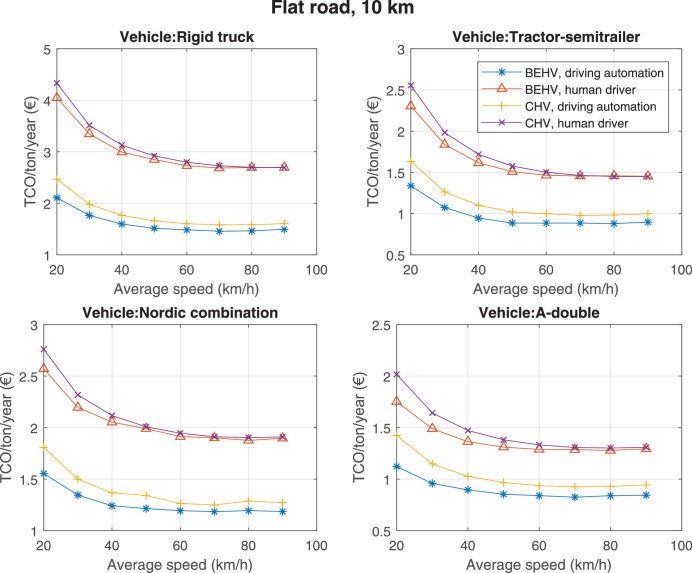
Flat road of 10 km length; the road hilliness and length are fixed while other transportation scenario parameters vary in the figure. Each dot in the plots corresponds to a vehicle with an optimized propulsion hardware and infrastructure. See Table 4 for the other road types.

**Fig. 19:**
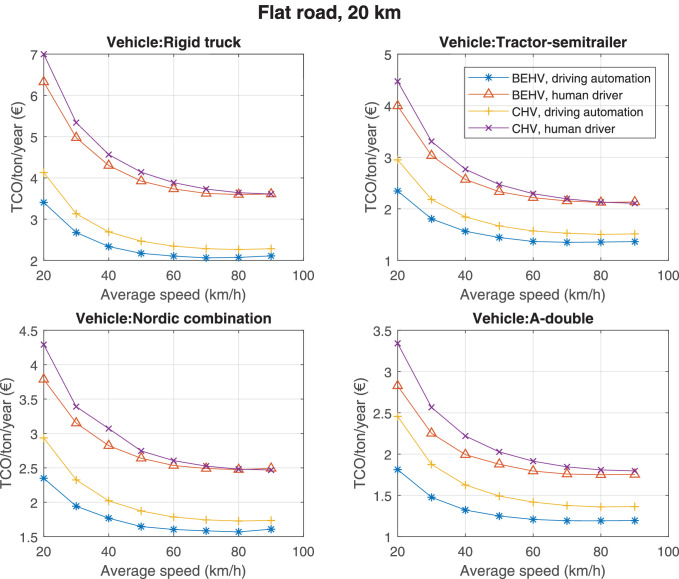
Flat road of 20 km length; the road hilliness and length are fixed while other transportation scenario parameters vary in the figure. Each dot in the plots corresponds to a vehicle with an optimized propulsion hardware and infrastructure. See Table 4 for the other road types.

**Fig. 20:**
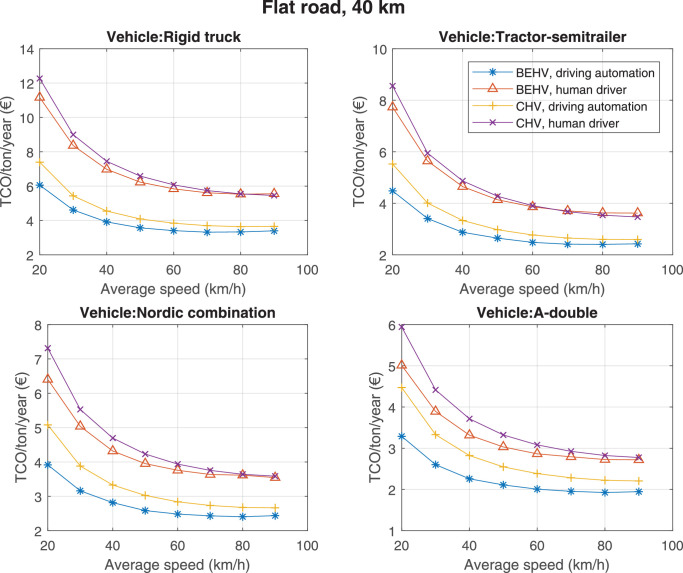
Flat road of 40 km length; the road hilliness and length are fixed while other transportation scenario parameters vary in the figure. Each dot in the plots corresponds to a vehicle with an optimized propulsion hardware and infrastructure. See Table 4 for the other road types.

**Fig. 21:**
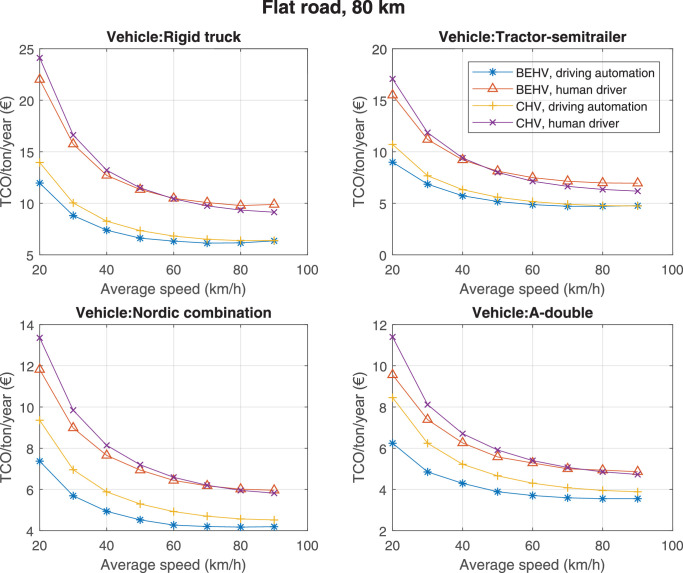
Flat road of 80 km length; the road hilliness and length are fixed while other transportation scenario parameters vary in the figure. Each dot in the plots corresponds to a vehicle with an optimized propulsion hardware and infrastructure. See Table 4 for the other road types.

**Fig. 22:**
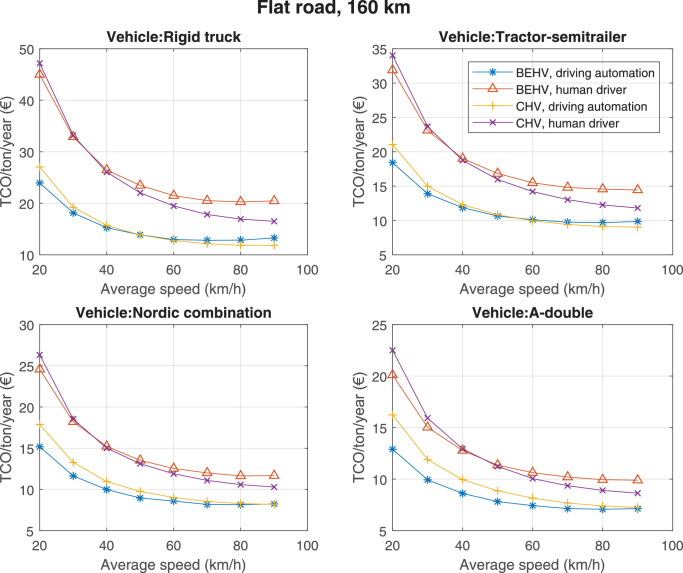
Flat road of 160 km length; the road hilliness and length are fixed while other transportation scenario parameters vary in the figure. Each dot in the plots corresponds to a vehicle with an optimized propulsion hardware and infrastructure. See Table 4 for the other road types.

**Fig. 23:**
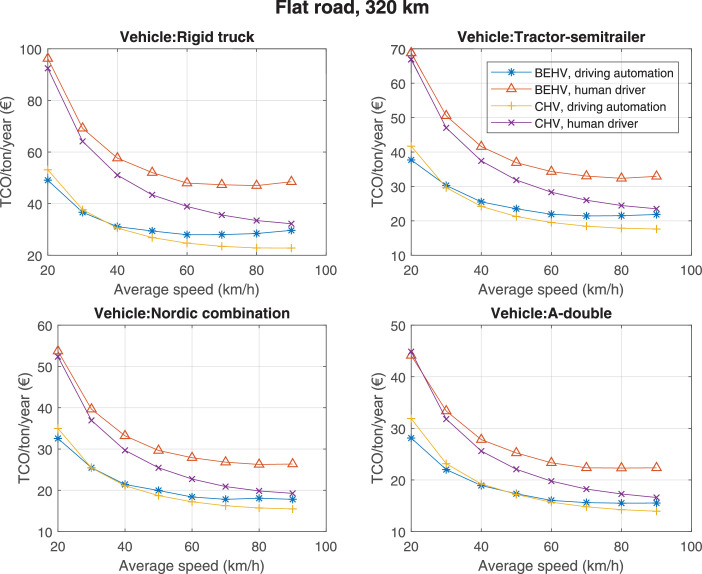
Flat road of 320 km length; the road hilliness and length are fixed while other transportation scenario parameters vary in the figure. Each dot in the plots corresponds to a vehicle with an optimized propulsion hardware and infrastructure. See Table 4 for the other road types.

**Fig. 24:**
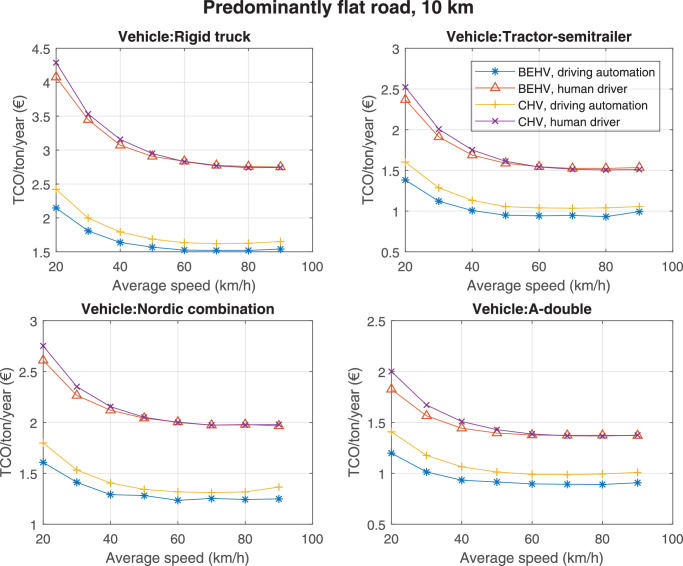
Predominantly flat road of 10 km length; the road hilliness and length are fixed while other transportation scenario parameters vary in the figure. Each dot in the plots corresponds to a vehicle with an optimized propulsion hardware and infrastructure. See Table 4 for the other road types.

**Fig. 25:**
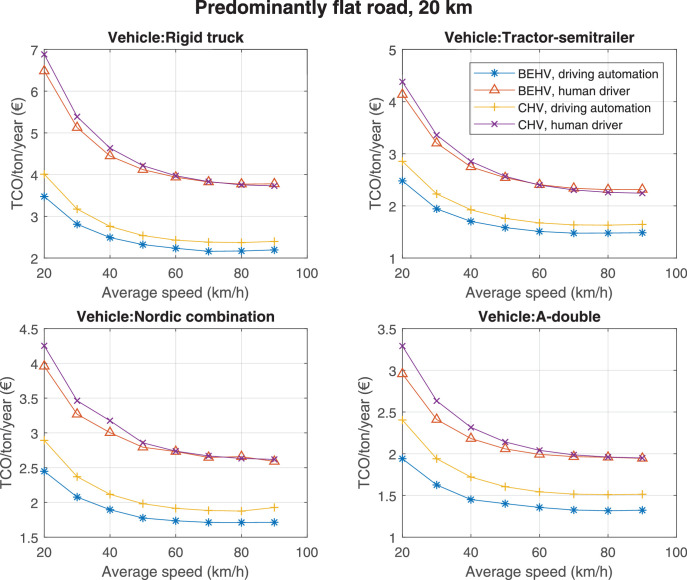
Predominantly flat road of 20 km length; the road hilliness and length are fixed while other transportation scenario parameters vary in the figure. Each dot in the plots corresponds to a vehicle with an optimized propulsion hardware and infrastructure. See Table 4 for the other road types.

**Fig. 26:**
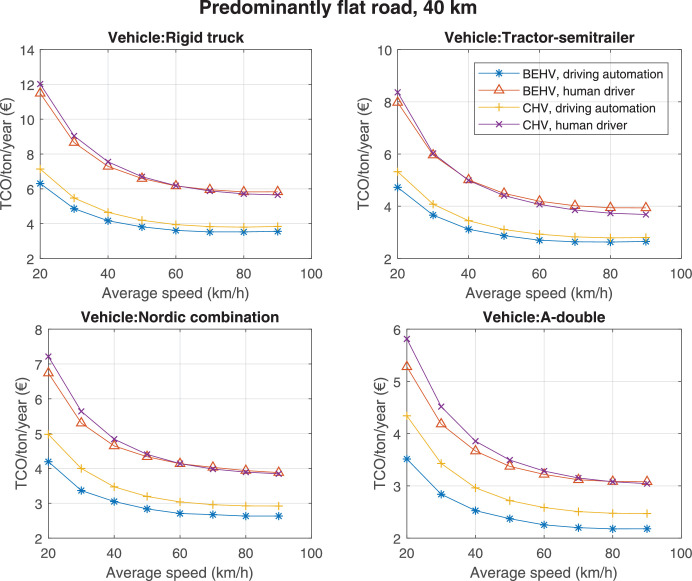
Predominantly flat road of 40 km length; the road hilliness and length are fixed while other transportation scenario parameters vary in the figure. Each dot in the plots corresponds to a vehicle with an optimized propulsion hardware and infrastructure. See Table 4 for the other road types.

**Fig. 27:**
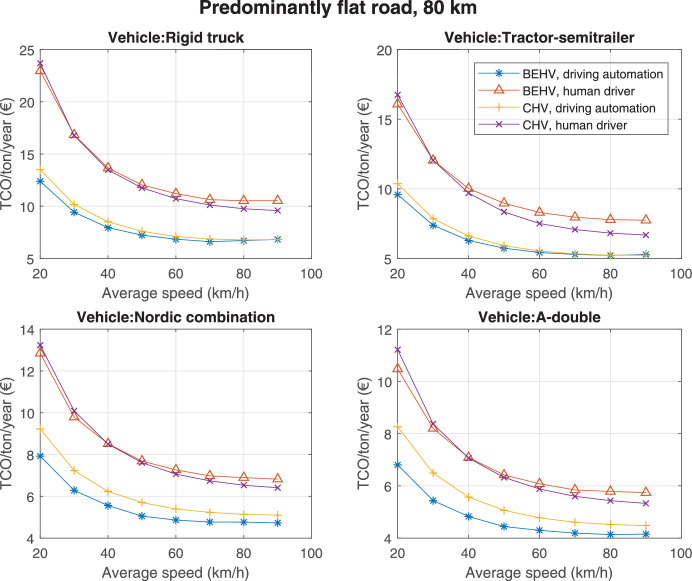
Predominantly flat road of 80 km length; the road hilliness and length are fixed while other transportation scenario parameters vary in the figure. Each dot in the plots corresponds to a vehicle with an optimized propulsion hardware and infrastructure. See Table 4 for the other road types.

**Fig. 28:**
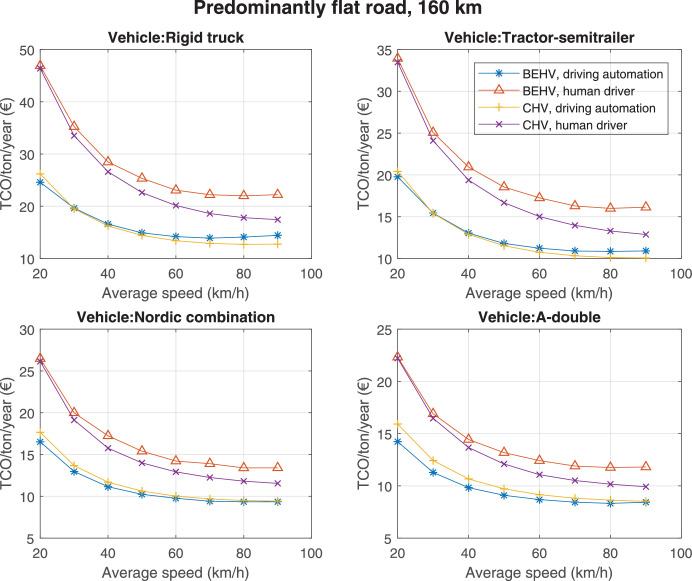
Predominantly flat road of 160 km length; the road hilliness and length are fixed while other transportation scenario parameters vary in the figure. Each dot in the plots corresponds to a vehicle with an optimized propulsion hardware and infrastructure. See Table 4 for the other road types.

**Fig. 29:**
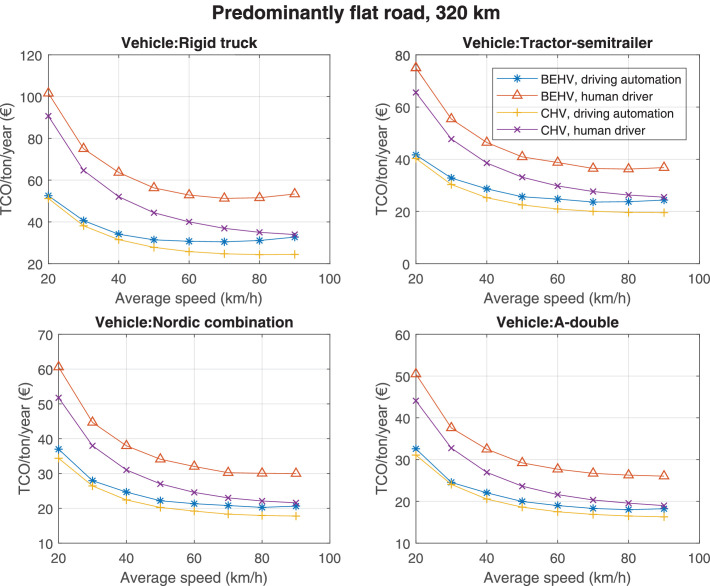
Predominantly flat road of 320 km length; the road hilliness and length are fixed while other transportation scenario parameters vary in the figure. Each dot in the plots corresponds to a vehicle with an optimized propulsion hardware and infrastructure. See Table 4 for the other road types.

**Fig. 30:**
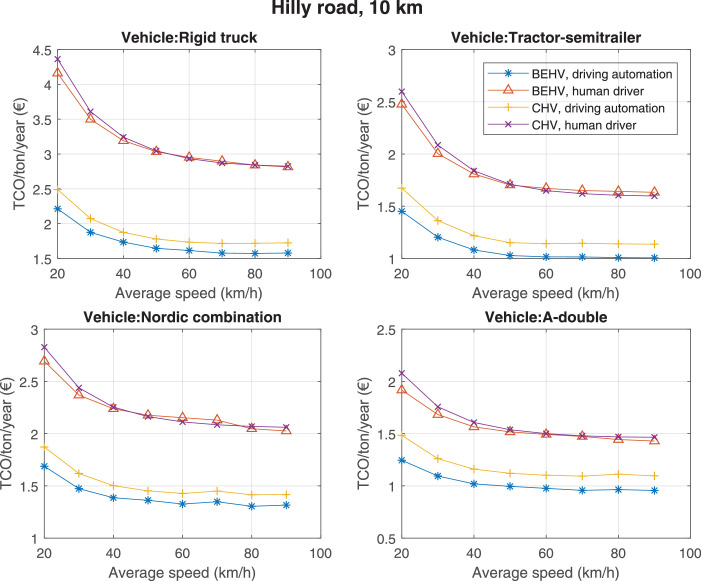
Hilly road of 10 km length; the road hilliness and length are fixed while other transportation scenario parameters vary in the figure. Each dot in the plots corresponds to a vehicle with an optimized propulsion hardware and infrastructure. See Table 4 for the other road types.

**Fig. 31:**
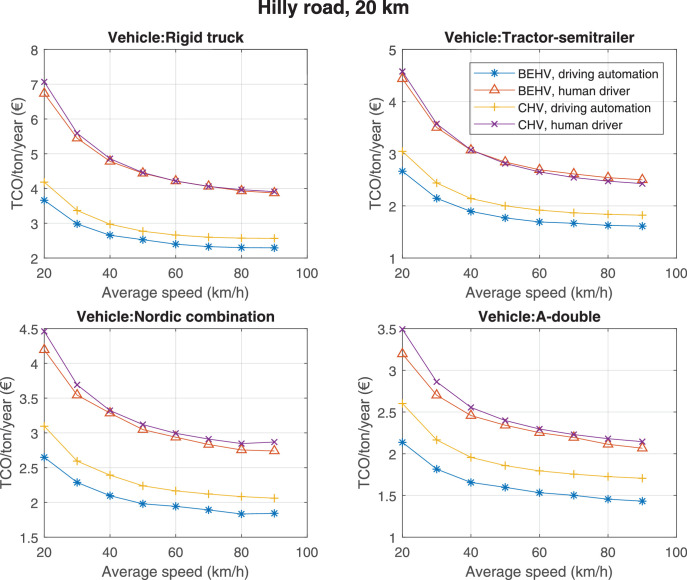
Hilly road of 20 km length; the road hilliness and length are fixed while other transportation scenario parameters vary in the figure. Each dot in the plots corresponds to a vehicle with an optimized propulsion hardware and infrastructure. See Table 4 for the other road types.

**Fig. 32:**
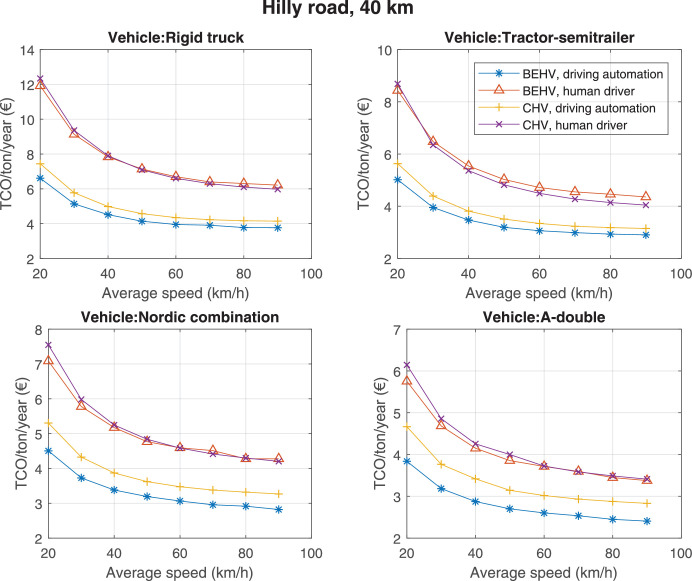
Hilly road of 40 km length; the road hilliness and length are fixed while other transportation scenario parameters vary in the figure. Each dot in the plots corresponds to a vehicle with an optimized propulsion hardware and infrastructure. See Table 4 for the other road types.

**Fig. 33:**
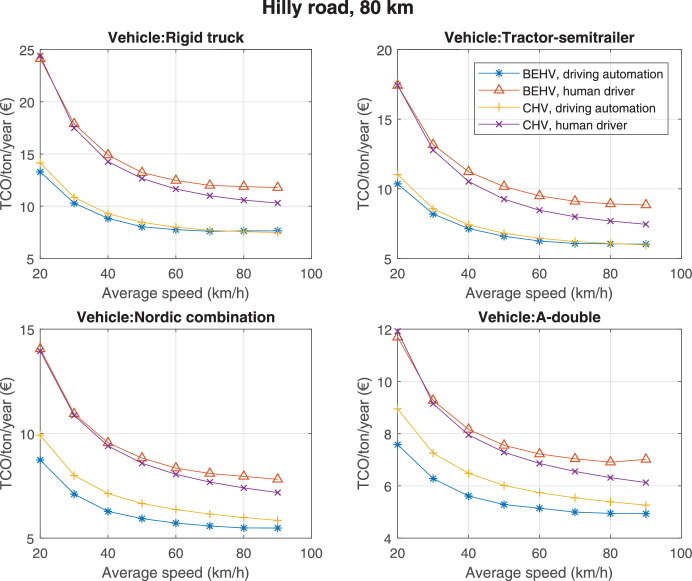
Hilly road of 80 km length; the road hilliness and length are fixed while other transportation scenario parameters vary in the figure. Each dot in the plots corresponds to a vehicle with an optimized propulsion hardware and infrastructure. See Table 4 for the other road types.

**Fig. 34:**
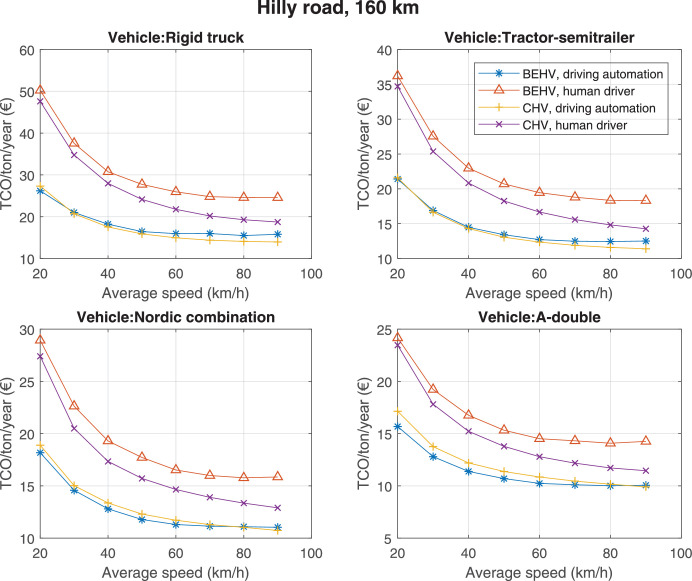
Hilly road of 160 km length; the road hilliness and length are fixed while other transportation scenario parameters vary in the figure. Each dot in the plots corresponds to a vehicle with an optimized propulsion hardware and infrastructure. See Table 4 for the other road types.

**Fig. 35:**
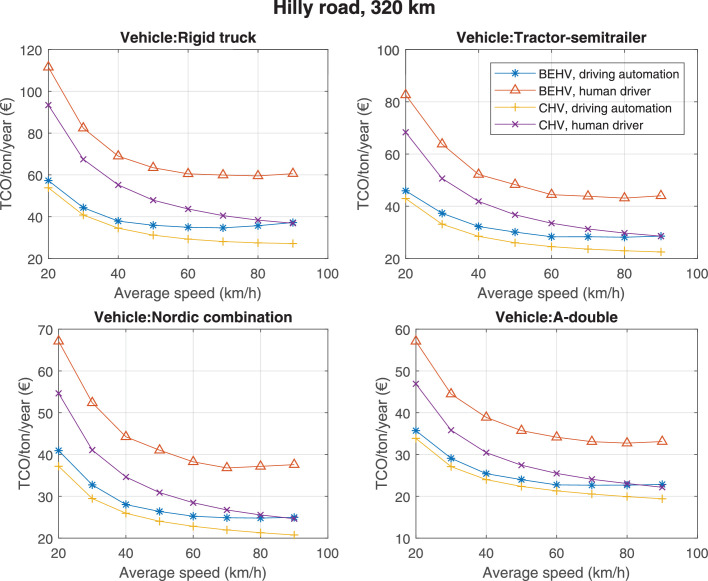
Hilly road of 320 km length; the road hilliness and length are fixed while other transportation scenario parameters vary in the figure. Each dot in the plots corresponds to a vehicle with an optimized propulsion hardware and infrastructure. See Table 4 for the other road types.

**Fig. 36:**
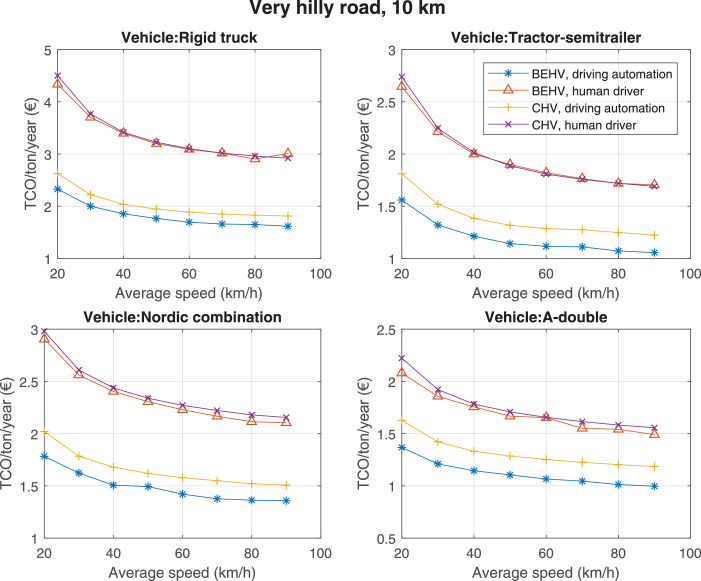
Very hilly road of 10 km length; the road hilliness and length are fixed while other transportation scenario parameters vary in the figure. Each dot in the plots corresponds to a vehicle with an optimized propulsion hardware and infrastructure. See Table 4 for the other road types.

**Fig. 37:**
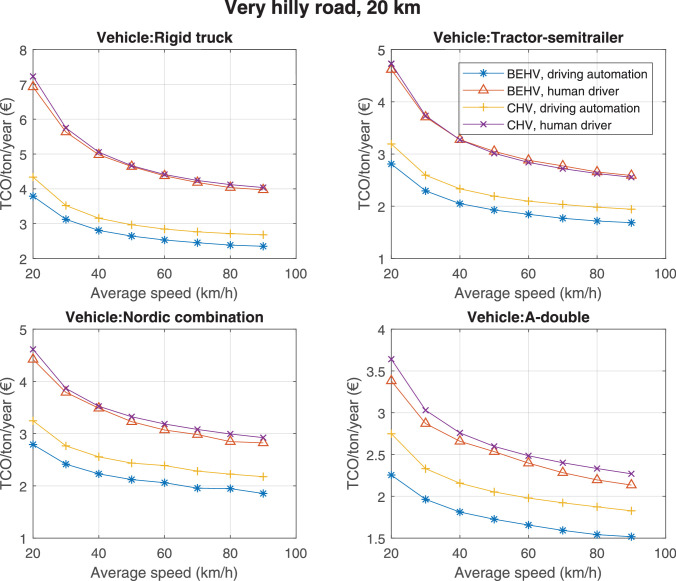
Very hilly road of 20 km length; the road hilliness and length are fixed while other transportation scenario parameters vary in the figure. Each dot in the plots corresponds to a vehicle with an optimized propulsion hardware and infrastructure. See Table 4 for the other road types.

**Fig. 38:**
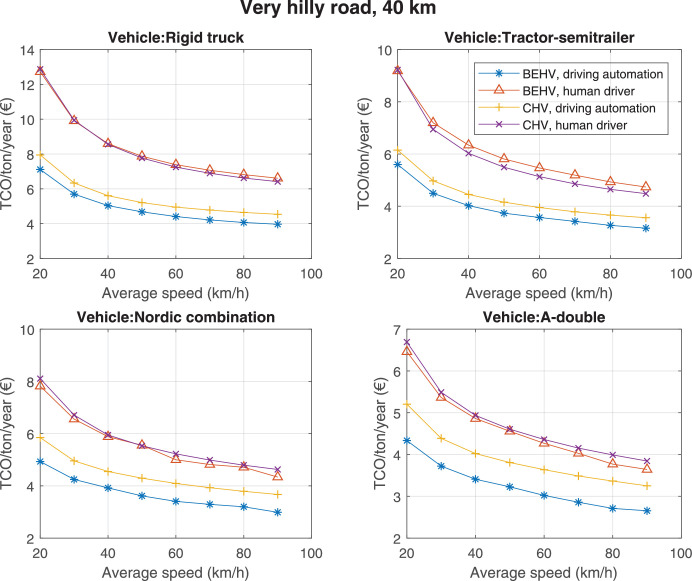
Very hilly road of 40 km length; the road hilliness and length are fixed while other transportation scenario parameters vary in the figure. Each dot in the plots corresponds to a vehicle with an optimized propulsion hardware and infrastructure. See Table 4 for the other road types.

**Fig. 39:**
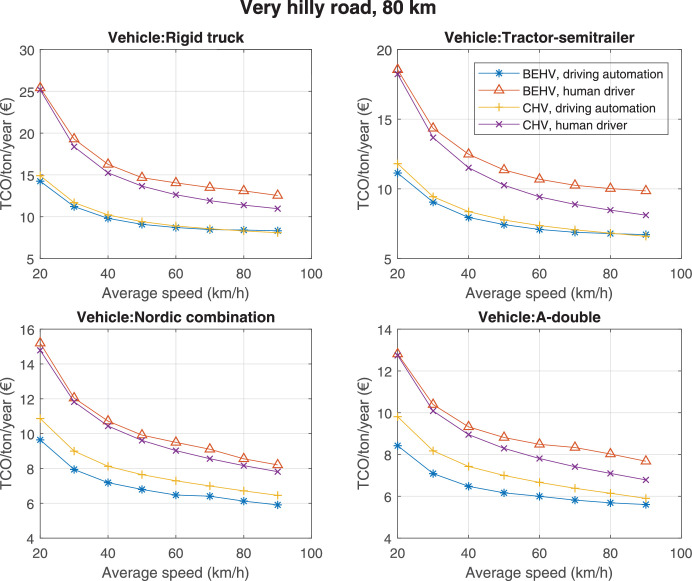
Very hilly road of 80 km length; the road hilliness and length are fixed while other transportation scenario parameters vary in the figure. Each dot in the plots corresponds to a vehicle with an optimized propulsion hardware and infrastructure. See Table 4 for the other road types.

**Fig. 40:**
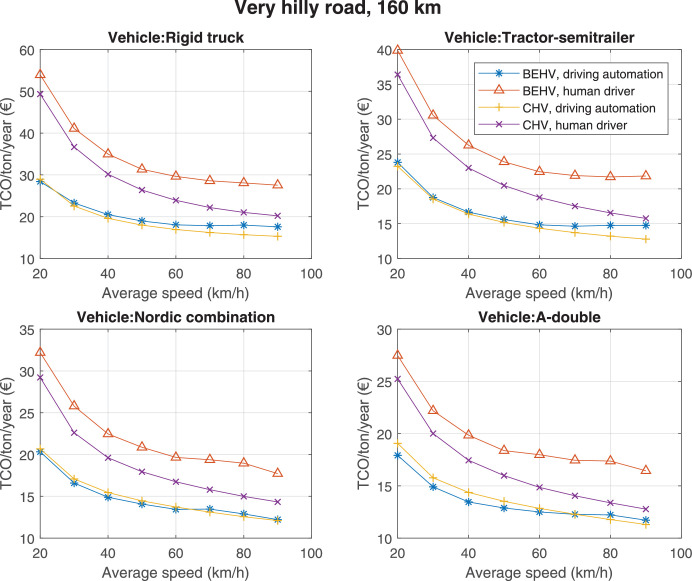
Very hilly road of 160 km length; the road hilliness and length are fixed while other transportation scenario parameters vary in the figure. Each dot in the plots corresponds to a vehicle with an optimized propulsion hardware and infrastructure. See Table 4 for the other road types.

**Fig. 41:**
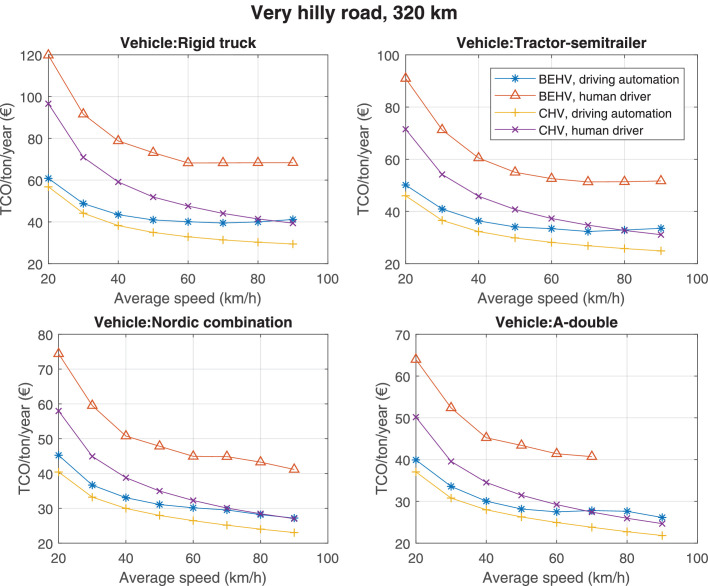
Very hilly road of 320 km length; the road hilliness and length are fixed while other transportation scenario parameters vary in the figure. Each dot in the plots corresponds to a vehicle with an optimized propulsion hardware and infrastructure. See Table 4 for the other road types.

**Fig. 42:**
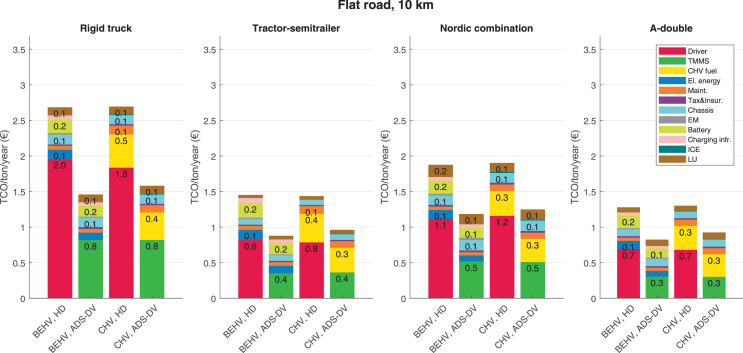
Flat road of 10 km length; the optimized TCO components of different vehicles and driving systems are shown for the optimum speed of the transportation scenario. See Table 5 for the other road types.

**Fig. 43:**
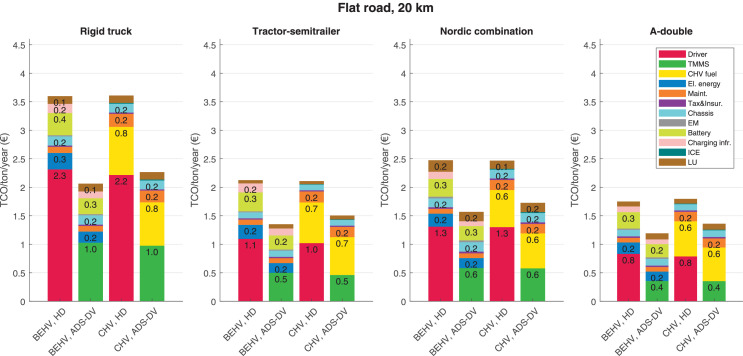
Flat road of 20 km length; the optimized TCO components of different vehicles and driving systems are shown for the optimum speed of the transportation scenario. See Table 5 for the other road types.

**Fig. 44:**
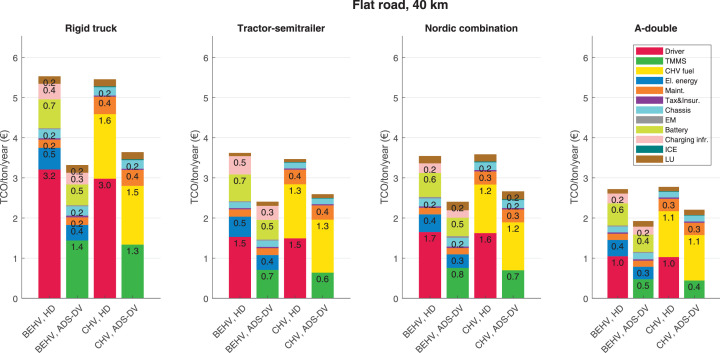
Flat road of 40 km length; the optimized TCO components of different vehicles and driving systems are shown for the optimum speed of the transportation scenario. See Table 5 for the other road types.

**Fig. 45:**
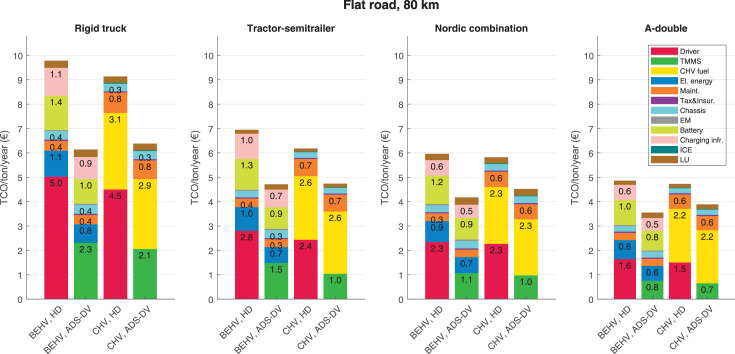
Flat road of 80 km length; the optimized TCO components of different vehicles and driving systems are shown for the optimum speed of the transportation scenario. See Table 5 for the other road types.

**Fig. 46:**
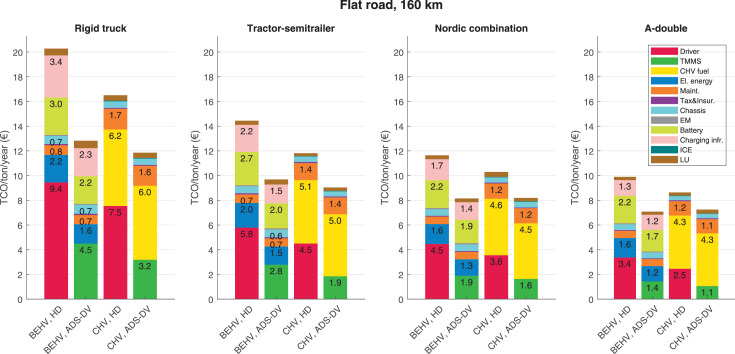
Flat road of 160 km length; the optimized TCO components of different vehicles and driving systems are shown for the optimum speed of the transportation scenario. See Table 5 for the other road types.

**Fig. 47:**
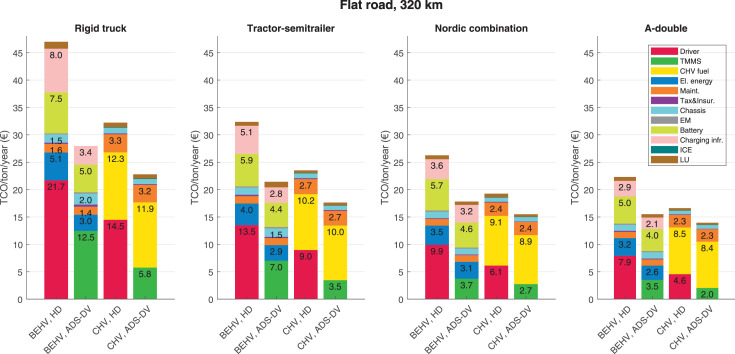
Flat road of 320 km length; the optimized TCO components of different vehicles and driving systems are shown for the optimum speed of the transportation scenario. See Table 5 for the other road types.

**Fig. 48:**
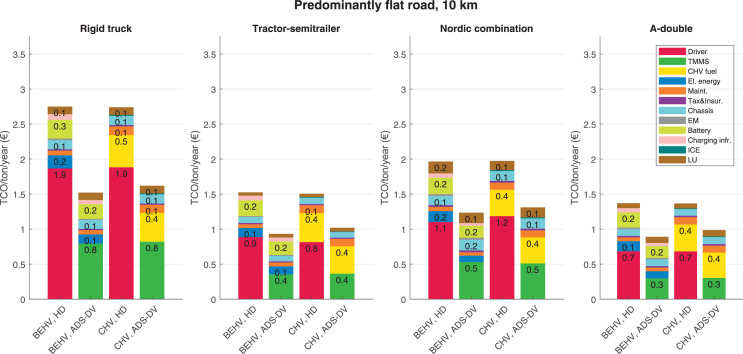
Predominantly flat road of 10 km length; the optimized TCO components of different vehicles and driving systems are shown for the optimum speed of the transportation scenario. See Table 5 for the other road types.

**Fig. 49:**
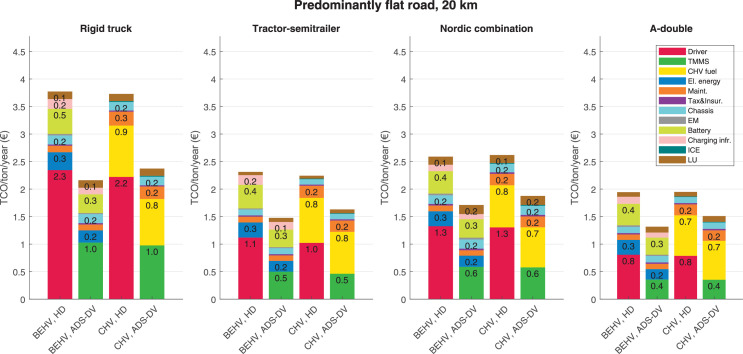
Predominantly flat road of 20 km length; the optimized TCO components of different vehicles and driving systems are shown for the optimum speed of the transportation scenario. See Table 5 for the other road types.

**Fig. 50:**
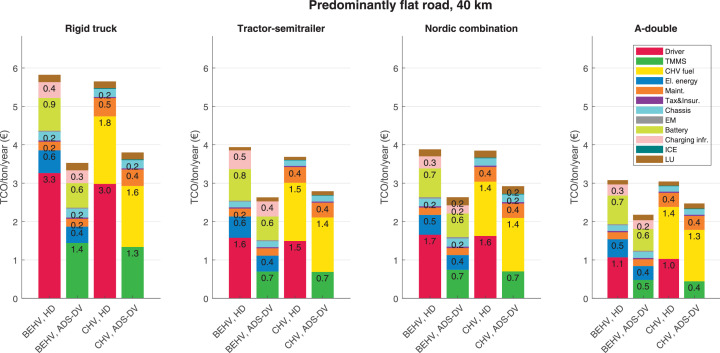
Predominantly flat road of 40 km length; the optimized TCO components of different vehicles and driving systems are shown for the optimum speed of the transportation scenario. See Table 5 for the other road types.

**Fig. 51:**
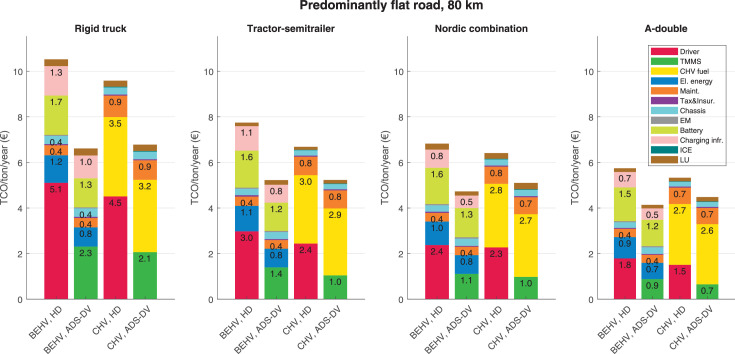
Predominantly flat road of 80 km length; the optimized TCO components of different vehicles and driving systems are shown for the optimum speed of the transportation scenario. See Table 5 for the other road types.

**Fig. 52:**
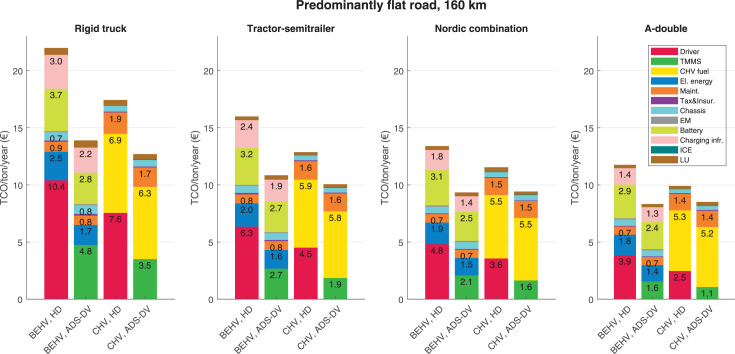
Predominantly flat road of 160 km length; the optimized TCO components of different vehicles and driving systems are shown for the optimum speed of the transportation scenario. See Table 5 for the other road types.

**Fig. 53:**
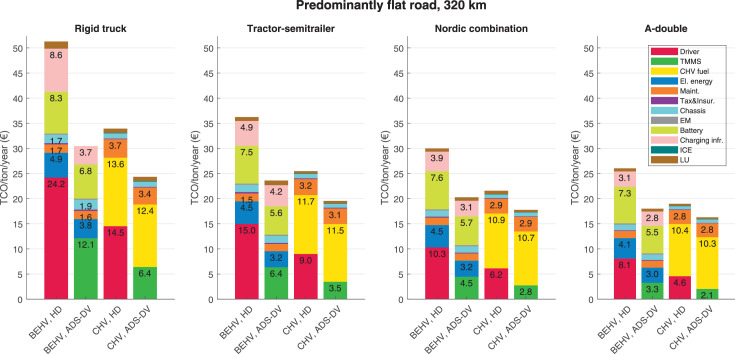
Predominantly flat road of 320 km length; the optimized TCO components of different vehicles and driving systems are shown for the optimum speed of the transportation scenario. See Table 5 for the other road types.

**Fig. 54:**
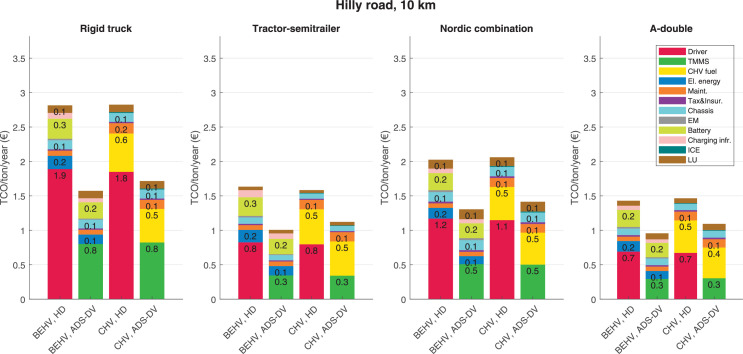
Hilly road of 10 km length; the optimized TCO components of different vehicles and driving systems are shown for the optimum speed of the transportation scenario. See Table 5 for the other road types.

**Fig. 55:**
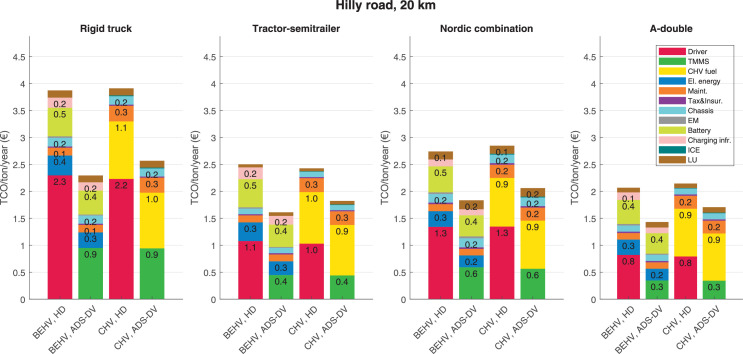
Hilly road of 20 km length; the optimized TCO components of different vehicles and driving systems are shown for the optimum speed of the transportation scenario. See Table 5 for the other road types.

**Fig. 56:**
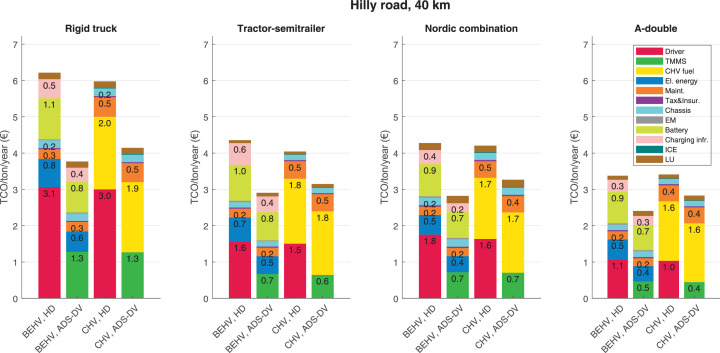
Hilly road of 40 km length; the optimized TCO components of different vehicles and driving systems are shown for the optimum speed of the transportation scenario. See Table 5 for the other road types.

**Fig. 57:**
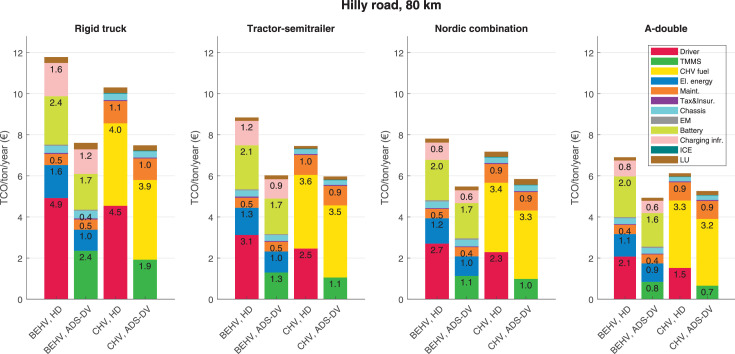
Hilly road of 80 km length; the optimized TCO components of different vehicles and driving systems are shown for the optimum speed of the transportation scenario. See Table 5 for the other road types.

**Fig. 58:**
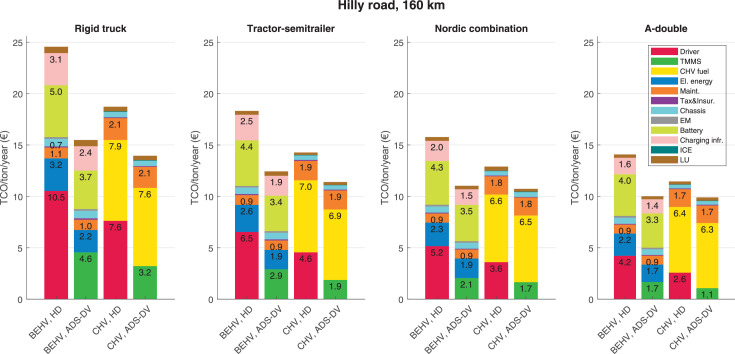
Hilly road of 160 km length; the optimized TCO components of different vehicles and driving systems are shown for the optimum speed of the transportation scenario. See Table 5 for the other road types.

**Fig. 59:**
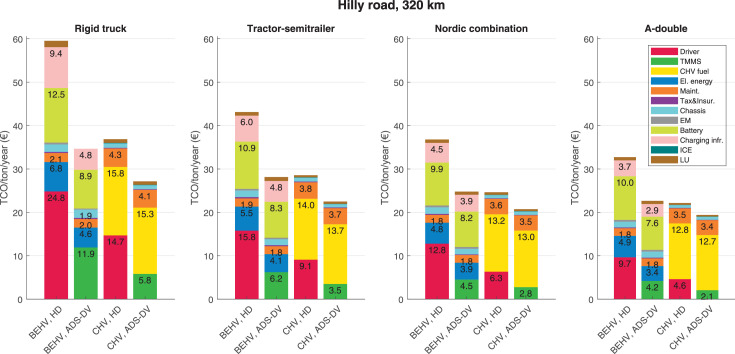
Hilly road of 320 km length; the optimized TCO components of different vehicles and driving systems are shown for the optimum speed of the transportation scenario. See Table 5 for the other road types.

**Fig. 60:**
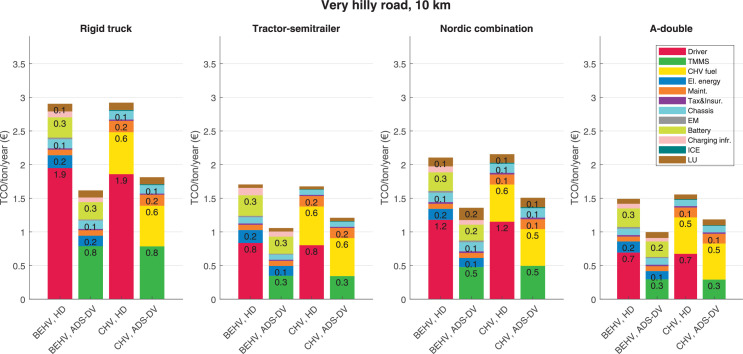
Very hilly road of 10 km length; the optimized TCO components of different vehicles and driving systems are shown for the optimum speed of the transportation scenario. See Table 5 for the other road types.

**Fig. 61:**
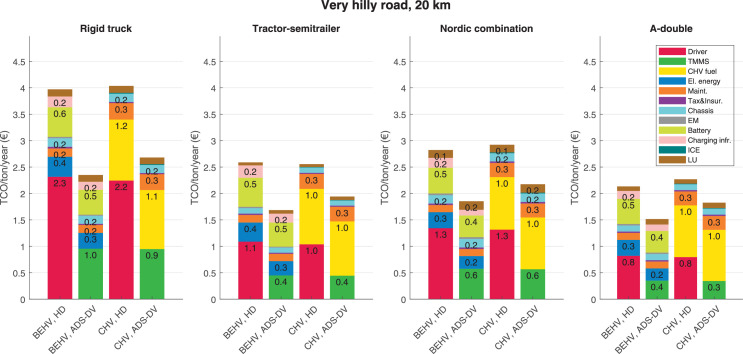
Very hilly road of 20 km length; the optimized TCO components of different vehicles and driving systems are shown for the optimum speed of the transportation scenario. See Table 5 for the other road types.

**Fig. 62:**
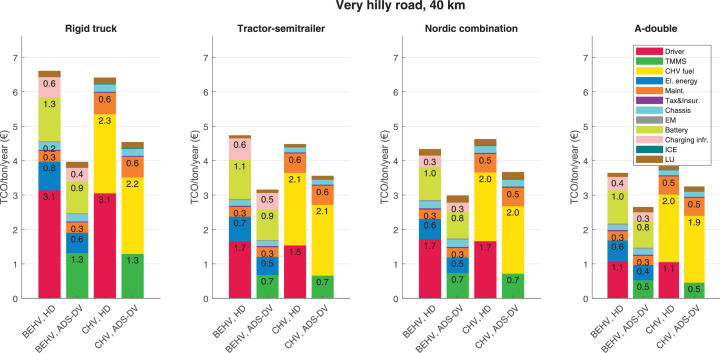
Very hilly road of 40 km length; the optimized TCO components of different vehicles and driving systems are shown for the optimum speed of the transportation scenario. See Table 5 for the other road types.

**Fig. 63:**
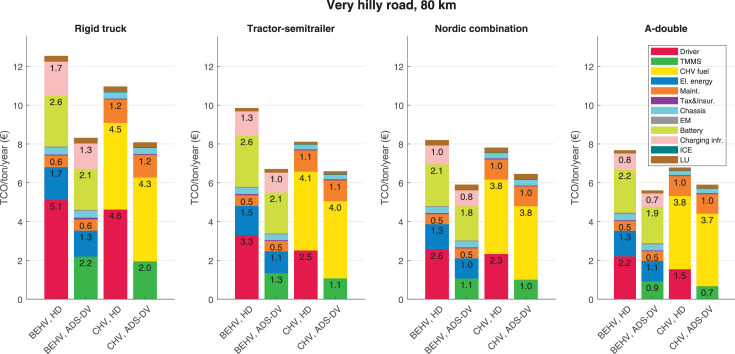
Very hilly road of 80 km length; the optimized TCO components of different vehicles and driving systems are shown for the optimum speed of the transportation scenario. See Table 5 for the other road types.

**Fig. 64:**
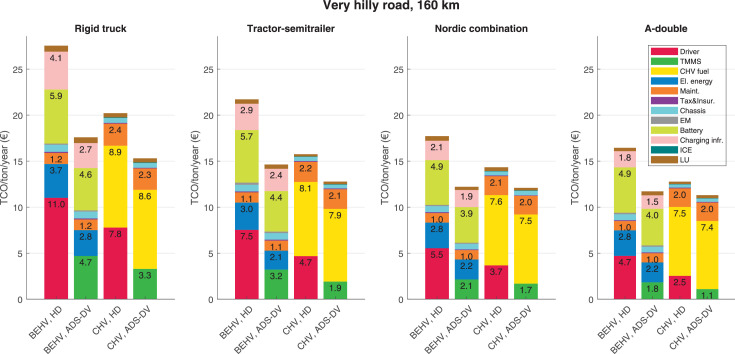
Very hilly road of 160 km length; the optimized TCO components of different vehicles and driving systems are shown for the optimum speed of the transportation scenario. See Table 5 for the other road types.

**Fig. 65:**
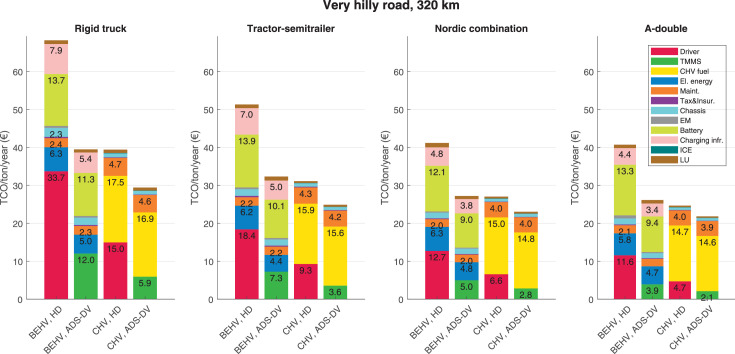
Very hilly road of 320 km length; the optimized TCO components of different vehicles and driving systems are shown for the optimum speed of the transportation scenario. See Table 5 for the other road types.

**Fig. 66:**
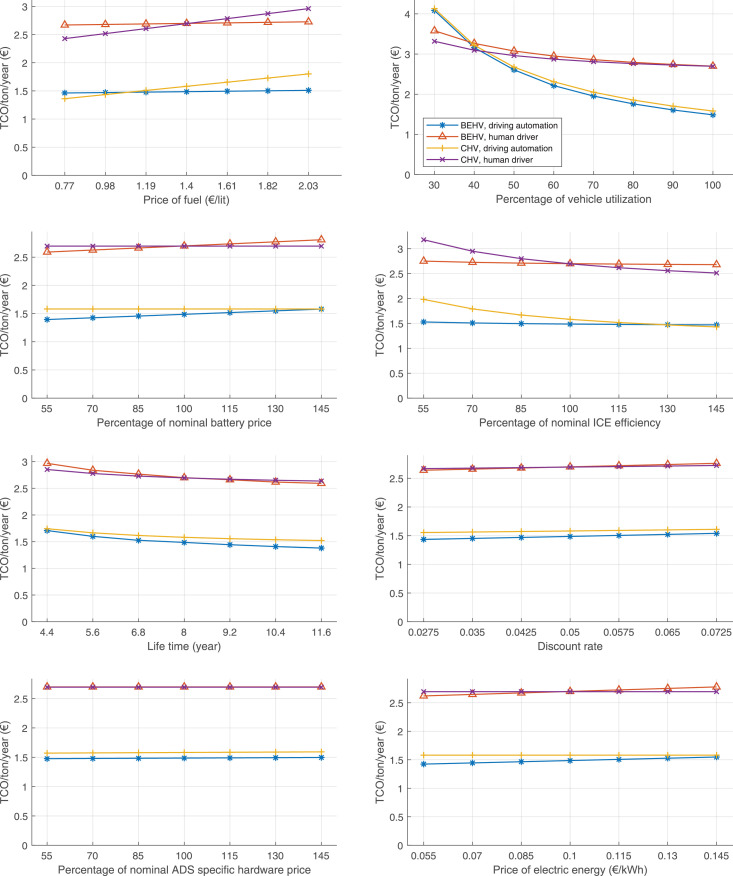
Rigid truck on a flat road of 10 km length; sensitivity of TCO to different parameters are shown for the optimum speed of the transportation scenario. See Table 6 for the other vehicle sizes and road types.

**Fig. 67:**
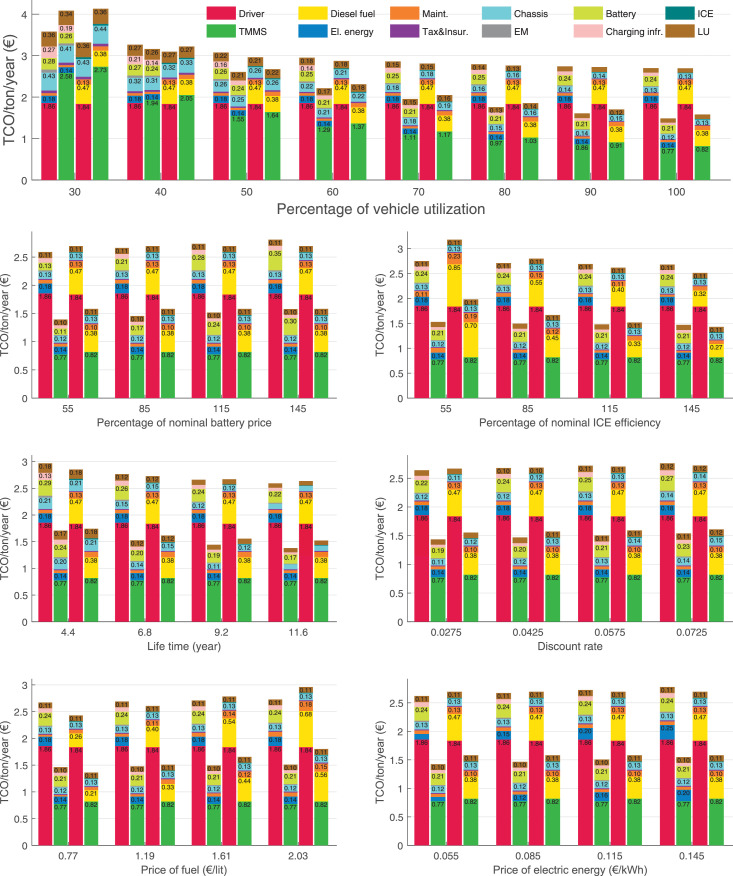
Rigid truck on a flat road of 10 km length; sensitivity of the TCO components of different vehicles and driving systems are shown for the optimum speed of the transportation scenario. A group of four bars from left to right represent BEHV HD, BEHV ADS-V, CHV HD and CHV ADS-DV, respectively. See Table 6 for the other vehicle sizes and road types.

**Fig. 68:**
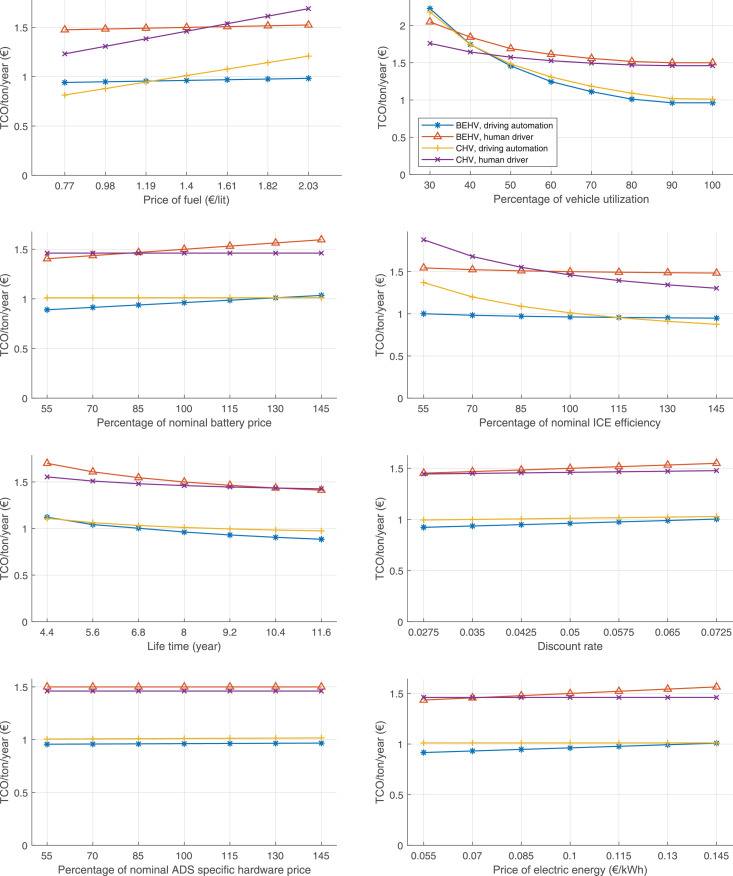
Tractor-semitrailer on a flat road of 10 km length; sensitivity of TCO to different parameters are shown for the optimum speed of the transportation scenario. See Table 6 for the other vehicle sizes and road types.

**Fig. 69:**
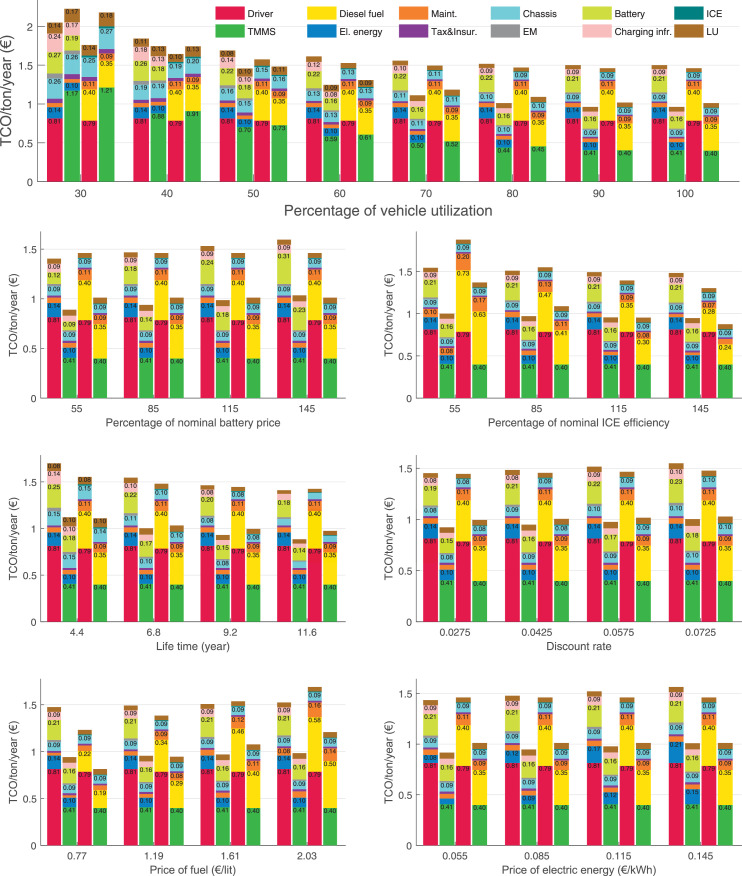
Tractor-semitrailer on a flat road of 10 km length; sensitivity of the TCO components of different vehicles and driving systems are shown for the optimum speed of the transportation scenario. A group of four bars from left to right represent BEHV HD, BEHV ADS-V, CHV HD and CHV ADS-DV, respectively. See Table 6 for the other vehicle sizes and road types.

**Fig. 70:**
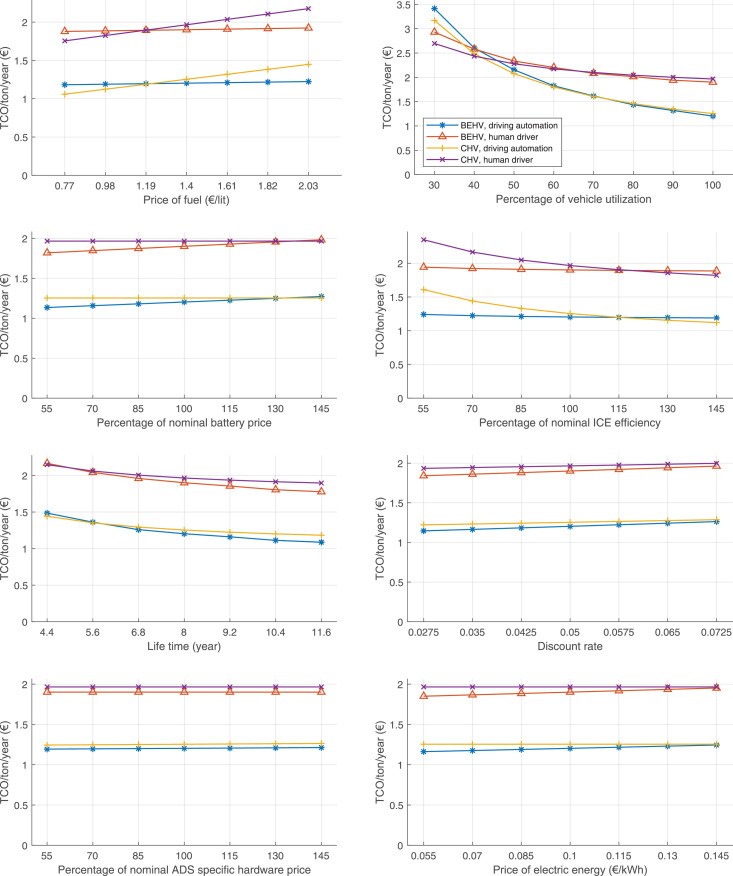
Nordic combination on a flat road of 10 km length; sensitivity of TCO to different parameters are shown for the optimum speed of the transportation scenario. See Table 6 for the other vehicle sizes and road types.

**Fig. 71:**
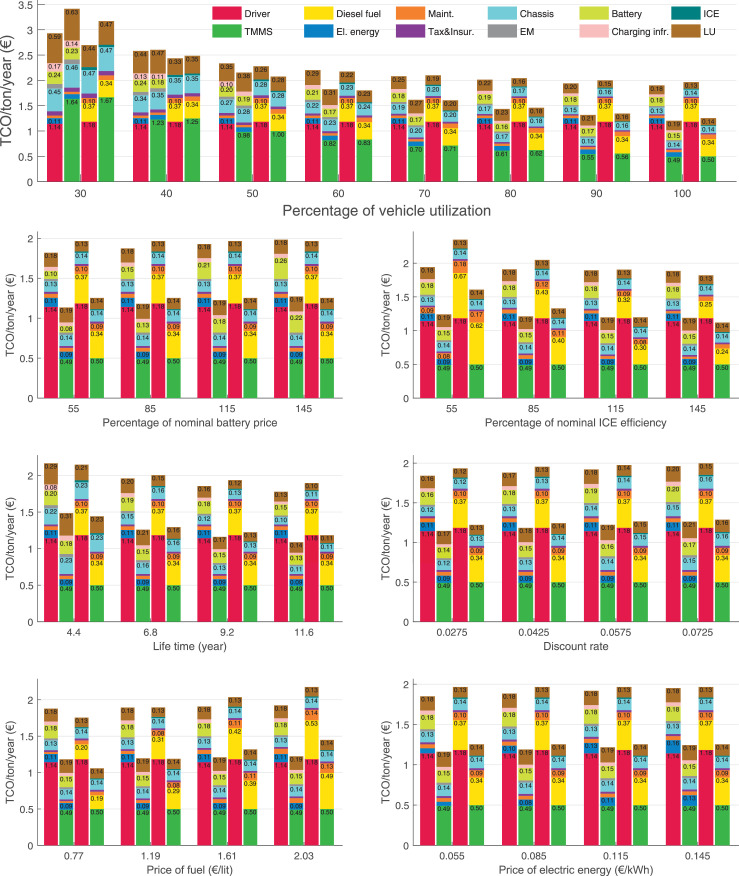
Nordic combination on a flat road of 10 km length; sensitivity of the TCO components of different vehicles and driving systems are shown for the optimum speed of the transportation scenario. A group of four bars from left to right represent BEHV HD, BEHV ADS-V, CHV HD and CHV ADS-DV, respectively. See Table 6 for the other vehicle sizes and road types.

**Fig. 72:**
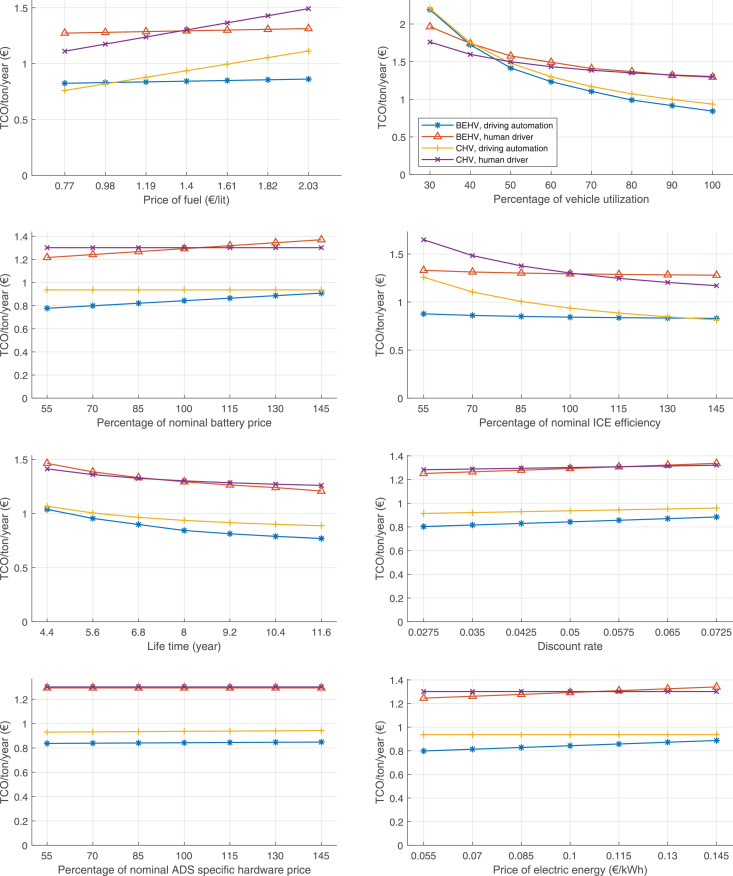
A-double on a flat road of 10 km length; sensitivity of TCO to different parameters are shown for the optimum speed of the transportation scenario. See Table 6 for the other vehicle sizes and road types.

**Fig. 73:**
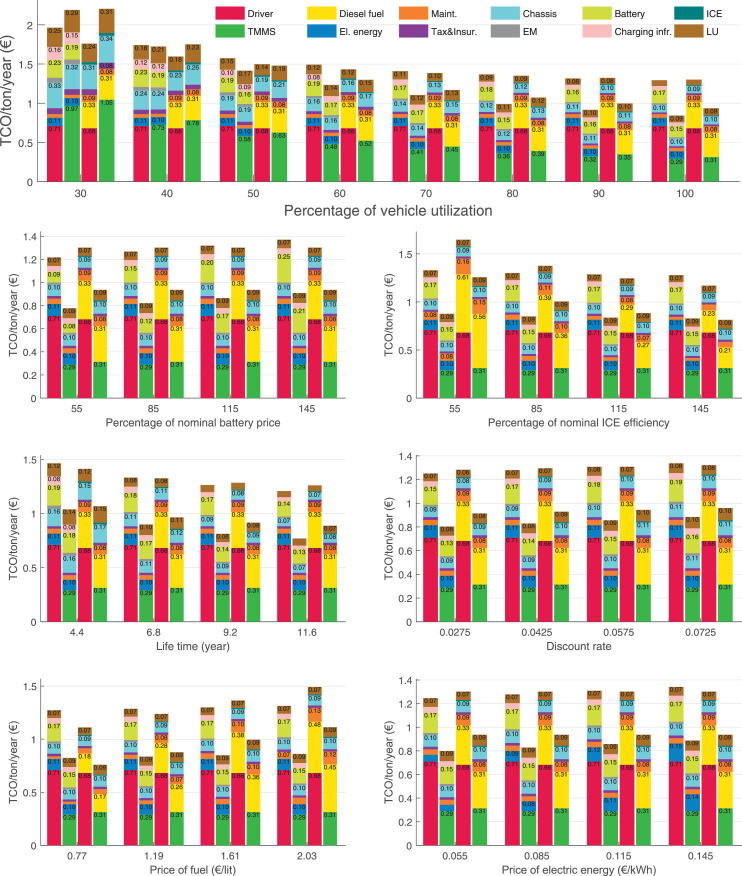
A-double on a flat road of 10 km length; sensitivity of the TCO components of different vehicles and driving systems are shown for the optimum speed of the transportation scenario. A group of four bars from left to right represent BEHV HD, BEHV ADS-V, CHV HD and CHV ADS-DV, respectively. See Table 6 for the other vehicle sizes and road types.

**Fig. 74:**
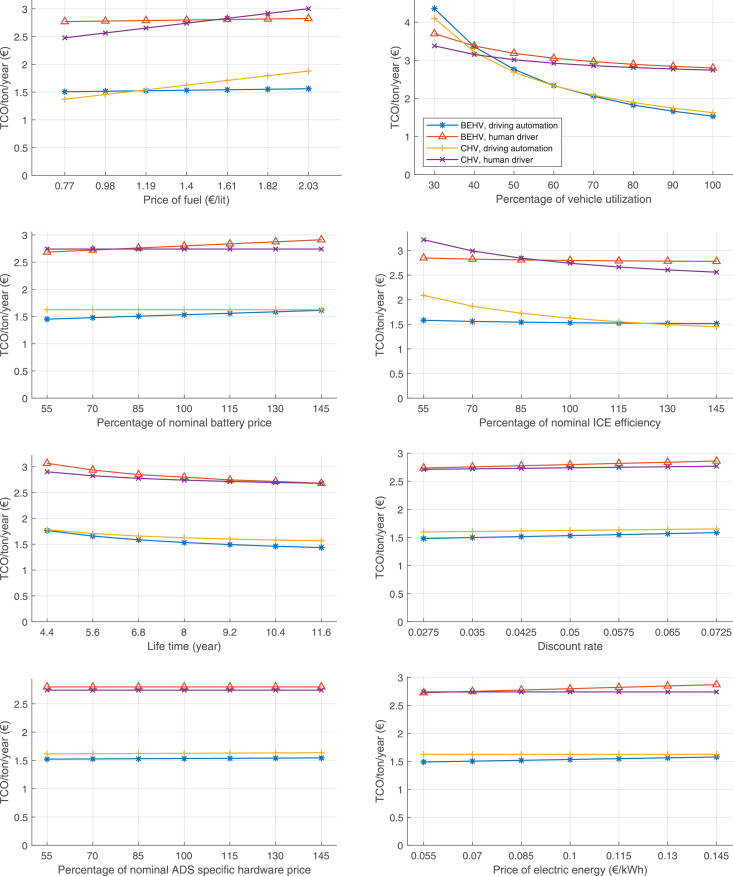
Rigid truck on a predominantly flat road of 10 km length; sensitivity of TCO to different parameters are shown for the optimum speed of the transportation scenario. See Table 6 for the other vehicle sizes and road types.

**Fig. 75:**
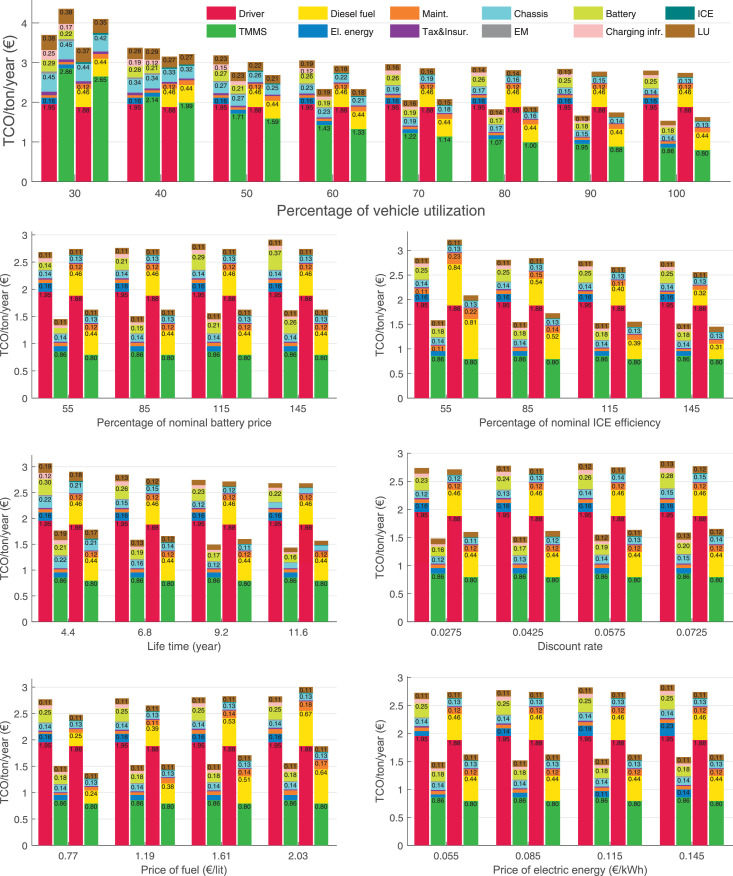
Rigid truck on a predominantly flat road of 10 km length; sensitivity of the TCO components of different vehicles and driving systems are shown for the optimum speed of the transportation scenario. A group of four bars from left to right represent BEHV HD, BEHV ADS-V, CHV HD and CHV ADS-DV, respectively. See Table 6 for the other vehicle sizes and road types.

**Fig. 76:**
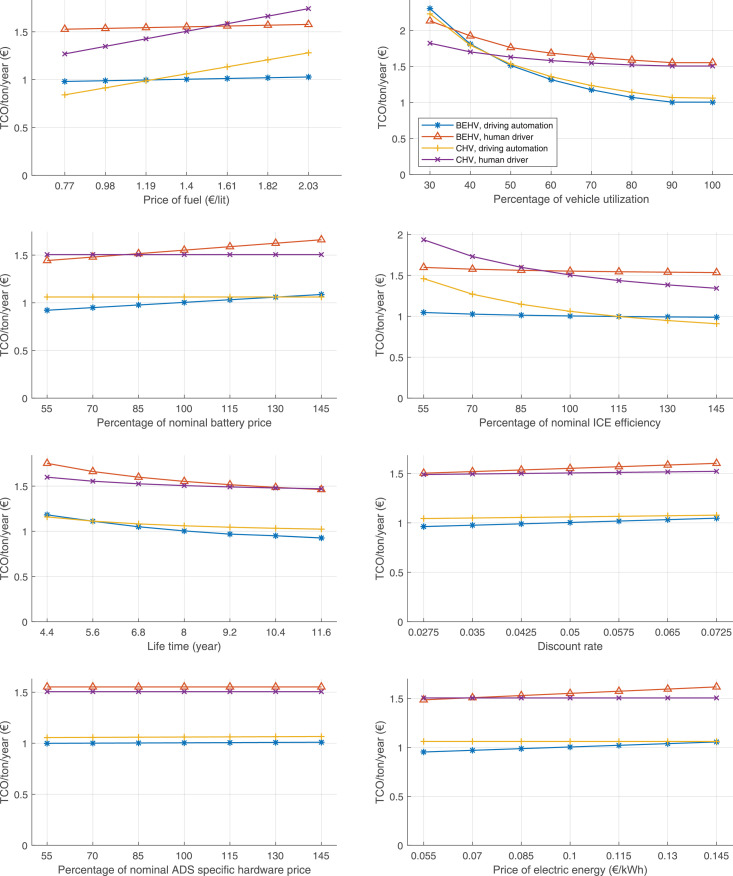
Tractor-semitrailer on a predominantly flat road of 10 km length; sensitivity of TCO to different parameters are shown for the optimum speed of the transportation scenario. See Table 6 for the other vehicle sizes and road types.

**Fig. 77:**
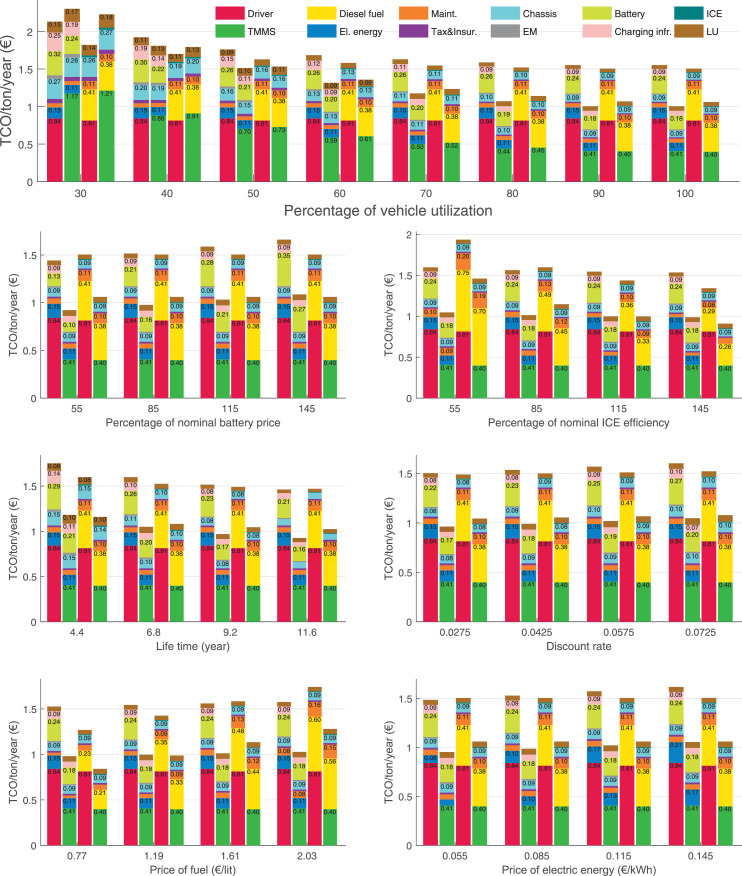
Tractor-semitrailer on a predominantly flat road of 10 km length; sensitivity of the TCO components of different vehicles and driving systems are shown for the optimum speed of the transportation scenario. A group of four bars from left to right represent BEHV HD, BEHV ADS-V, CHV HD and CHV ADS-DV, respectively. See Table 6 for the other vehicle sizes and road types.

**Fig. 78:**
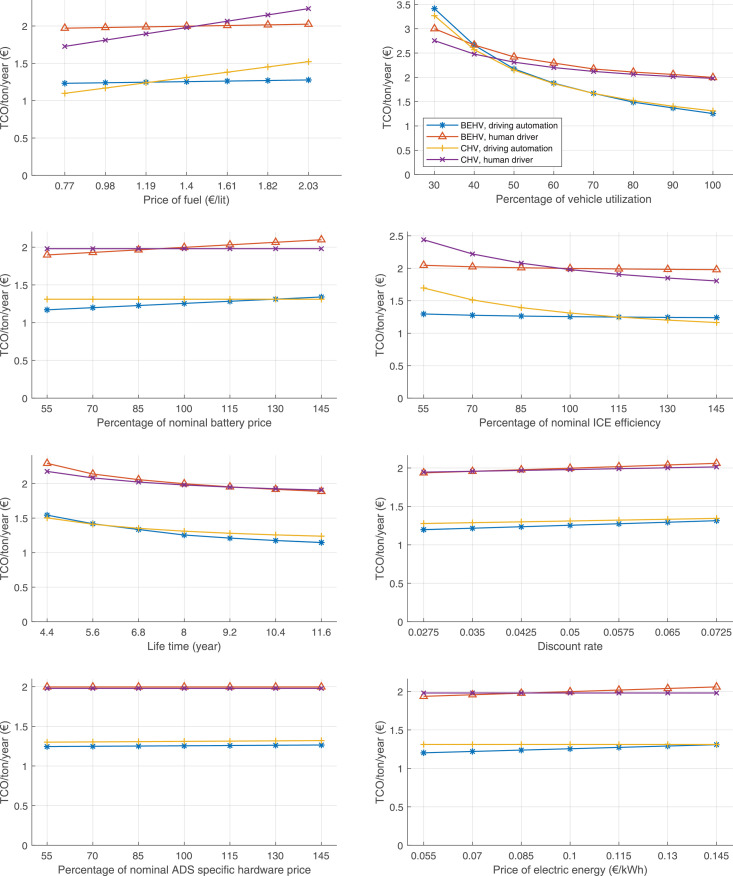
Nordic combination on a predominantly flat road of 10 km length; sensitivity of TCO to different parameters are shown for the optimum speed of the transportation scenario. See Table 6 for the other vehicle sizes and road types.

**Fig. 79:**
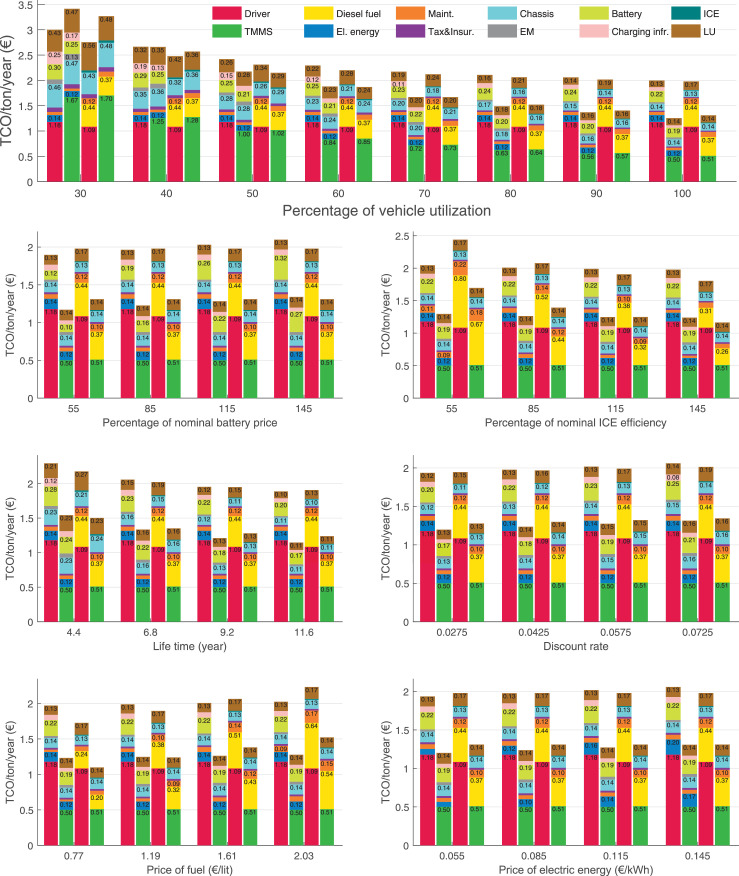
Nordic combination on a predominantly flat road of 10 km length; sensitivity of the TCO components of different vehicles and driving systems are shown for the optimum speed of the transportation scenario. A group of four bars from left to right represent BEHV HD, BEHV ADS-V, CHV HD and CHV ADS-DV, respectively. See Table 6 for the other vehicle sizes and road types.

**Fig. 80:**
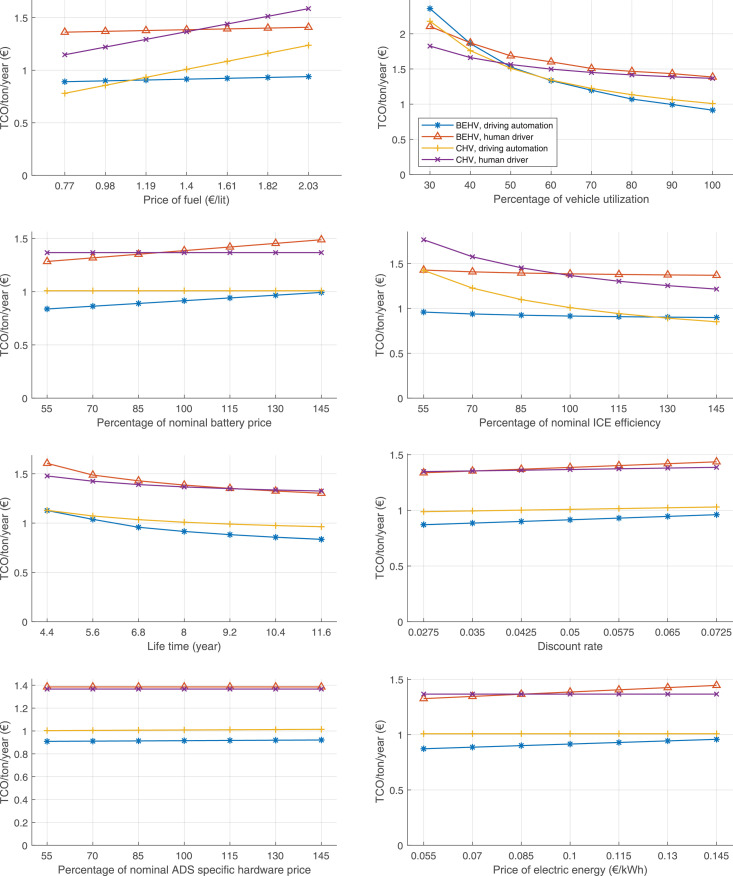
A-double on a predominantly flat road of 10 km length; sensitivity of TCO to different parameters are shown for the optimum speed of the transportation scenario. See Table 6 for the other vehicle sizes and road types.

**Fig. 81:**
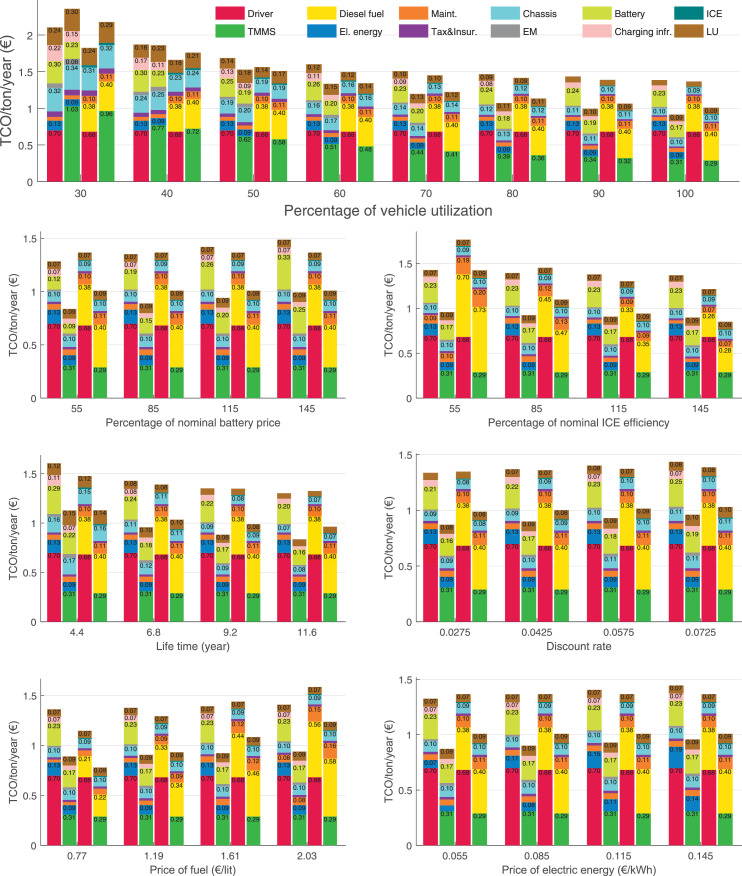
A-double on a predominantly flat road of 10 km length; sensitivity of the TCO components of different vehicles and driving systems are shown for the optimum speed of the transportation scenario. A group of four bars from left to right represent BEHV HD, BEHV ADS-V, CHV HD and CHV ADS-DV, respectively. See Table 6 for the other vehicle sizes and road types.

**Fig. 82:**
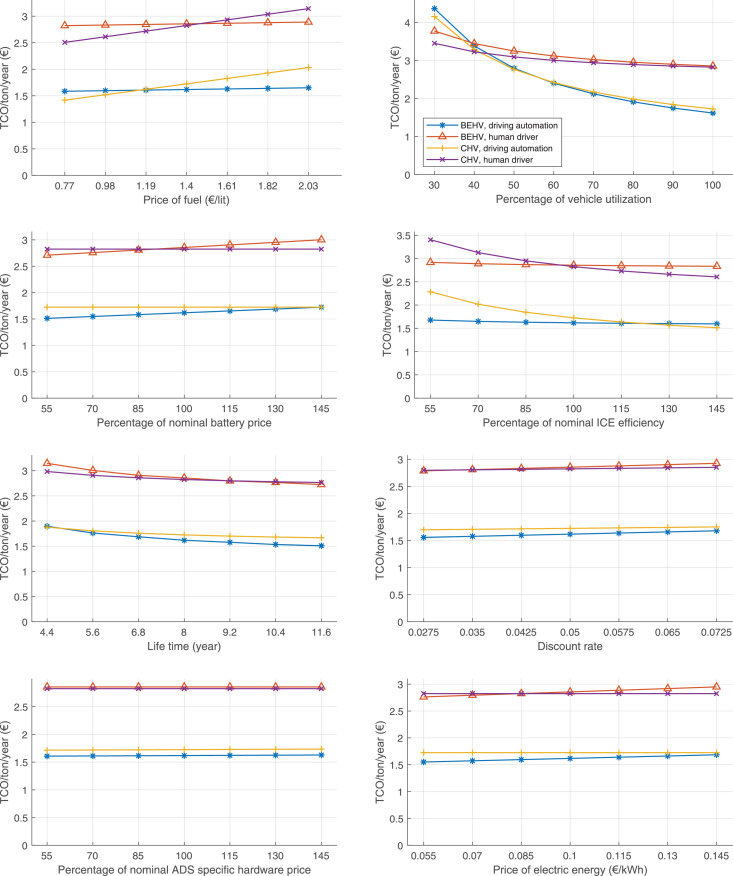
Rigid truck on a hilly road of 10 km length; sensitivity of TCO to different parameters are shown for the optimum speed of the transportation scenario. See Table 6 for the other vehicle sizes and road types.

**Fig. 83:**
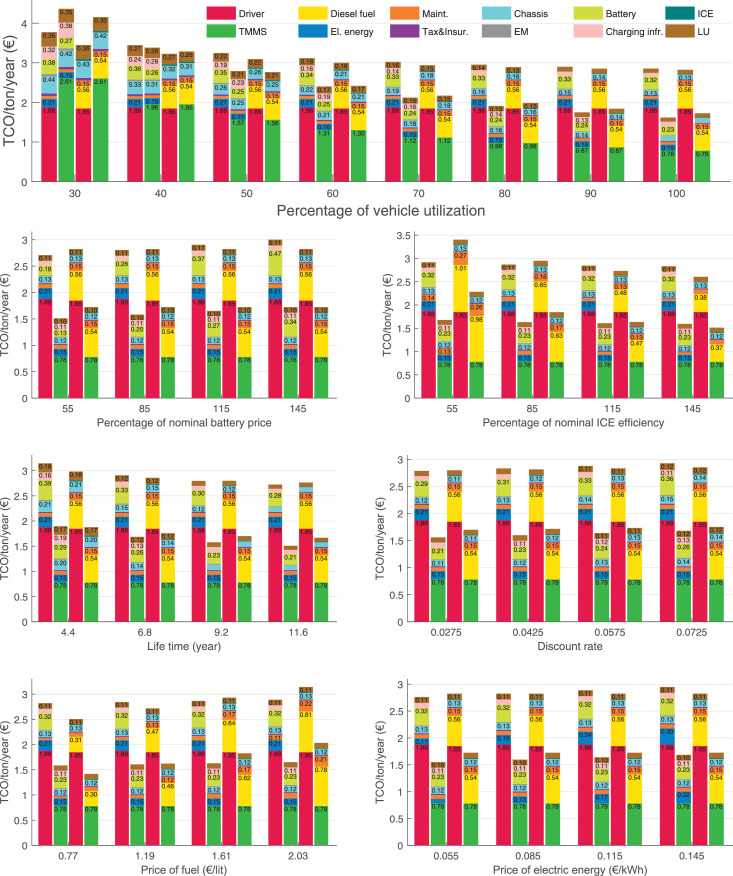
Rigid truck on a hilly road of 10 km length; sensitivity of the TCO components of different vehicles and driving systems are shown for the optimum speed of the transportation scenario. A group of four bars from left to right represent BEHV HD, BEHV ADS-V, CHV HD and CHV ADS-DV, respectively. See Table 6 for the other vehicle sizes and road types.

**Fig. 84:**
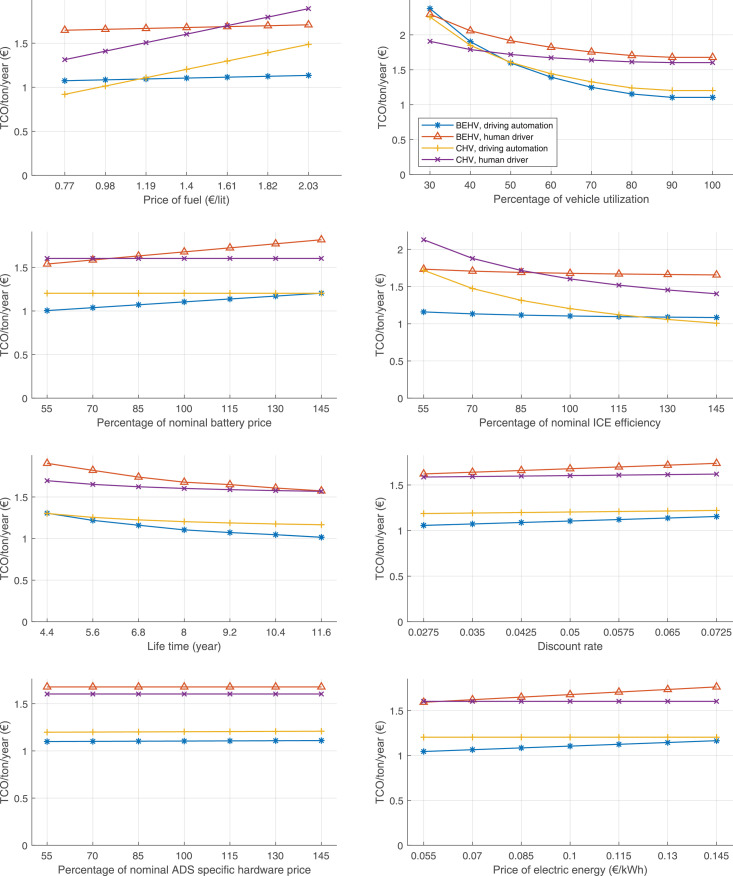
Tractor-semitrailer on a hilly road of 10 km length; sensitivity of TCO to different parameters are shown for the optimum speed of the transportation scenario. See Table 6 for the other vehicle sizes and road types.

**Fig. 85:**
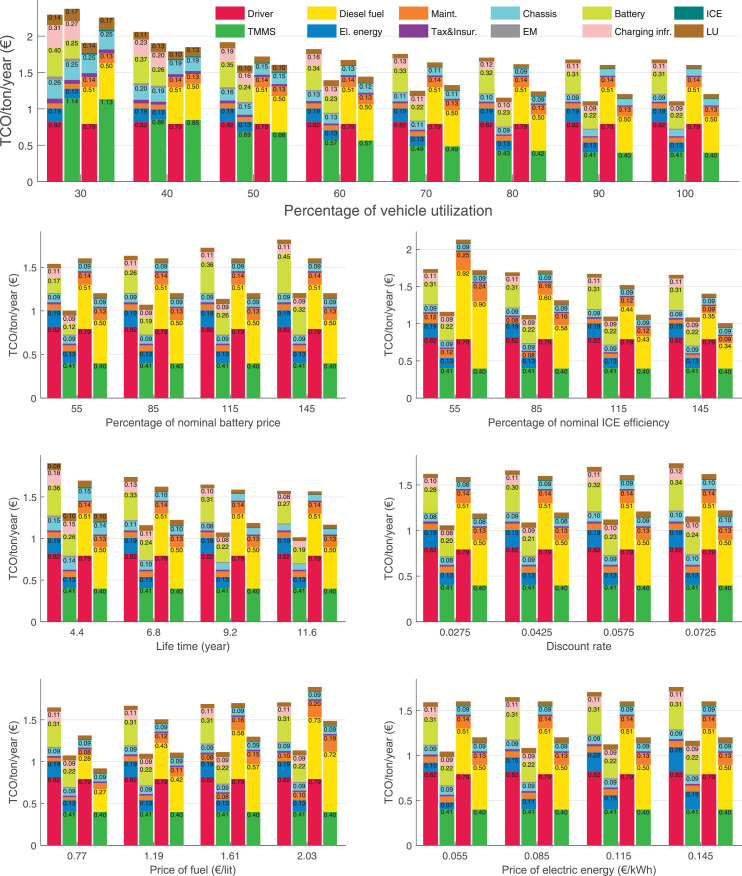
Tractor-semitrailer on a hilly road of 10 km length; sensitivity of the TCO components of different vehicles and driving systems are shown for the optimum speed of the transportation scenario. A group of four bars from left to right represent BEHV HD, BEHV ADS-V, CHV HD and CHV ADS-DV, respectively. See Table 6 for the other vehicle sizes and road types.

**Fig. 86:**
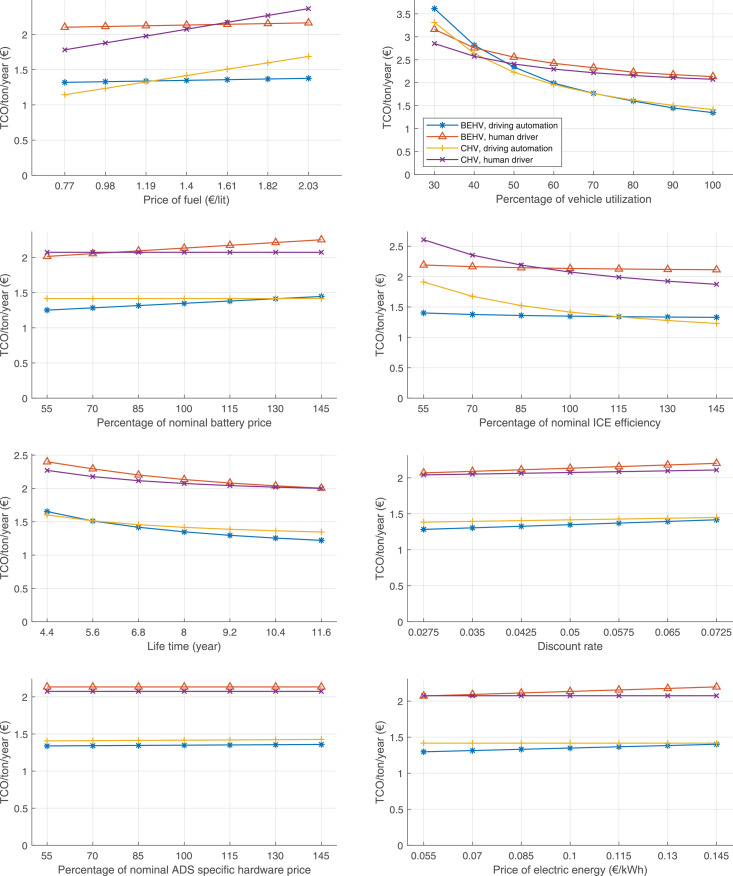
Nordic combination on a hilly road of 10 km length; sensitivity of TCO to different parameters are shown for the optimum speed of the transportation scenario. See Table 6 for the other vehicle sizes and road types.

**Fig. 87:**
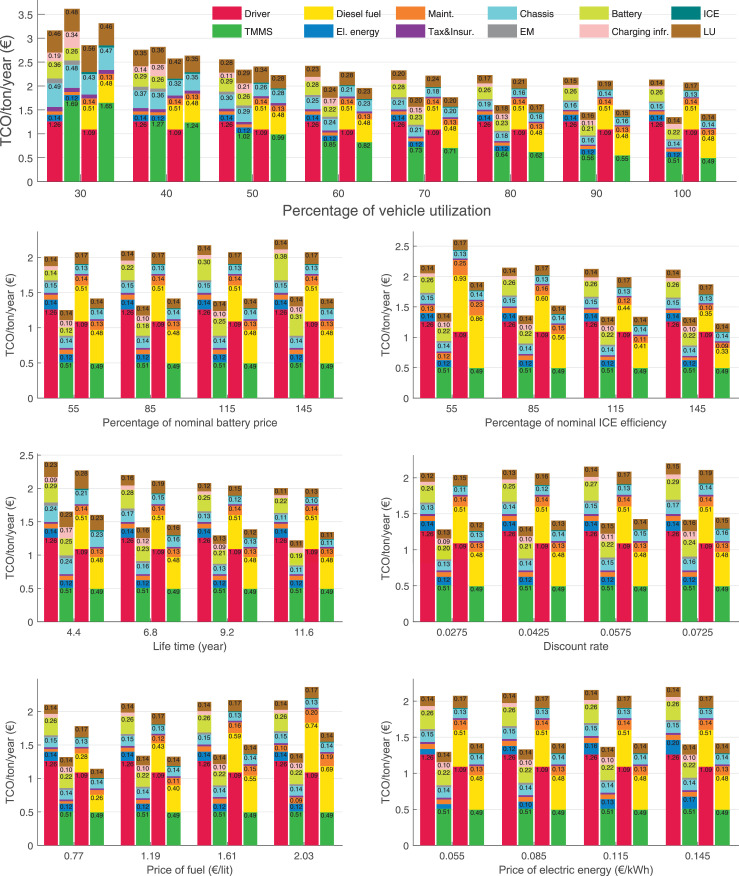
Nordic combination on a hilly road of 10 km length; sensitivity of the TCO components of different vehicles and driving systems are shown for the optimum speed of the transportation scenario. A group of four bars from left to right represent BEHV HD, BEHV ADS-V, CHV HD and CHV ADS-DV, respectively. See Table 6 for the other vehicle sizes and road types.

**Fig. 88:**
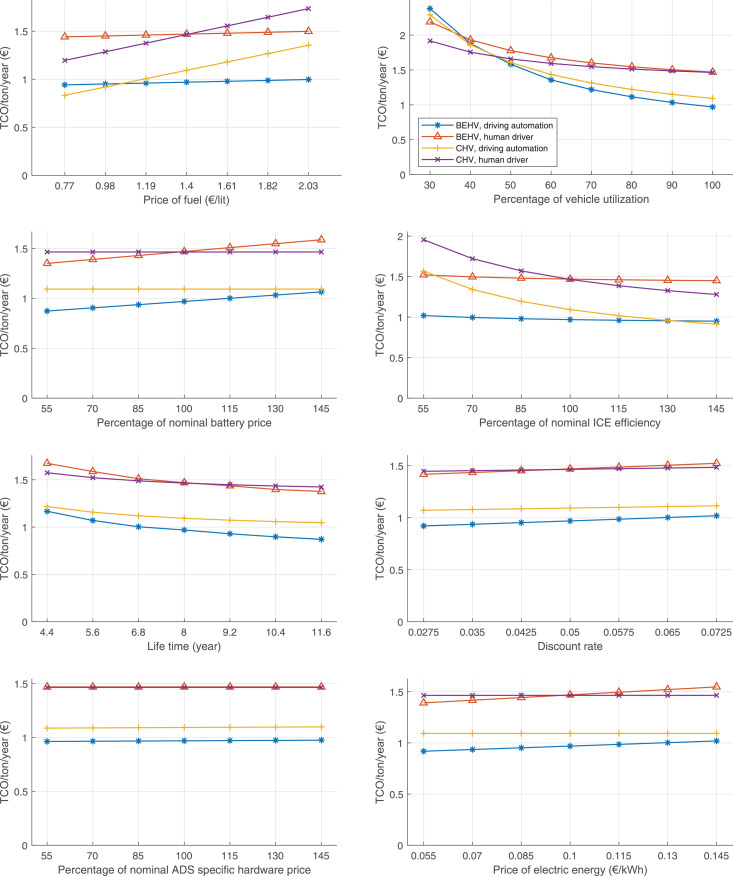
A-double on a hilly road of 10 km length; sensitivity of TCO to different parameters are shown for the optimum speed of the transportation scenario. See Table 6 for the other vehicle sizes and road types.

**Fig. 89:**
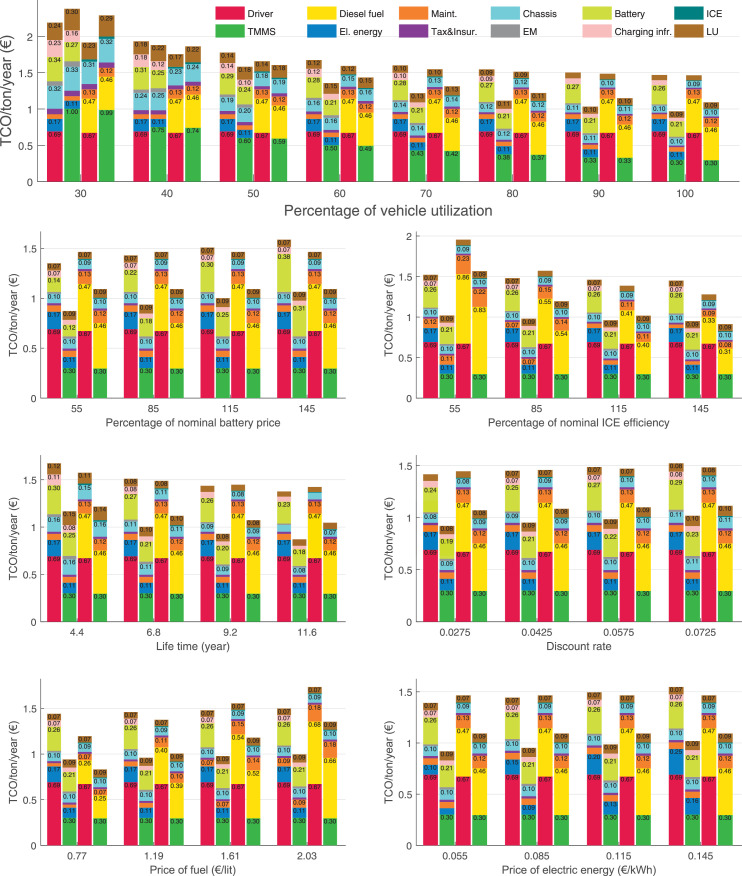
A-double on a hilly road of 10 km length; sensitivity of the TCO components of different vehicles and driving systems are shown for the optimum speed of the transportation scenario. A group of four bars from left to right represent BEHV HD, BEHV ADS-V, CHV HD and CHV ADS-DV, respectively. See Table 6 for the other vehicle sizes and road types.

**Fig. 90:**
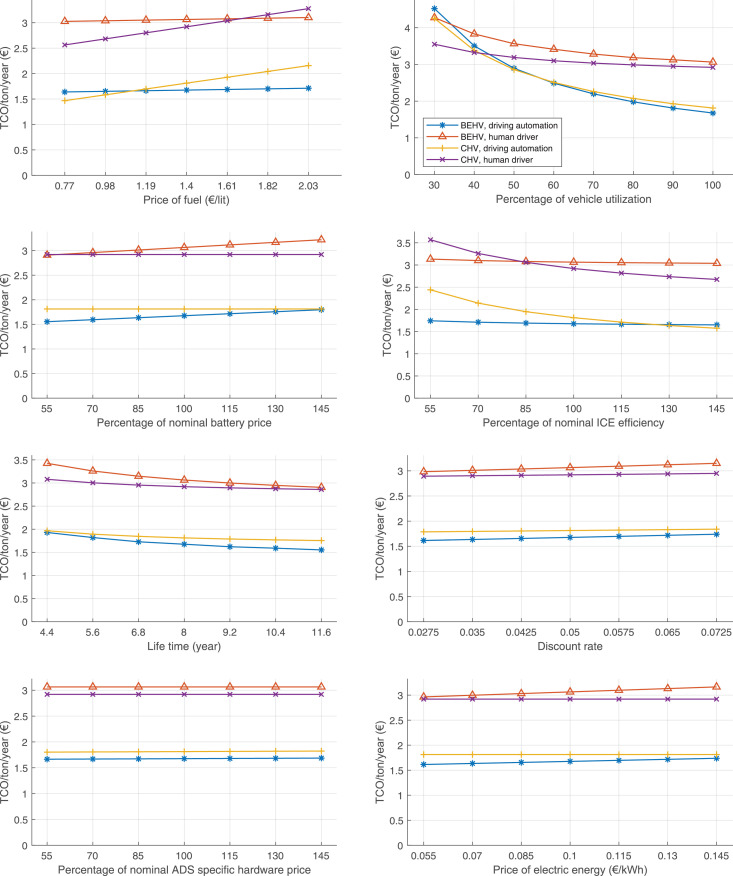
Rigid truck on a very hilly road of 10 km length; sensitivity of TCO to different parameters are shown for the optimum speed of the transportation scenario. See Table 6 for the other vehicle sizes and road types.

**Fig. 91:**
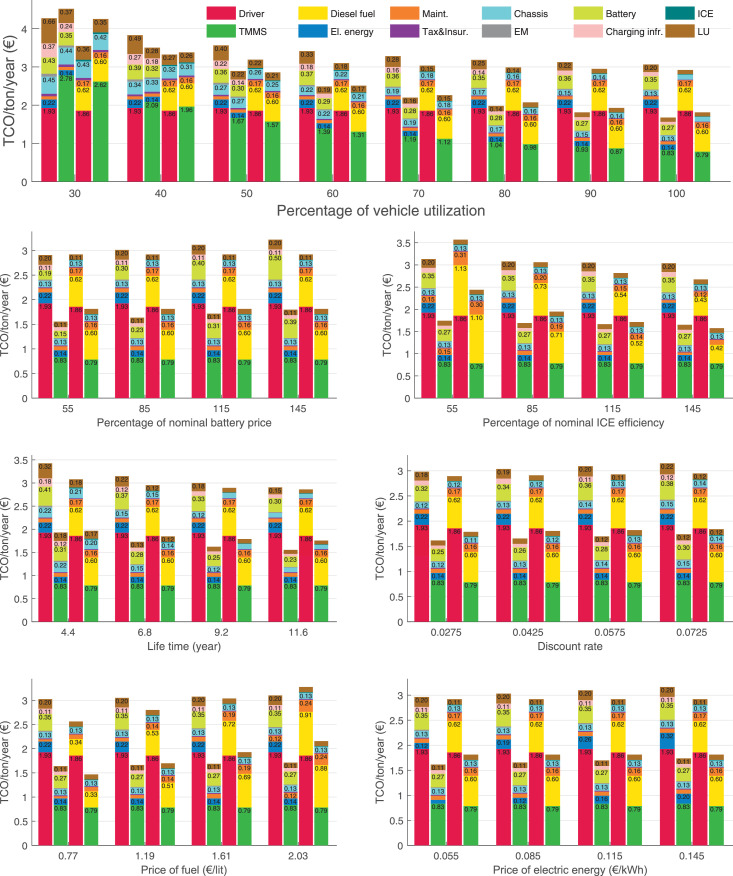
Rigid truck on a very hilly road of 10 km length; sensitivity of the TCO components of different vehicles and driving systems are shown for the optimum speed of the transportation scenario. A group of four bars from left to right represent BEHV HD, BEHV ADS-V, CHV HD and CHV ADS-DV, respectively. See Table 6 for the other vehicle sizes and road types.

**Fig. 92:**
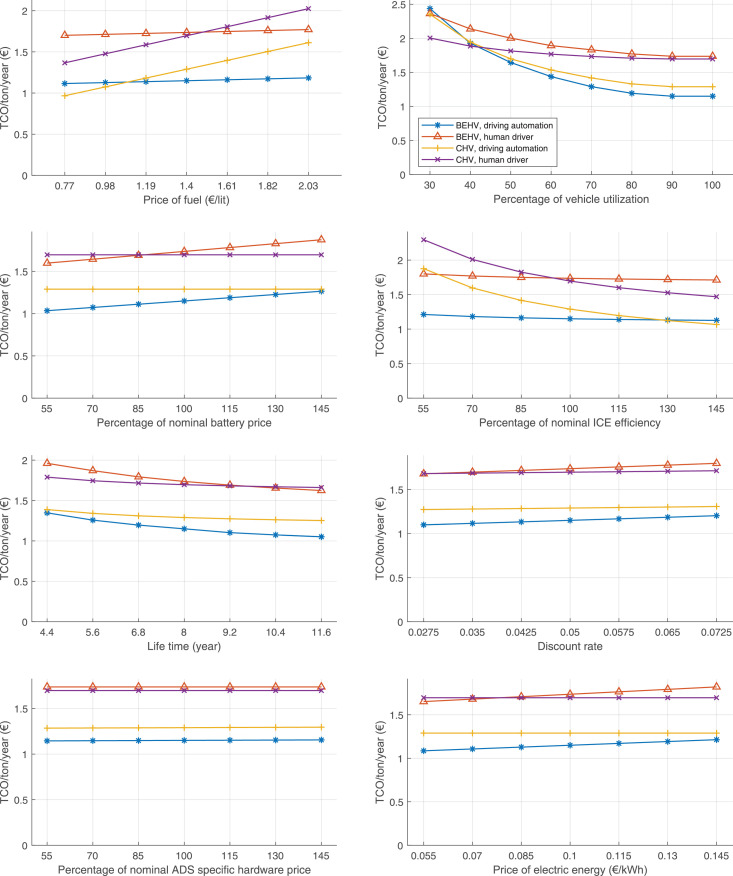
Tractor-semitrailer on a very hilly road of 10 km length; sensitivity of TCO to different parameters are shown for the optimum speed of the transportation scenario. See Table 6 for the other vehicle sizes and road types.

**Fig. 93:**
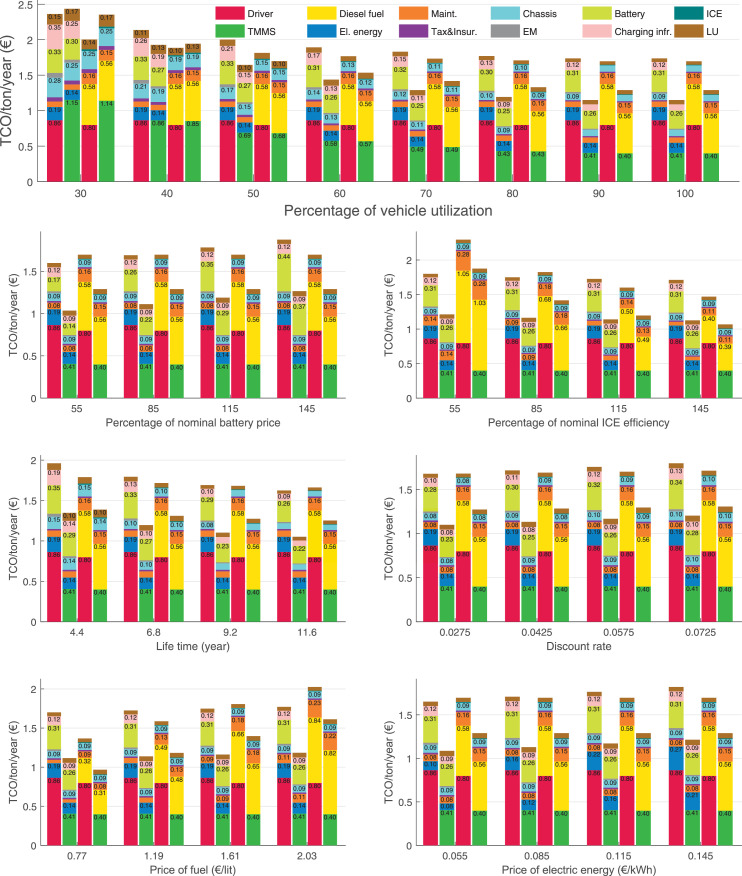
Tractor-semitrailer on a very hilly road of 10 km length; sensitivity of the TCO components of different vehicles and driving systems are shown for the optimum speed of the transportation scenario. A group of four bars from left to right represent BEHV HD, BEHV ADS-V, CHV HD and CHV ADS-DV, respectively. See Table 6 for the other vehicle sizes and road types.

**Fig. 94:**
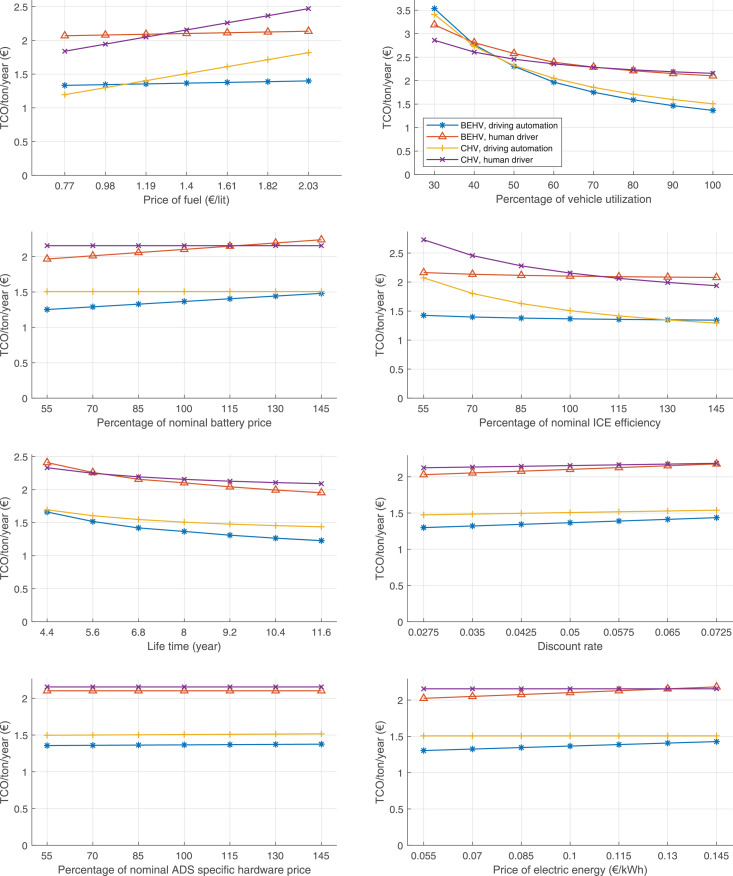
Nordic combination on a very hilly road of 10 km length; sensitivity of TCO to different parameters are shown for the optimum speed of the transportation scenario. See Table 6 for the other vehicle sizes and road types.

**Fig. 95:**
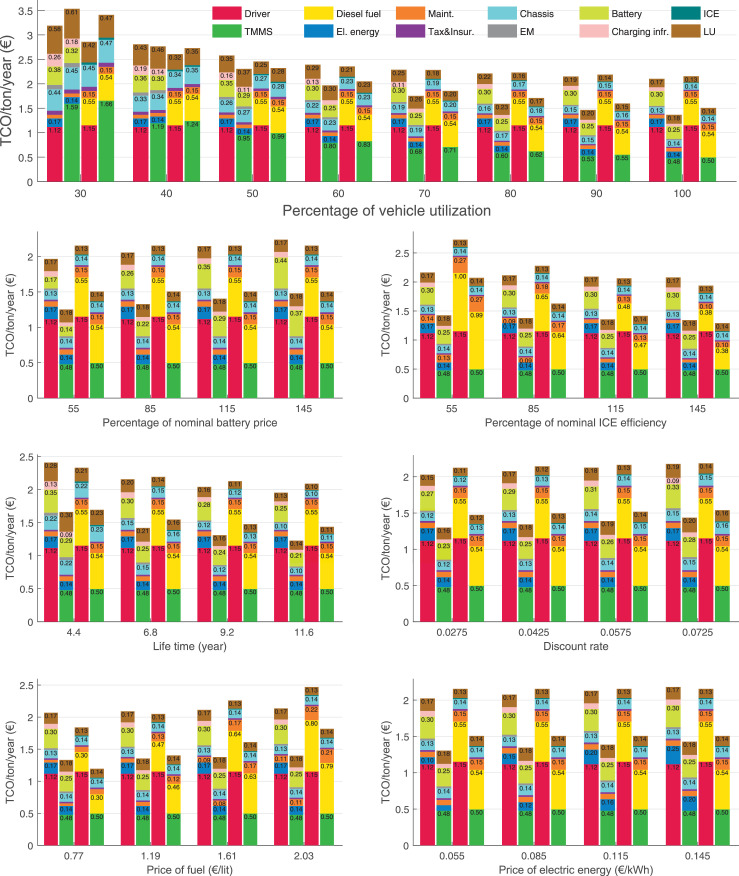
Nordic combination on a very hilly road of 10 km length; sensitivity of the TCO components of different vehicles and driving systems are shown for the optimum speed of the transportation scenario. A group of four bars from left to right represent BEHV HD, BEHV ADS-V, CHV HD and CHV ADS-DV, respectively. See Table 6 for the other vehicle sizes and road types.

**Fig. 96:**
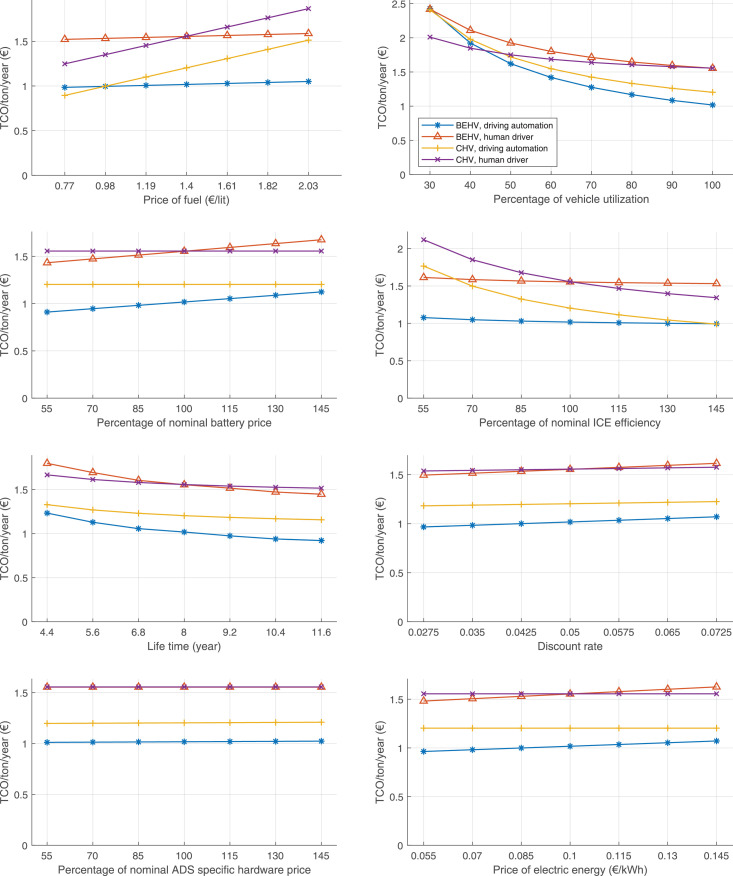
A-double on a very hilly road of 10 km length; sensitivity of TCO to different parameters are shown for the optimum speed of the transportation scenario. See Table 6 for the other vehicle sizes and road types.

**Fig. 97:**
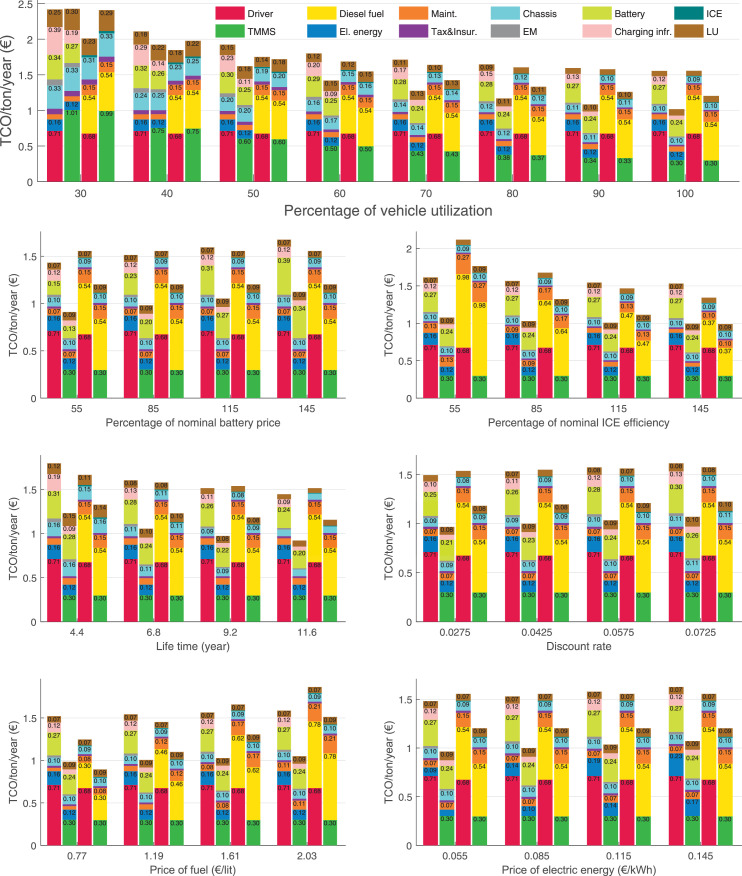
A-double on a very hilly road of 10 km length; sensitivity of the TCO components of different vehicles and driving systems are shown for the optimum speed of the transportation scenario. A group of four bars from left to right represent BEHV HD, BEHV ADS-V, CHV HD and CHV ADS-DV, respectively. See Table 6 for the other vehicle sizes and road types.

**Fig. 98:**
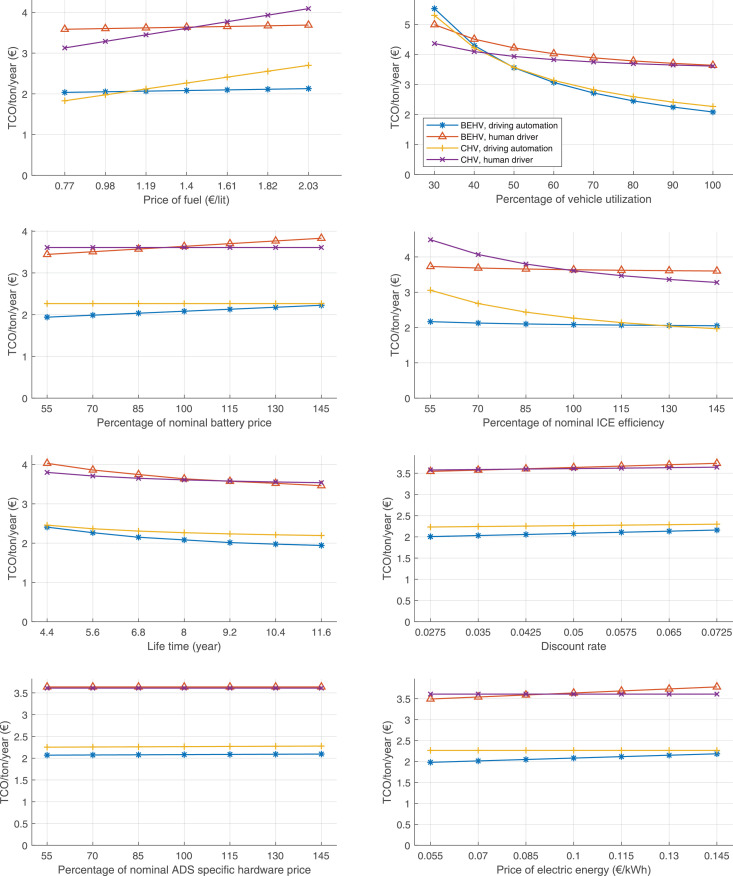
Rigid truck on a flat road of 20 km length; sensitivity of TCO to different parameters are shown for the optimum speed of the transportation scenario. See Table 6 for the other vehicle sizes and road types.

**Fig. 99:**
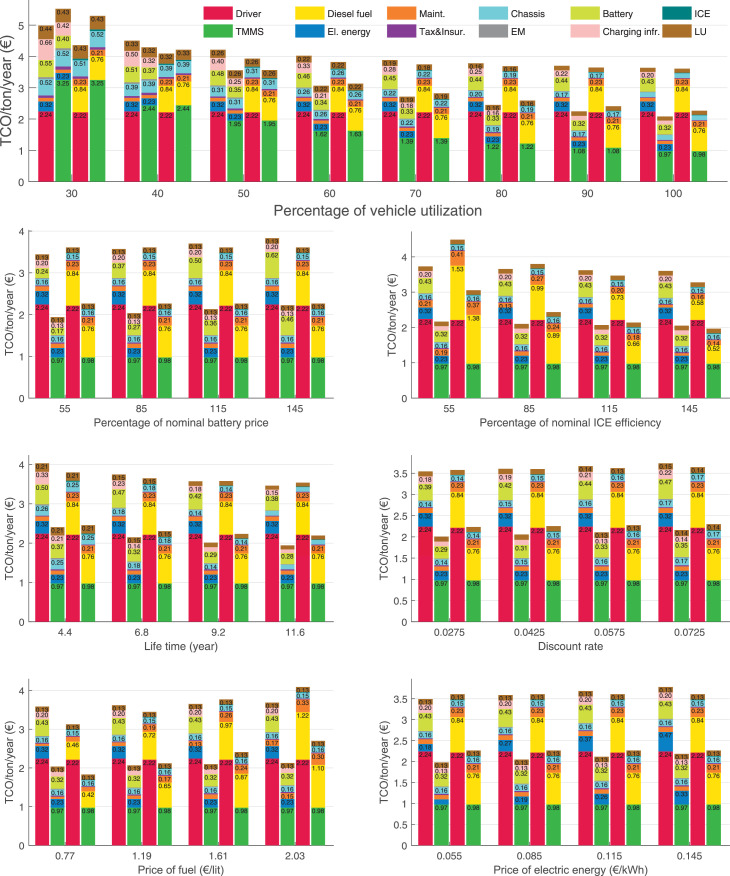
Rigid truck on a flat road of 20 km length; sensitivity of the TCO components of different vehicles and driving systems are shown for the optimum speed of the transportation scenario. A group of four bars from left to right represent BEHV HD, BEHV ADS-V, CHV HD and CHV ADS-DV, respectively. See Table 6 for the other vehicle sizes and road types.

**Fig. 100:**
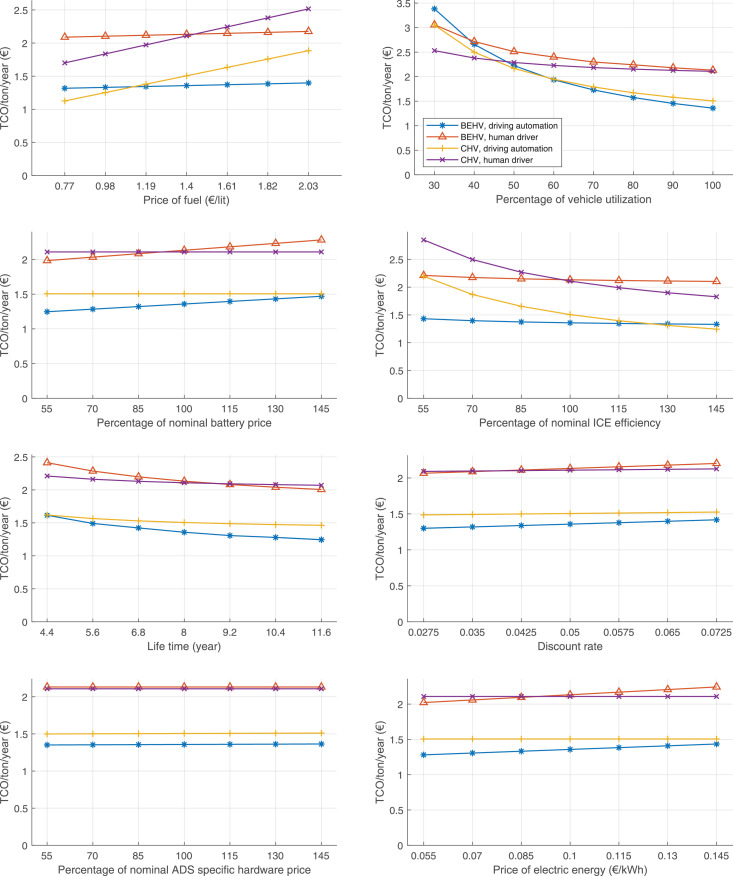
Tractor-semitrailer on a flat road of 20 km length; sensitivity of TCO to different parameters are shown for the optimum speed of the transportation scenario. See Table 6 for the other vehicle sizes and road types.

**Fig. 101:**
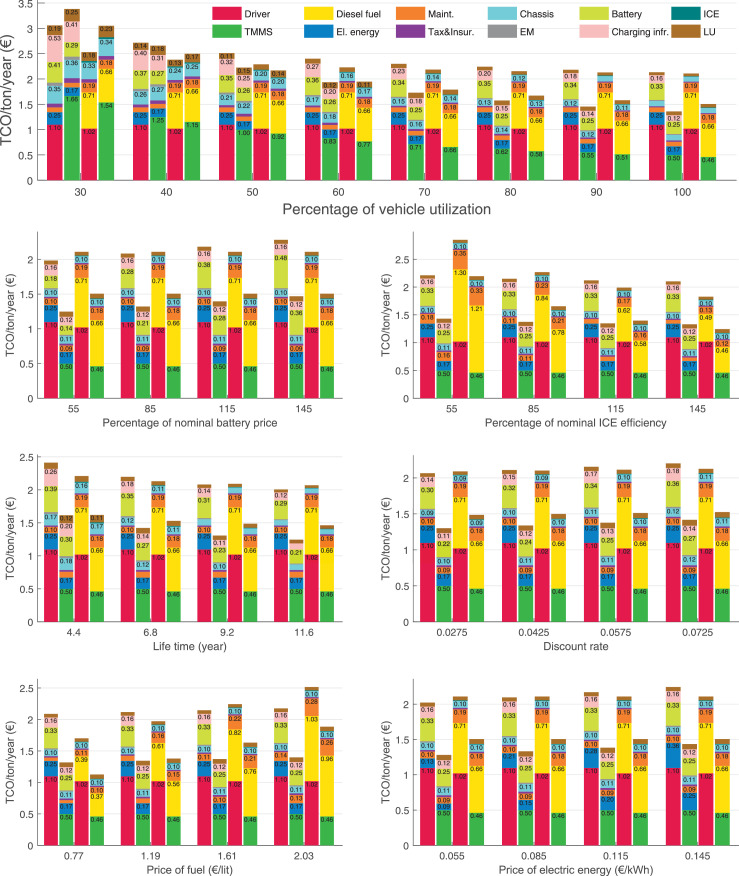
Tractor-semitrailer on a flat road of 20 km length; sensitivity of the TCO components of different vehicles and driving systems are shown for the optimum speed of the transportation scenario. A group of four bars from left to right represent BEHV HD, BEHV ADS-V, CHV HD and CHV ADS-DV, respectively. See Table 6 for the other vehicle sizes and road types.

**Fig. 102:**
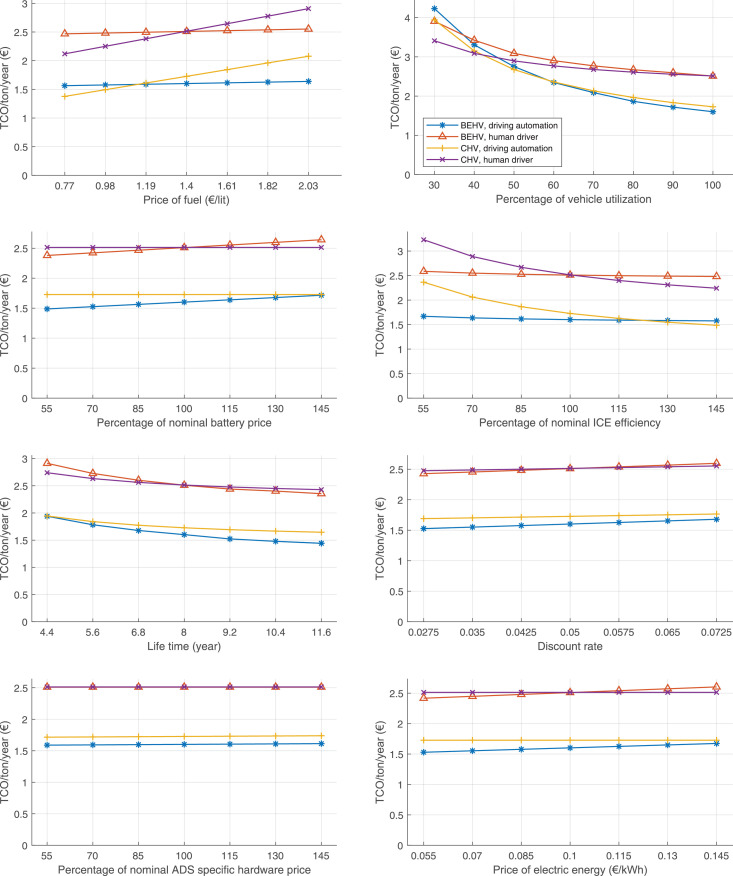
Nordic combination on a flat road of 20 km length; sensitivity of TCO to different parameters are shown for the optimum speed of the transportation scenario. See Table 6 for the other vehicle sizes and road types.

**Fig. 103:**
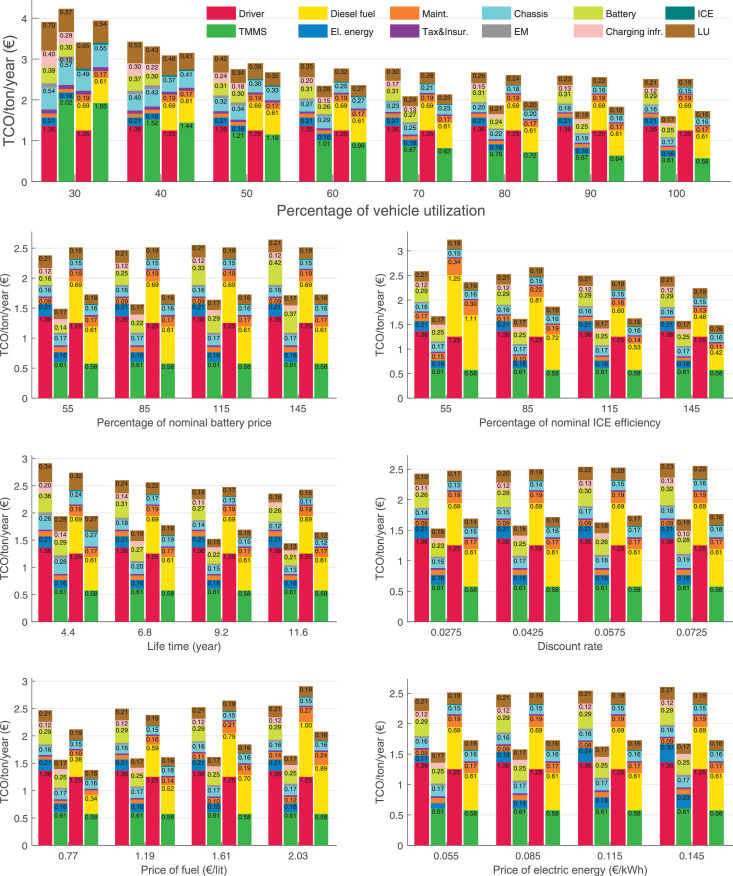
Nordic combination on a flat road of 20 km length; sensitivity of the TCO components of different vehicles and driving systems are shown for the optimum speed of the transportation scenario. A group of four bars from left to right represent BEHV HD, BEHV ADS-V, CHV HD and CHV ADS-DV, respectively. See Table 6 for the other vehicle sizes and road types.

**Fig. 104:**
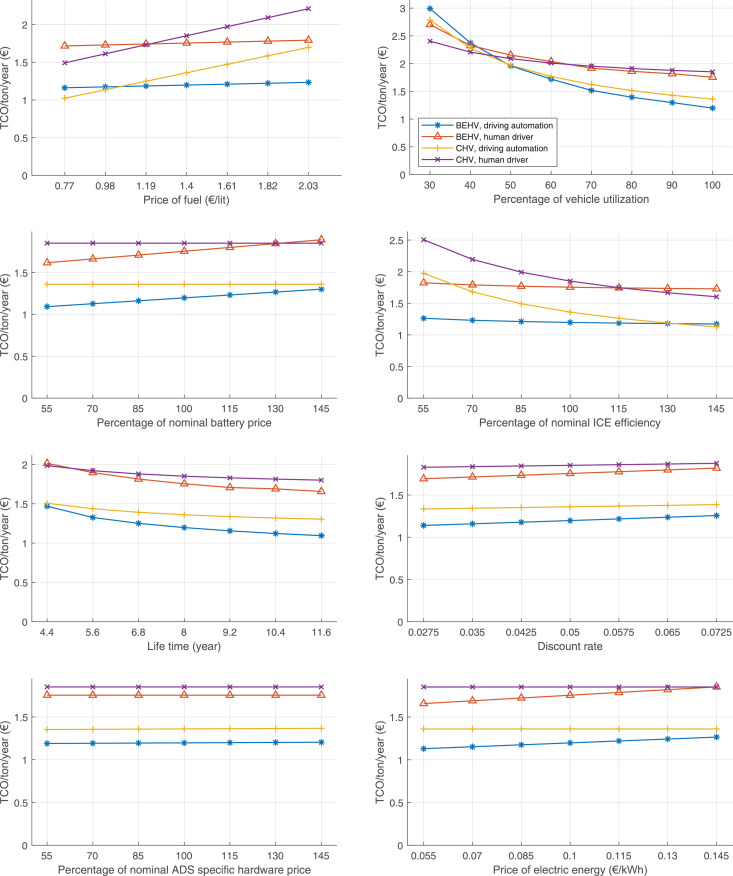
A-double on a flat road of 20 km length; sensitivity of TCO to different parameters are shown for the optimum speed of the transportation scenario. See Table 6 for the other vehicle sizes and road types.

**Fig. 105:**
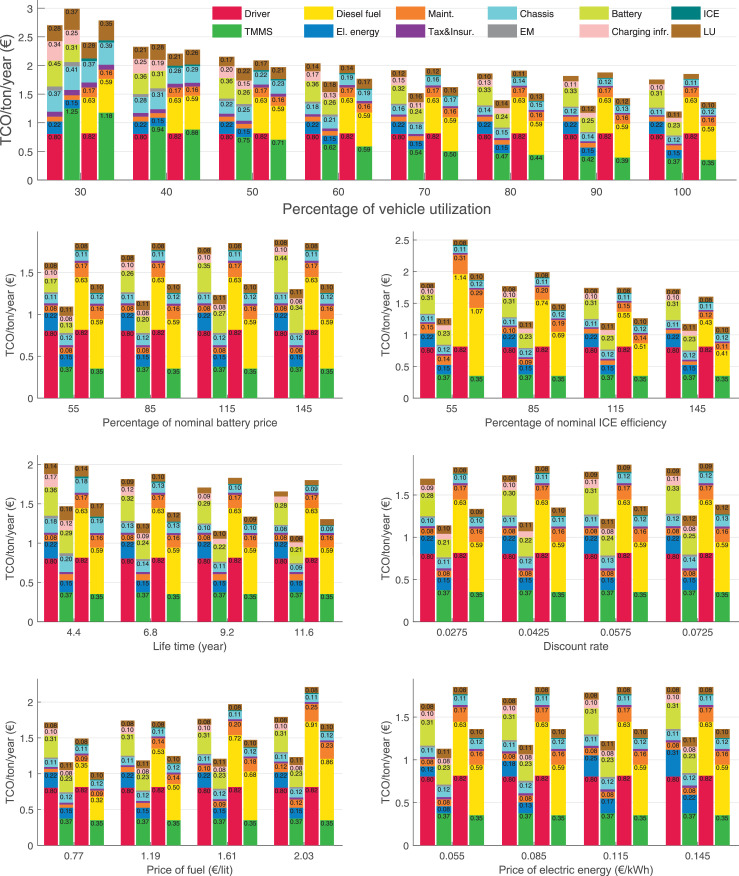
A-double on a flat road of 20 km length; sensitivity of the TCO components of different vehicles and driving systems are shown for the optimum speed of the transportation scenario. A group of four bars from left to right represent BEHV HD, BEHV ADS-V, CHV HD and CHV ADS-DV, respectively. See Table 6 for the other vehicle sizes and road types.

**Fig. 106:**
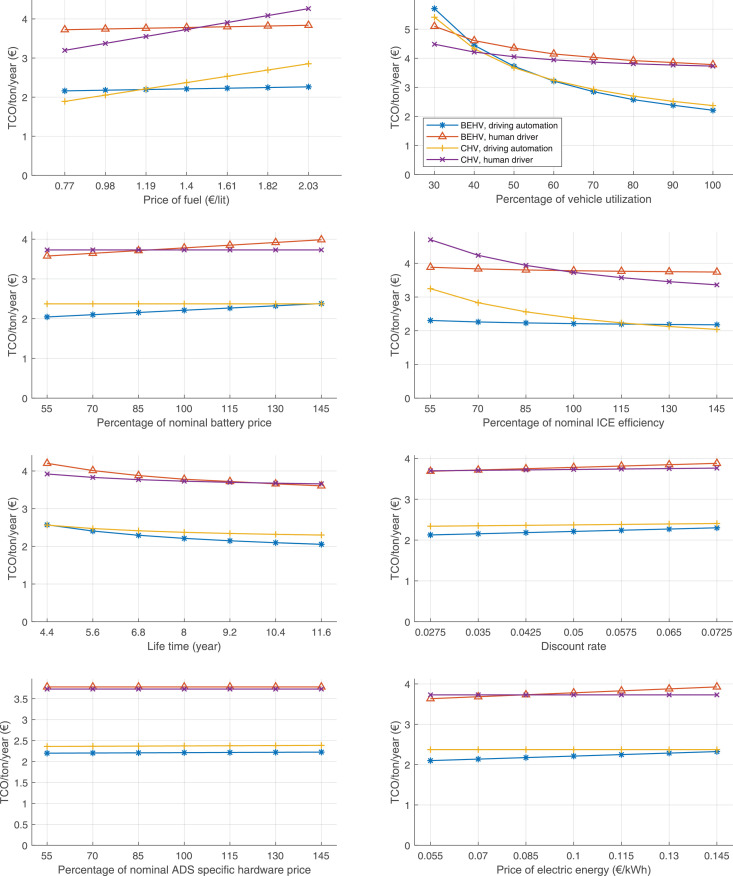
Rigid truck on a predominantly flat road of 20 km length; sensitivity of TCO to different parameters are shown for the optimum speed of the transportation scenario. See Table 6 for the other vehicle sizes and road types.

**Fig. 107:**
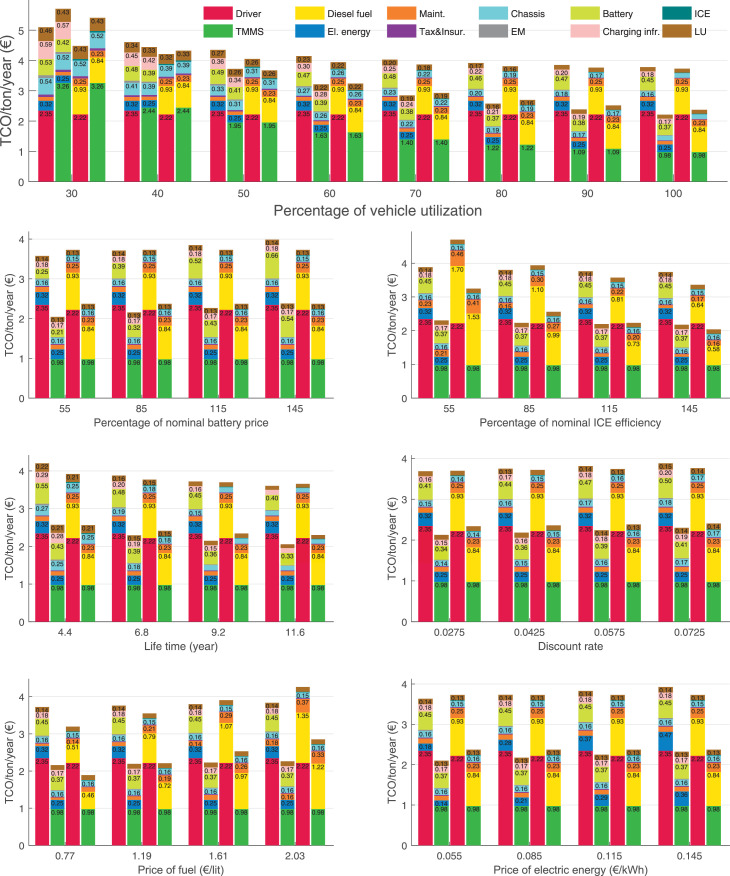
Rigid truck on a predominantly flat road of 20 km length; sensitivity of the TCO components of different vehicles and driving systems are shown for the optimum speed of the transportation scenario. A group of four bars from left to right represent BEHV HD, BEHV ADS-V, CHV HD and CHV ADS-DV, respectively. See Table 6 for the other vehicle sizes and road types.

**Fig. 108:**
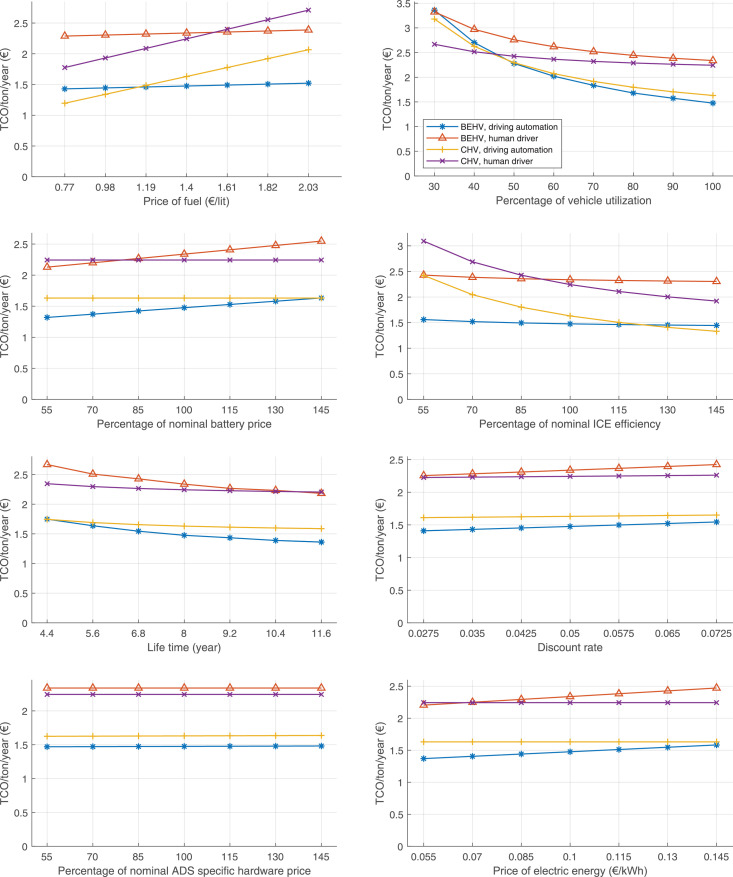
Tractor-semitrailer on a predominantly flat road of 20 km length; sensitivity of TCO to different parameters are shown for the optimum speed of the transportation scenario. See Table 6 for the other vehicle sizes and road types.

**Fig. 109:**
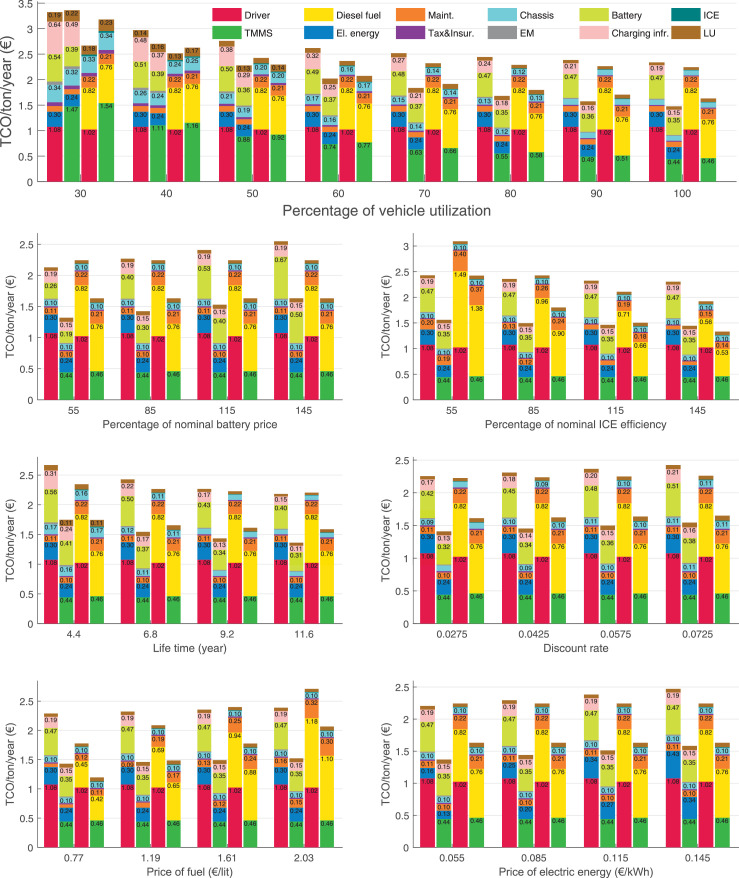
Tractor-semitrailer on a predominantly flat road of 20 km length; sensitivity of the TCO components of different vehicles and driving systems are shown for the optimum speed of the transportation scenario. A group of four bars from left to right represent BEHV HD, BEHV ADS-V, CHV HD and CHV ADS-DV, respectively. See Table 6 for the other vehicle sizes and road types.

**Fig. 110:**
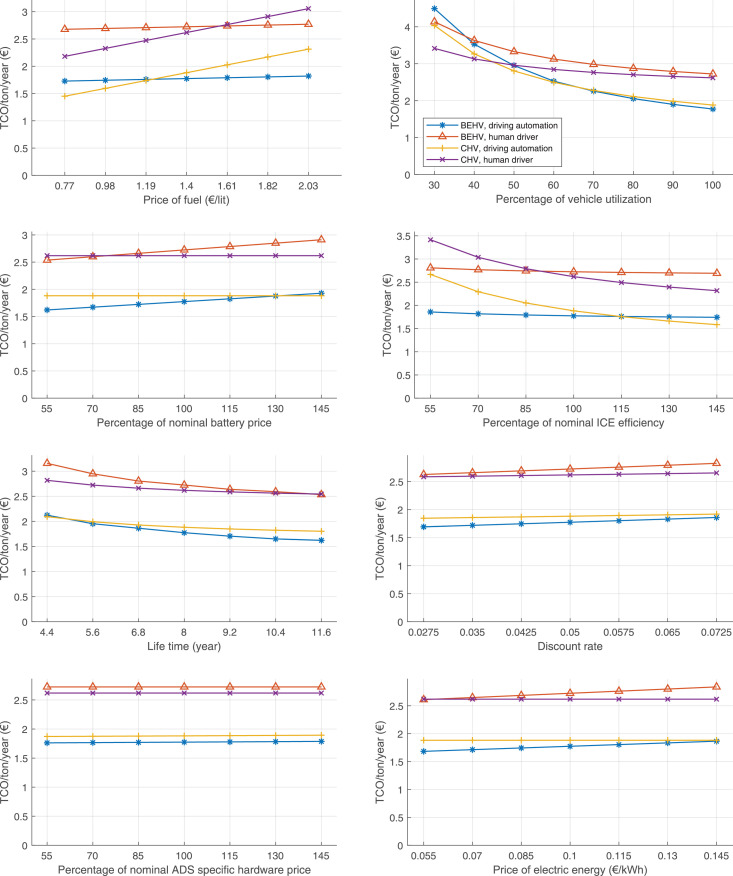
Nordic combination on a predominantly flat road of 20 km length; sensitivity of TCO to different parameters are shown for the optimum speed of the transportation scenario. See Table 6 for the other vehicle sizes and road types.

**Fig. 111:**
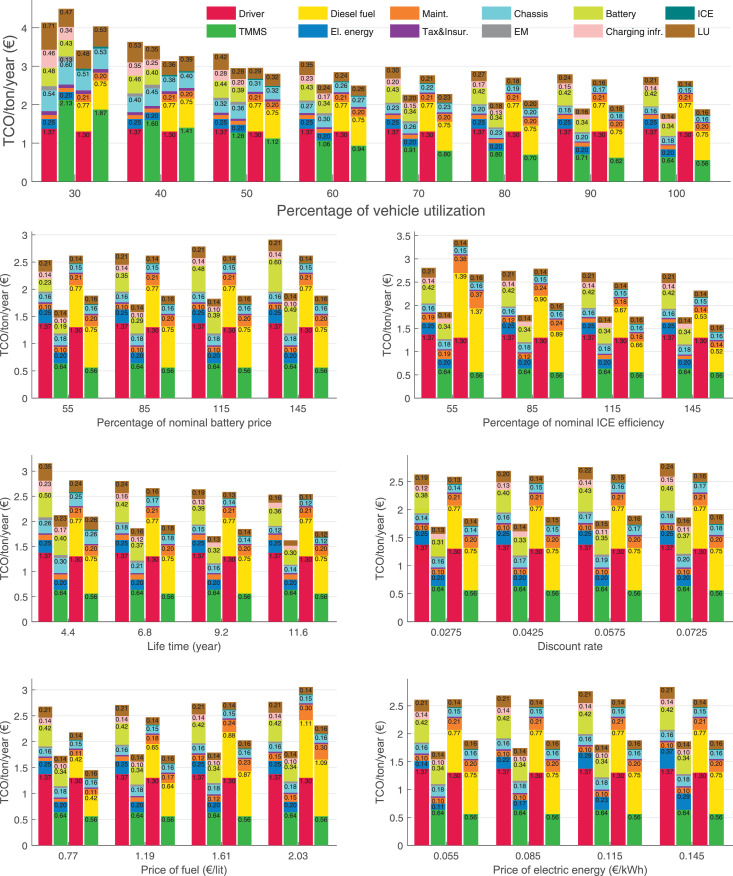
Nordic combination on a predominantly flat road of 20 km length; sensitivity of the TCO components of different vehicles and driving systems are shown for the optimum speed of the transportation scenario. A group of four bars from left to right represent BEHV HD, BEHV ADS-V, CHV HD and CHV ADS-DV, respectively. See Table 6 for the other vehicle sizes and road types.

**Fig. 112:**
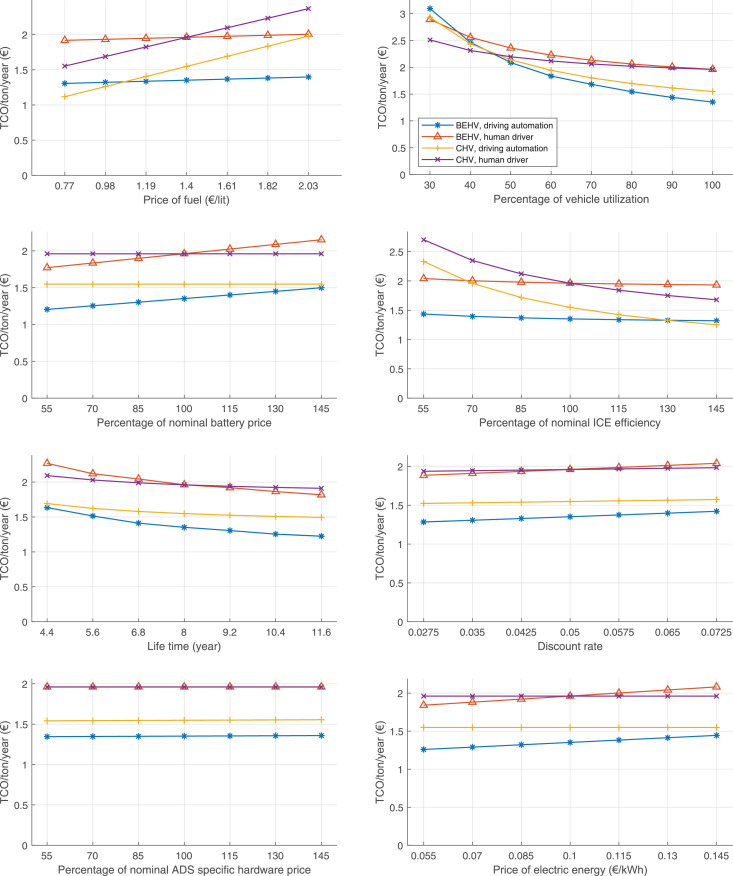
A-double on a predominantly flat road of 20 km length; sensitivity of TCO to different parameters are shown for the optimum speed of the transportation scenario. See Table 6 for the other vehicle sizes and road types.

**Fig. 113:**
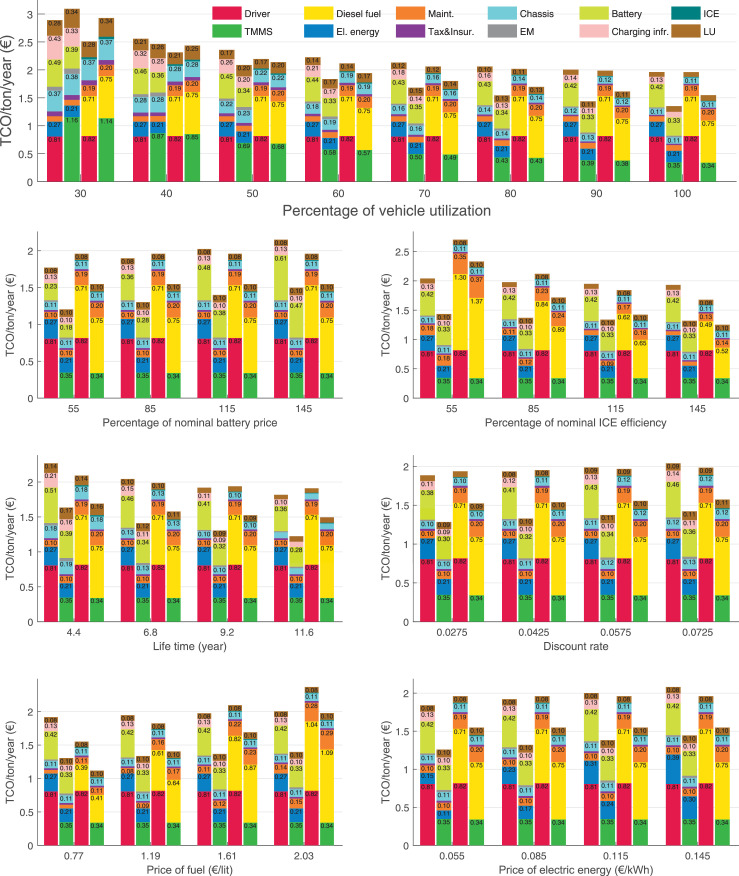
A-double on a predominantly flat road of 20 km length; sensitivity of the TCO components of different vehicles and driving systems are shown for the optimum speed of the transportation scenario. A group of four bars from left to right represent BEHV HD, BEHV ADS-V, CHV HD and CHV ADS-DV, respectively. See Table 6 for the other vehicle sizes and road types.

**Fig. 114:**
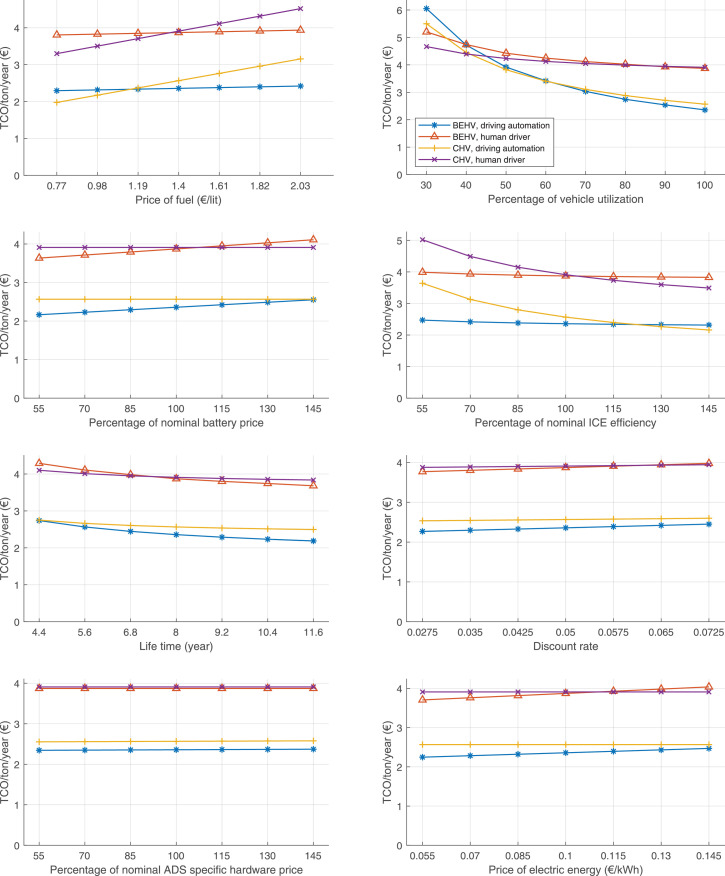
Rigid truck on a hilly road of 20 km length; sensitivity of TCO to different parameters are shown for the optimum speed of the transportation scenario. See Table 6 for the other vehicle sizes and road types.

**Fig. 115:**
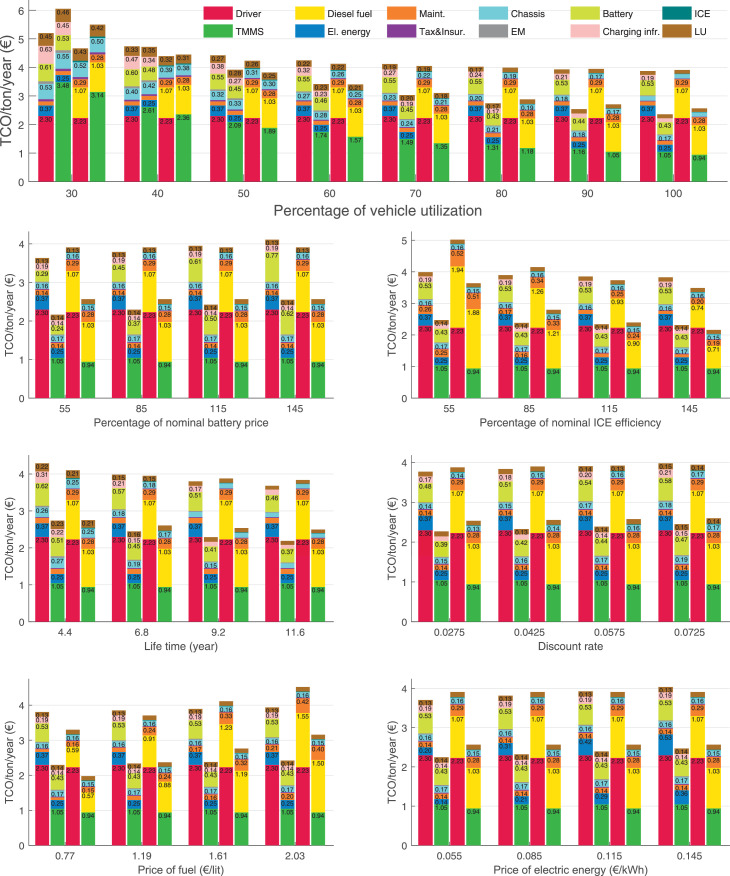
Rigid truck on a hilly road of 20 km length; sensitivity of the TCO components of different vehicles and driving systems are shown for the optimum speed of the transportation scenario. A group of four bars from left to right represent BEHV HD, BEHV ADS-V, CHV HD and CHV ADS-DV, respectively. See Table 6 for the other vehicle sizes and road types.

**Fig. 116:**
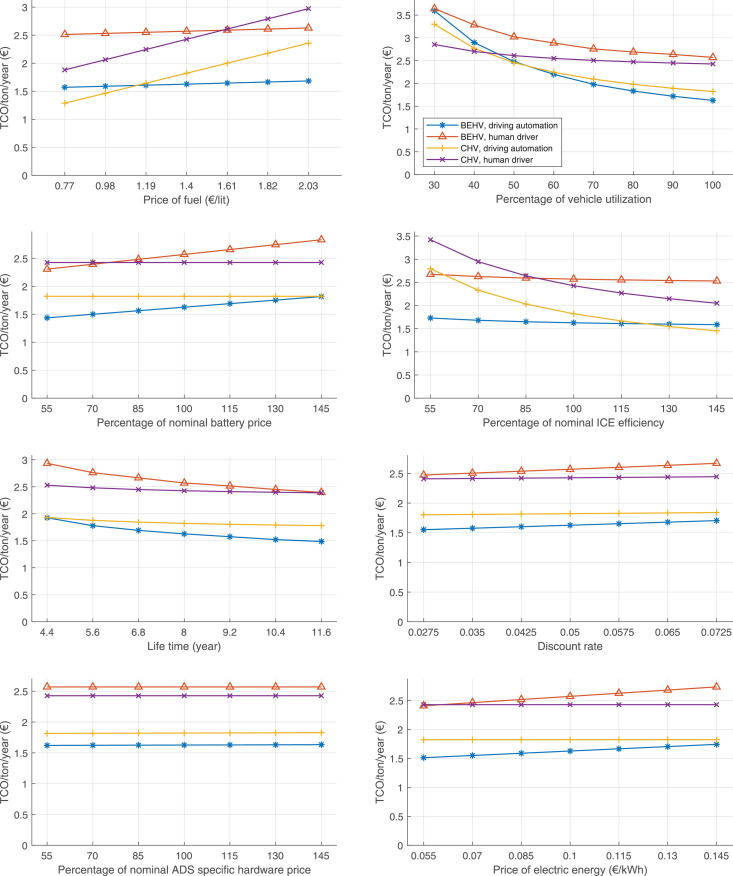
Tractor-semitrailer on a hilly road of 20 km length; sensitivity of TCO to different parameters are shown for the optimum speed of the transportation scenario. See Table 6 for the other vehicle sizes and road types.

**Fig. 117:**
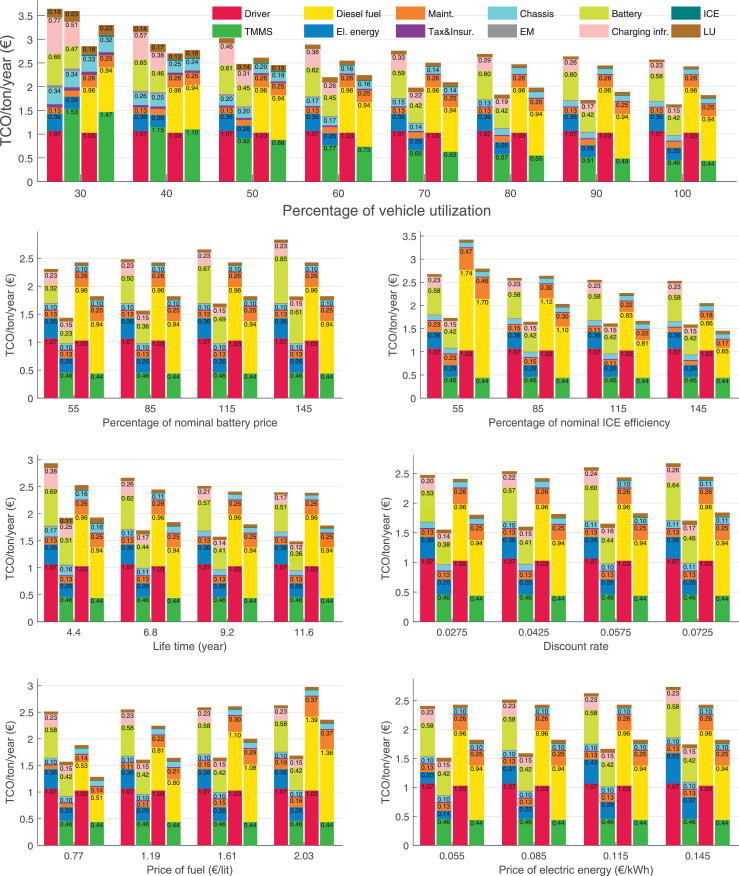
Tractor-semitrailer on a hilly road of 20 km length; sensitivity of the TCO components of different vehicles and driving systems are shown for the optimum speed of the transportation scenario. A group of four bars from left to right represent BEHV HD, BEHV ADS-V, CHV HD and CHV ADS-DV, respectively. See Table 6 for the other vehicle sizes and road types.

**Fig. 118:**
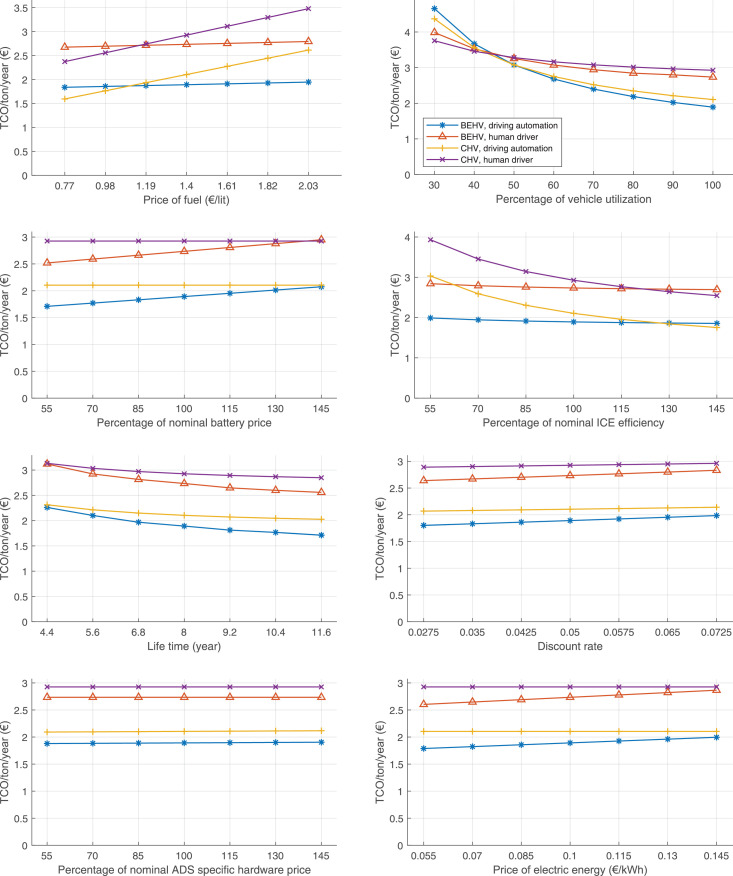
Nordic combination on a hilly road of 20 km length; sensitivity of TCO to different parameters are shown for the optimum speed of the transportation scenario. See Table 6 for the other vehicle sizes and road types.

**Fig. 119:**
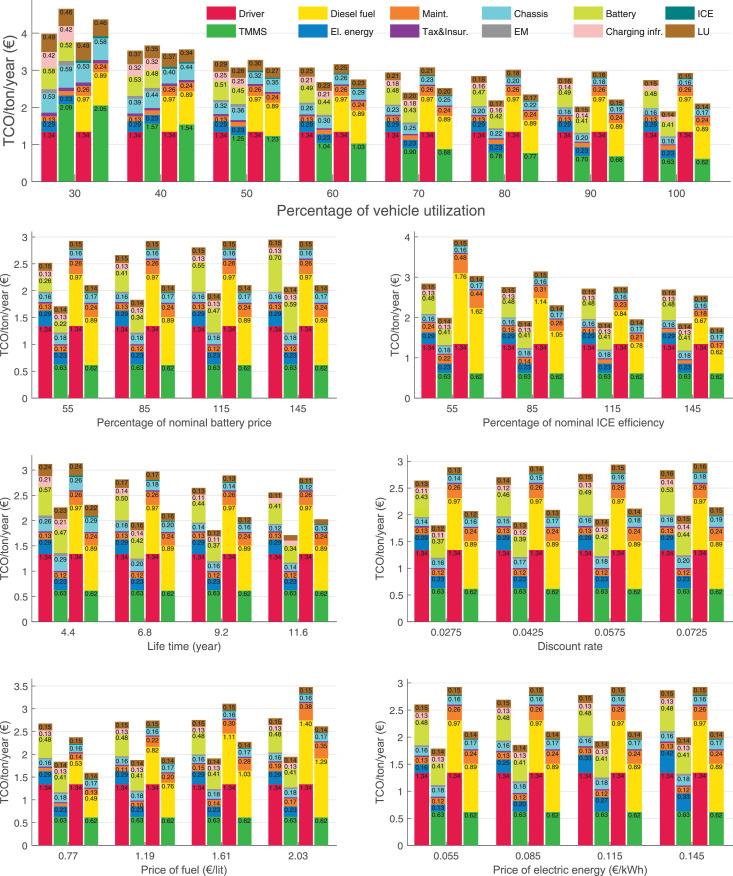
Nordic combination on a hilly road of 20 km length; sensitivity of the TCO components of different vehicles and driving systems are shown for the optimum speed of the transportation scenario. A group of four bars from left to right represent BEHV HD, BEHV ADS-V, CHV HD and CHV ADS-DV, respectively. See Table 6 for the other vehicle sizes and road types.

**Fig. 120:**
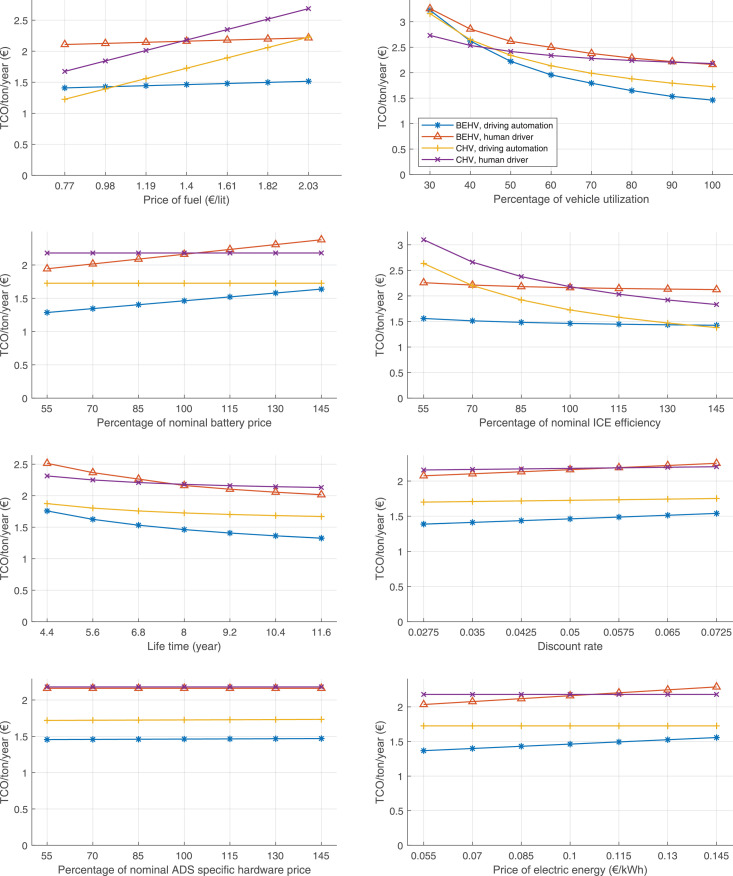
A-double on a hilly road of 20 km length; sensitivity of TCO to different parameters are shown for the optimum speed of the transportation scenario. See Table 6 for the other vehicle sizes and road types.

**Fig. 121:**
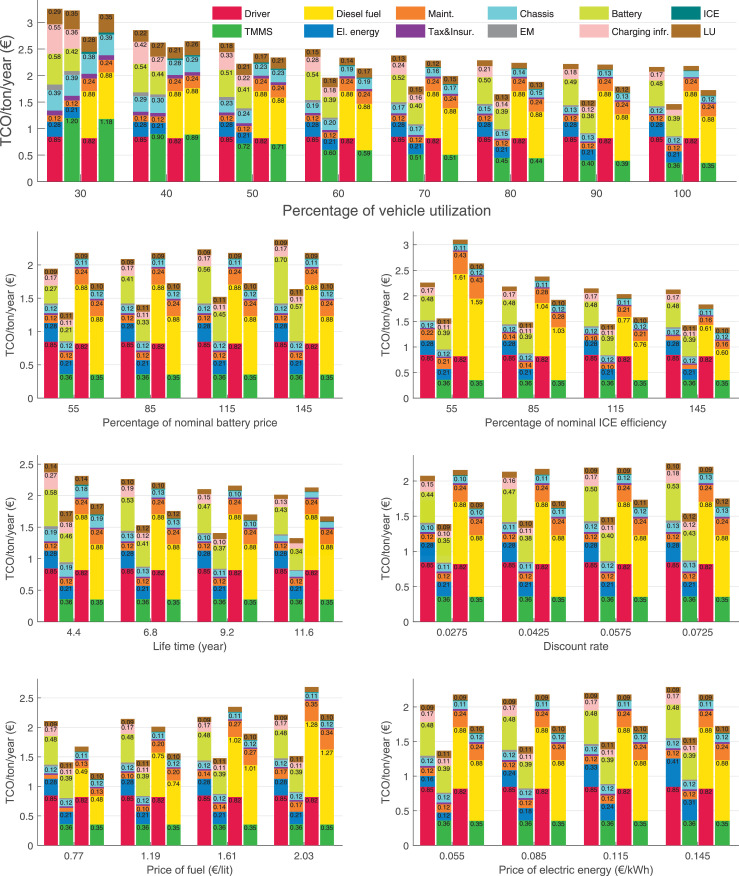
A-double on a hilly road of 20 km length; sensitivity of the TCO components of different vehicles and driving systems are shown for the optimum speed of the transportation scenario. A group of four bars from left to right represent BEHV HD, BEHV ADS-V, CHV HD and CHV ADS-DV, respectively. See Table 6 for the other vehicle sizes and road types.

**Fig. 122:**
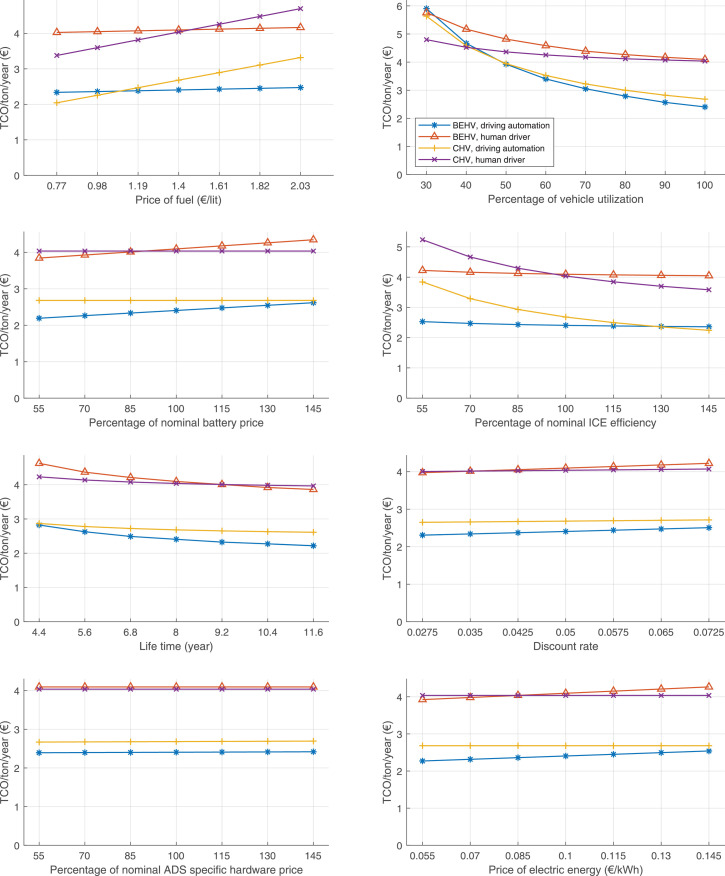
Rigid truck on a very hilly road of 20 km length; sensitivity of TCO to different parameters are shown for the optimum speed of the transportation scenario. See Table 6 for the other vehicle sizes and road types.

**Fig. 123:**
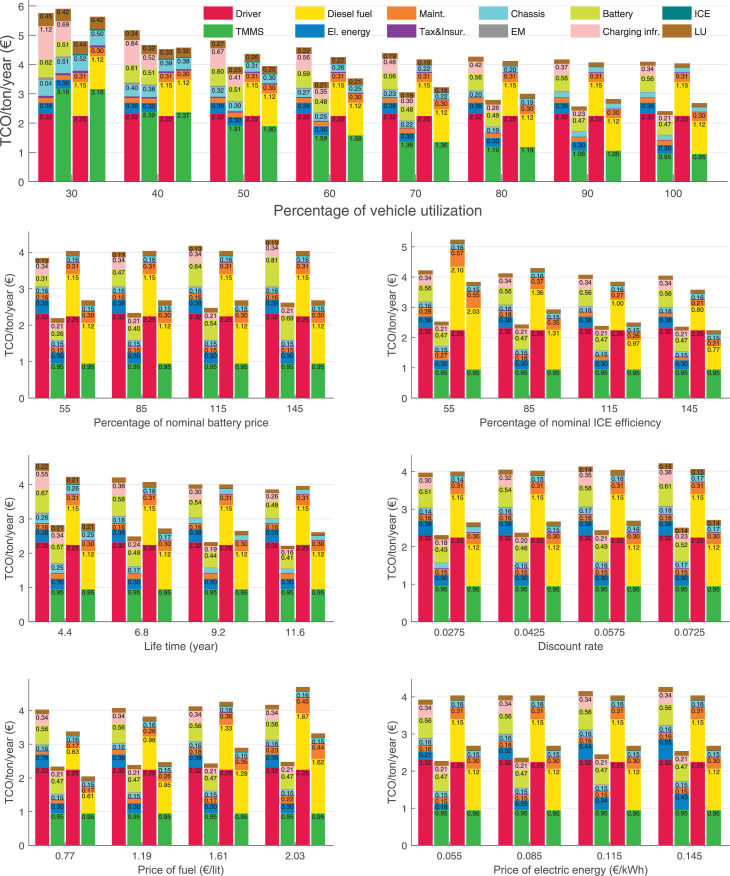
Rigid truck on a very hilly road of 20 km length; sensitivity of the TCO components of different vehicles and driving systems are shown for the optimum speed of the transportation scenario. A group of four bars from left to right represent BEHV HD, BEHV ADS-V, CHV HD and CHV ADS-DV, respectively. See Table 6 for the other vehicle sizes and road types.

**Fig. 124:**
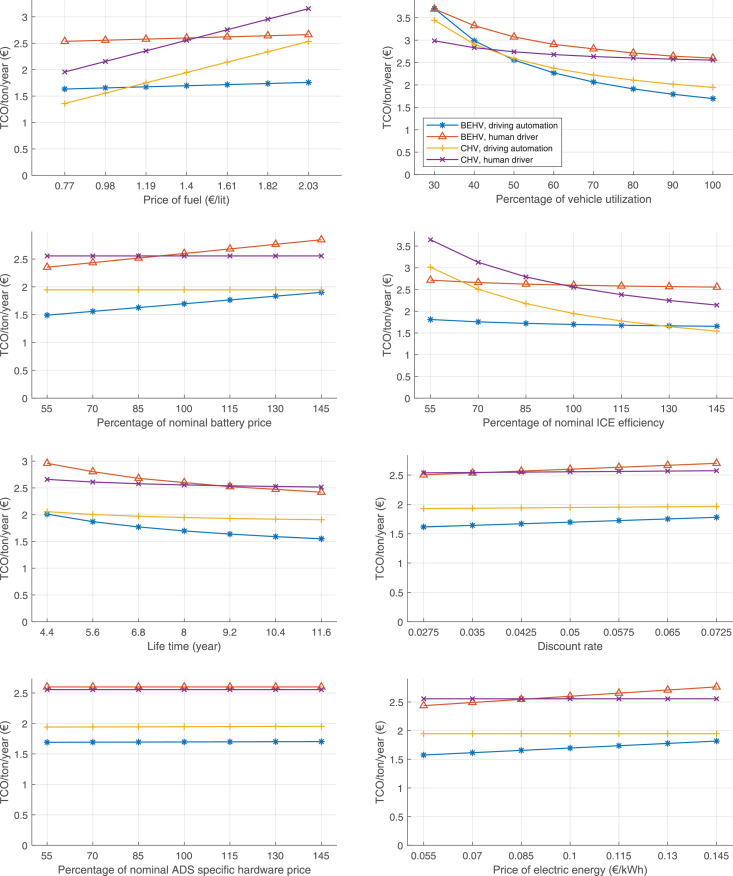
Tractor-semitrailer on a very hilly road of 20 km length; sensitivity of TCO to different parameters are shown for the optimum speed of the transportation scenario. See Table 6 for the other vehicle sizes and road types.

**Fig. 125:**
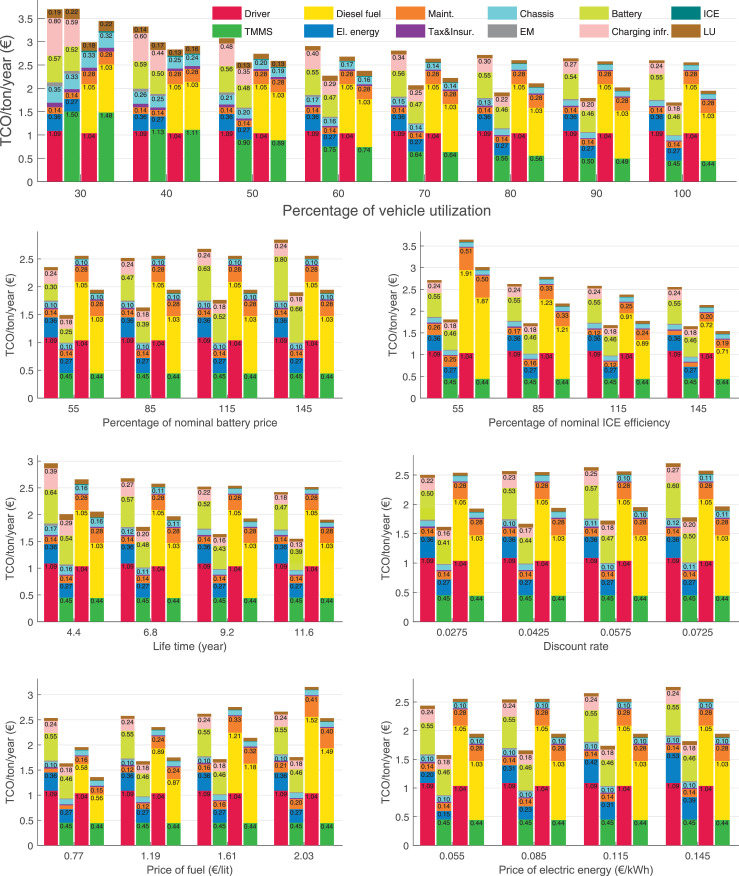
Tractor-semitrailer on a very hilly road of 20 km length; sensitivity of the TCO components of different vehicles and driving systems are shown for the optimum speed of the transportation scenario. A group of four bars from left to right represent BEHV HD, BEHV ADS-V, CHV HD and CHV ADS-DV, respectively. See Table 6 for the other vehicle sizes and road types.

**Fig. 126:**
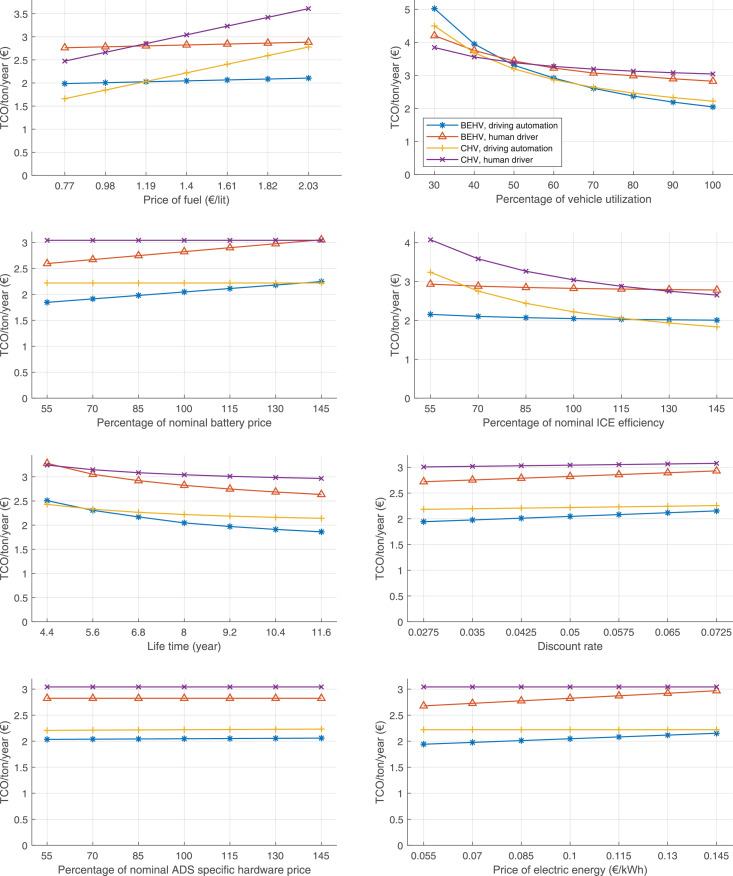
Nordic combination on a very hilly road of 20 km length; sensitivity of TCO to different parameters are shown for the optimum speed of the transportation scenario. See Table 6 for the other vehicle sizes and road types.

**Fig. 127:**
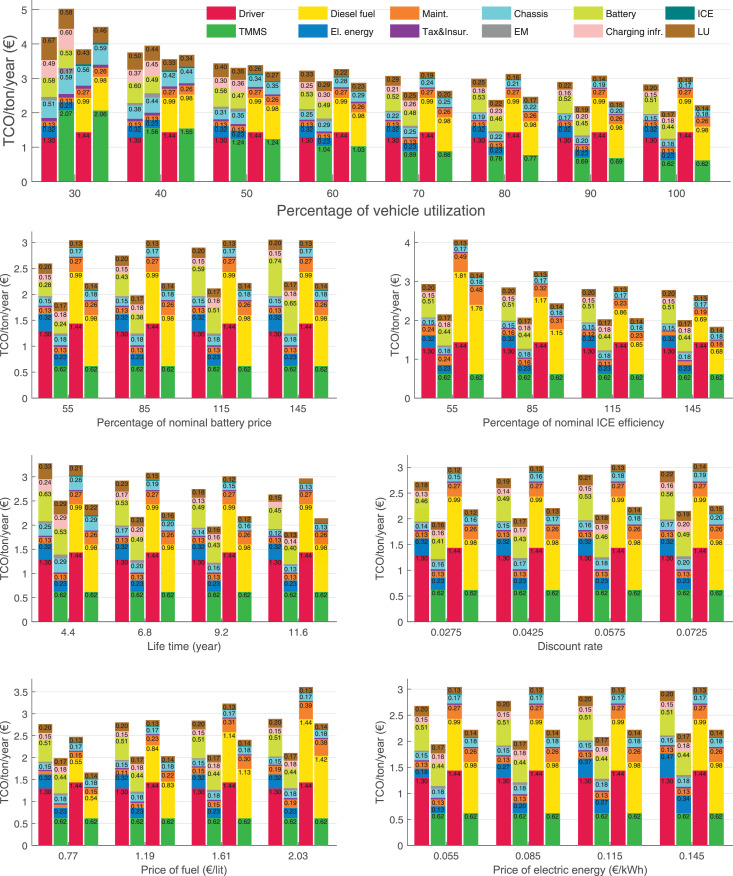
Nordic combination on a very hilly road of 20 km length; sensitivity of the TCO components of different vehicles and driving systems are shown for the optimum speed of the transportation scenario. A group of four bars from left to right represent BEHV HD, BEHV ADS-V, CHV HD and CHV ADS-DV, respectively. See Table 6 for the other vehicle sizes and road types.

**Fig. 128:**
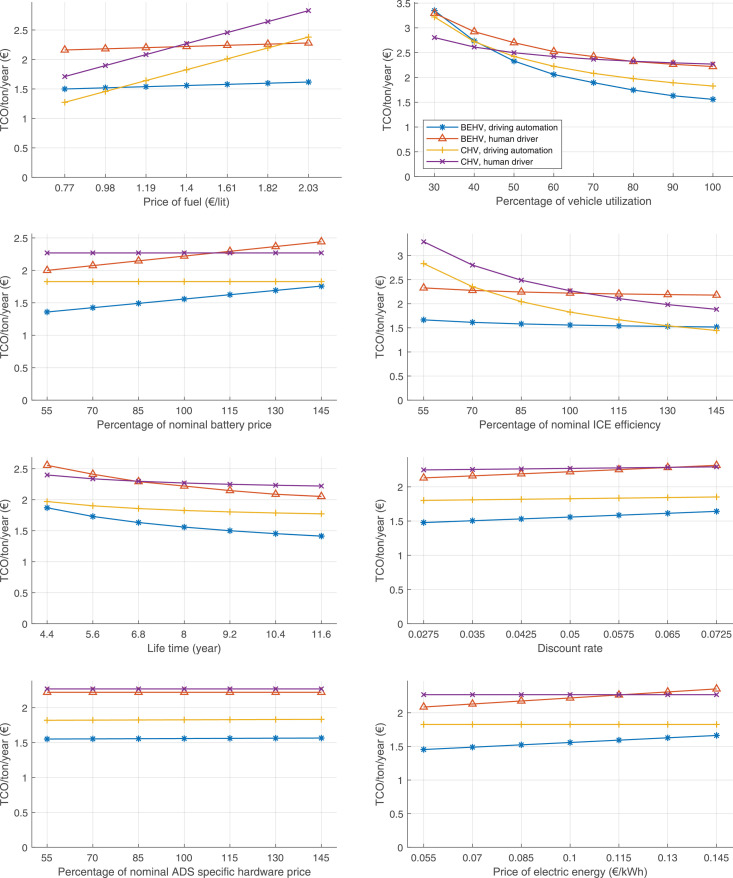
A-double on a very hilly road of 20 km length; sensitivity of TCO to different parameters are shown for the optimum speed of the transportation scenario. See Table 6 for the other vehicle sizes and road types.

**Fig. 129:**
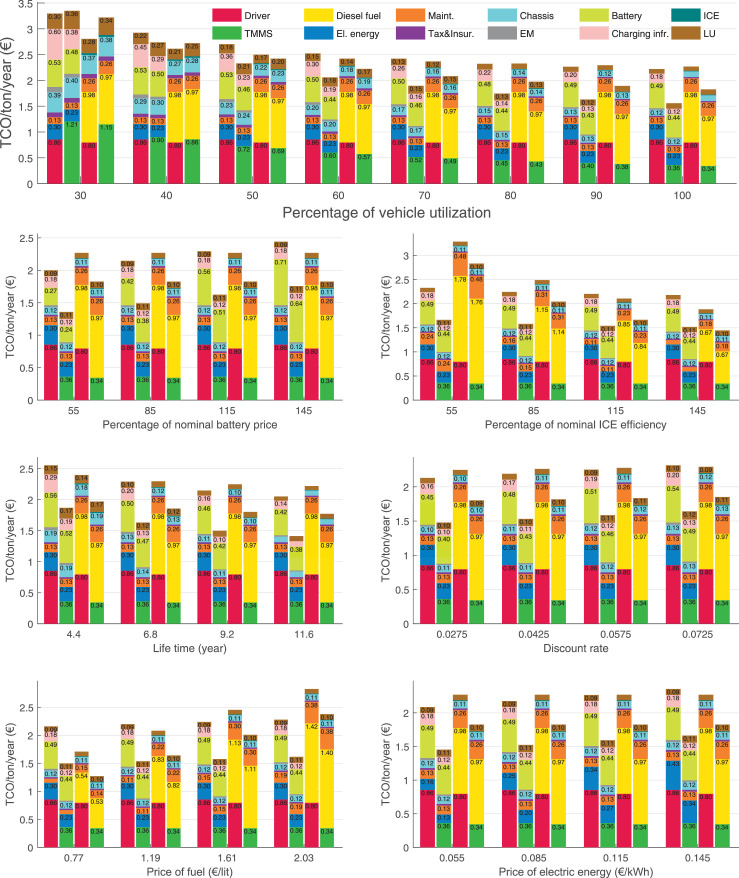
A-double on a very hilly road of 20 km length; sensitivity of the TCO components of different vehicles and driving systems are shown for the optimum speed of the transportation scenario. A group of four bars from left to right represent BEHV HD, BEHV ADS-V, CHV HD and CHV ADS-DV, respectively. See Table 6 for the other vehicle sizes and road types.

**Fig. 130:**
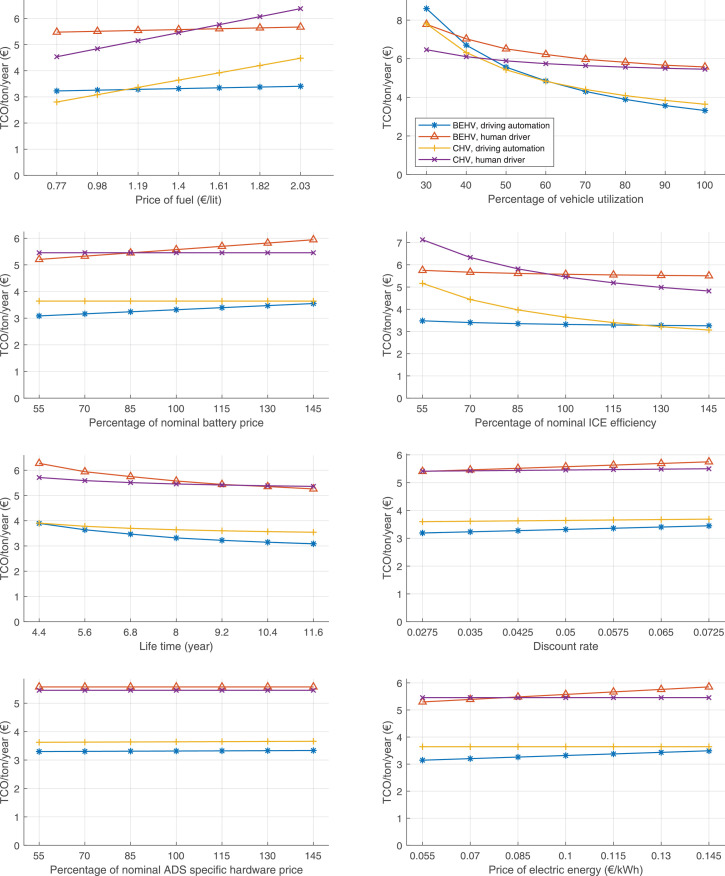
Rigid truck on a flat road of 40 km length; sensitivity of TCO to different parameters are shown for the optimum speed of the transportation scenario. See Table 6 for the other vehicle sizes and road types.

**Fig. 131:**
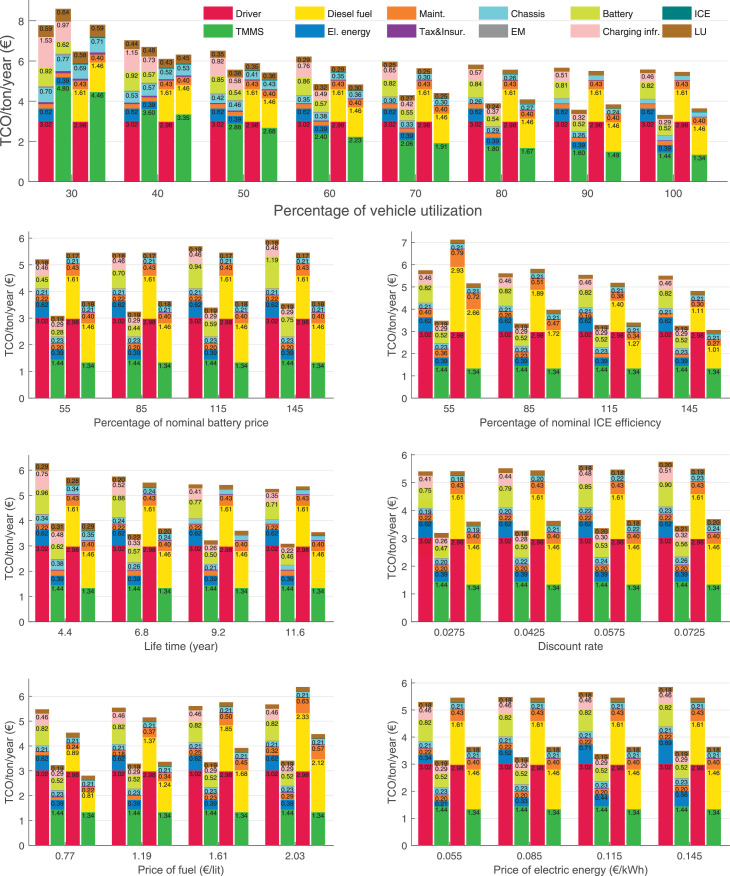
Rigid truck on a flat road of 40 km length; sensitivity of the TCO components of different vehicles and driving systems are shown for the optimum speed of the transportation scenario. A group of four bars from left to right represent BEHV HD, BEHV ADS-V, CHV HD and CHV ADS-DV, respectively. See Table 6 for the other vehicle sizes and road types.

**Fig. 132:**
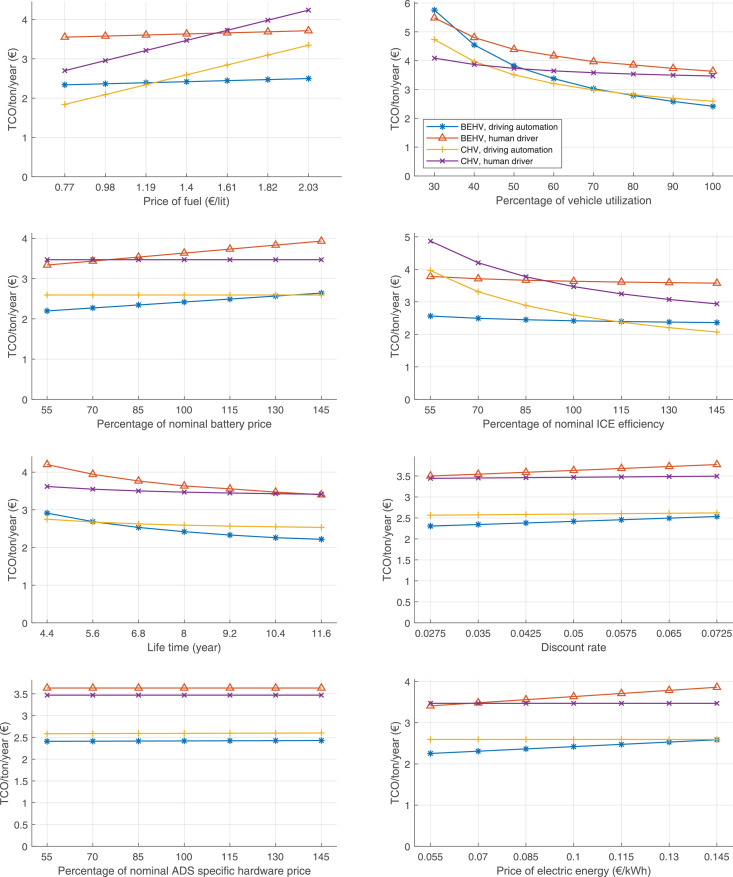
Tractor-semitrailer on a flat road of 40 km length; sensitivity of TCO to different parameters are shown for the optimum speed of the transportation scenario. See Table 6 for the other vehicle sizes and road types.

**Fig. 133:**
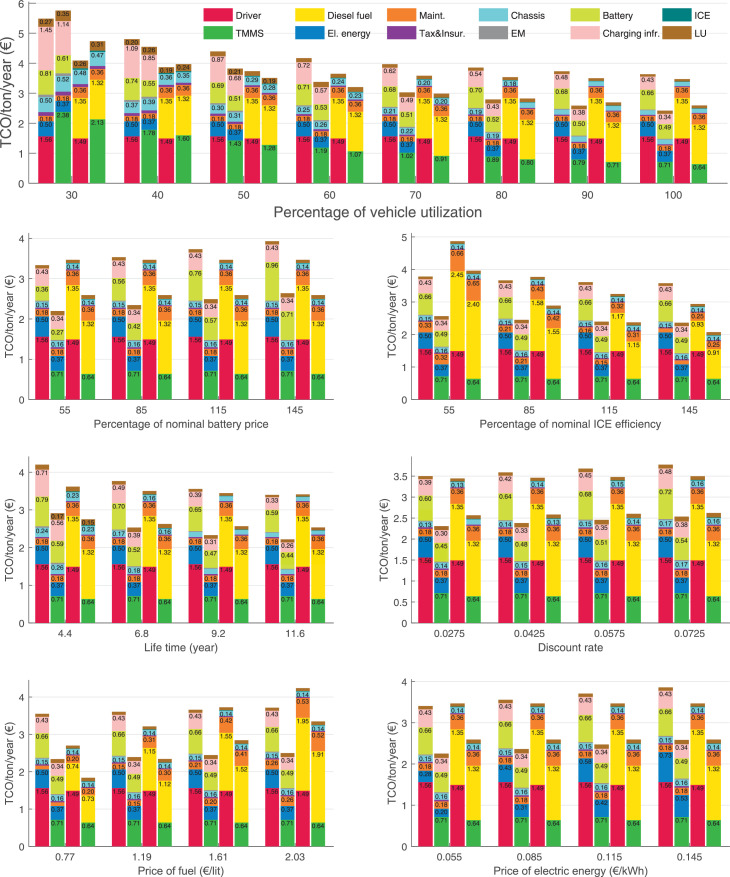
Tractor-semitrailer on a flat road of 40 km length; sensitivity of the TCO components of different vehicles and driving systems are shown for the optimum speed of the transportation scenario. A group of four bars from left to right represent BEHV HD, BEHV ADS-V, CHV HD and CHV ADS-DV, respectively. See Table 6 for the other vehicle sizes and road types.

**Fig. 134:**
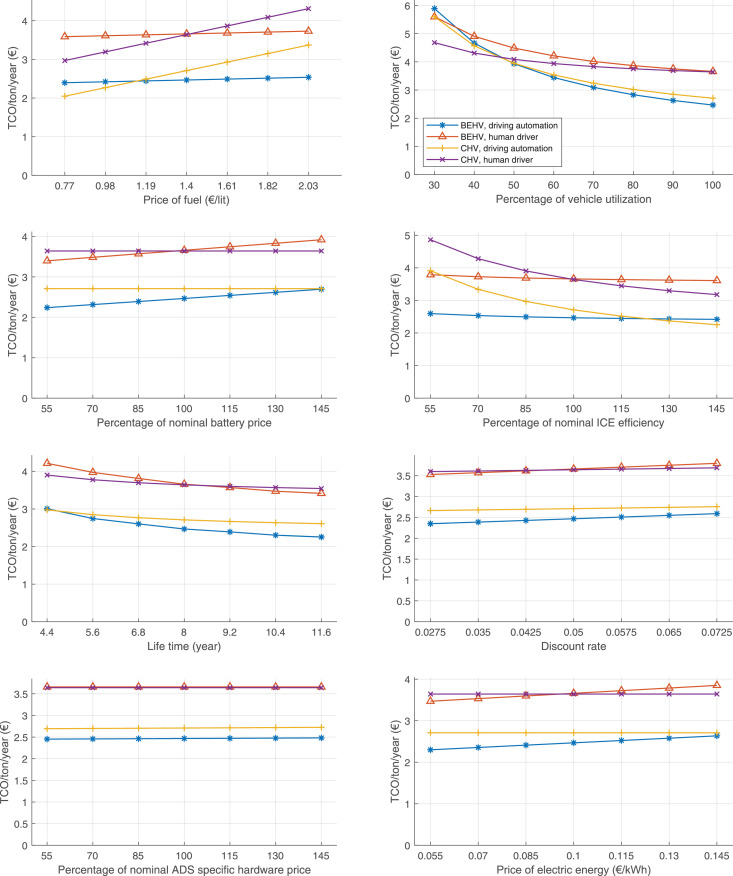
Nordic combination on a flat road of 40 km length; sensitivity of TCO to different parameters are shown for the optimum speed of the transportation scenario. See Table 6 for the other vehicle sizes and road types.

**Fig. 135:**
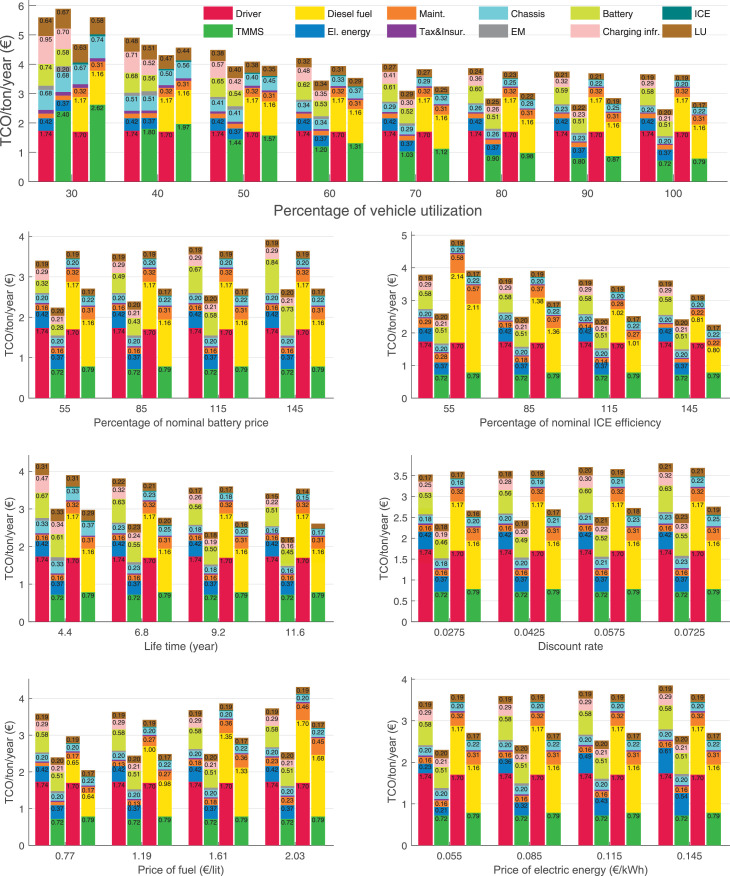
Nordic combination on a flat road of 40 km length; sensitivity of the TCO components of different vehicles and driving systems are shown for the optimum speed of the transportation scenario. A group of four bars from left to right represent BEHV HD, BEHV ADS-V, CHV HD and CHV ADS-DV, respectively. See Table 6 for the other vehicle sizes and road types.

**Fig. 136:**
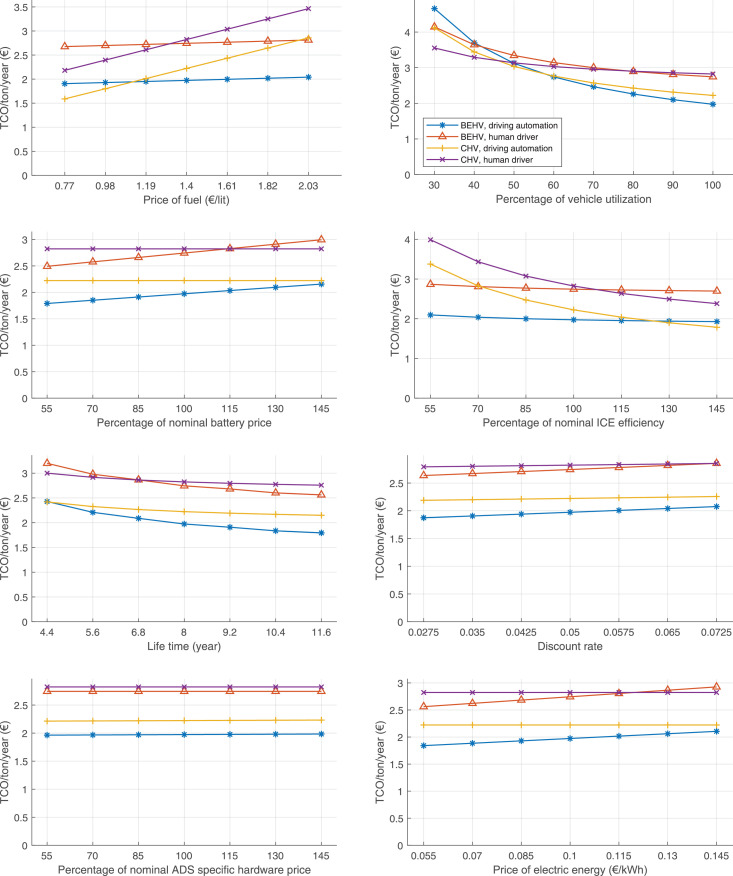
A-double on a flat road of 40 km length; sensitivity of TCO to different parameters are shown for the optimum speed of the transportation scenario. See Table 6 for the other vehicle sizes and road types.

**Fig. 137:**
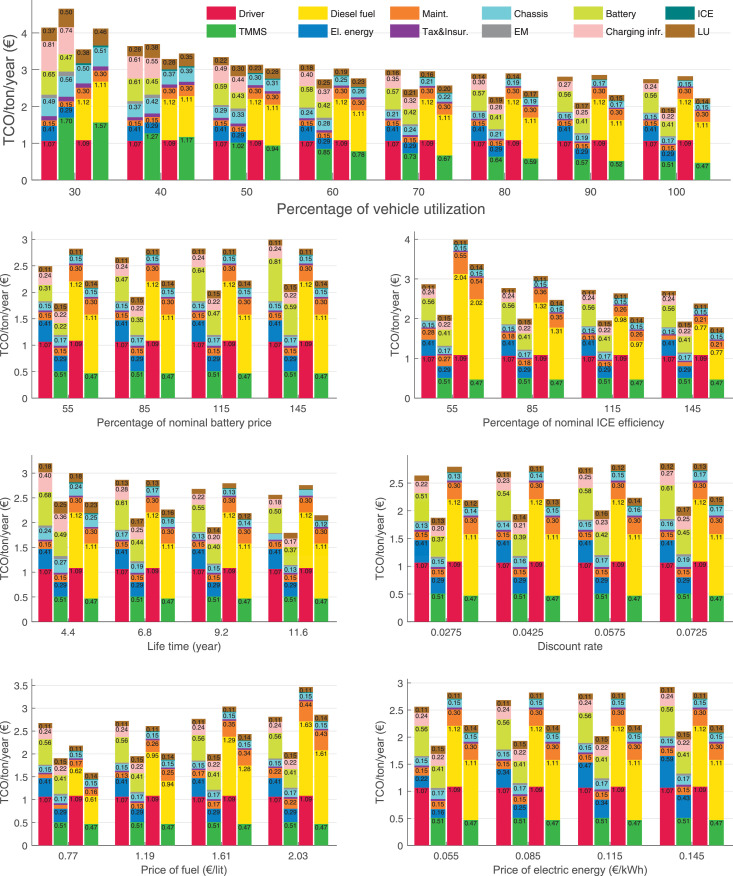
A-double on a flat road of 40 km length; sensitivity of the TCO components of different vehicles and driving systems are shown for the optimum speed of the transportation scenario. A group of four bars from left to right represent BEHV HD, BEHV ADS-V, CHV HD and CHV ADS-DV, respectively. See Table 6 for the other vehicle sizes and road types.

**Fig. 138:**
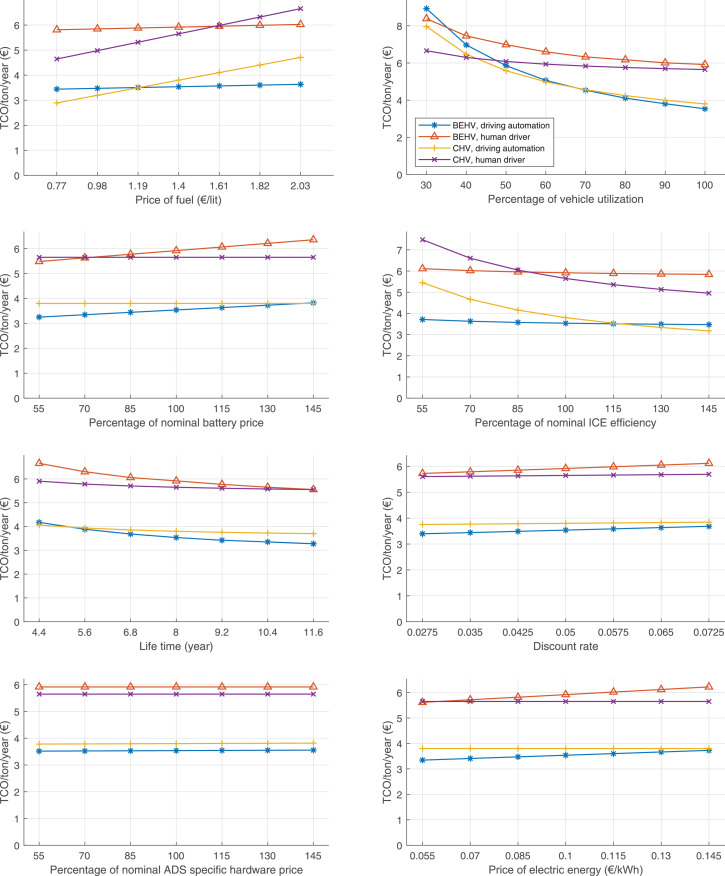
Rigid truck on a predominantly flat road of 40 km length; sensitivity of TCO to different parameters are shown for the optimum speed of the transportation scenario. See Table 6 for the other vehicle sizes and road types.

**Fig. 139:**
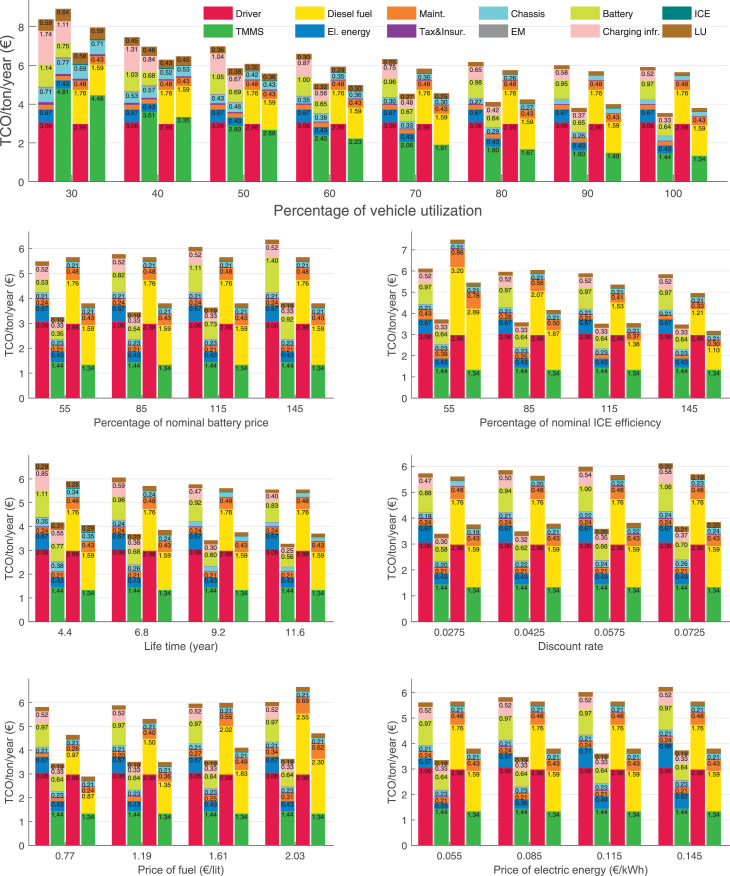
Rigid truck on a predominantly flat road of 40 km length; sensitivity of the TCO components of different vehicles and driving systems are shown for the optimum speed of the transportation scenario. A group of four bars from left to right represent BEHV HD, BEHV ADS-V, CHV HD and CHV ADS-DV, respectively. See Table 6 for the other vehicle sizes and road types.

**Fig. 140:**
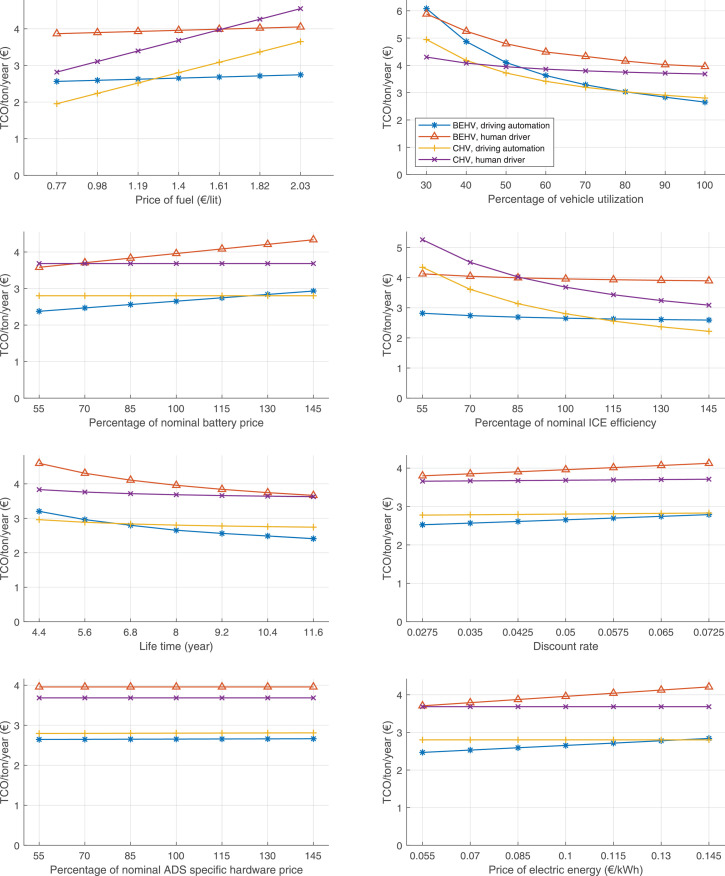
Tractor-semitrailer on a predominantly flat road of 40 km length; sensitivity of TCO to different parameters are shown for the optimum speed of the transportation scenario. See Table 6 for the other vehicle sizes and road types.

**Fig. 141:**
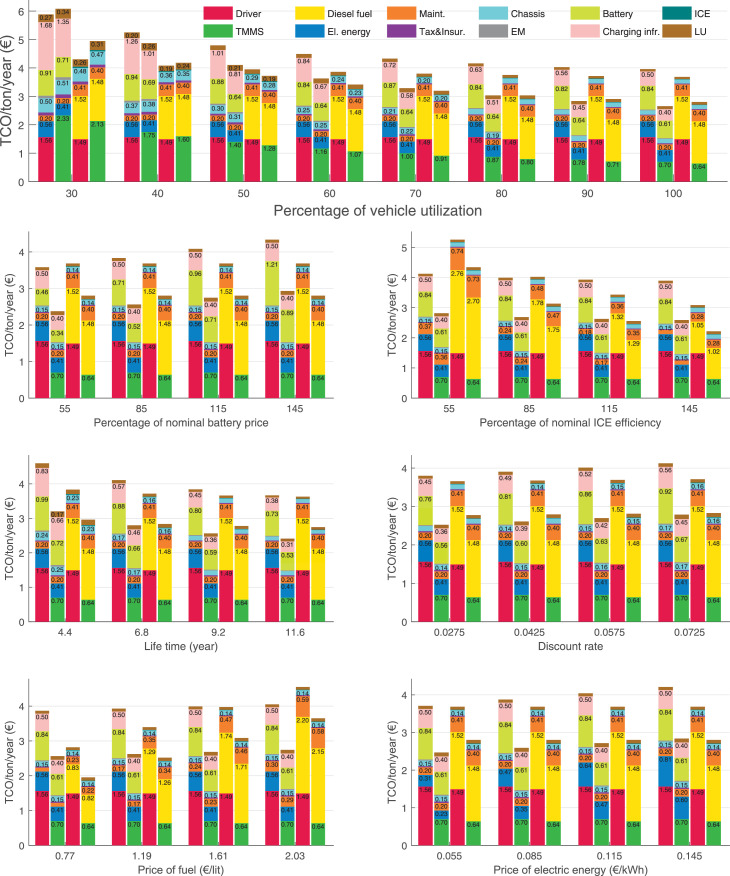
Tractor-semitrailer on a predominantly flat road of 40 km length; sensitivity of the TCO components of different vehicles and driving systems are shown for the optimum speed of the transportation scenario. A group of four bars from left to right represent BEHV HD, BEHV ADS-V, CHV HD and CHV ADS-DV, respectively. See Table 6 for the other vehicle sizes and road types.

**Fig. 142:**
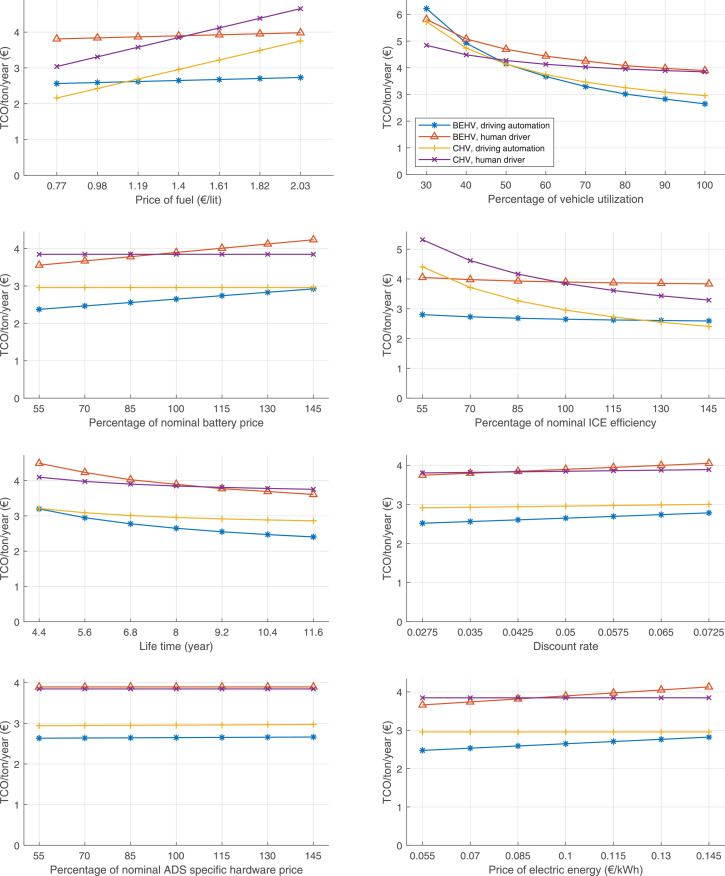
Nordic combination on a predominantly flat road of 40 km length; sensitivity of TCO to different parameters are shown for the optimum speed of the transportation scenario. See Table 6 for the other vehicle sizes and road types.

**Fig. 143:**
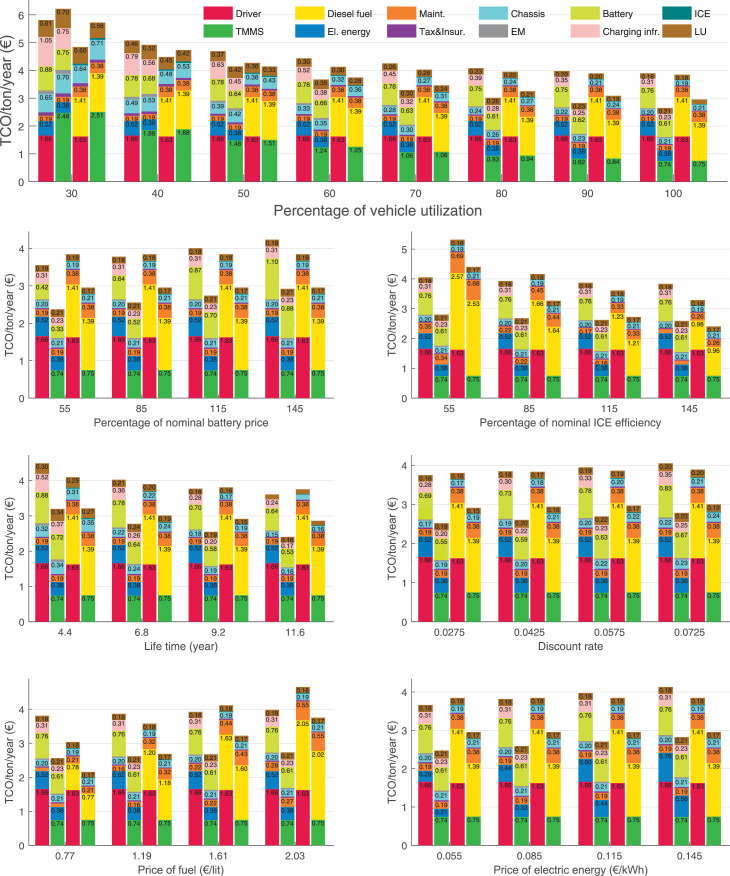
Nordic combination on a predominantly flat road of 40 km length; sensitivity of the TCO components of different vehicles and driving systems are shown for the optimum speed of the transportation scenario. A group of four bars from left to right represent BEHV HD, BEHV ADS-V, CHV HD and CHV ADS-DV, respectively. See Table 6 for the other vehicle sizes and road types.

**Fig. 144:**
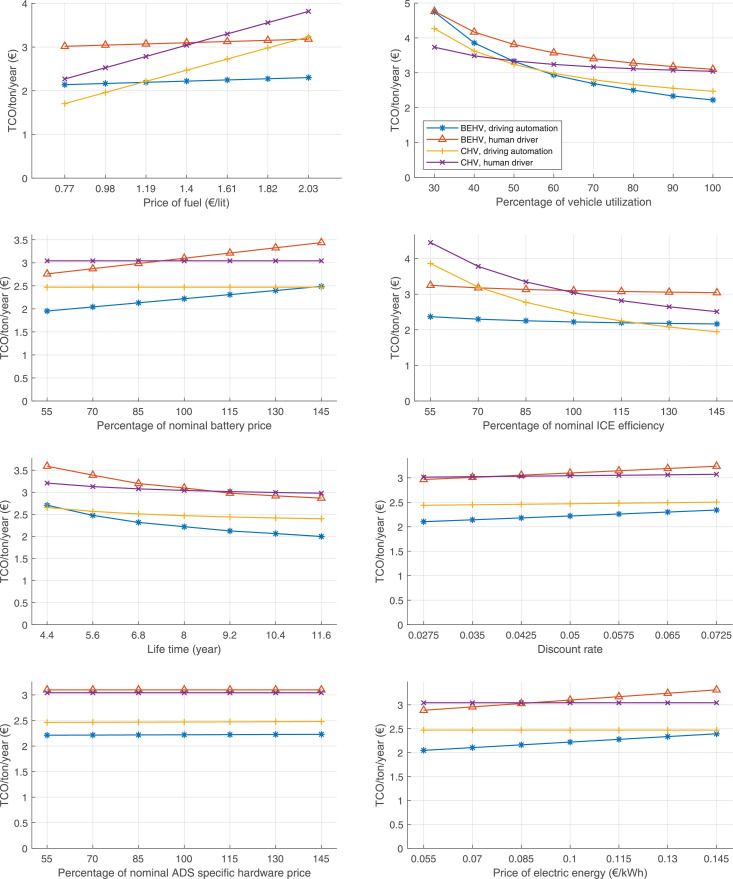
A-double on a predominantly flat road of 40 km length; sensitivity of TCO to different parameters are shown for the optimum speed of the transportation scenario. See Table 6 for the other vehicle sizes and road types.

**Fig. 145:**
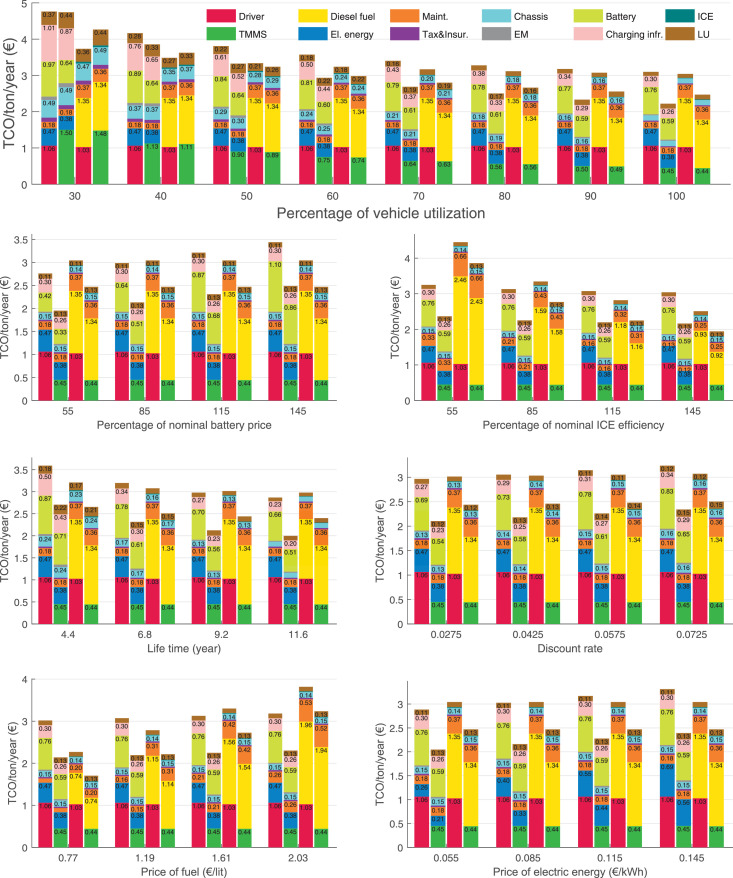
A-double on a predominantly flat road of 40 km length; sensitivity of the TCO components of different vehicles and driving systems are shown for the optimum speed of the transportation scenario. A group of four bars from left to right represent BEHV HD, BEHV ADS-V, CHV HD and CHV ADS-DV, respectively. See Table 6 for the other vehicle sizes and road types.

**Fig. 146:**
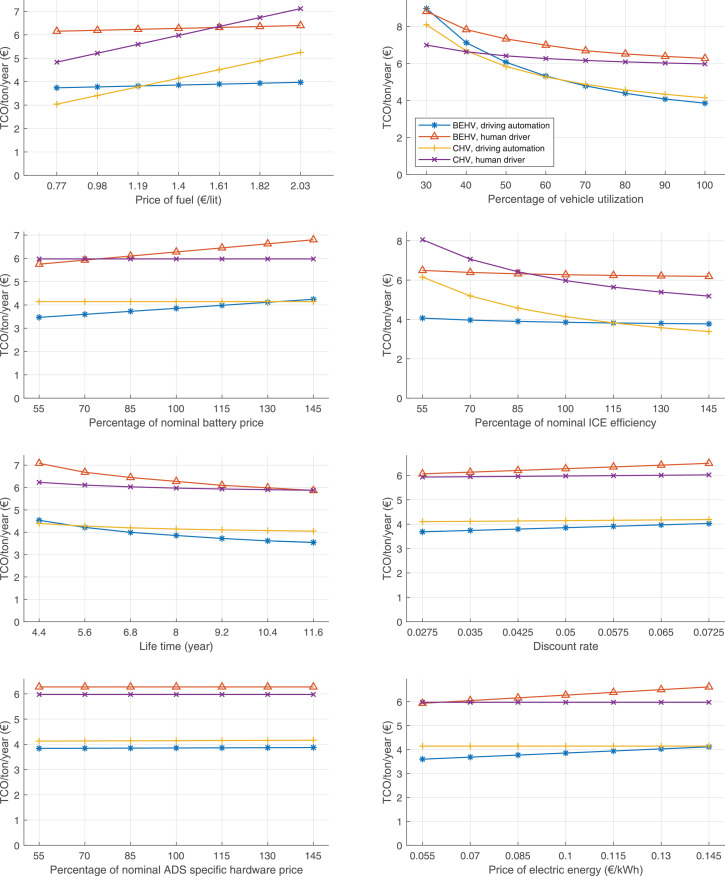
Rigid truck on a hilly road of 40 km length; sensitivity of TCO to different parameters are shown for the optimum speed of the transportation scenario. See Table 6 for the other vehicle sizes and road types.

**Fig. 147:**
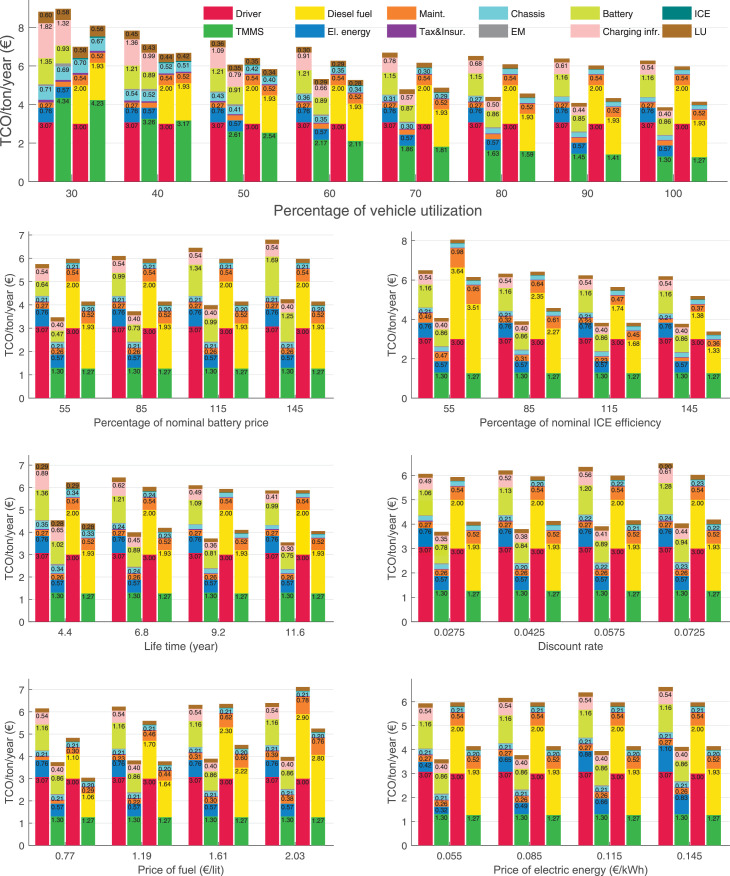
Rigid truck on a hilly road of 40 km length; sensitivity of the TCO components of different vehicles and driving systems are shown for the optimum speed of the transportation scenario. A group of four bars from left to right represent BEHV HD, BEHV ADS-V, CHV HD and CHV ADS-DV, respectively. See Table 6 for the other vehicle sizes and road types.

**Fig. 148:**
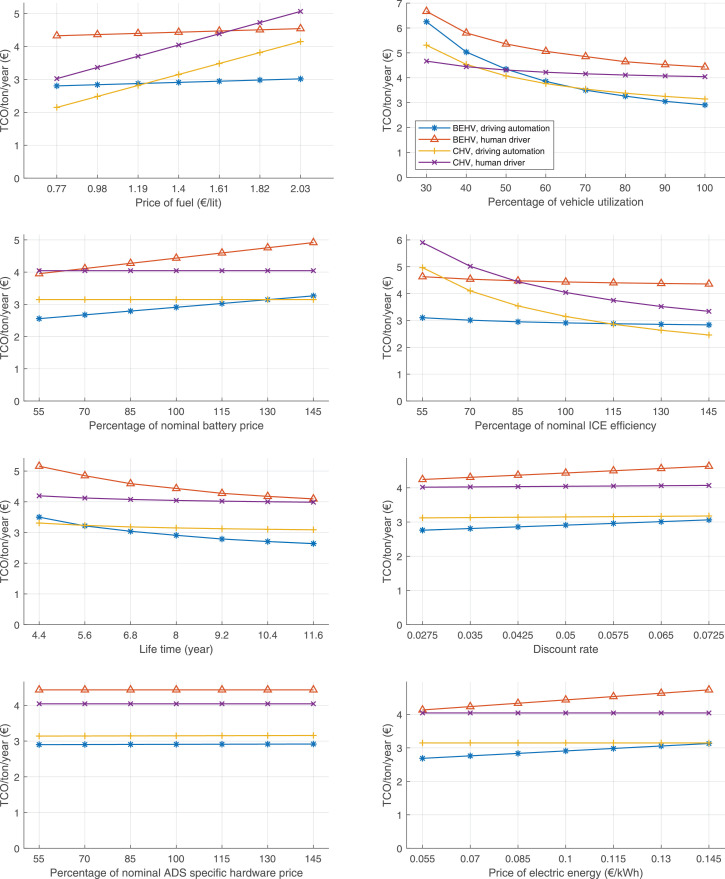
Tractor-semitrailer on a hilly road of 40 km length; sensitivity of TCO to different parameters are shown for the optimum speed of the transportation scenario. See Table 6 for the other vehicle sizes and road types.

**Fig. 149:**
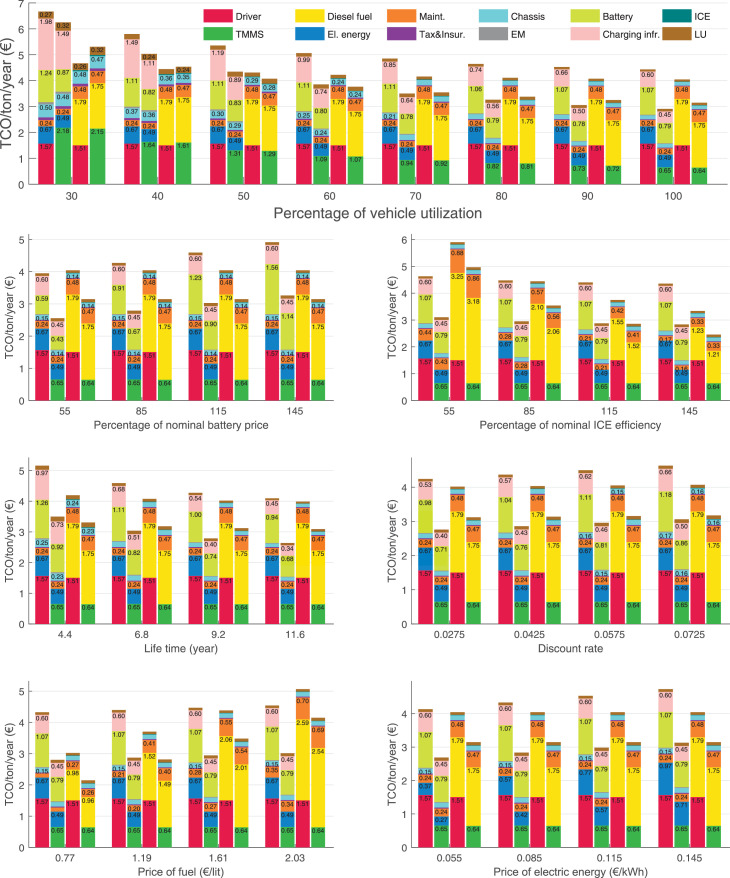
Tractor-semitrailer on a hilly road of 40 km length; sensitivity of the TCO components of different vehicles and driving systems are shown for the optimum speed of the transportation scenario. A group of four bars from left to right represent BEHV HD, BEHV ADS-V, CHV HD and CHV ADS-DV, respectively. See Table 6 for the other vehicle sizes and road types.

**Fig. 150:**
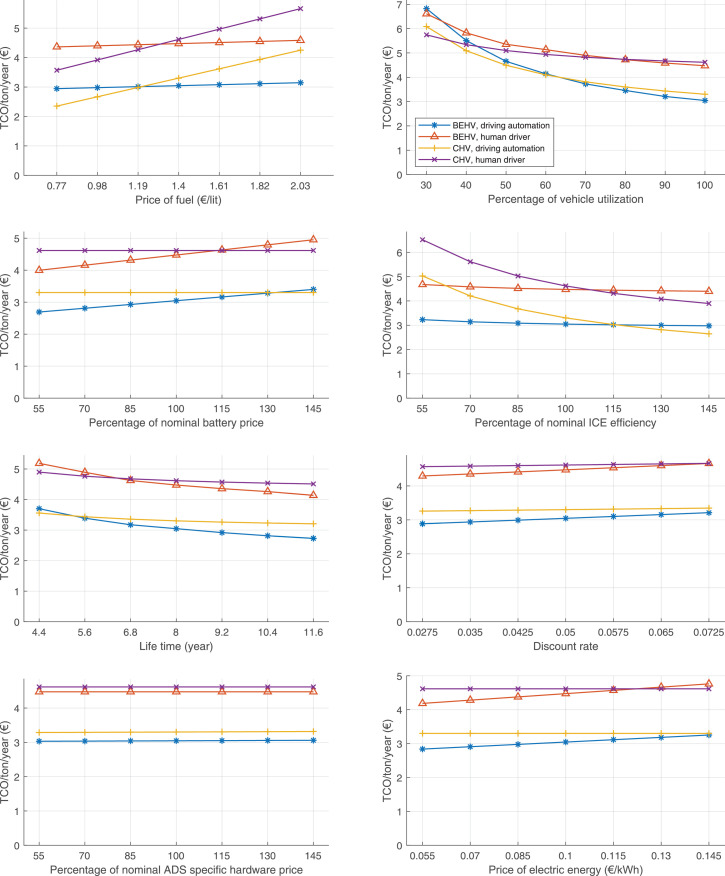
Nordic combination on a hilly road of 40 km length; sensitivity of TCO to different parameters are shown for the optimum speed of the transportation scenario. See Table 6 for the other vehicle sizes and road types.

**Fig. 151:**
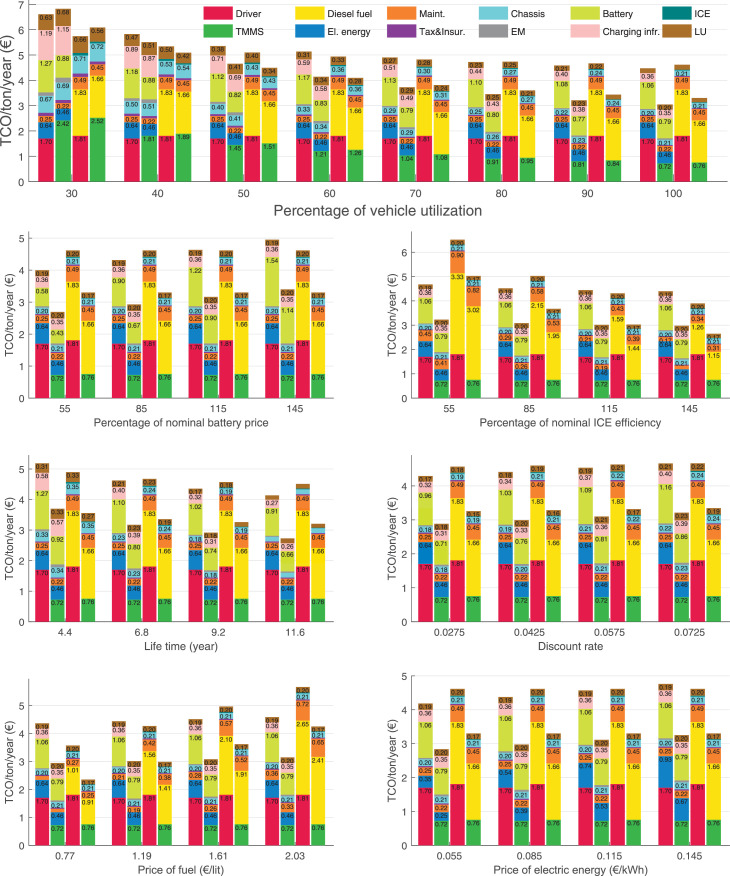
Nordic combination on a hilly road of 40 km length; sensitivity of the TCO components of different vehicles and driving systems are shown for the optimum speed of the transportation scenario. A group of four bars from left to right represent BEHV HD, BEHV ADS-V, CHV HD and CHV ADS-DV, respectively. See Table 6 for the other vehicle sizes and road types.

**Fig. 152:**
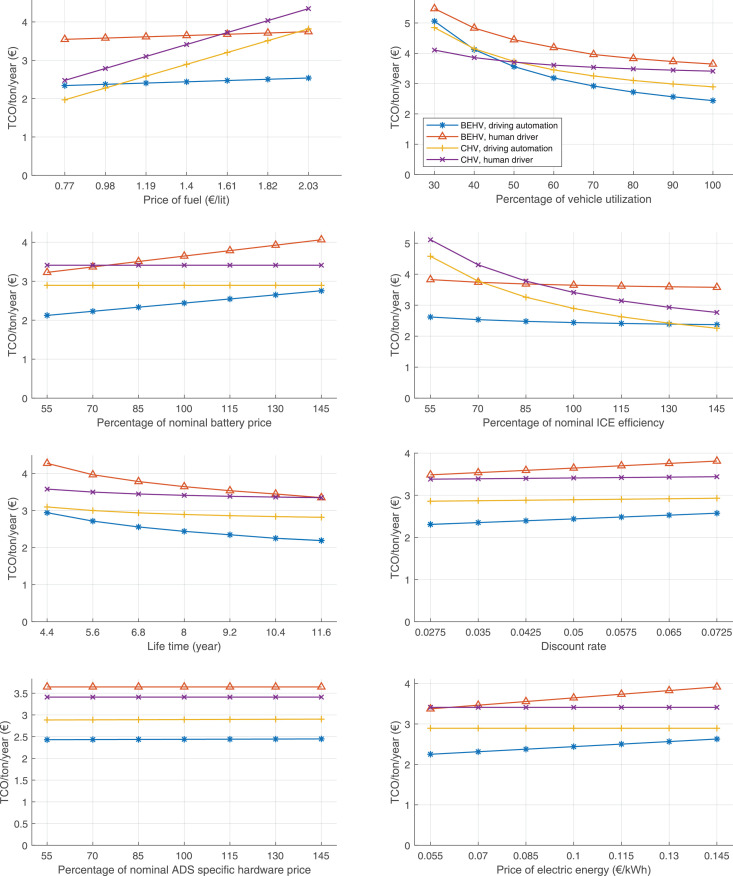
A-double on a hilly road of 40 km length; sensitivity of TCO to different parameters are shown for the optimum speed of the transportation scenario. See Table 6 for the other vehicle sizes and road types.

**Fig. 153:**
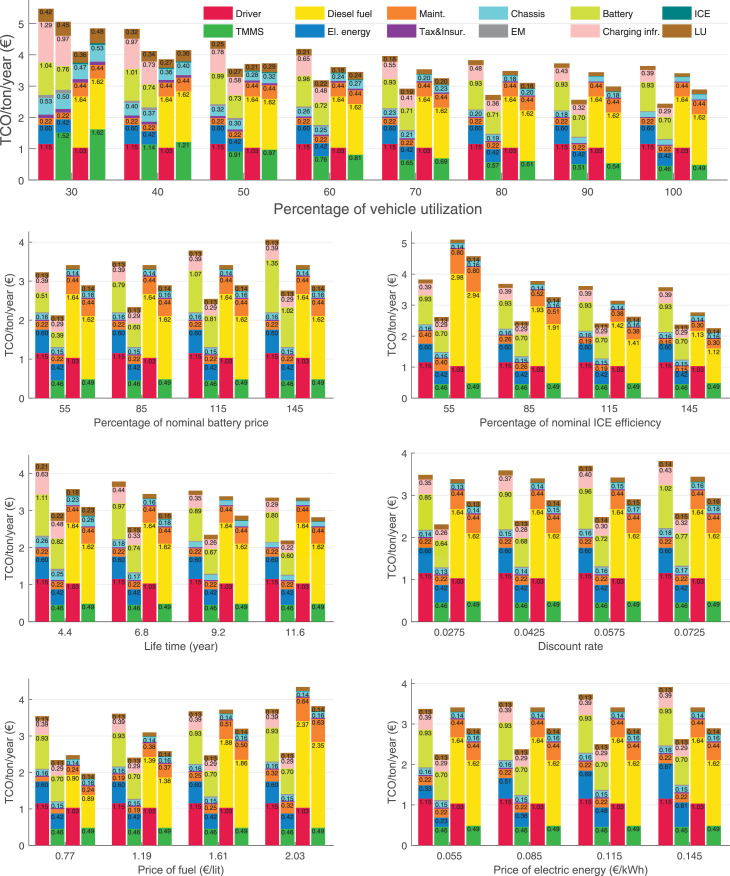
A-double on a hilly road of 40 km length; sensitivity of the TCO components of different vehicles and driving systems are shown for the optimum speed of the transportation scenario. A group of four bars from left to right represent BEHV HD, BEHV ADS-V, CHV HD and CHV ADS-DV, respectively. See Table 6 for the other vehicle sizes and road types.

**Fig. 154:**
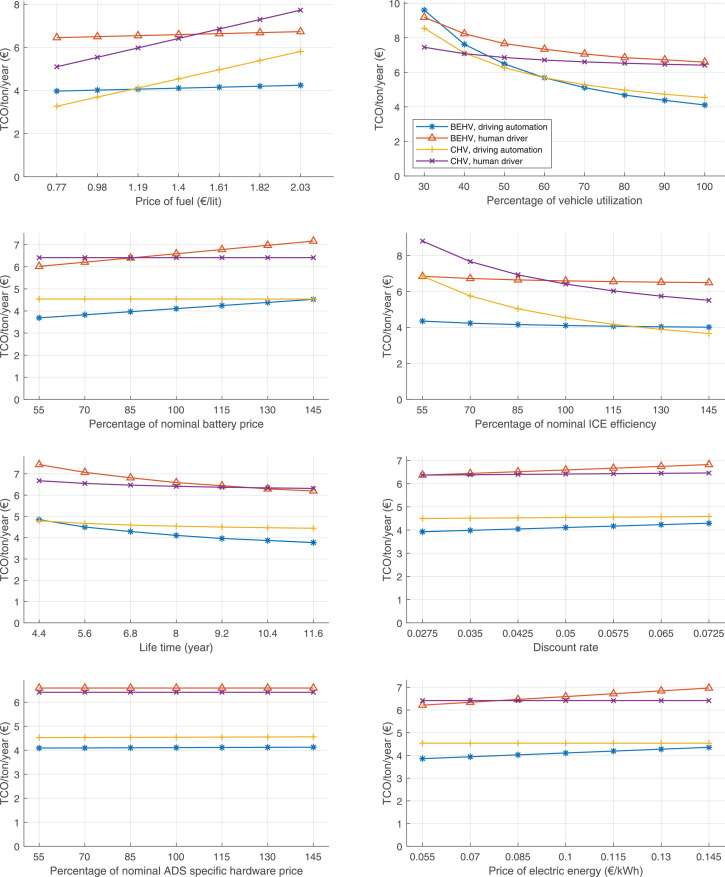
Rigid truck on a very hilly road of 40 km length; sensitivity of TCO to different parameters are shown for the optimum speed of the transportation scenario. See Table 6 for the other vehicle sizes and road types.

**Fig. 155:**
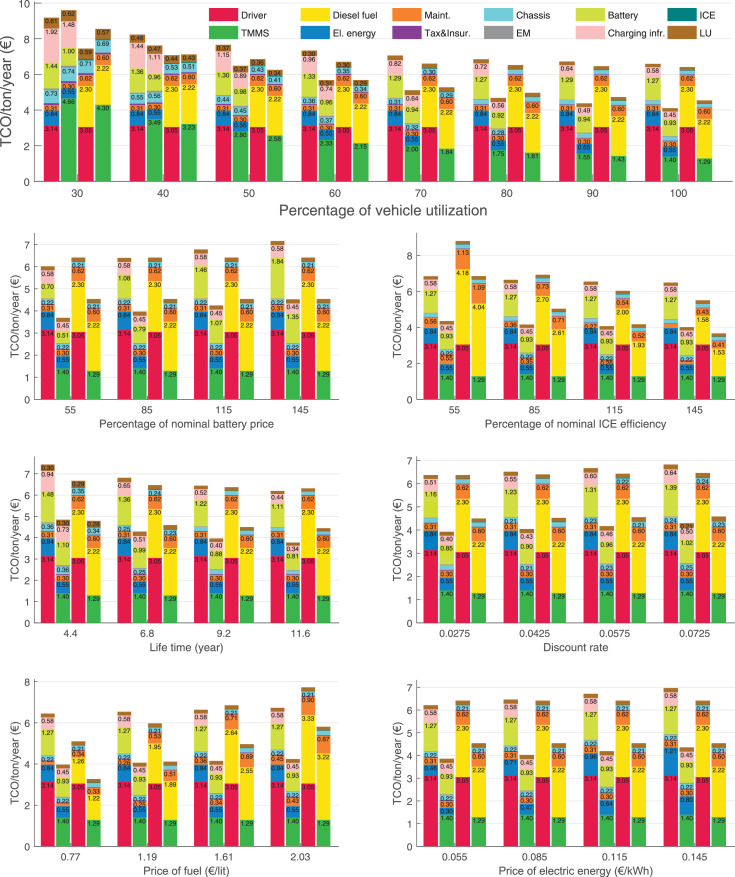
Rigid truck on a very hilly road of 40 km length; sensitivity of the TCO components of different vehicles and driving systems are shown for the optimum speed of the transportation scenario. A group of four bars from left to right represent BEHV HD, BEHV ADS-V, CHV HD and CHV ADS-DV, respectively. See Table 6 for the other vehicle sizes and road types.

**Fig. 156:**
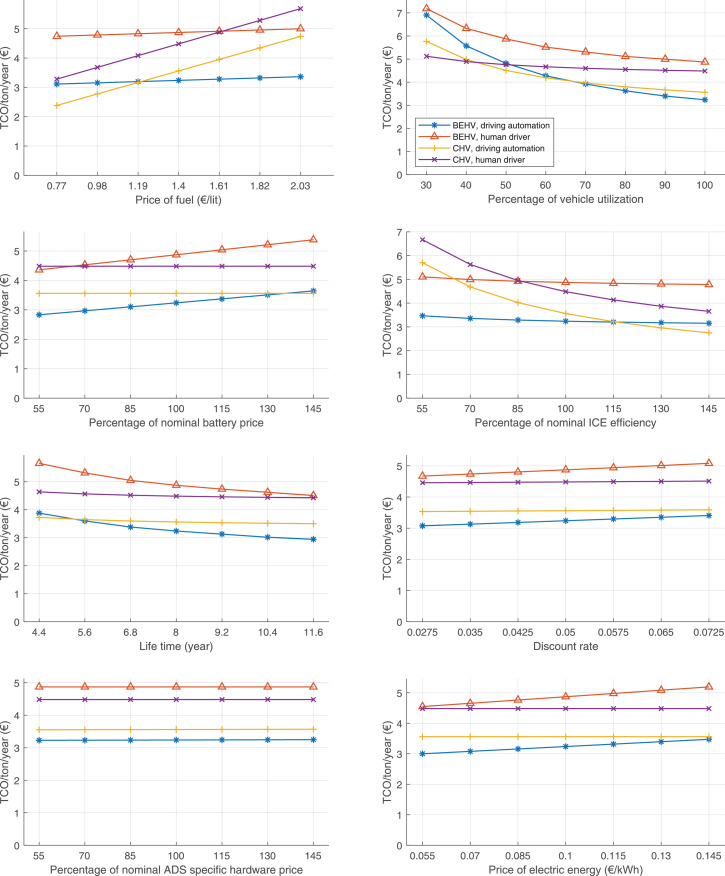
Tractor-semitrailer on a very hilly road of 40 km length; sensitivity of TCO to different parameters are shown for the optimum speed of the transportation scenario. See Table 6 for the other vehicle sizes and road types.

**Fig. 157:**
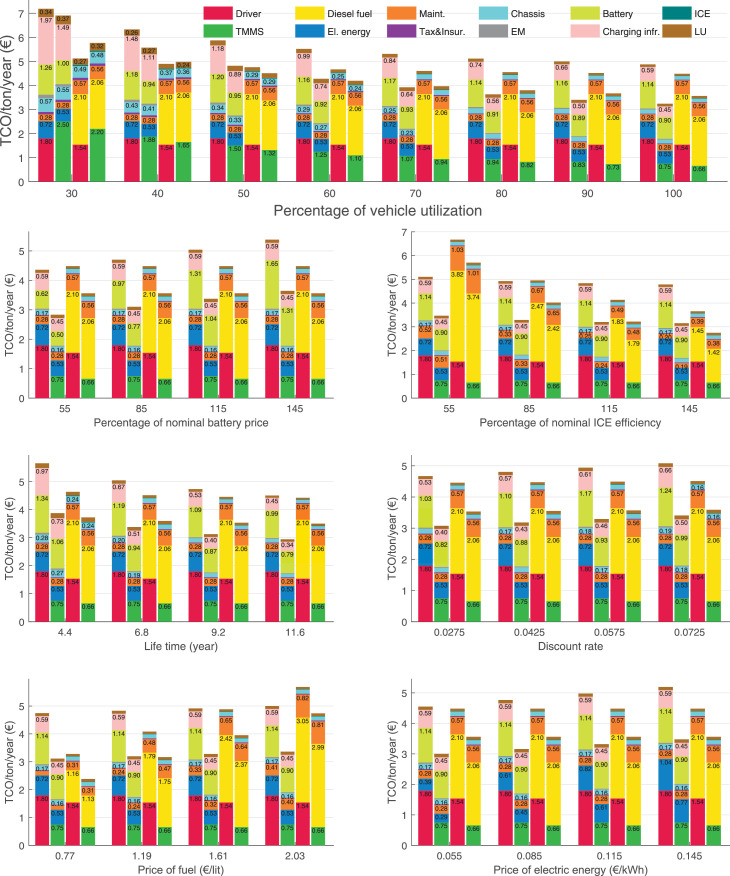
Tractor-semitrailer on a very hilly road of 40 km length; sensitivity of the TCO components of different vehicles and driving systems are shown for the optimum speed of the transportation scenario. A group of four bars from left to right represent BEHV HD, BEHV ADS-V, CHV HD and CHV ADS-DV, respectively. See Table 6 for the other vehicle sizes and road types.

**Fig. 158:**
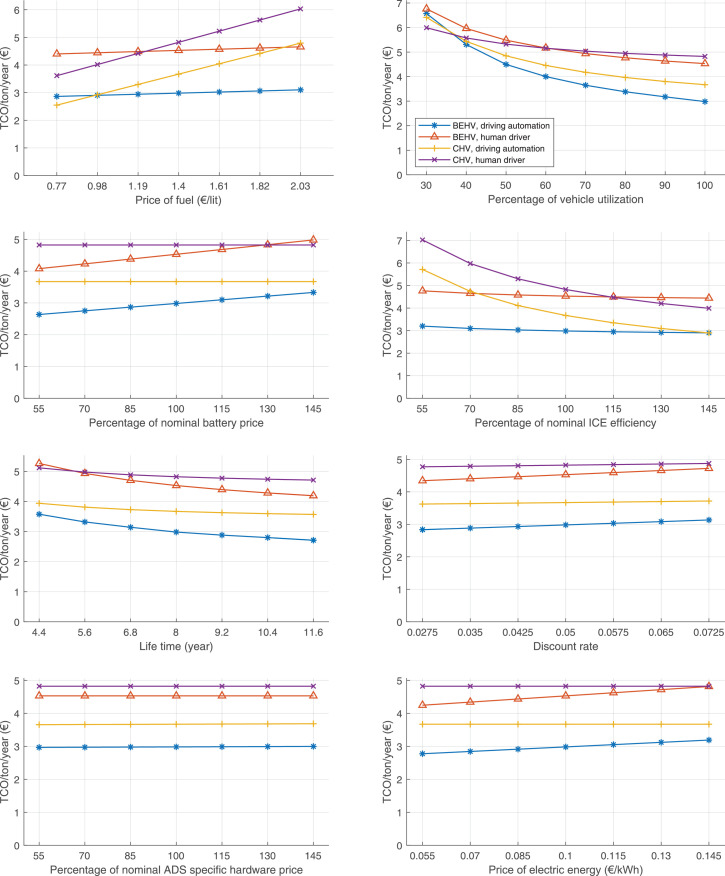
Nordic combination on a very hilly road of 40 km length; sensitivity of TCO to different parameters are shown for the optimum speed of the transportation scenario. See Table 6 for the other vehicle sizes and road types.

**Fig. 159:**
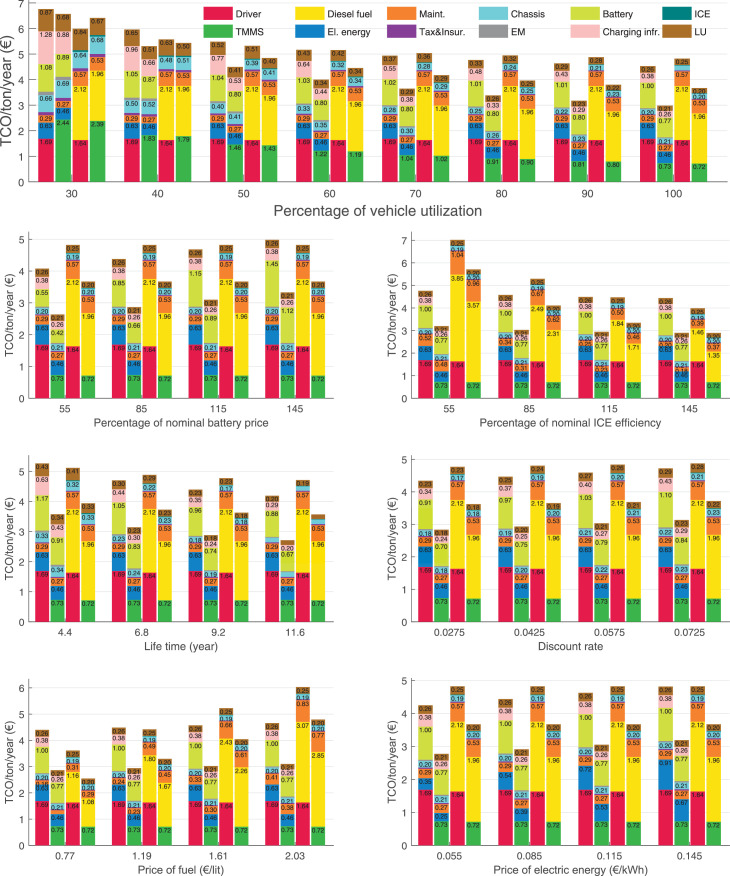
Nordic combination on a very hilly road of 40 km length; sensitivity of the TCO components of different vehicles and driving systems are shown for the optimum speed of the transportation scenario. A group of four bars from left to right represent BEHV HD, BEHV ADS-V, CHV HD and CHV ADS-DV, respectively. See Table 6 for the other vehicle sizes and road types.

**Fig. 160:**
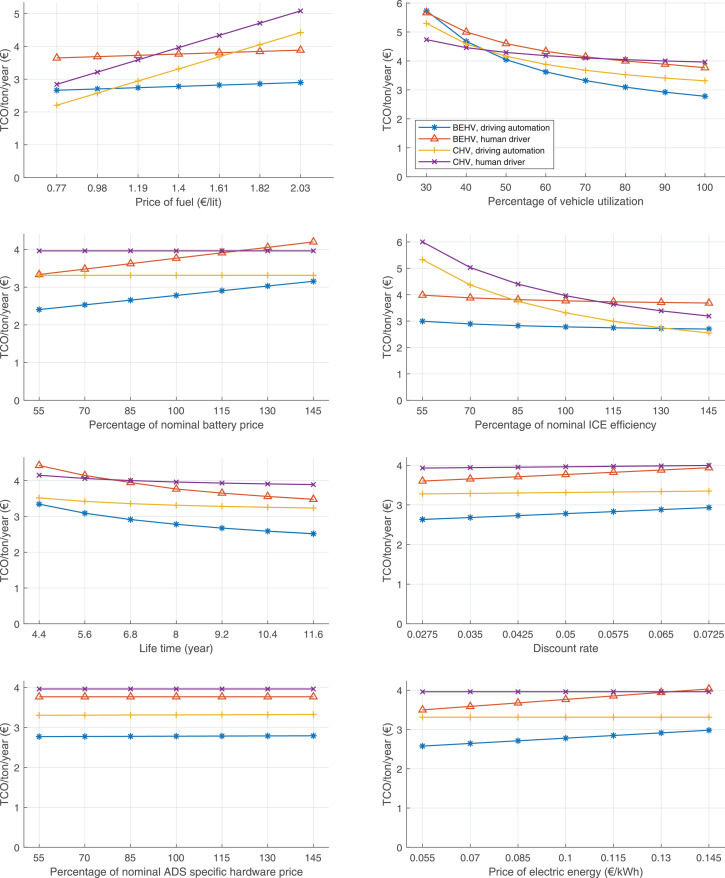
A-double on a very hilly road of 40 km length; sensitivity of TCO to different parameters are shown for the optimum speed of the transportation scenario. See Table 6 for the other vehicle sizes and road types.

**Fig. 161:**
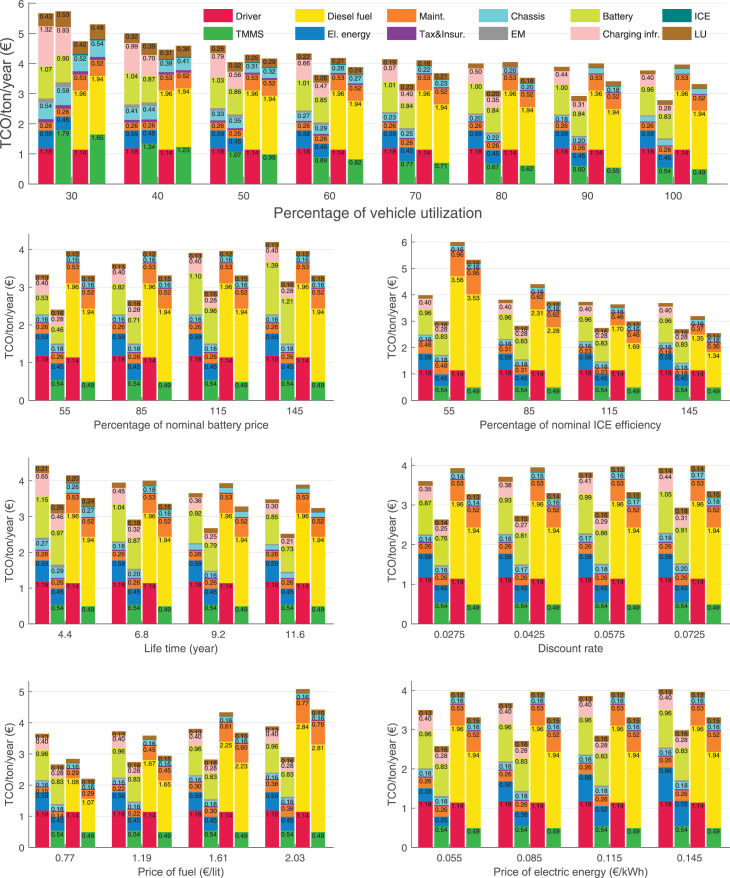
A-double on a very hilly road of 40 km length; sensitivity of the TCO components of different vehicles and driving systems are shown for the optimum speed of the transportation scenario. A group of four bars from left to right represent BEHV HD, BEHV ADS-V, CHV HD and CHV ADS-DV, respectively. See Table 6 for the other vehicle sizes and road types.

**Fig. 162:**
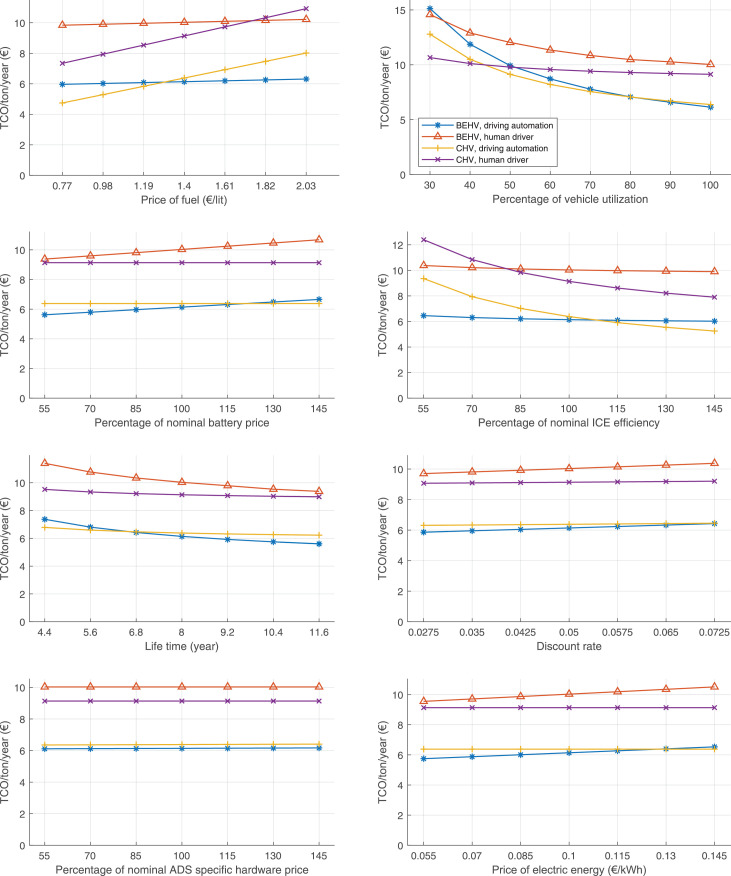
Rigid truck on a flat road of 80 km length; sensitivity of TCO to different parameters are shown for the optimum speed of the transportation scenario. See Table 6 for the other vehicle sizes and road types.

**Fig. 163:**
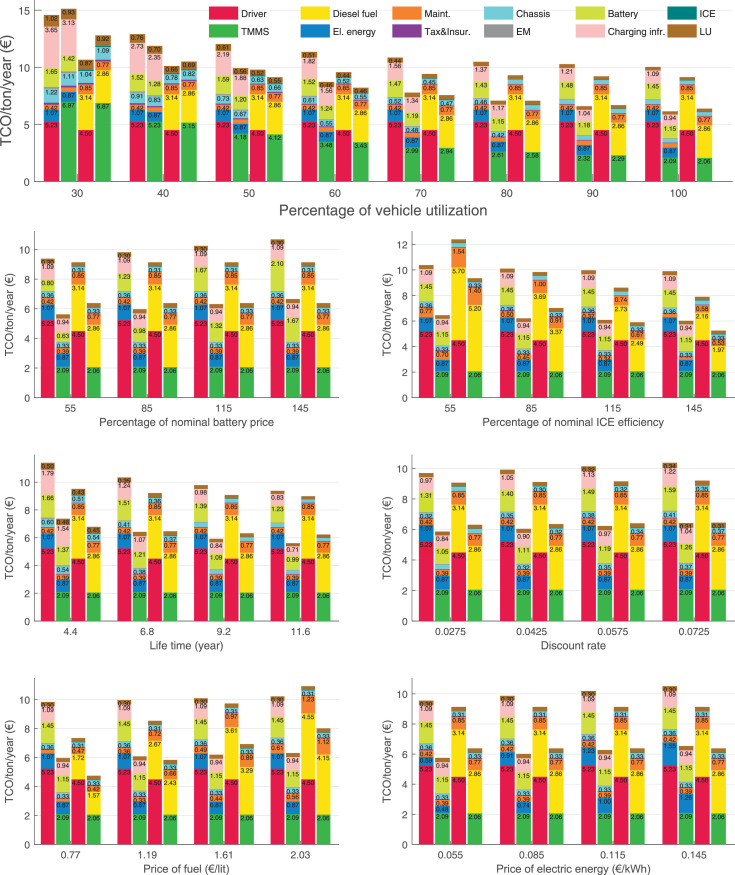
Rigid truck on a flat road of 80 km length; sensitivity of the TCO components of different vehicles and driving systems are shown for the optimum speed of the transportation scenario. A group of four bars from left to right represent BEHV HD, BEHV ADS-V, CHV HD and CHV ADS-DV, respectively. See Table 6 for the other vehicle sizes and road types.

**Fig. 164:**
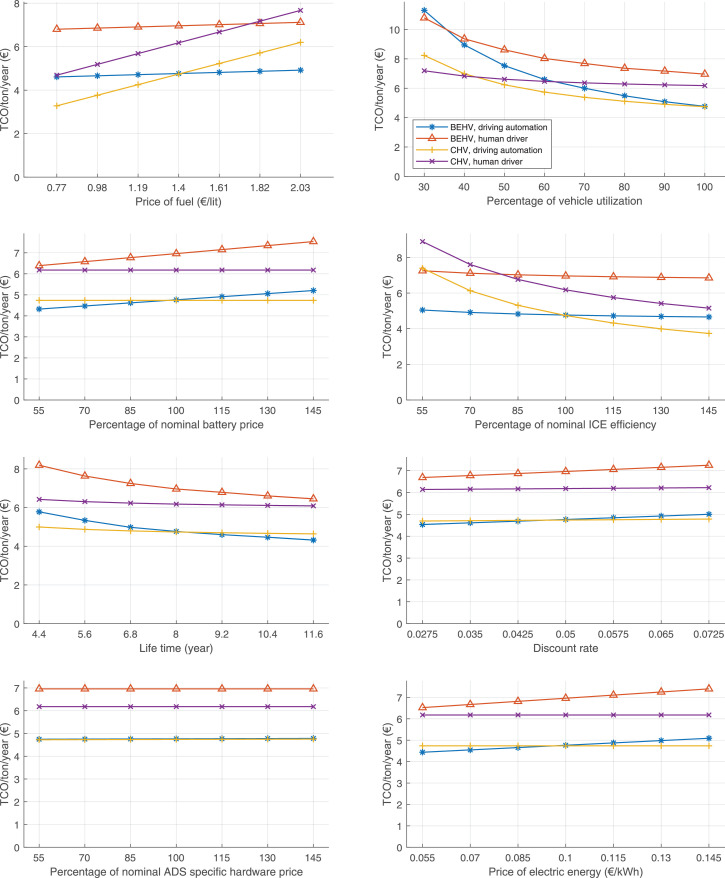
Tractor-semitrailer on a flat road of 80 km length; sensitivity of TCO to different parameters are shown for the optimum speed of the transportation scenario. See Table 6 for the other vehicle sizes and road types.

**Fig. 165:**
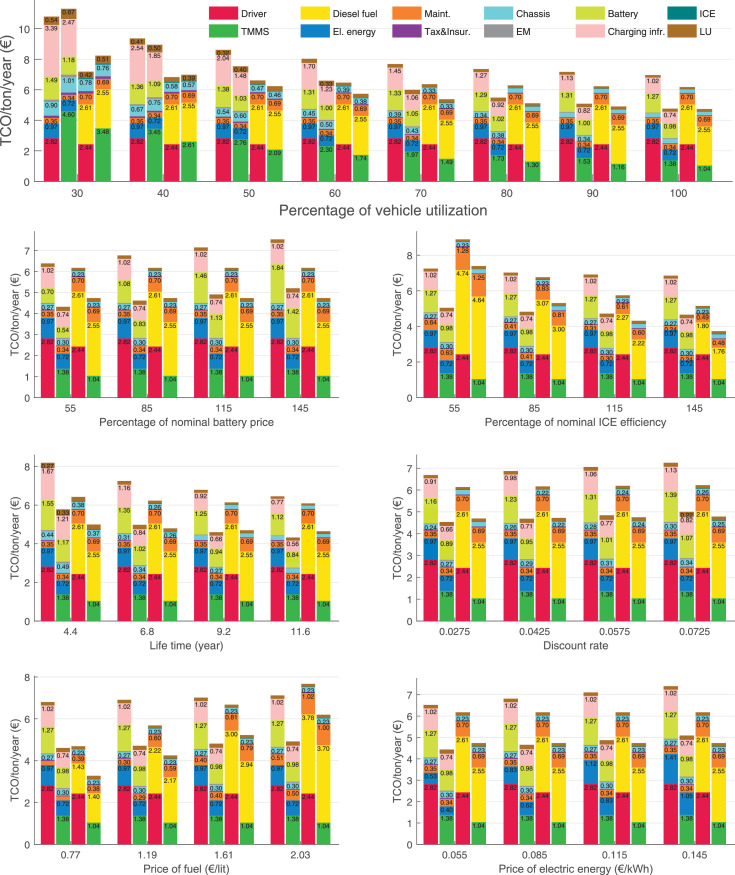
Tractor-semitrailer on a flat road of 80 km length; sensitivity of the TCO components of different vehicles and driving systems are shown for the optimum speed of the transportation scenario. A group of four bars from left to right represent BEHV HD, BEHV ADS-V, CHV HD and CHV ADS-DV, respectively. See Table 6 for the other vehicle sizes and road types.

**Fig. 166:**
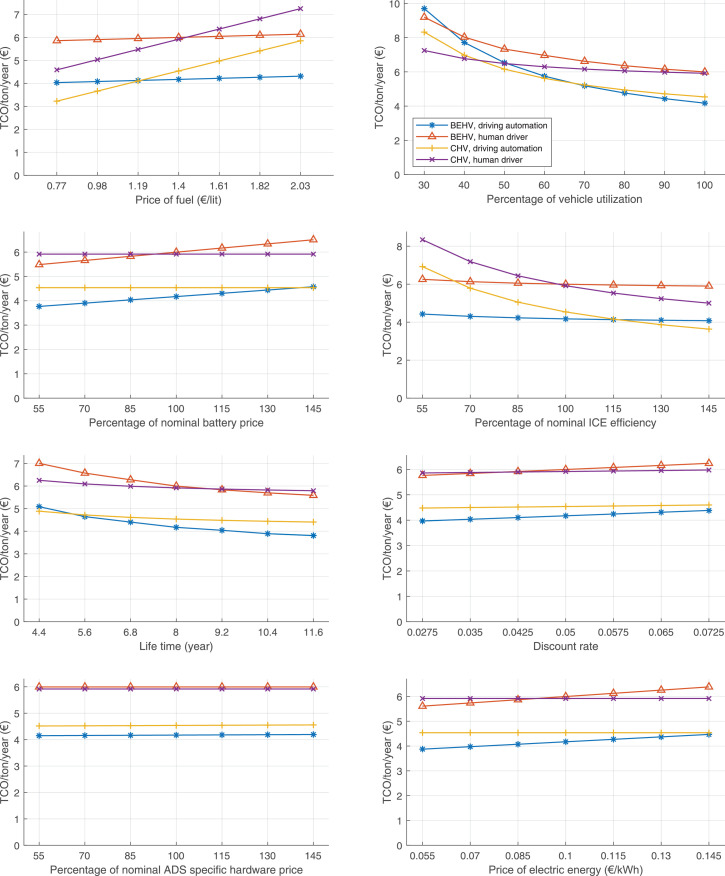
Nordic combination on a flat road of 80 km length; sensitivity of TCO to different parameters are shown for the optimum speed of the transportation scenario. See Table 6 for the other vehicle sizes and road types.

**Fig. 167:**
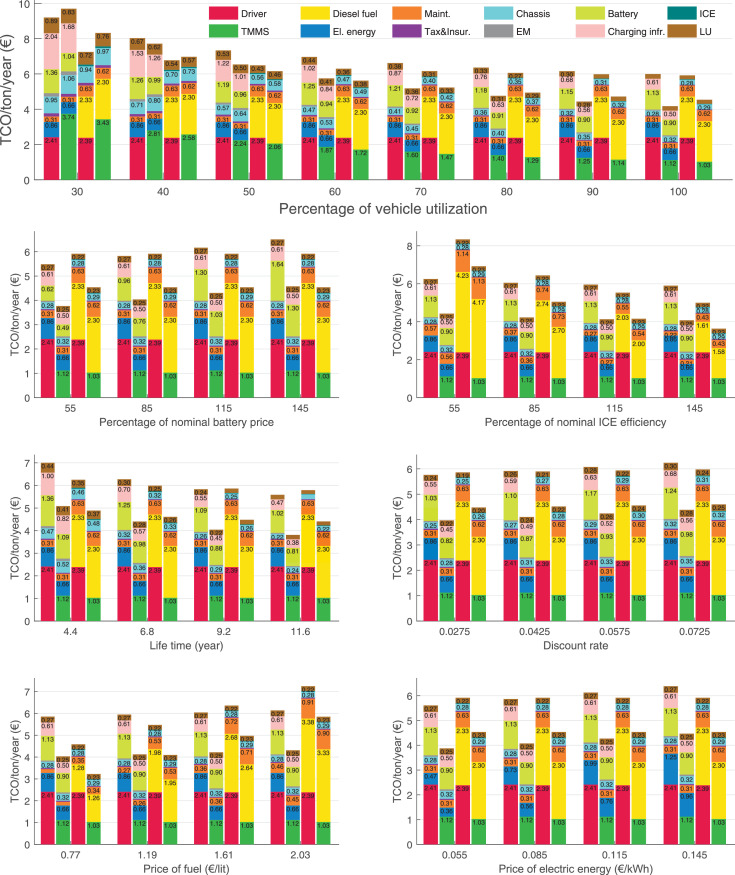
Nordic combination on a flat road of 80 km length; sensitivity of the TCO components of different vehicles and driving systems are shown for the optimum speed of the transportation scenario. A group of four bars from left to right represent BEHV HD, BEHV ADS-V, CHV HD and CHV ADS-DV, respectively. See Table 6 for the other vehicle sizes and road types.

**Fig. 168:**
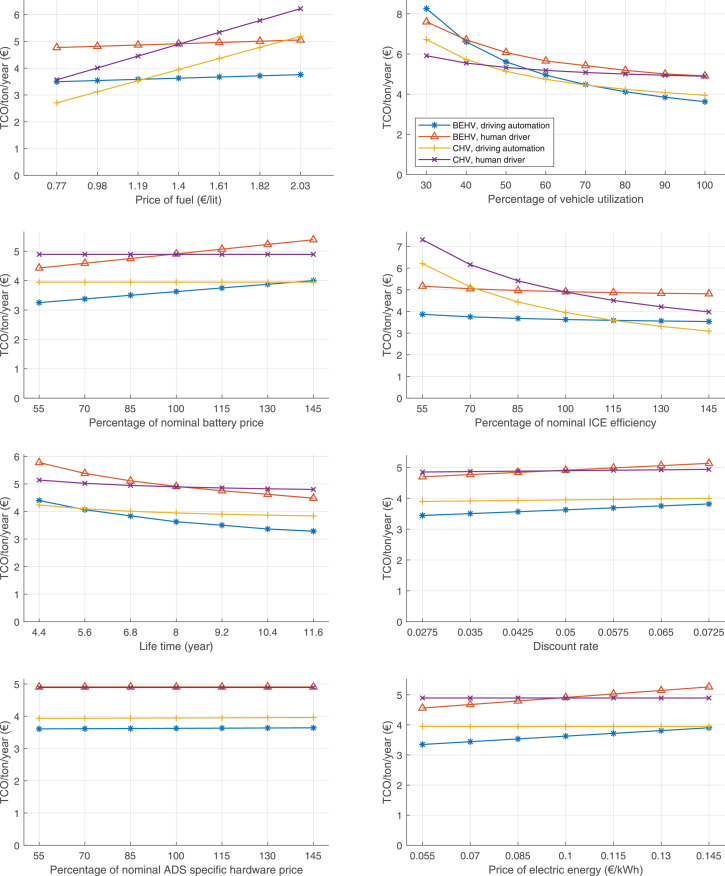
A-double on a flat road of 80 km length; sensitivity of TCO to different parameters are shown for the optimum speed of the transportation scenario. See Table 6 for the other vehicle sizes and road types.

**Fig. 169:**
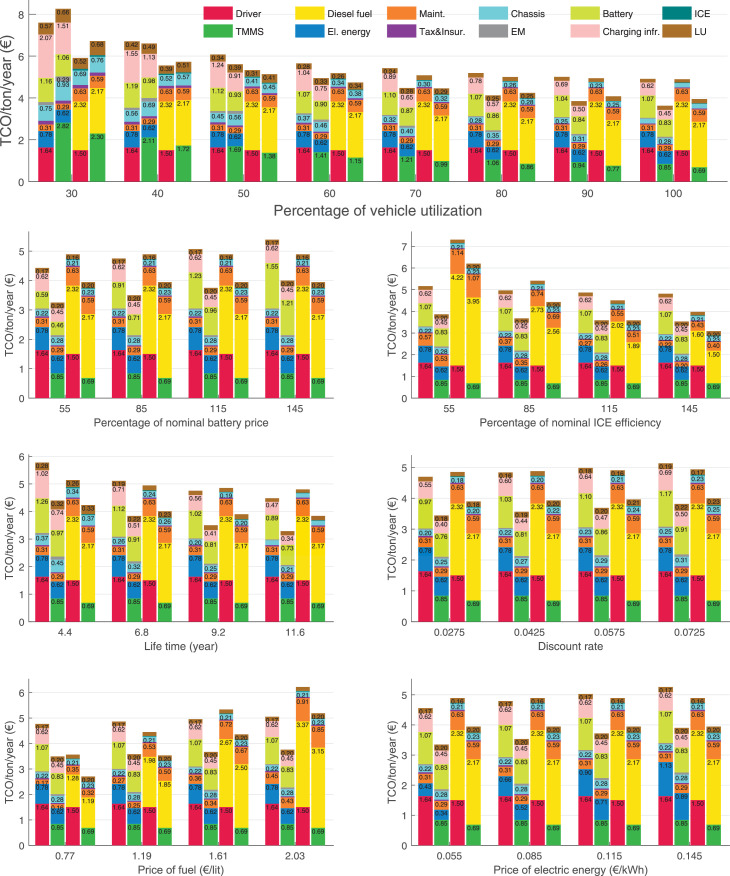
A-double on a flat road of 80 km length; sensitivity of the TCO components of different vehicles and driving systems are shown for the optimum speed of the transportation scenario. A group of four bars from left to right represent BEHV HD, BEHV ADS-V, CHV HD and CHV ADS-DV, respectively. See Table 6 for the other vehicle sizes and road types.

**Fig. 170:**
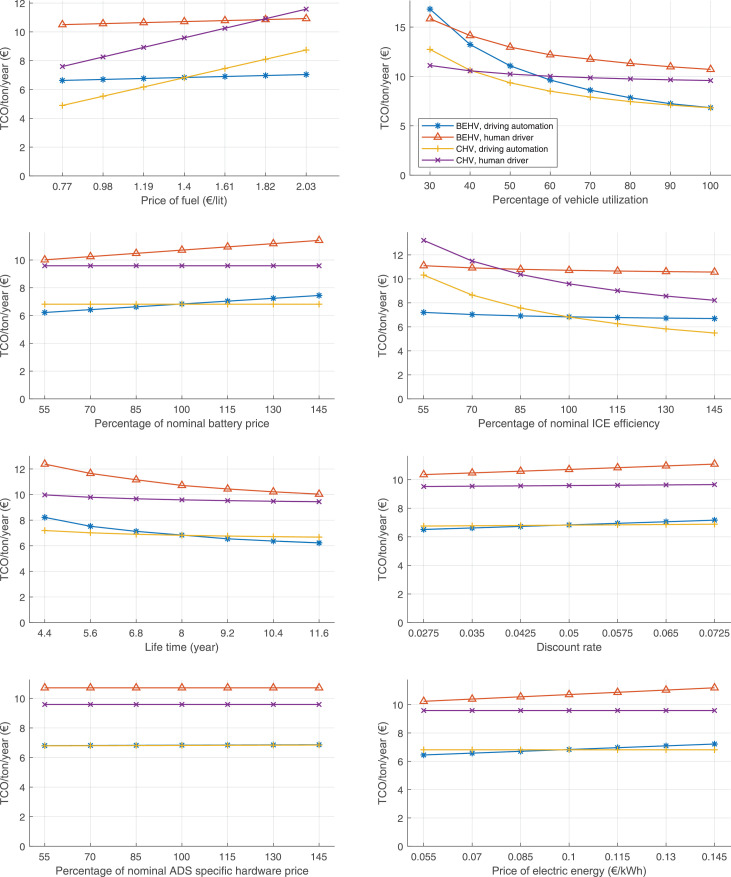
Rigid truck on a predominantly flat road of 80 km length; sensitivity of TCO to different parameters are shown for the optimum speed of the transportation scenario. See Table 6 for the other vehicle sizes and road types.

**Fig. 171:**
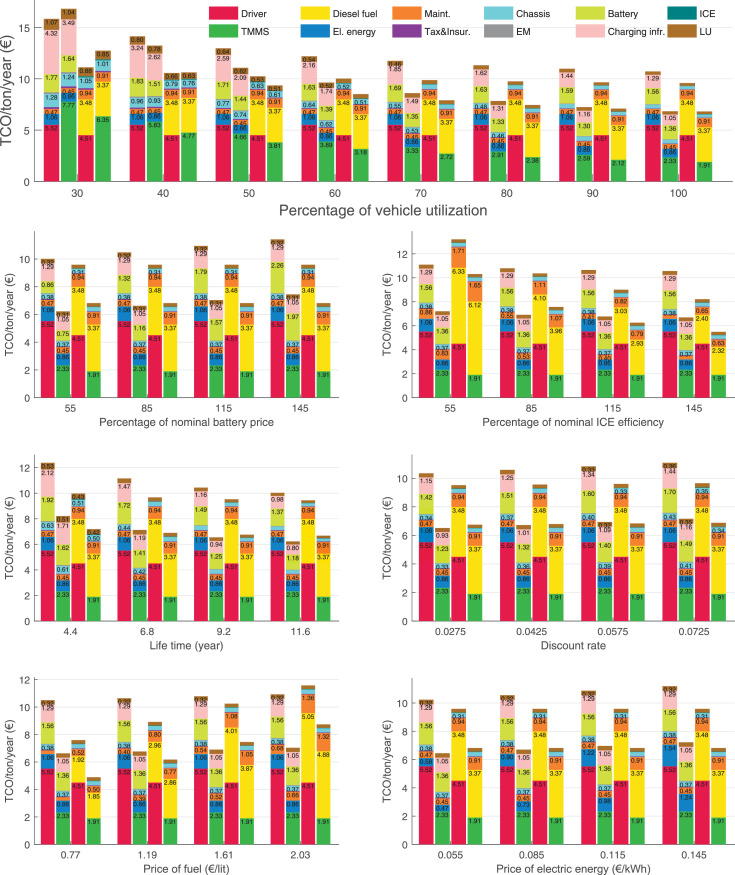
Rigid truck on a predominantly flat road of 80 km length; sensitivity of the TCO components of different vehicles and driving systems are shown for the optimum speed of the transportation scenario. A group of four bars from left to right represent BEHV HD, BEHV ADS-V, CHV HD and CHV ADS-DV, respectively. See Table 6 for the other vehicle sizes and road types.

**Fig. 172:**
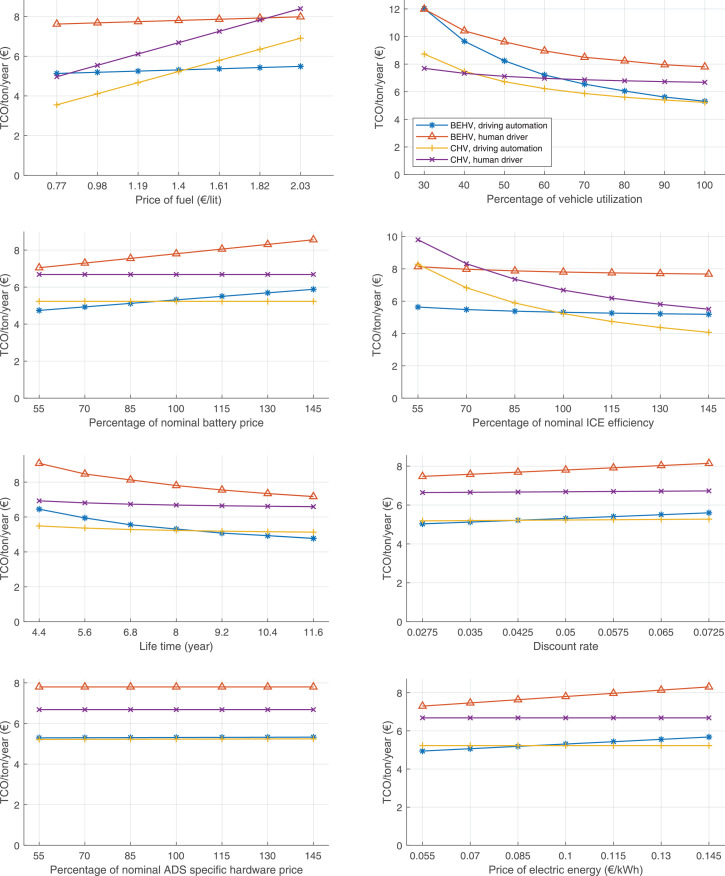
Tractor-semitrailer on a predominantly flat road of 80 km length; sensitivity of TCO to different parameters are shown for the optimum speed of the transportation scenario. See Table 6 for the other vehicle sizes and road types.

**Fig. 173:**
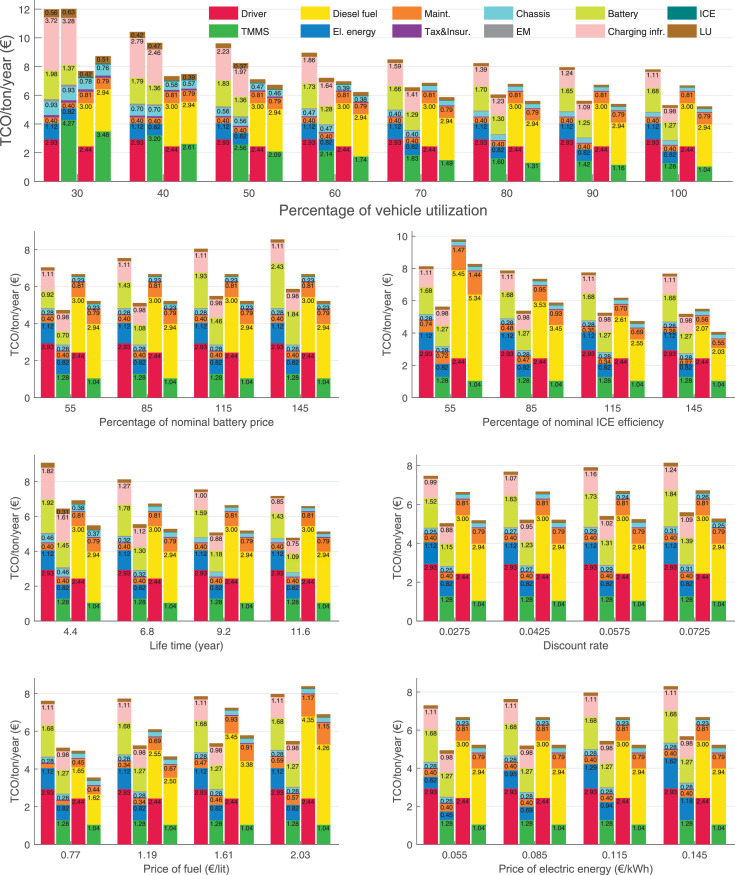
Tractor-semitrailer on a predominantly flat road of 80 km length; sensitivity of the TCO components of different vehicles and driving systems are shown for the optimum speed of the transportation scenario. A group of four bars from left to right represent BEHV HD, BEHV ADS-V, CHV HD and CHV ADS-DV, respectively. See Table 6 for the other vehicle sizes and road types.

**Fig. 174:**
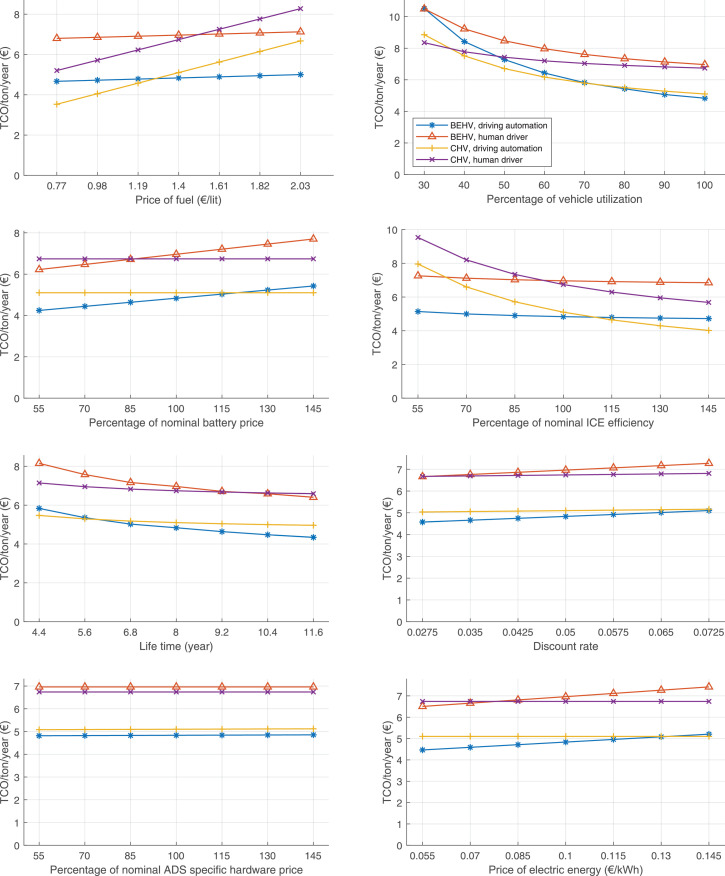
Nordic combination on a predominantly flat road of 80 km length; sensitivity of TCO to different parameters are shown for the optimum speed of the transportation scenario. See Table 6 for the other vehicle sizes and road types.

**Fig. 175:**
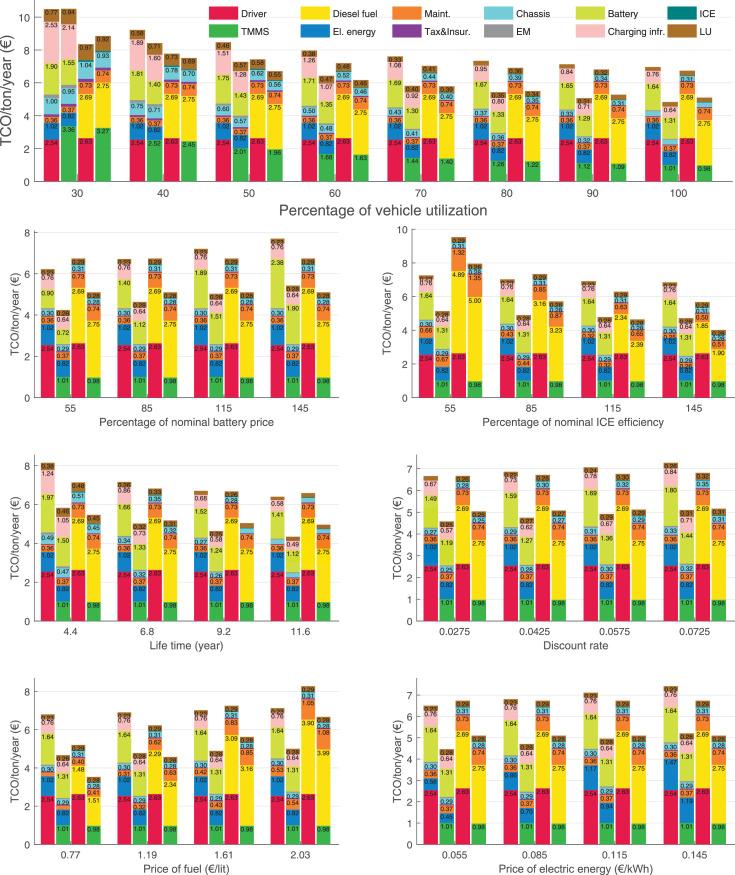
Nordic combination on a predominantly flat road of 80 km length; sensitivity of the TCO components of different vehicles and driving systems are shown for the optimum speed of the transportation scenario. A group of four bars from left to right represent BEHV HD, BEHV ADS-V, CHV HD and CHV ADS-DV, respectively. See Table 6 for the other vehicle sizes and road types.

**Fig. 176:**
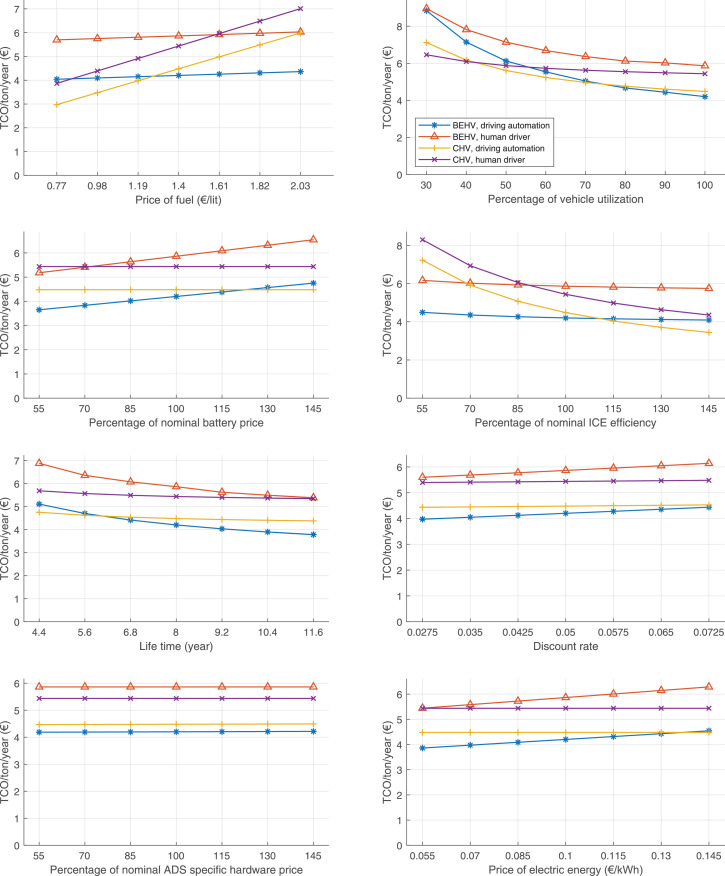
A-double on a predominantly flat road of 80 km length; sensitivity of TCO to different parameters are shown for the optimum speed of the transportation scenario. See Table 6 for the other vehicle sizes and road types.

**Fig. 177:**
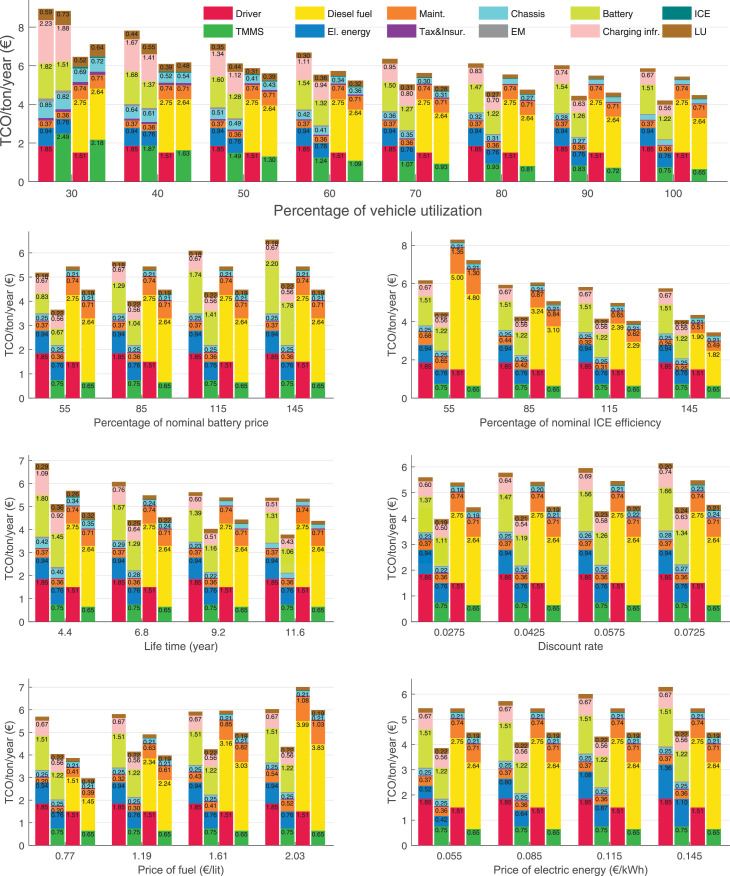
A-double on a predominantly flat road of 80 km length; sensitivity of the TCO components of different vehicles and driving systems are shown for the optimum speed of the transportation scenario. A group of four bars from left to right represent BEHV HD, BEHV ADS-V, CHV HD and CHV ADS-DV, respectively. See Table 6 for the other vehicle sizes and road types.

**Fig. 178:**
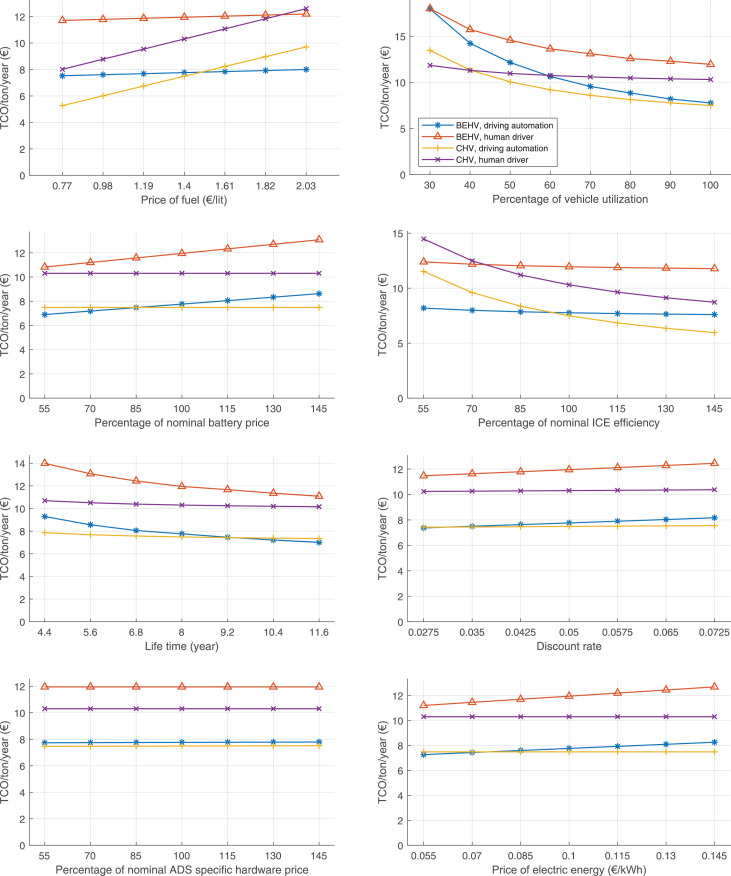
Rigid truck on a hilly road of 80 km length; sensitivity of TCO to different parameters are shown for the optimum speed of the transportation scenario. See Table 6 for the other vehicle sizes and road types.

**Fig. 179:**
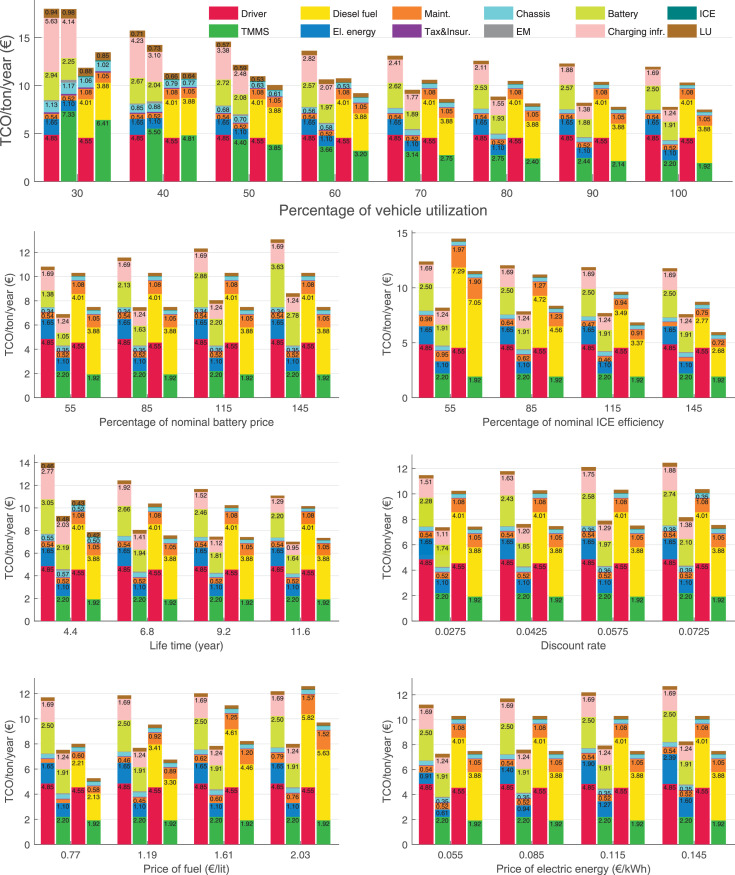
Rigid truck on a hilly road of 80 km length; sensitivity of the TCO components of different vehicles and driving systems are shown for the optimum speed of the transportation scenario. A group of four bars from left to right represent BEHV HD, BEHV ADS-V, CHV HD and CHV ADS-DV, respectively. See Table 6 for the other vehicle sizes and road types.

**Fig. 180:**
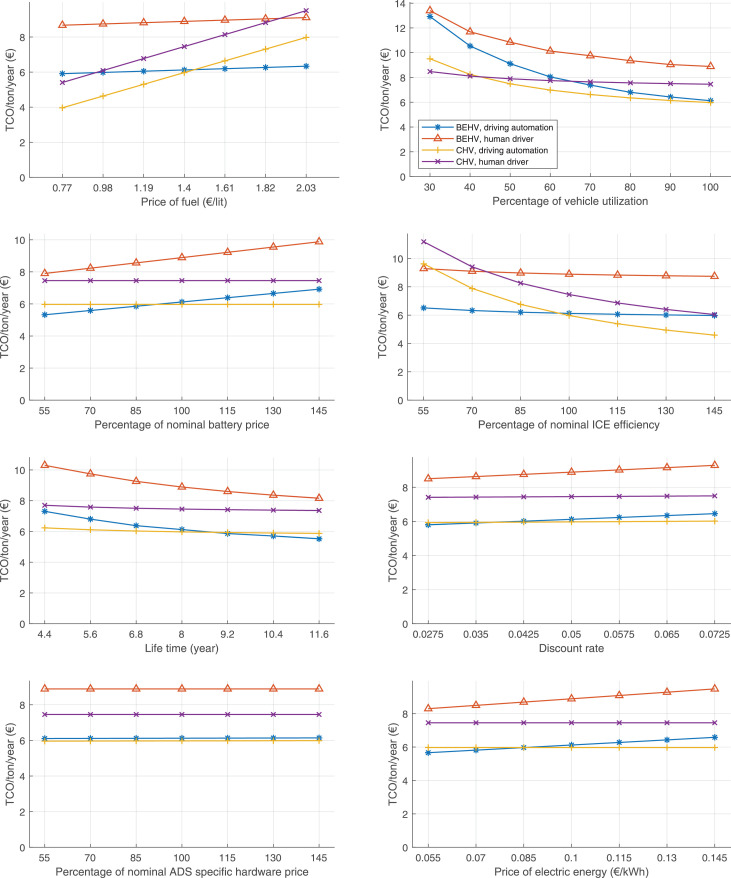
Tractor-semitrailer on a hilly road of 80 km length; sensitivity of TCO to different parameters are shown for the optimum speed of the transportation scenario. See Table 6 for the other vehicle sizes and road types.

**Fig. 181:**
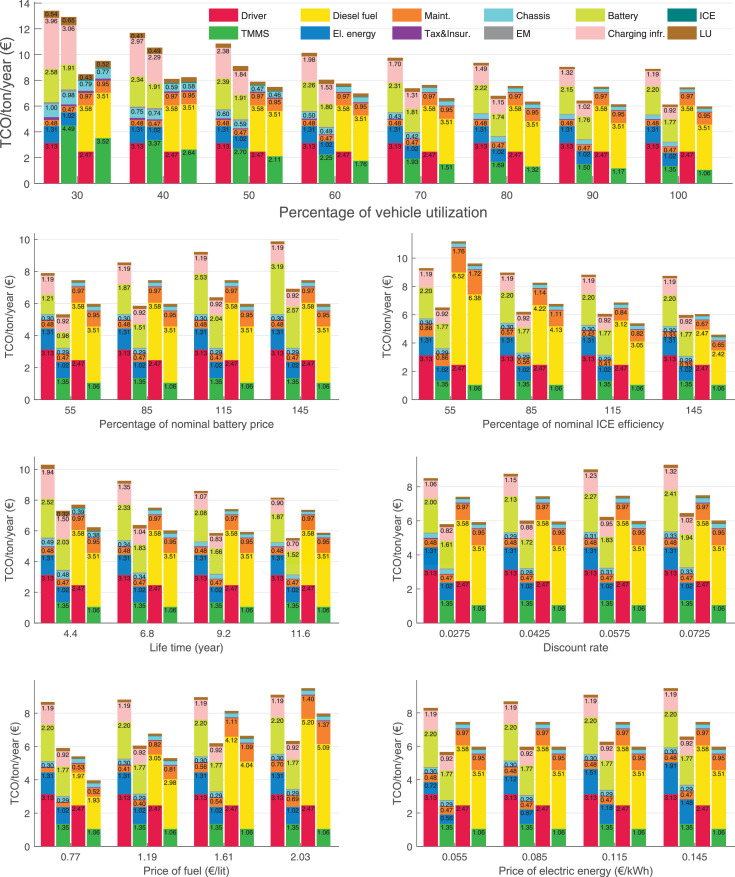
Tractor-semitrailer on a hilly road of 80 km length; sensitivity of the TCO components of different vehicles and driving systems are shown for the optimum speed of the transportation scenario. A group of four bars from left to right represent BEHV HD, BEHV ADS-V, CHV HD and CHV ADS-DV, respectively. See Table 6 for the other vehicle sizes and road types.

**Fig. 182:**
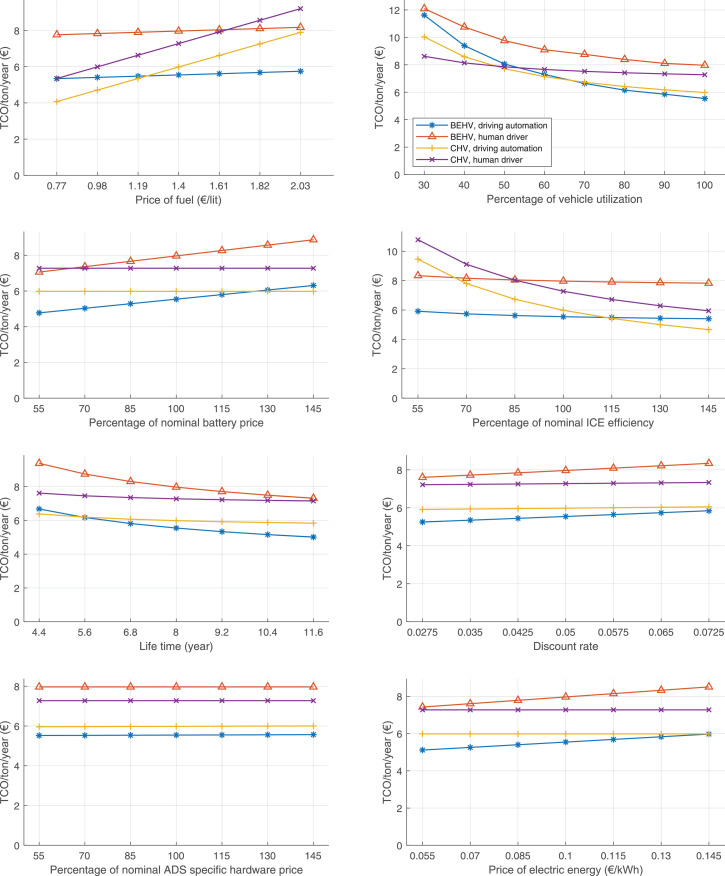
Nordic combination on a hilly road of 80 km length; sensitivity of TCO to different parameters are shown for the optimum speed of the transportation scenario. See Table 6 for the other vehicle sizes and road types.

**Fig. 183:**
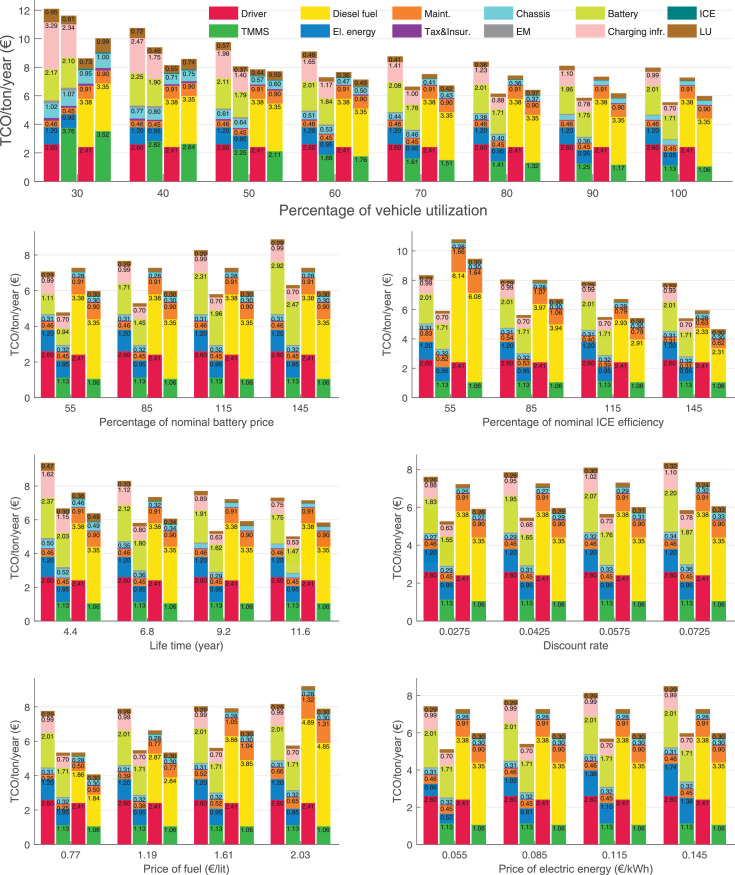
Nordic combination on a hilly road of 80 km length; sensitivity of the TCO components of different vehicles and driving systems are shown for the optimum speed of the transportation scenario. A group of four bars from left to right represent BEHV HD, BEHV ADS-V, CHV HD and CHV ADS-DV, respectively. See Table 6 for the other vehicle sizes and road types.

**Fig. 184:**
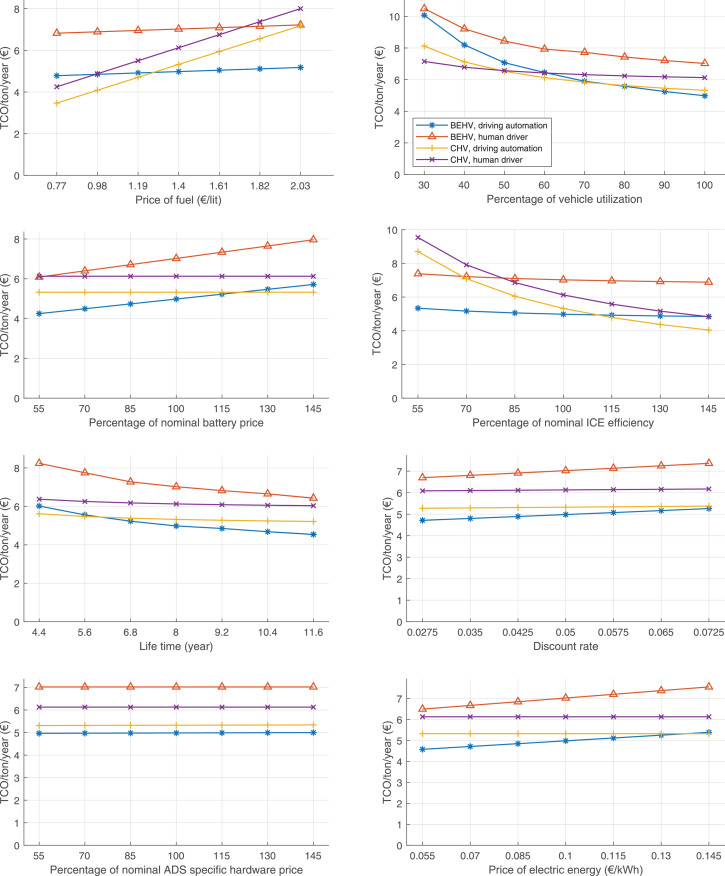
A-double on a hilly road of 80 km length; sensitivity of TCO to different parameters are shown for the optimum speed of the transportation scenario. See Table 6 for the other vehicle sizes and road types.

**Fig. 185:**
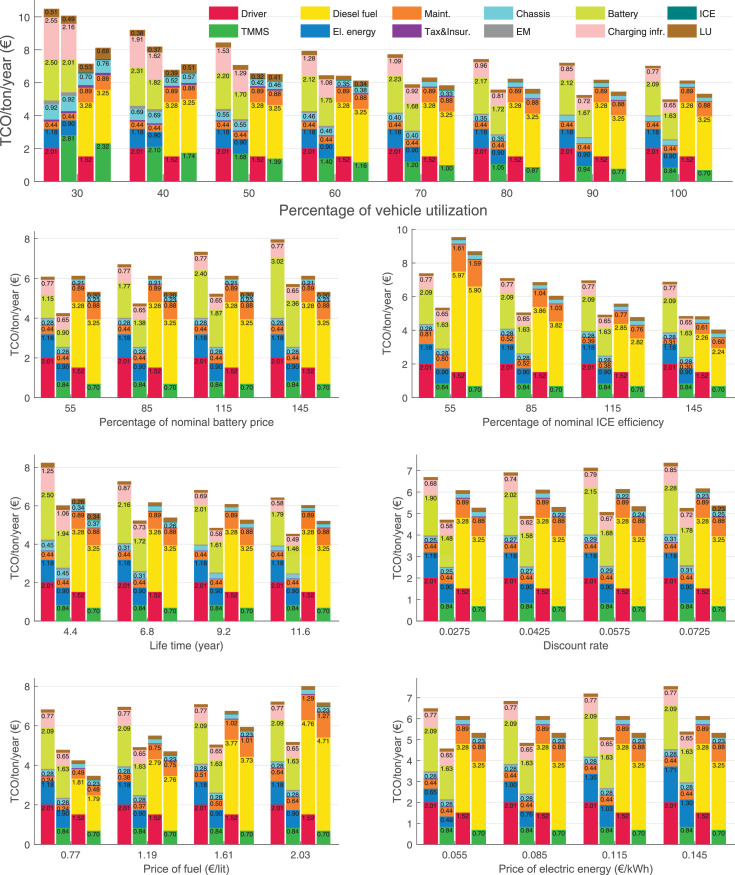
A-double on a hilly road of 80 km length; sensitivity of the TCO components of different vehicles and driving systems are shown for the optimum speed of the transportation scenario. A group of four bars from left to right represent BEHV HD, BEHV ADS-V, CHV HD and CHV ADS-DV, respectively. See Table 6 for the other vehicle sizes and road types.

**Fig. 186:**
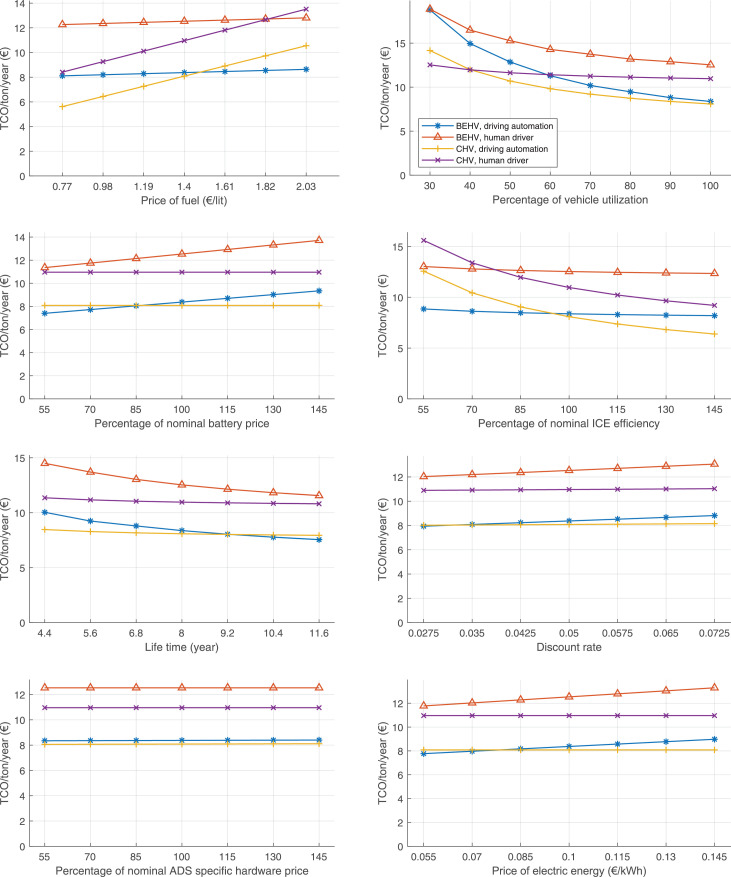
Rigid truck on a very hilly road of 80 km length; sensitivity of TCO to different parameters are shown for the optimum speed of the transportation scenario. See Table 6 for the other vehicle sizes and road types.

**Fig. 187:**
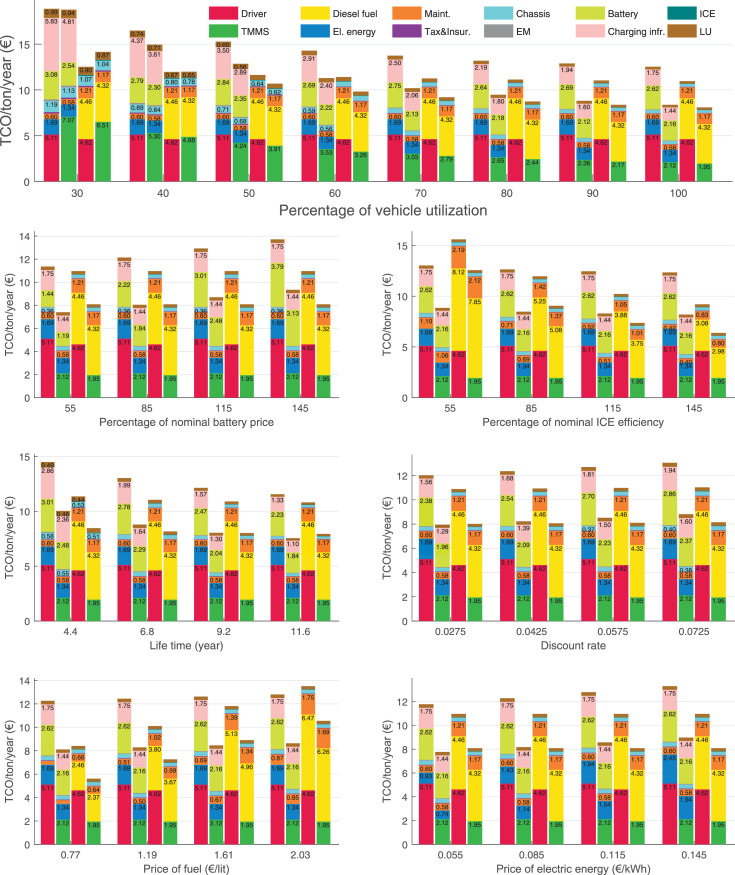
Rigid truck on a very hilly road of 80 km length; sensitivity of the TCO components of different vehicles and driving systems are shown for the optimum speed of the transportation scenario. A group of four bars from left to right represent BEHV HD, BEHV ADS-V, CHV HD and CHV ADS-DV, respectively. See Table 6 for the other vehicle sizes and road types.

**Fig. 188:**
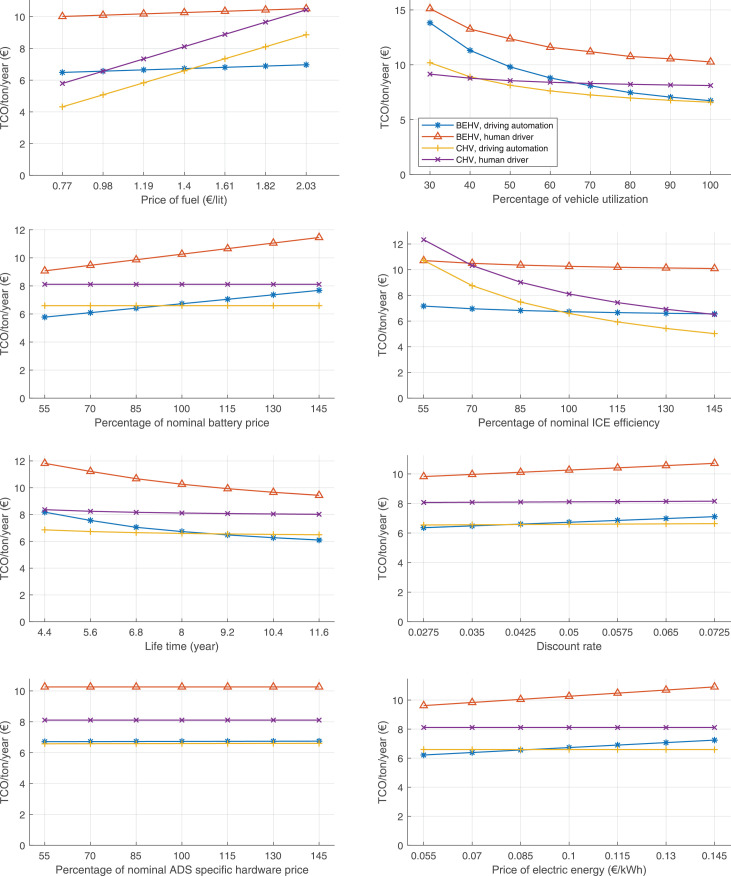
Tractor-semitrailer on a very hilly road of 80 km length; sensitivity of TCO to different parameters are shown for the optimum speed of the transportation scenario. See Table 6 for the other vehicle sizes and road types.

**Fig. 189:**
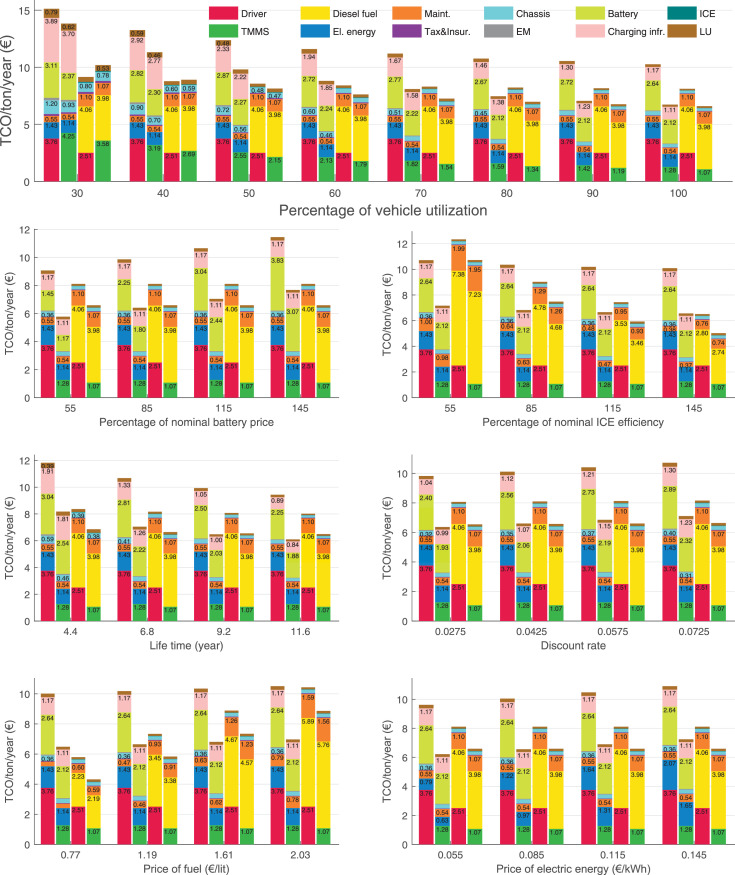
Tractor-semitrailer on a very hilly road of 80 km length; sensitivity of the TCO components of different vehicles and driving systems are shown for the optimum speed of the transportation scenario. A group of four bars from left to right represent BEHV HD, BEHV ADS-V, CHV HD and CHV ADS-DV, respectively. See Table 6 for the other vehicle sizes and road types.

**Fig. 190:**
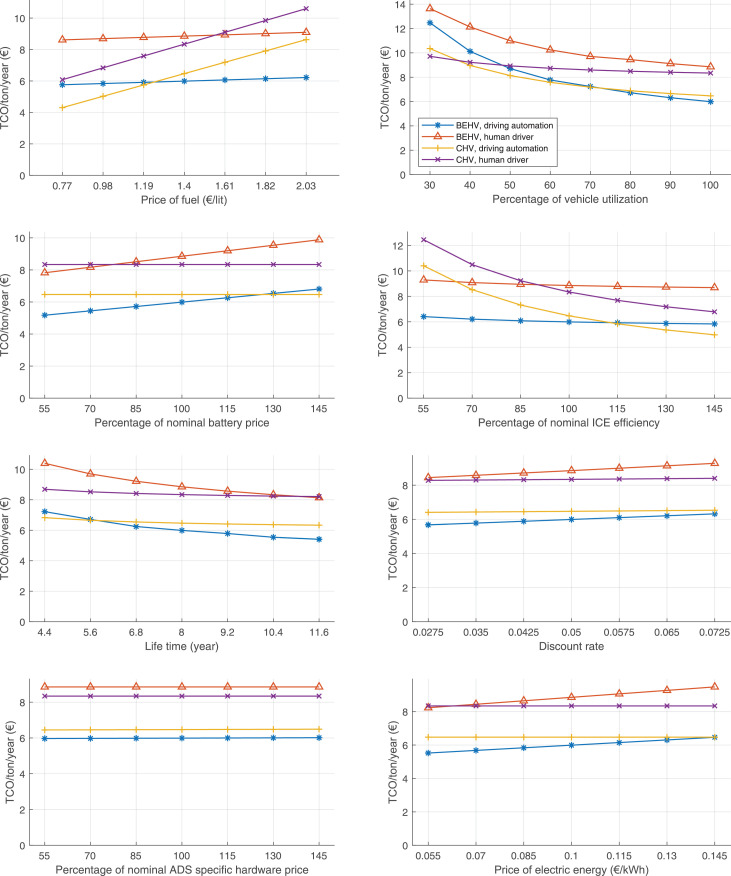
Nordic combination on a very hilly road of 80 km length; sensitivity of TCO to different parameters are shown for the optimum speed of the transportation scenario. See Table 6 for the other vehicle sizes and road types.

**Fig. 191:**
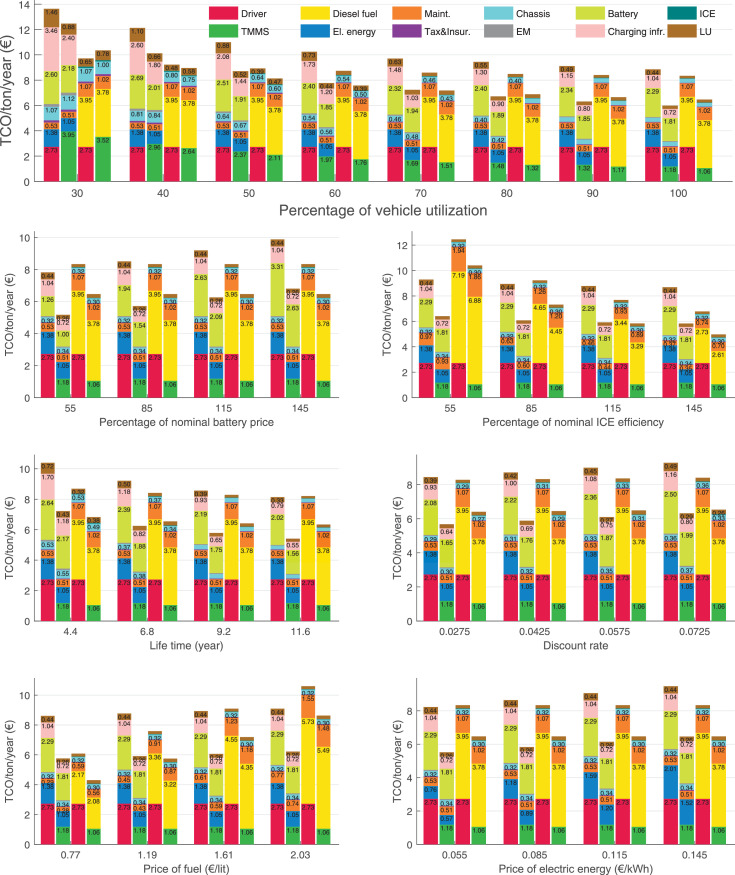
Nordic combination on a very hilly road of 80 km length; sensitivity of the TCO components of different vehicles and driving systems are shown for the optimum speed of the transportation scenario. A group of four bars from left to right represent BEHV HD, BEHV ADS-V, CHV HD and CHV ADS-DV, respectively. See Table 6 for the other vehicle sizes and road types.

**Fig. 192:**
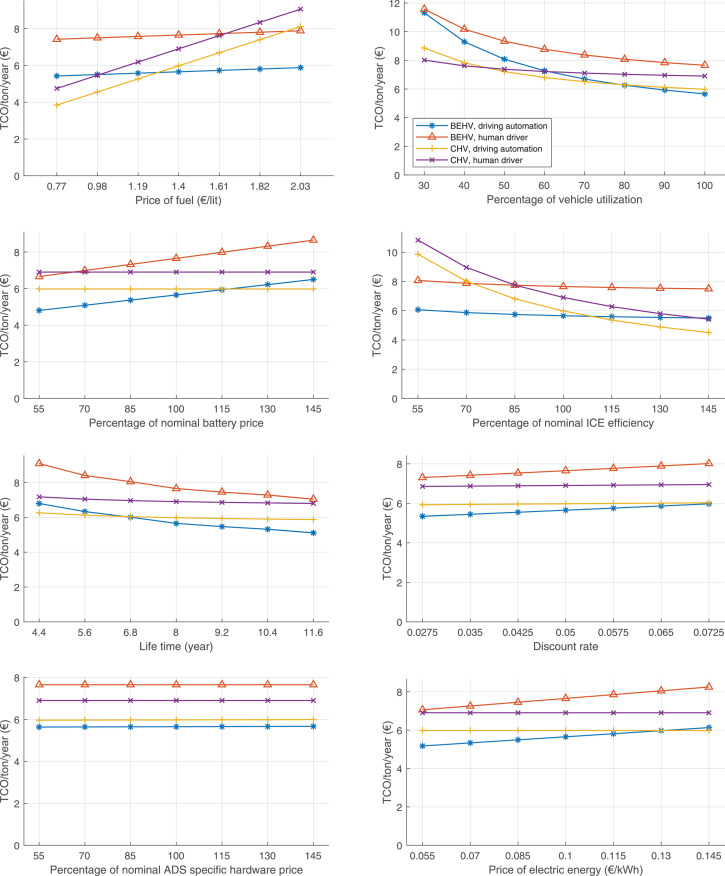
A-double on a very hilly road of 80 km length; sensitivity of TCO to different parameters are shown for the optimum speed of the transportation scenario. See Table 6 for the other vehicle sizes and road types.

**Fig. 193:**
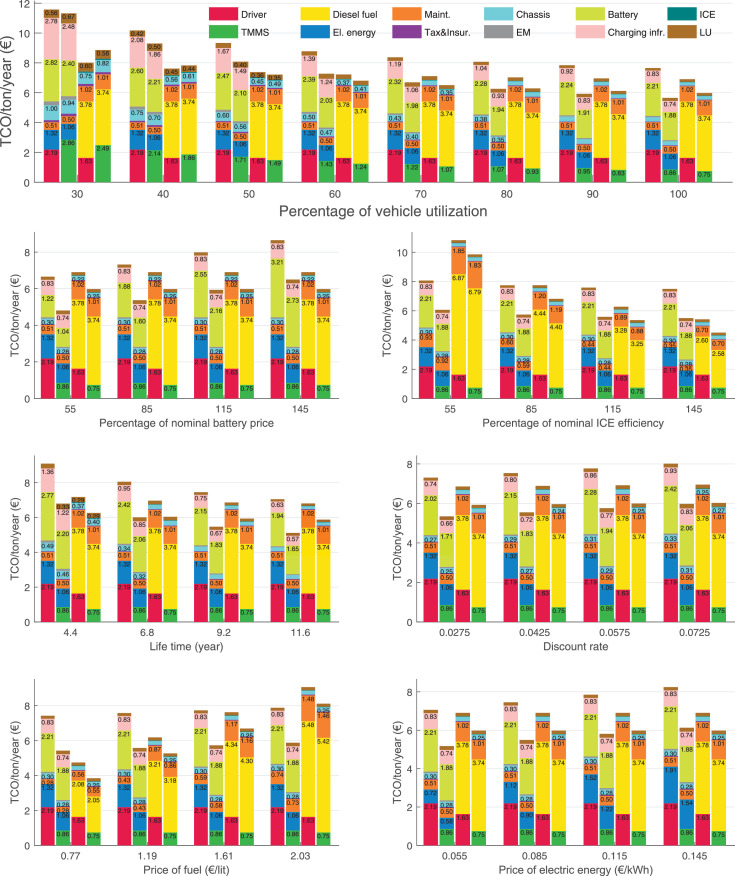
A-double on a very hilly road of 80 km length; sensitivity of the TCO components of different vehicles and driving systems are shown for the optimum speed of the transportation scenario. A group of four bars from left to right represent BEHV HD, BEHV ADS-V, CHV HD and CHV ADS-DV, respectively. See Table 6 for the other vehicle sizes and road types.

**Fig. 194:**
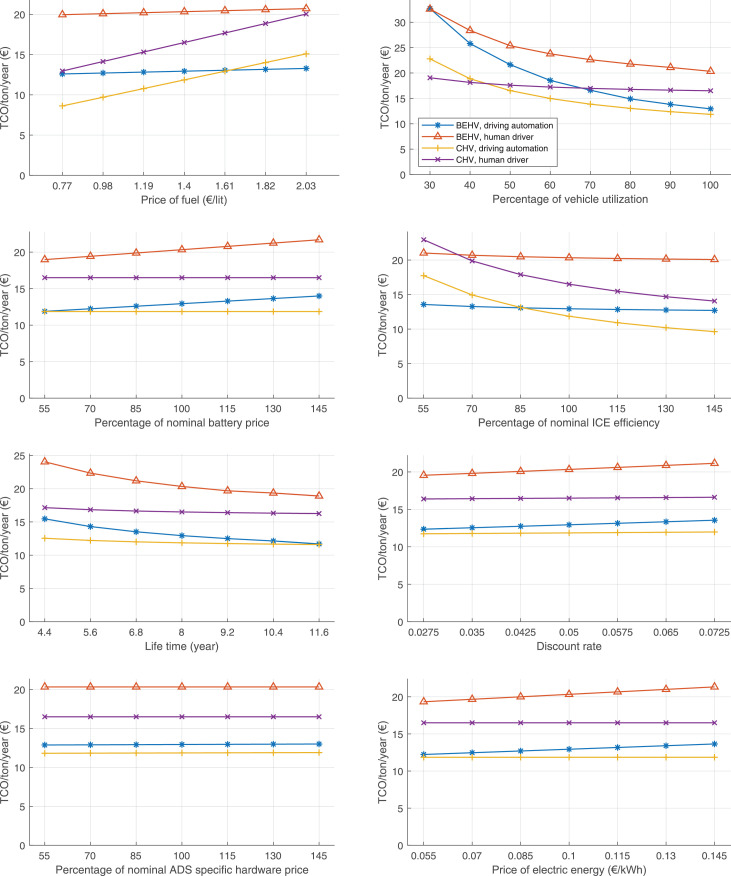
Rigid truck on a flat road of 160 km length; sensitivity of TCO to different parameters are shown for the optimum speed of the transportation scenario. See Table 6 for the other vehicle sizes and road types.

**Fig. 195:**
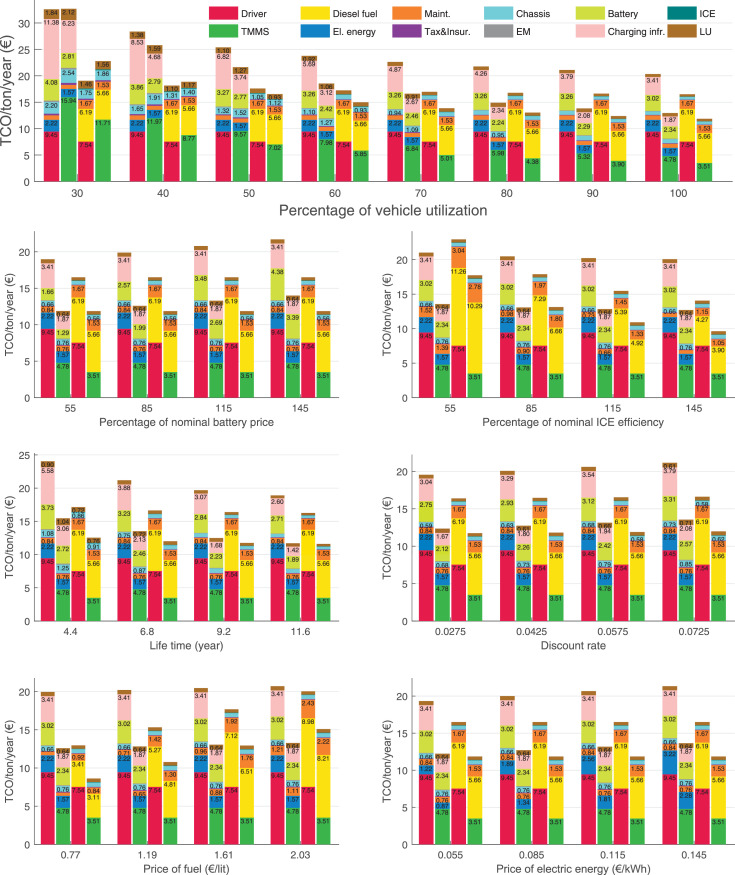
Rigid truck on a flat road of 160 km length; sensitivity of the TCO components of different vehicles and driving systems are shown for the optimum speed of the transportation scenario. A group of four bars from left to right represent BEHV HD, BEHV ADS-V, CHV HD and CHV ADS-DV, respectively. See Table 6 for the other vehicle sizes and road types.

**Fig. 196:**
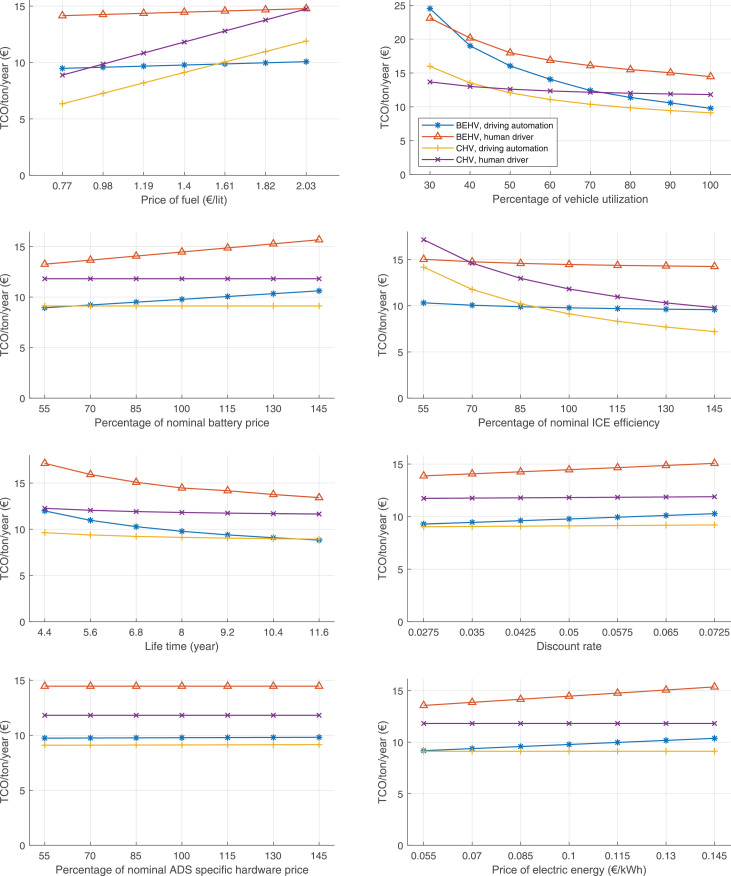
Tractor-semitrailer on a flat road of 160 km length; sensitivity of TCO to different parameters are shown for the optimum speed of the transportation scenario. See Table 6 for the other vehicle sizes and road types.

**Fig. 197:**
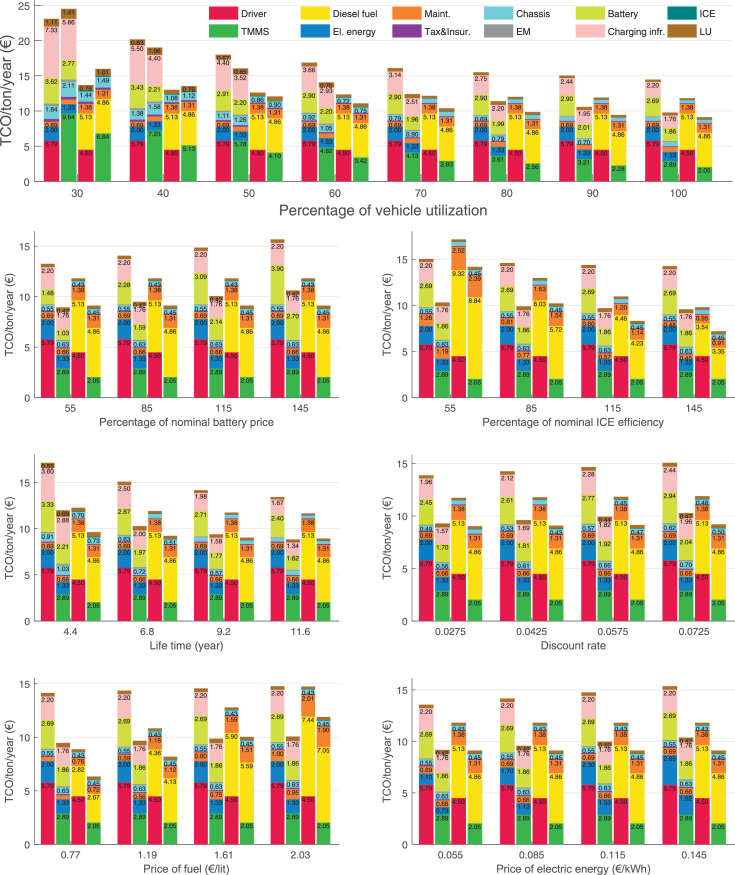
Tractor-semitrailer on a flat road of 160 km length; sensitivity of the TCO components of different vehicles and driving systems are shown for the optimum speed of the transportation scenario. A group of four bars from left to right represent BEHV HD, BEHV ADS-V, CHV HD and CHV ADS-DV, respectively. See Table 6 for the other vehicle sizes and road types.

**Fig. 198:**
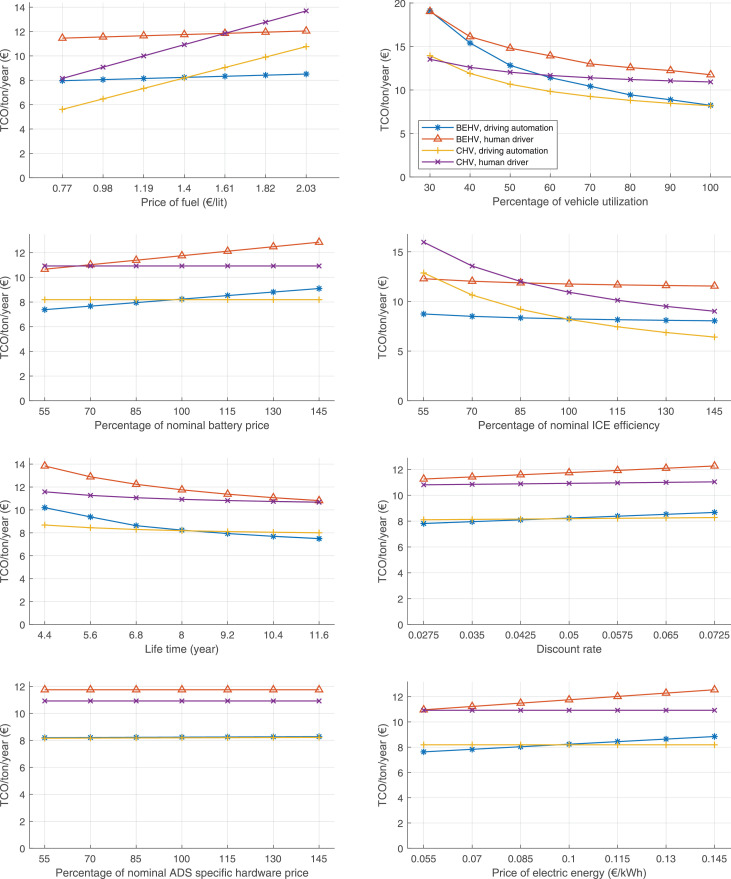
Nordic combination on a flat road of 160 km length; sensitivity of TCO to different parameters are shown for the optimum speed of the transportation scenario. See Table 6 for the other vehicle sizes and road types.

**Fig. 199:**
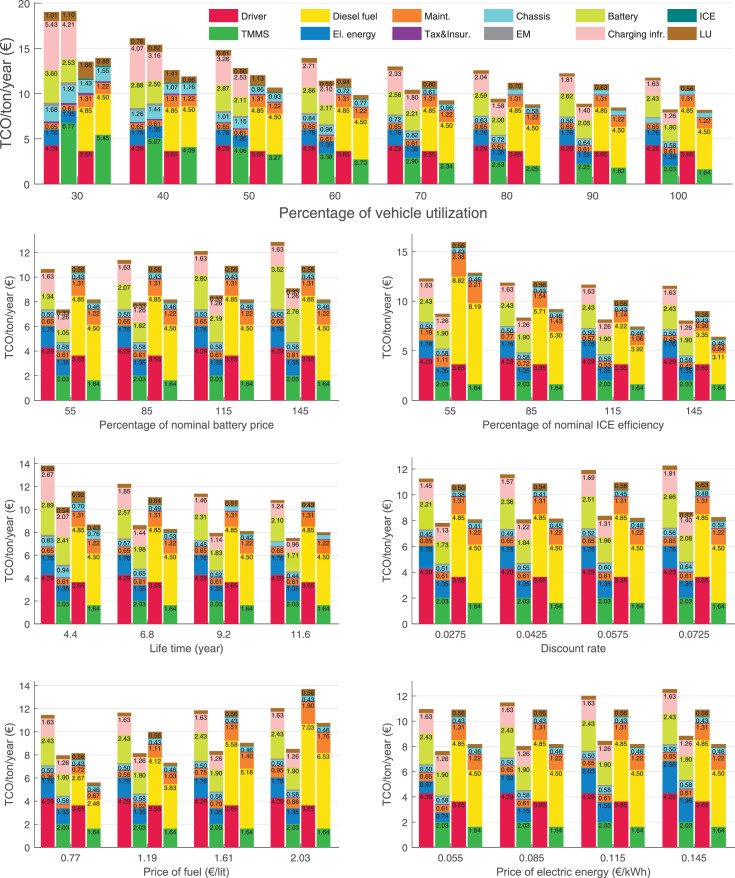
Nordic combination on a flat road of 160 km length; sensitivity of the TCO components of different vehicles and driving systems are shown for the optimum speed of the transportation scenario. A group of four bars from left to right represent BEHV HD, BEHV ADS-V, CHV HD and CHV ADS-DV, respectively. See Table 6 for the other vehicle sizes and road types.

**Fig. 200:**
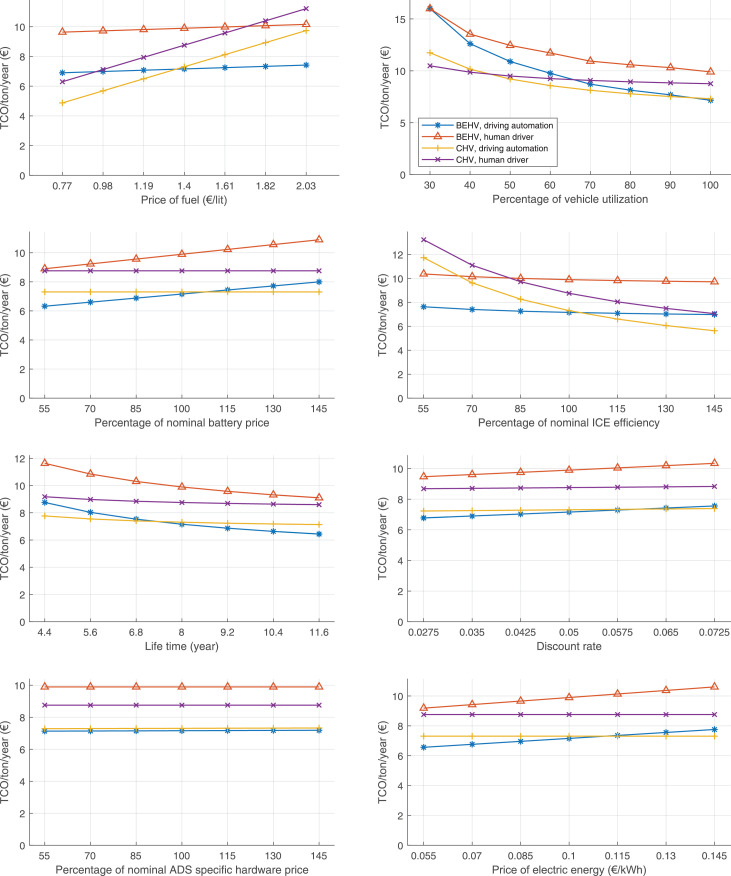
A-double on a flat road of 160 km length; sensitivity of TCO to different parameters are shown for the optimum speed of the transportation scenario. See Table 6 for the other vehicle sizes and road types.

**Fig. 201:**
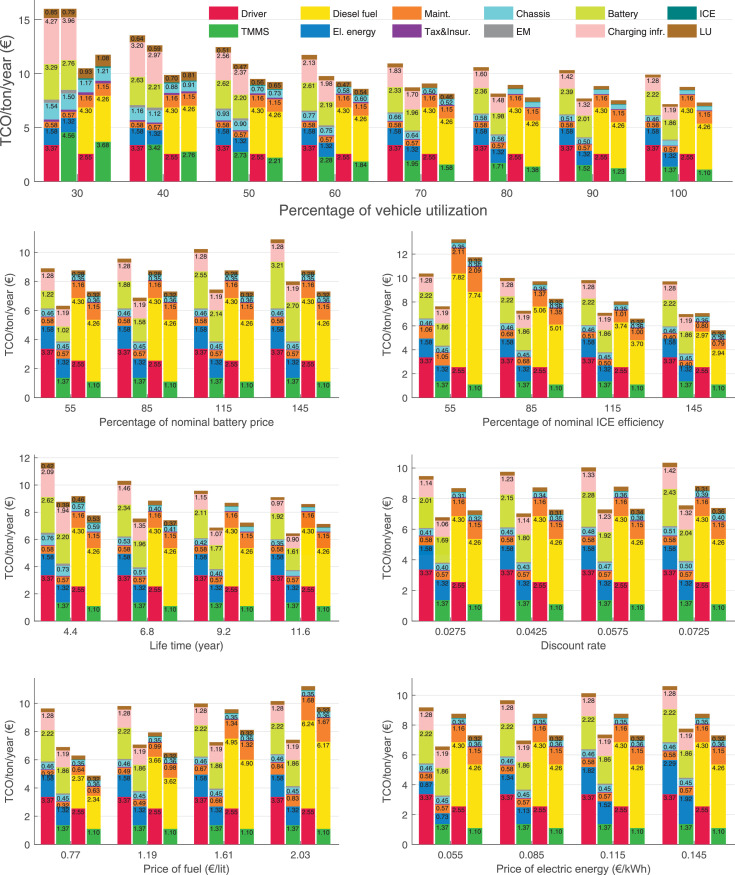
A-double on a flat road of 160 km length; sensitivity of the TCO components of different vehicles and driving systems are shown for the optimum speed of the transportation scenario. A group of four bars from left to right represent BEHV HD, BEHV ADS-V, CHV HD and CHV ADS-DV, respectively. See Table 6 for the other vehicle sizes and road types.

**Fig. 202:**
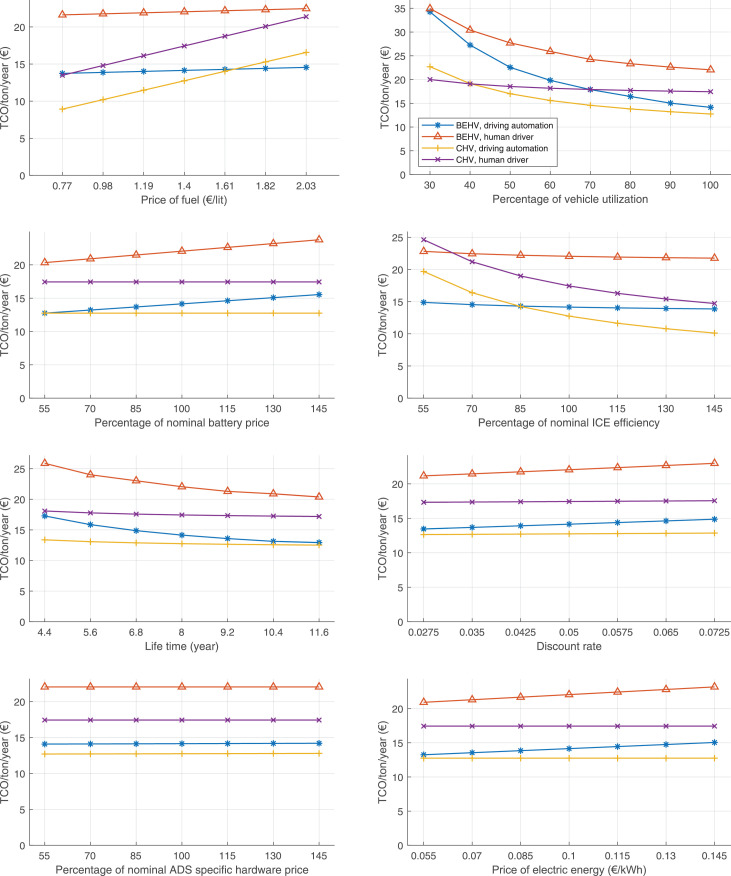
Rigid truck on a predominantly flat road of 160 km length; sensitivity of TCO to different parameters are shown for the optimum speed of the transportation scenario. See Table 6 for the other vehicle sizes and road types.

**Fig. 203:**
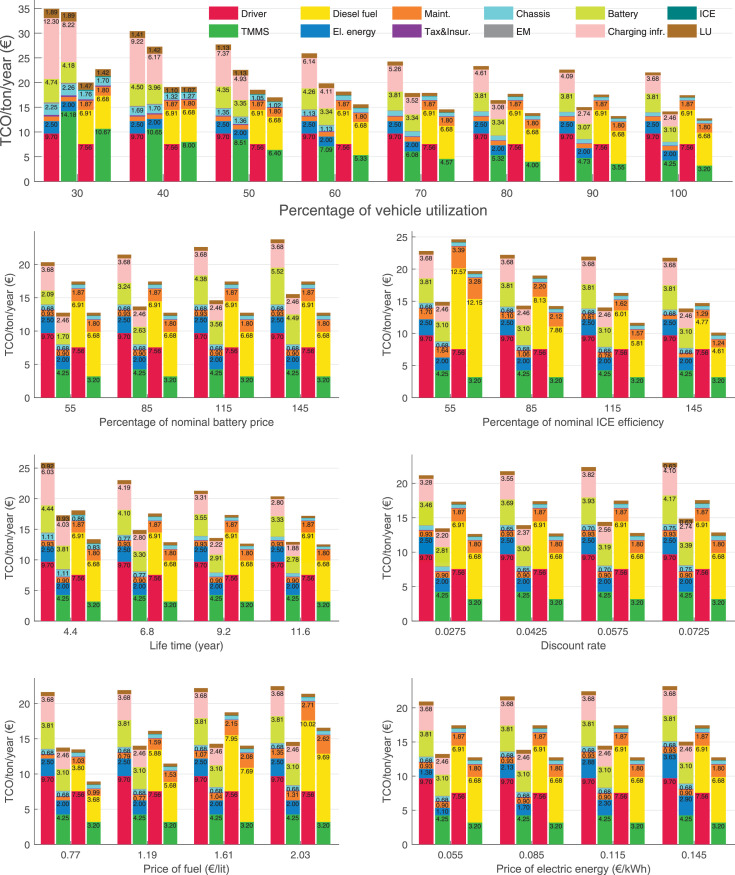
Rigid truck on a predominantly flat road of 160 km length; sensitivity of the TCO components of different vehicles and driving systems are shown for the optimum speed of the transportation scenario. A group of four bars from left to right represent BEHV HD, BEHV ADS-V, CHV HD and CHV ADS-DV, respectively. See Table 6 for the other vehicle sizes and road types.

**Fig. 204:**
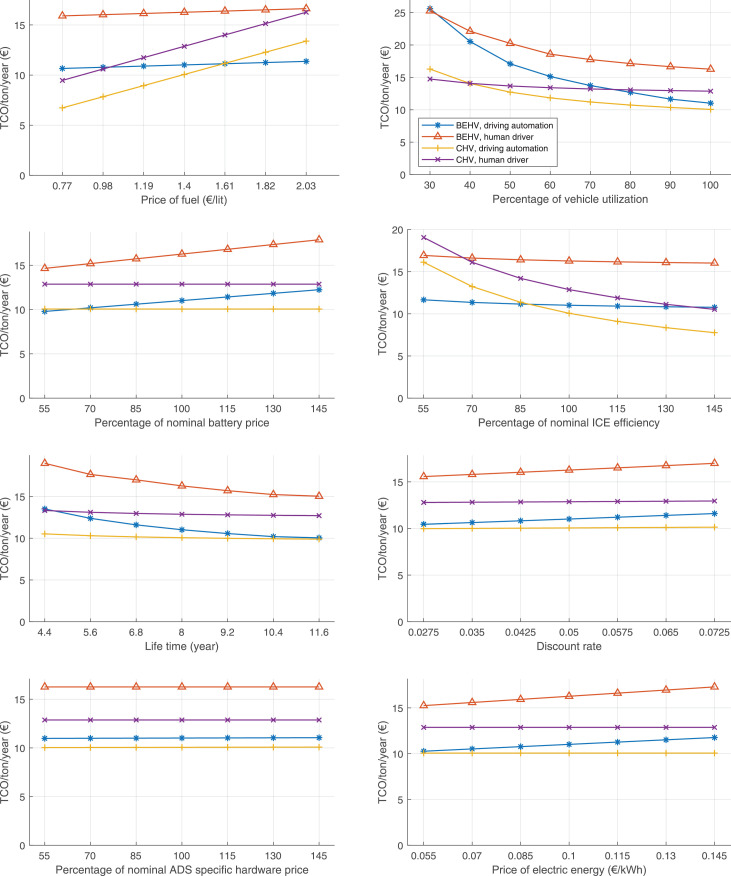
Tractor-semitrailer on a predominantly flat road of 160 km length; sensitivity of TCO to different parameters are shown for the optimum speed of the transportation scenario. See Table 6 for the other vehicle sizes and road types.

**Fig. 205:**
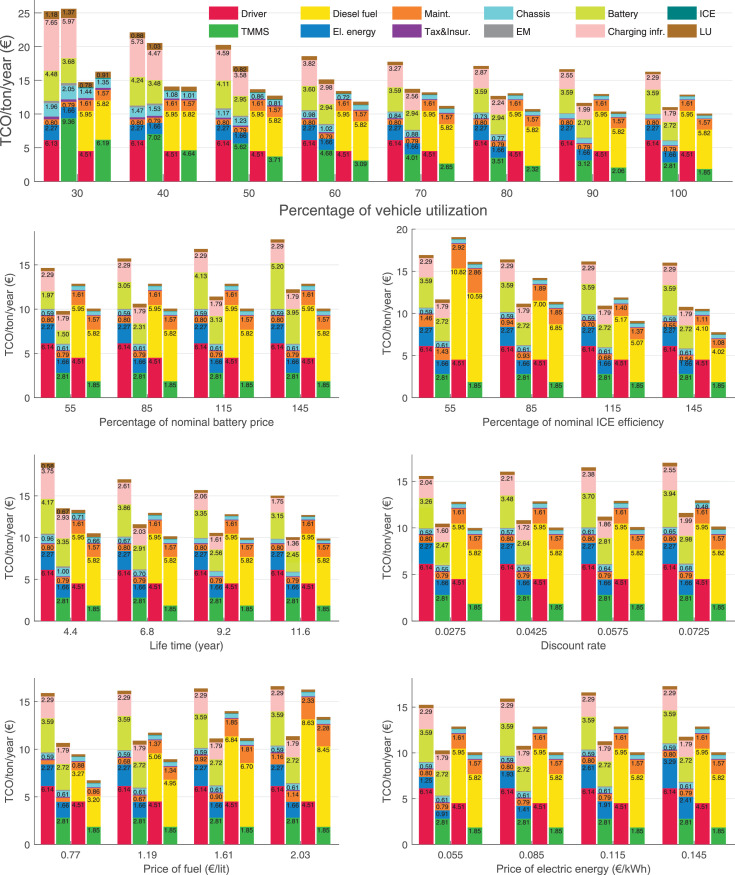
Tractor-semitrailer on a predominantly flat road of 160 km length; sensitivity of the TCO components of different vehicles and driving systems are shown for the optimum speed of the transportation scenario. A group of four bars from left to right represent BEHV HD, BEHV ADS-V, CHV HD and CHV ADS-DV, respectively. See Table 6 for the other vehicle sizes and road types.

**Fig. 206:**
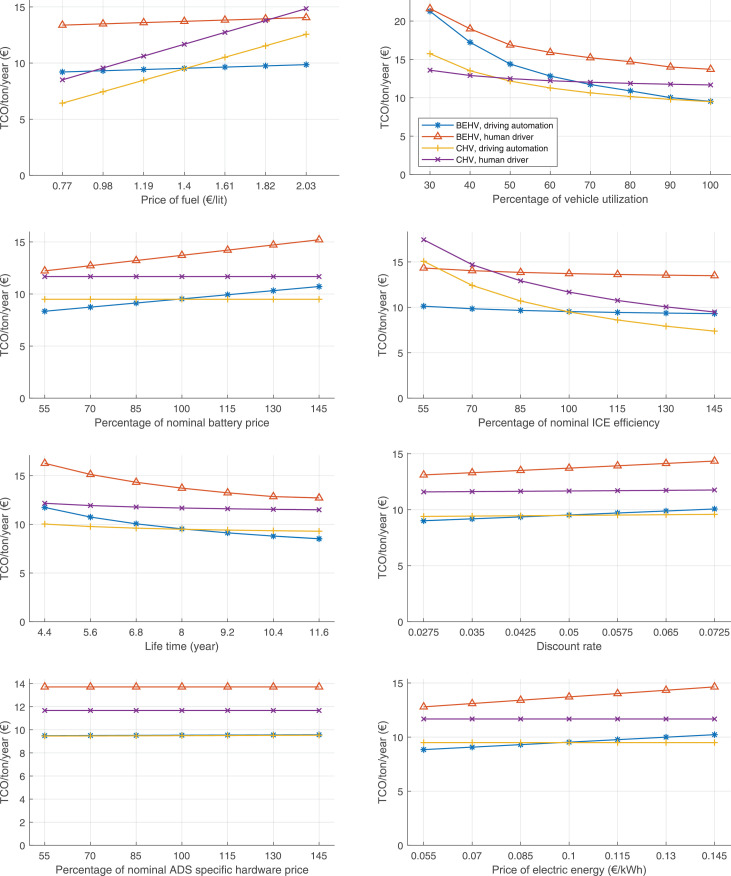
Nordic combination on a predominantly flat road of 160 km length; sensitivity of TCO to different parameters are shown for the optimum speed of the transportation scenario. See Table 6 for the other vehicle sizes and road types.

**Fig. 207:**
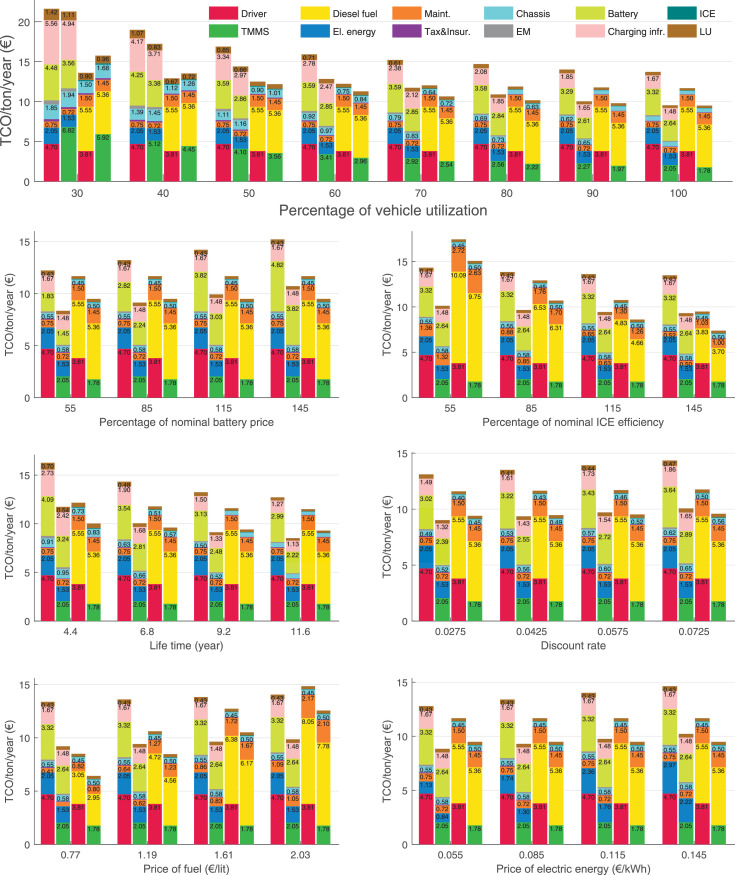
Nordic combination on a predominantly flat road of 160 km length; sensitivity of the TCO components of different vehicles and driving systems are shown for the optimum speed of the transportation scenario. A group of four bars from left to right represent BEHV HD, BEHV ADS-V, CHV HD and CHV ADS-DV, respectively. See Table 6 for the other vehicle sizes and road types.

**Fig. 208:**
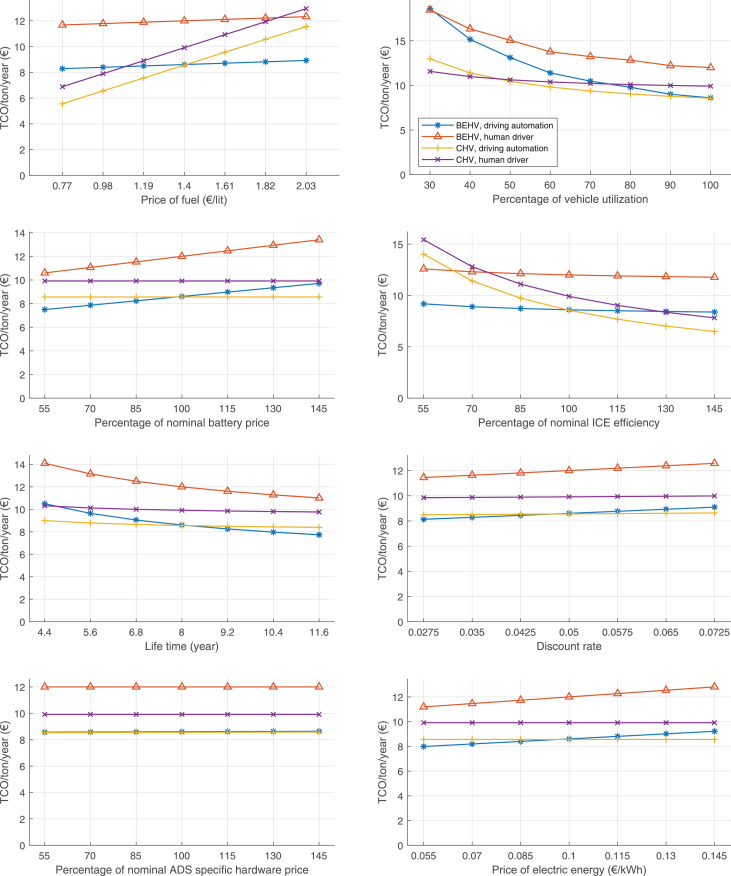
A-double on a predominantly flat road of 160 km length; sensitivity of TCO to different parameters are shown for the optimum speed of the transportation scenario. See Table 6 for the other vehicle sizes and road types.

**Fig. 209:**
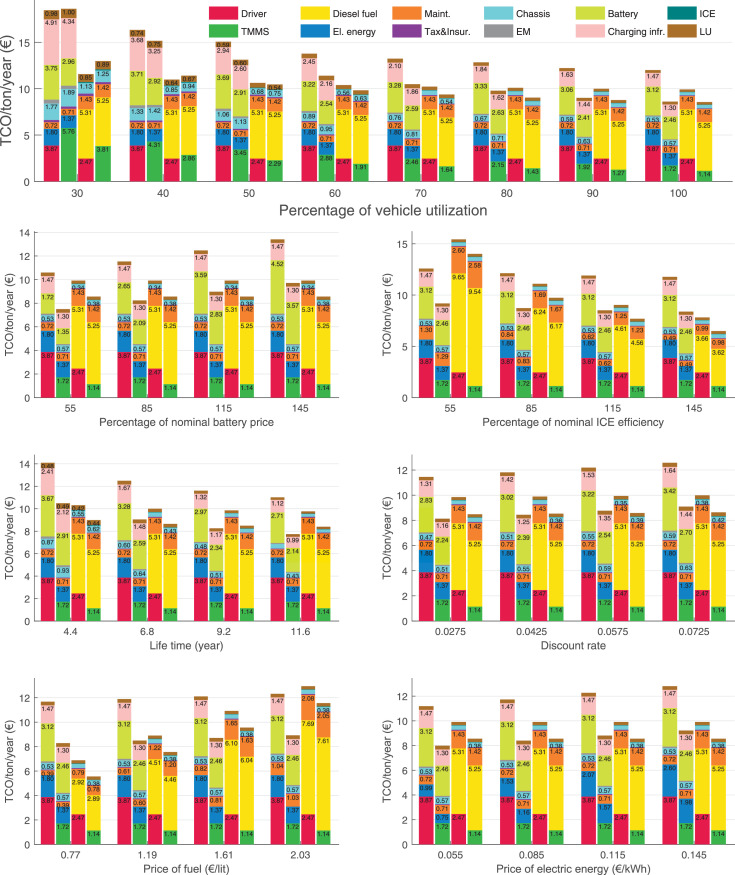
A-double on a predominantly flat road of 160 km length; sensitivity of the TCO components of different vehicles and driving systems are shown for the optimum speed of the transportation scenario. A group of four bars from left to right represent BEHV HD, BEHV ADS-V, CHV HD and CHV ADS-DV, respectively. See Table 6 for the other vehicle sizes and road types.

**Fig. 210:**
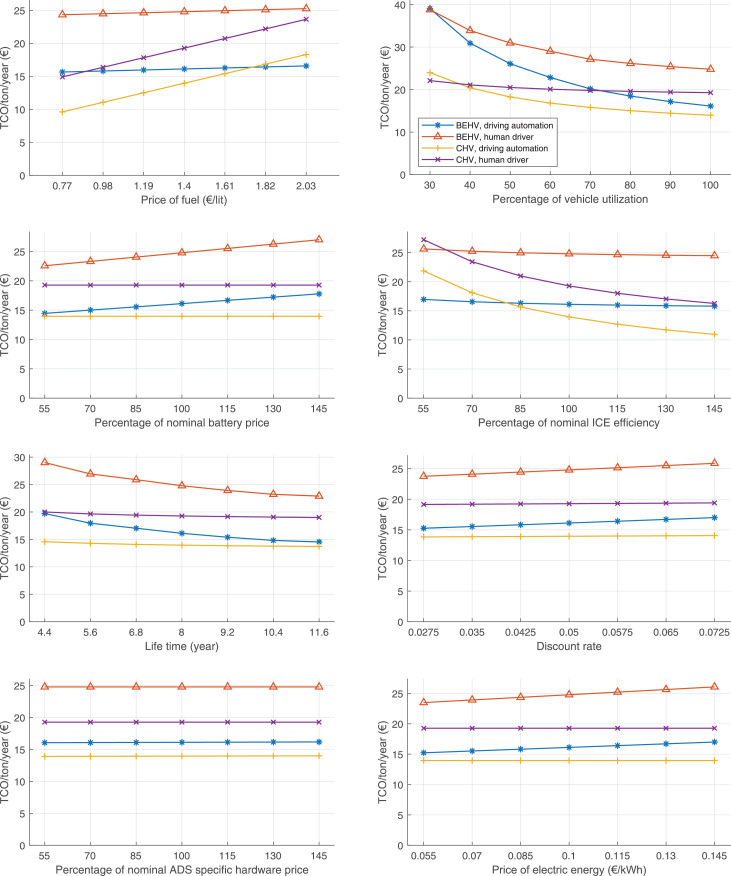
Rigid truck on a hilly road of 160 km length; sensitivity of TCO to different parameters are shown for the optimum speed of the transportation scenario. See Table 6 for the other vehicle sizes and road types.

**Fig. 211:**
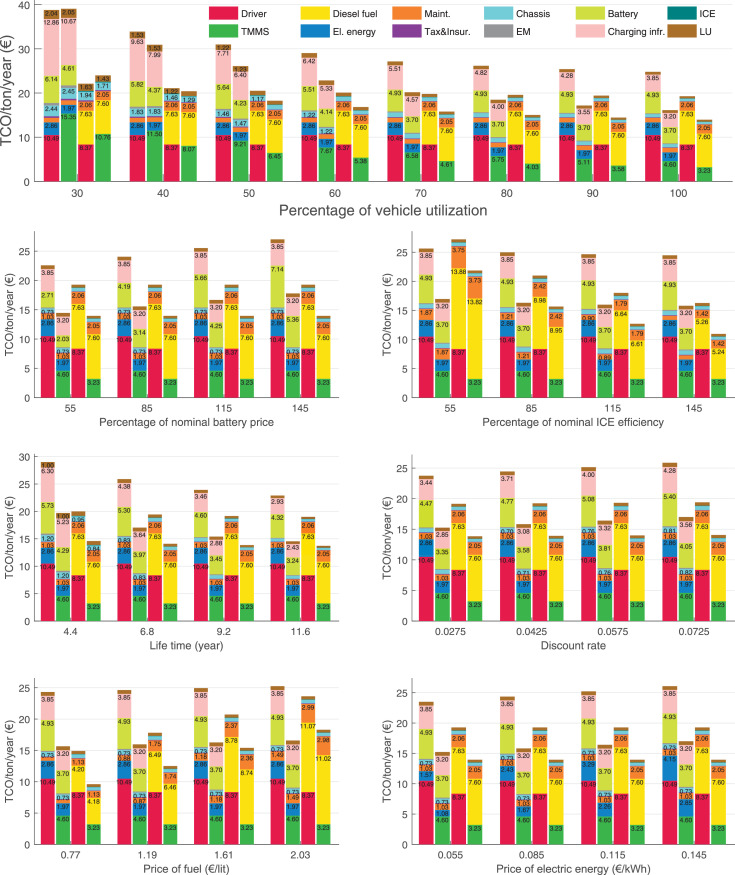
Rigid truck on a hilly road of 160 km length; sensitivity of the TCO components of different vehicles and driving systems are shown for the optimum speed of the transportation scenario. A group of four bars from left to right represent BEHV HD, BEHV ADS-V, CHV HD and CHV ADS-DV, respectively. See Table 6 for the other vehicle sizes and road types.

**Fig. 212:**
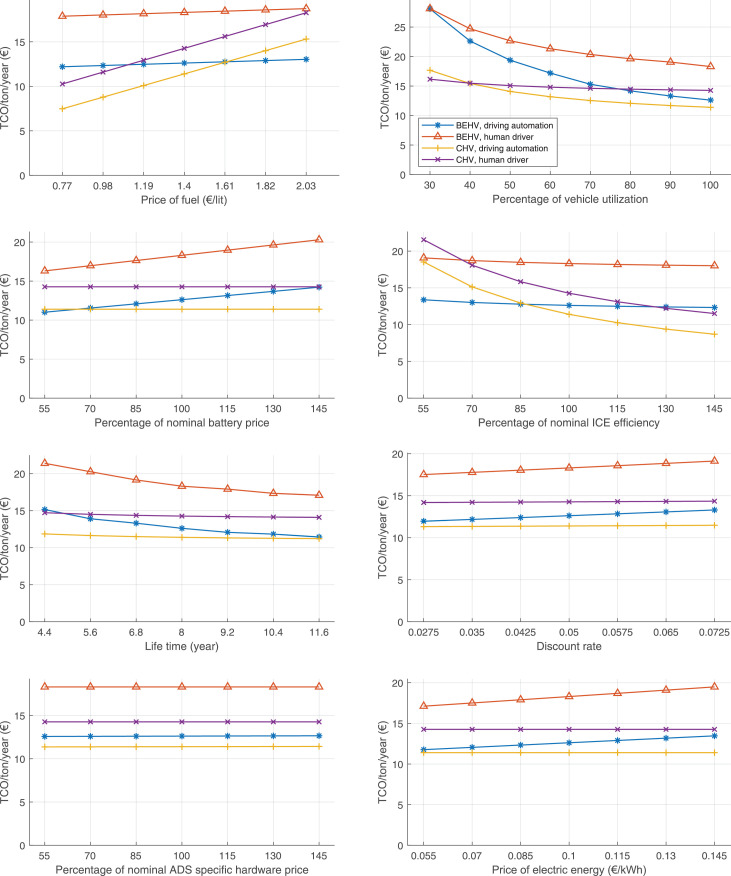
Tractor-semitrailer on a hilly road of 160 km length; sensitivity of TCO to different parameters are shown for the optimum speed of the transportation scenario. See Table 6 for the other vehicle sizes and road types.

**Fig. 213:**
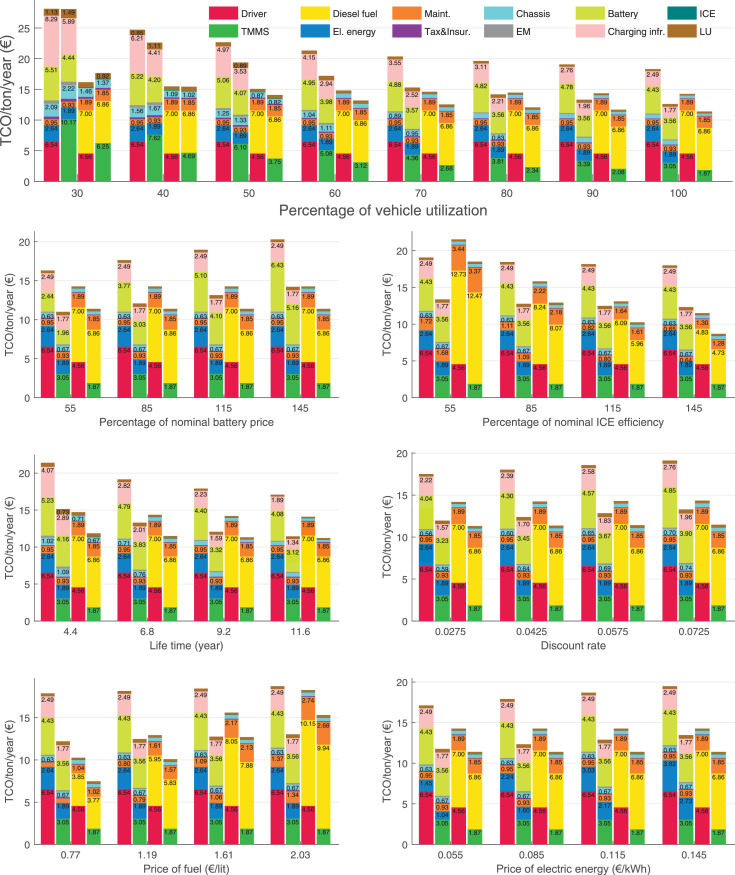
Tractor-semitrailer on a hilly road of 160 km length; sensitivity of the TCO components of different vehicles and driving systems are shown for the optimum speed of the transportation scenario. A group of four bars from left to right represent BEHV HD, BEHV ADS-V, CHV HD and CHV ADS-DV, respectively. See Table 6 for the other vehicle sizes and road types.

**Fig. 214:**
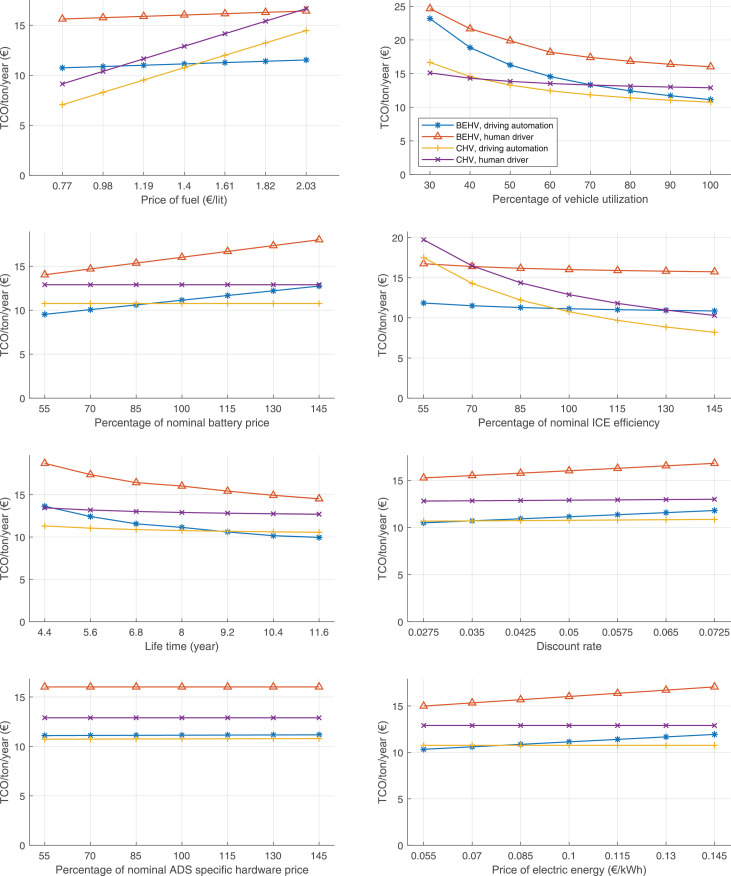
Nordic combination on a hilly road of 160 km length; sensitivity of TCO to different parameters are shown for the optimum speed of the transportation scenario. See Table 6 for the other vehicle sizes and road types.

**Fig. 215:**
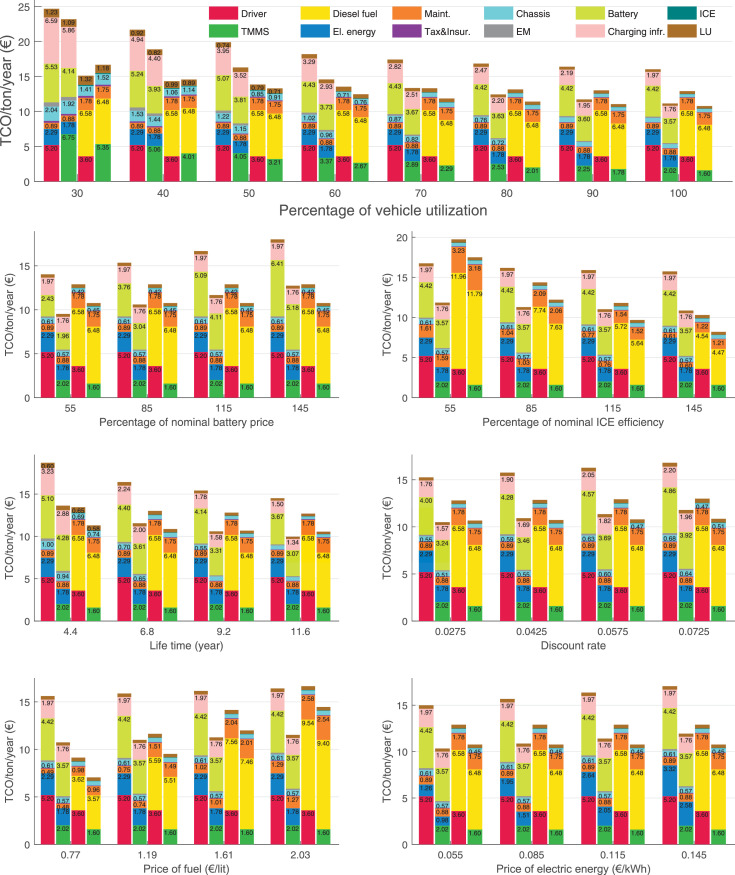
Nordic combination on a hilly road of 160 km length; sensitivity of the TCO components of different vehicles and driving systems are shown for the optimum speed of the transportation scenario. A group of four bars from left to right represent BEHV HD, BEHV ADS-V, CHV HD and CHV ADS-DV, respectively. See Table 6 for the other vehicle sizes and road types.

**Fig. 216:**
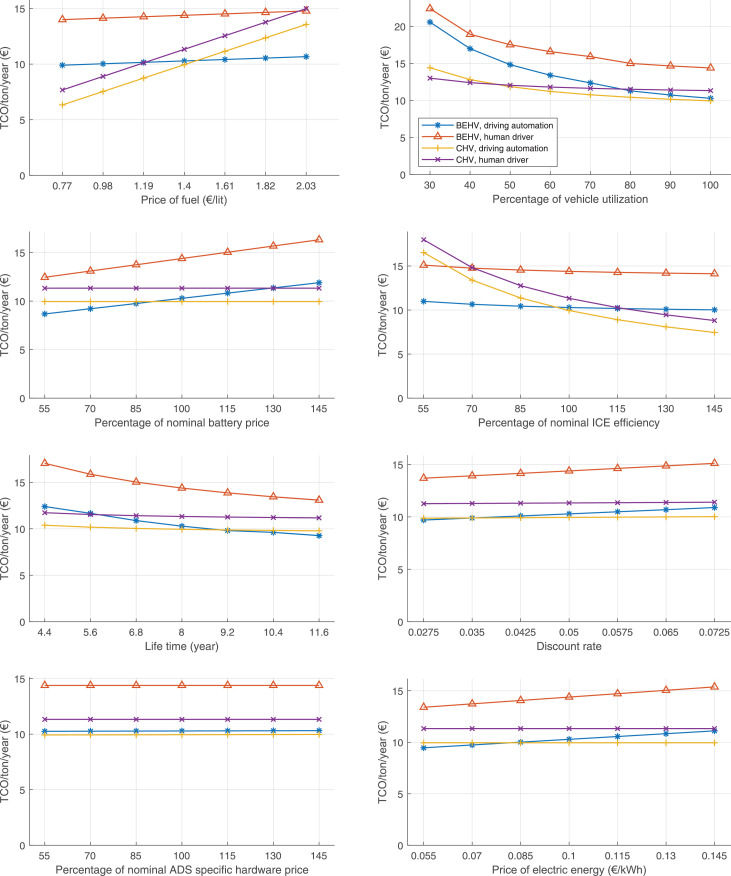
A-double on a hilly road of 160 km length; sensitivity of TCO to different parameters are shown for the optimum speed of the transportation scenario. See Table 6 for the other vehicle sizes and road types.

**Fig. 217:**
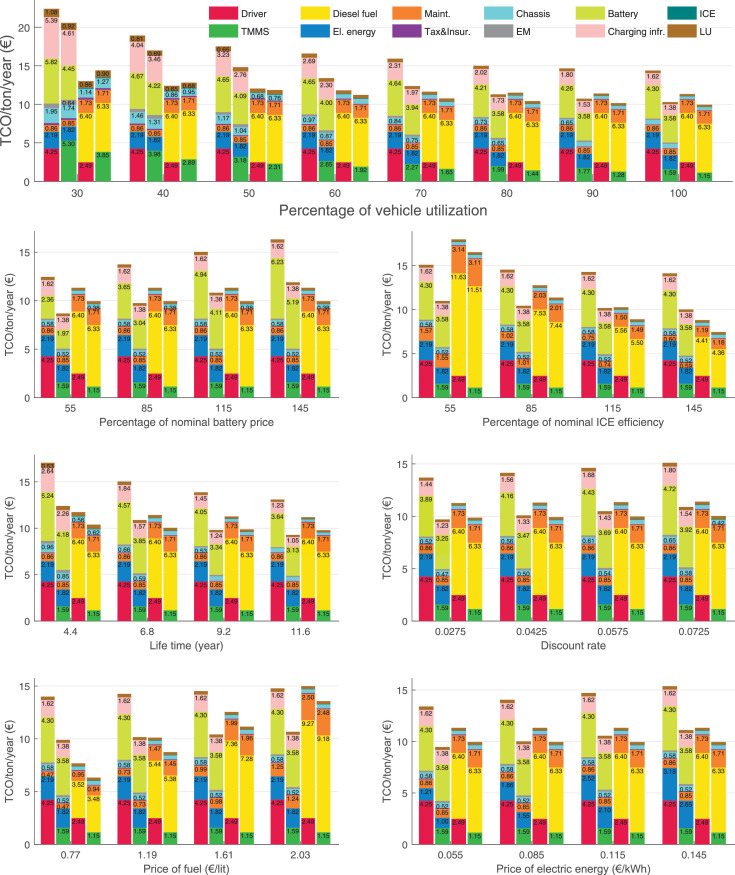
A-double on a hilly road of 160 km length; sensitivity of the TCO components of different vehicles and driving systems are shown for the optimum speed of the transportation scenario. A group of four bars from left to right represent BEHV HD, BEHV ADS-V, CHV HD and CHV ADS-DV, respectively. See Table 6 for the other vehicle sizes and road types.

**Fig. 218:**
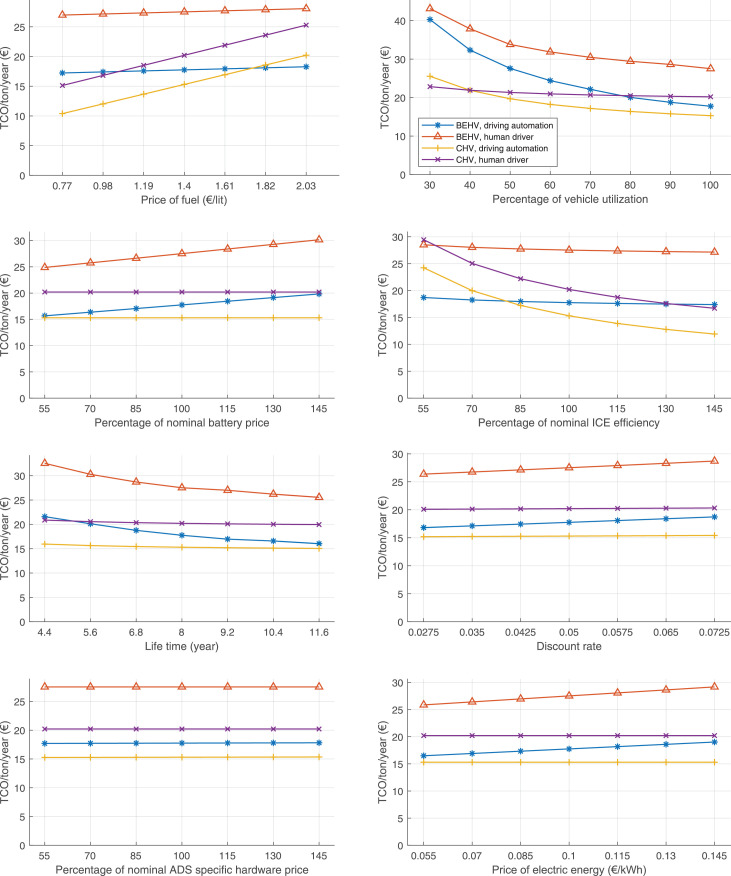
Rigid truck on a very hilly road of 160 km length; sensitivity of TCO to different parameters are shown for the optimum speed of the transportation scenario. See Table 6 for the other vehicle sizes and road types.

**Fig. 219:**
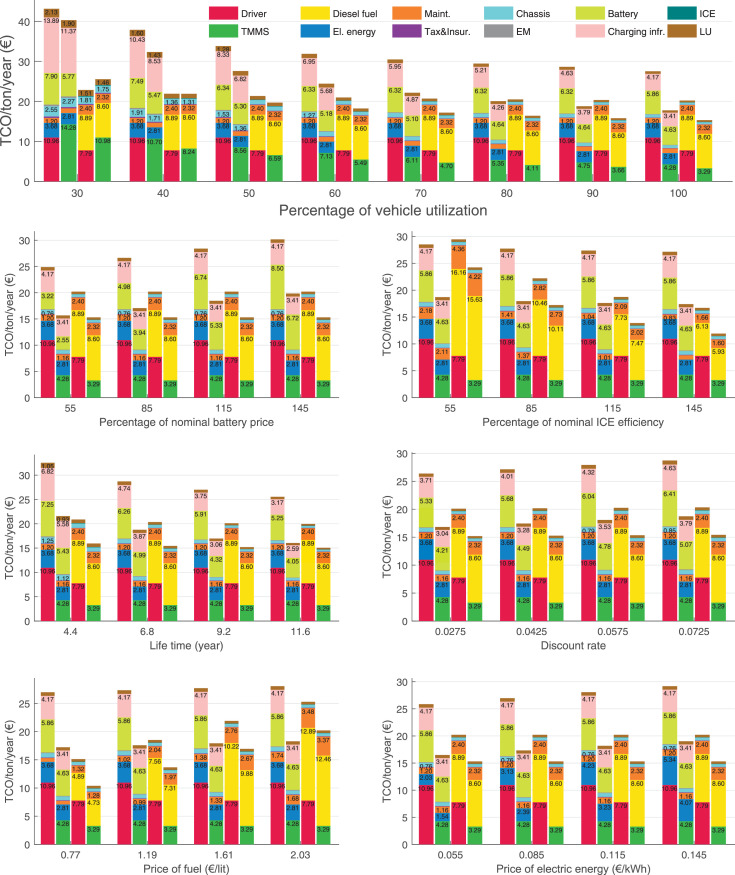
Rigid truck on a very hilly road of 160 km length; sensitivity of the TCO components of different vehicles and driving systems are shown for the optimum speed of the transportation scenario. A group of four bars from left to right represent BEHV HD, BEHV ADS-V, CHV HD and CHV ADS-DV, respectively. See Table 6 for the other vehicle sizes and road types.

**Fig. 220:**
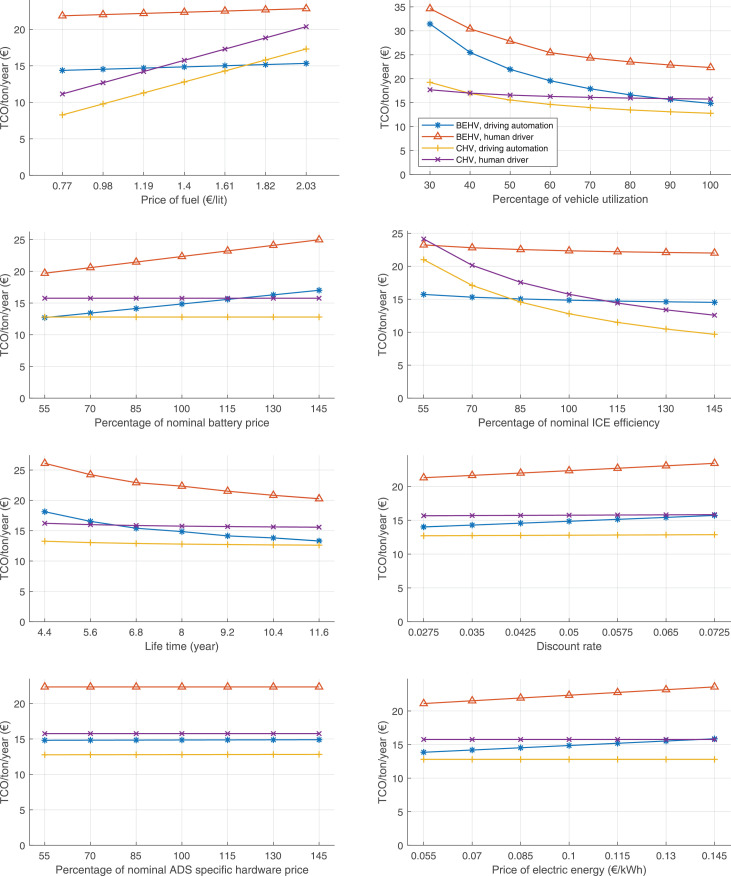
Tractor-semitrailer on a very hilly road of 160 km length; sensitivity of TCO to different parameters are shown for the optimum speed of the transportation scenario. See Table 6 for the other vehicle sizes and road types.

**Fig. 221:**
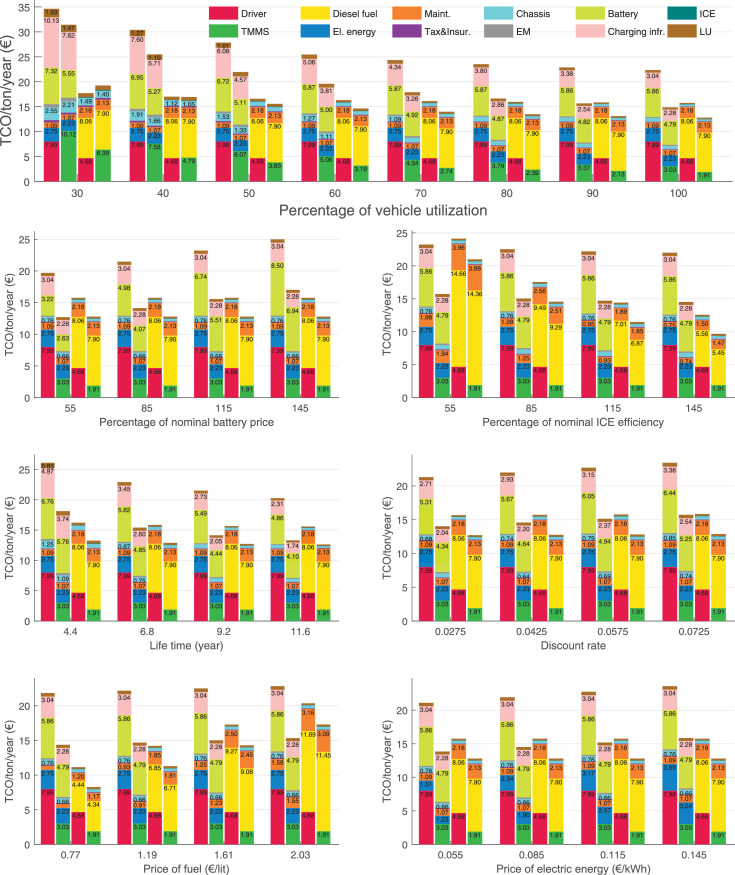
Tractor-semitrailer on a very hilly road of 160 km length; sensitivity of the TCO components of different vehicles and driving systems are shown for the optimum speed of the transportation scenario. A group of four bars from left to right represent BEHV HD, BEHV ADS-V, CHV HD and CHV ADS-DV, respectively. See Table 6 for the other vehicle sizes and road types.

**Fig. 222:**
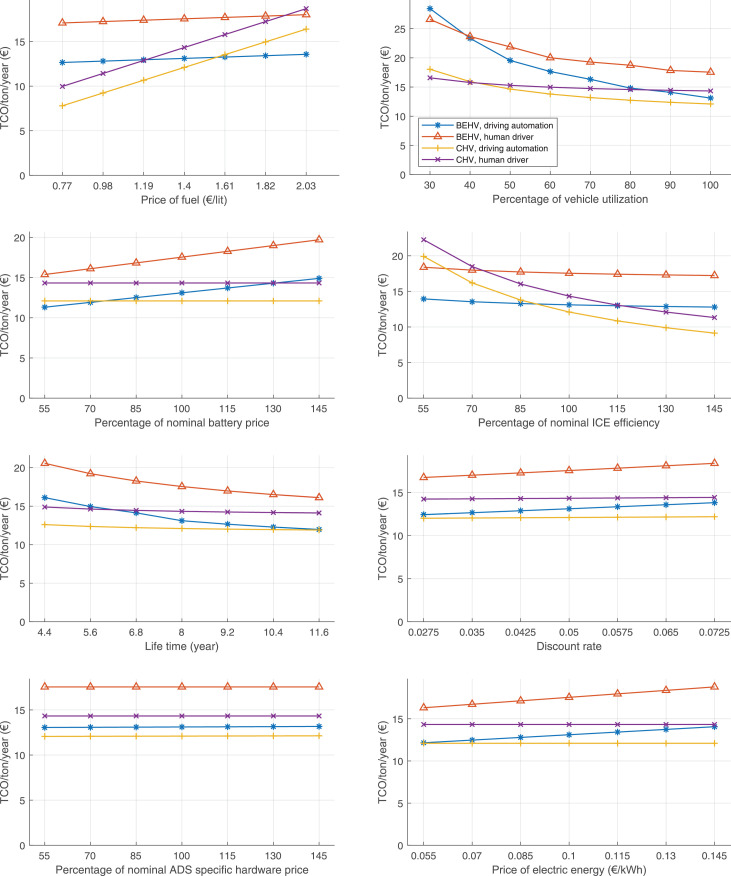
Nordic combination on a very hilly road of 160 km length; sensitivity of TCO to different parameters are shown for the optimum speed of the transportation scenario. See Table 6 for the other vehicle sizes and road types.

**Fig. 223:**
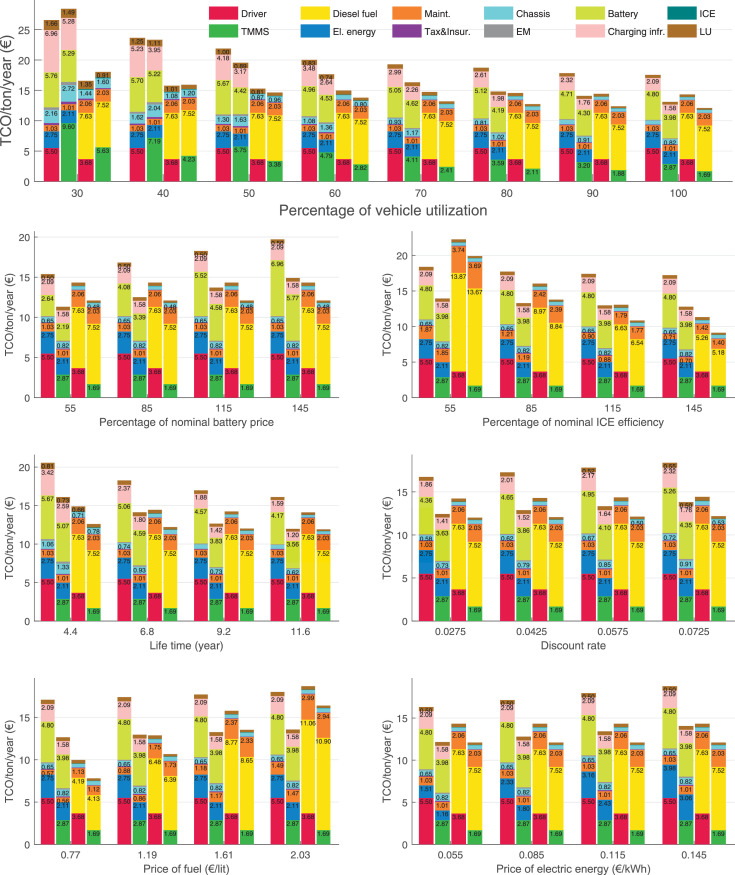
Nordic combination on a very hilly road of 160 km length; sensitivity of the TCO components of different vehicles and driving systems are shown for the optimum speed of the transportation scenario. A group of four bars from left to right represent BEHV HD, BEHV ADS-V, CHV HD and CHV ADS-DV, respectively. See Table 6 for the other vehicle sizes and road types.

**Fig. 224:**
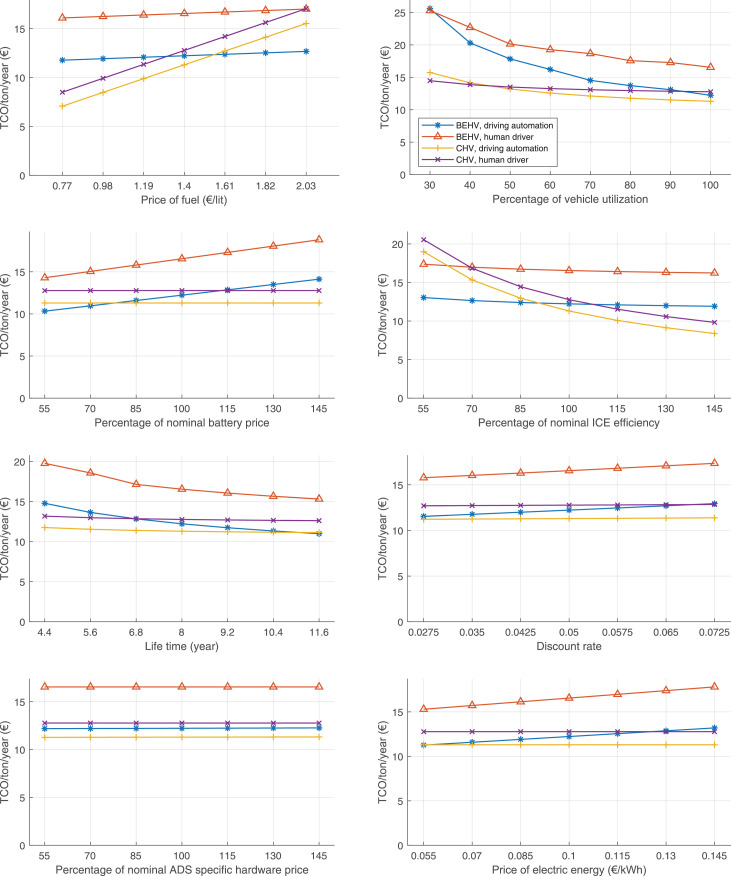
A-double on a very hilly road of 160 km length; sensitivity of TCO to different parameters are shown for the optimum speed of the transportation scenario. See Table 6 for the other vehicle sizes and road types.

**Fig. 225:**
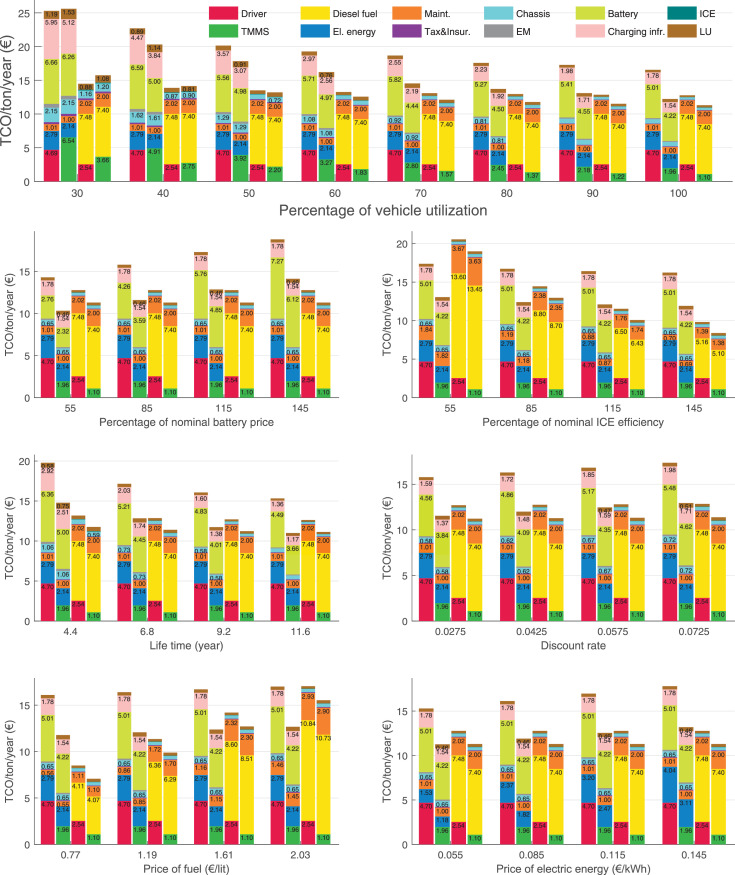
A-double on a very hilly road of 160 km length; sensitivity of the TCO components of different vehicles and driving systems are shown for the optimum speed of the transportation scenario. A group of four bars from left to right represent BEHV HD, BEHV ADS-V, CHV HD and CHV ADS-DV, respectively. See Table 6 for the other vehicle sizes and road types.

**Fig. 226:**
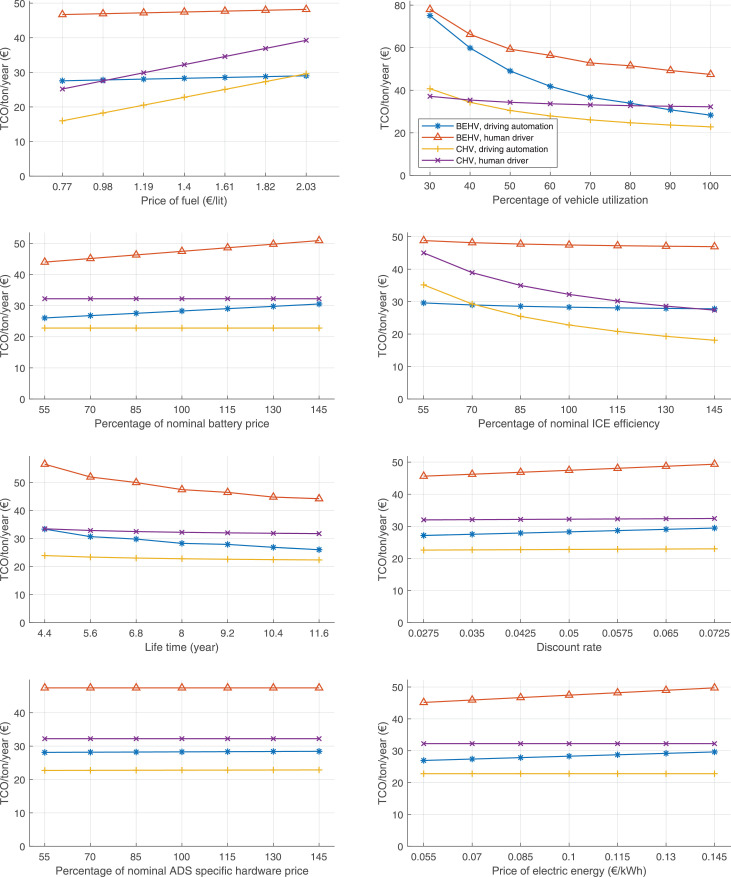
Rigid truck on a flat road of 320 km length; sensitivity of TCO to different parameters are shown for the optimum speed of the transportation scenario. See Table 6 for the other vehicle sizes and road types.

**Fig. 227:**
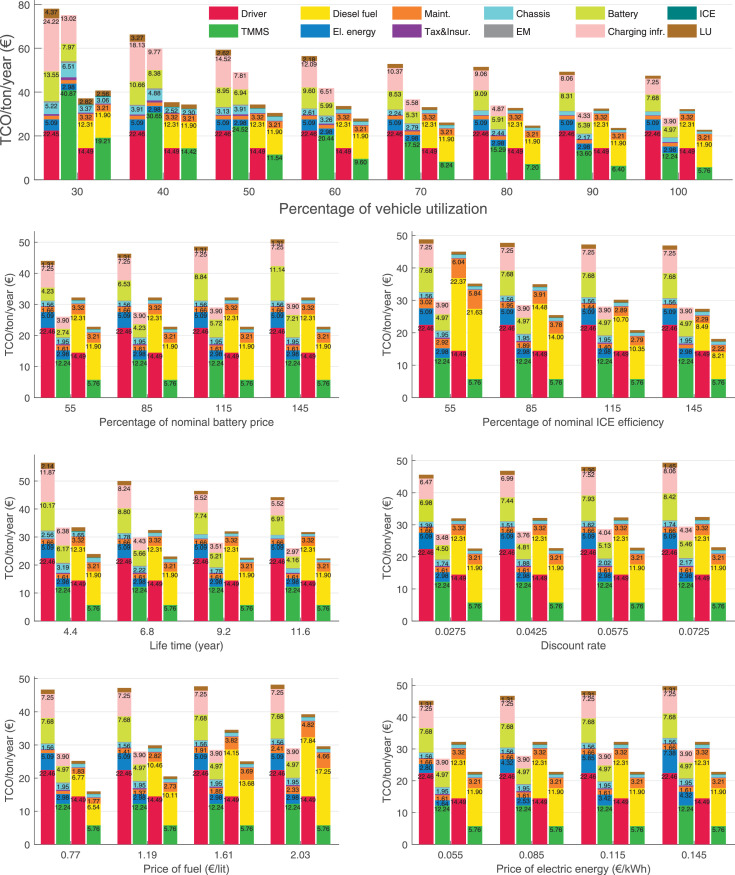
Rigid truck on a flat road of 320 km length; sensitivity of the TCO components of different vehicles and driving systems are shown for the optimum speed of the transportation scenario. A group of four bars from left to right represent BEHV HD, BEHV ADS-V, CHV HD and CHV ADS-DV, respectively. See Table 6 for the other vehicle sizes and road types.

**Fig. 228:**
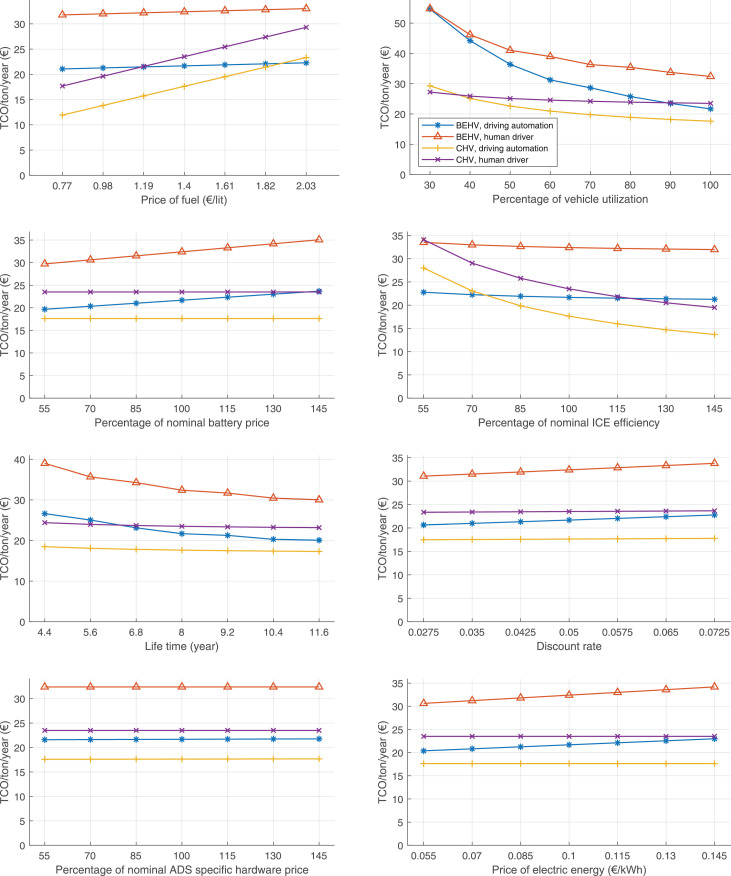
Tractor-semitrailer on a flat road of 320 km length; sensitivity of TCO to different parameters are shown for the optimum speed of the transportation scenario. See Table 6 for the other vehicle sizes and road types.

**Fig. 229:**
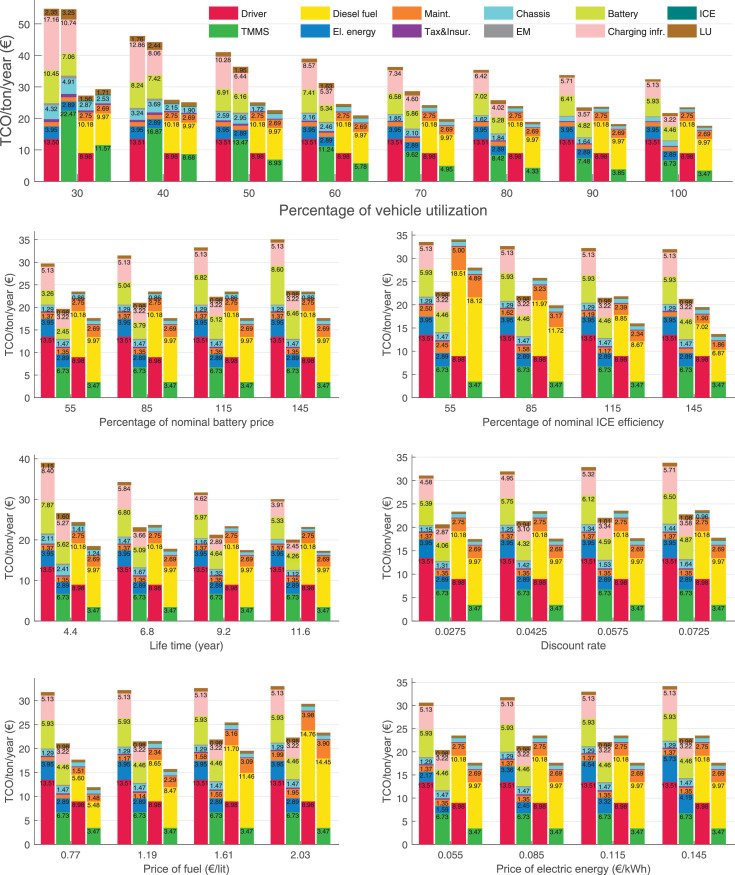
Tractor-semitrailer on a flat road of 320 km length; sensitivity of the TCO components of different vehicles and driving systems are shown for the optimum speed of the transportation scenario. A group of four bars from left to right represent BEHV HD, BEHV ADS-V, CHV HD and CHV ADS-DV, respectively. See Table 6 for the other vehicle sizes and road types.

**Fig. 230:**
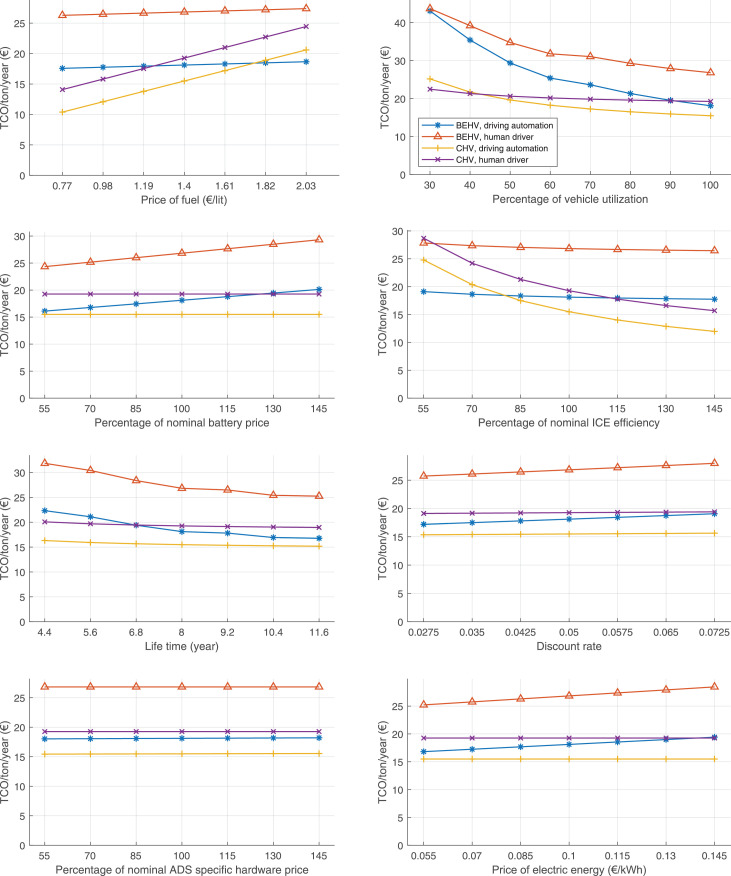
Nordic combination on a flat road of 320 km length; sensitivity of TCO to different parameters are shown for the optimum speed of the transportation scenario. See Table 6 for the other vehicle sizes and road types.

**Fig. 231:**
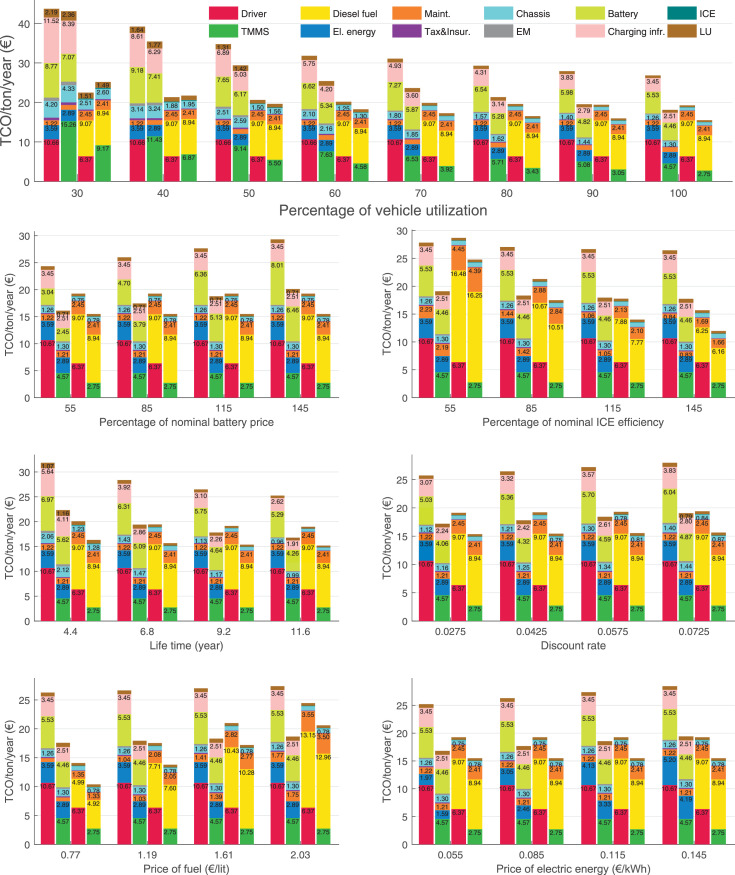
Nordic combination on a flat road of 320 km length; sensitivity of the TCO components of different vehicles and driving systems are shown for the optimum speed of the transportation scenario. A group of four bars from left to right represent BEHV HD, BEHV ADS-V, CHV HD and CHV ADS-DV, respectively. See Table 6 for the other vehicle sizes and road types.

**Fig. 232:**
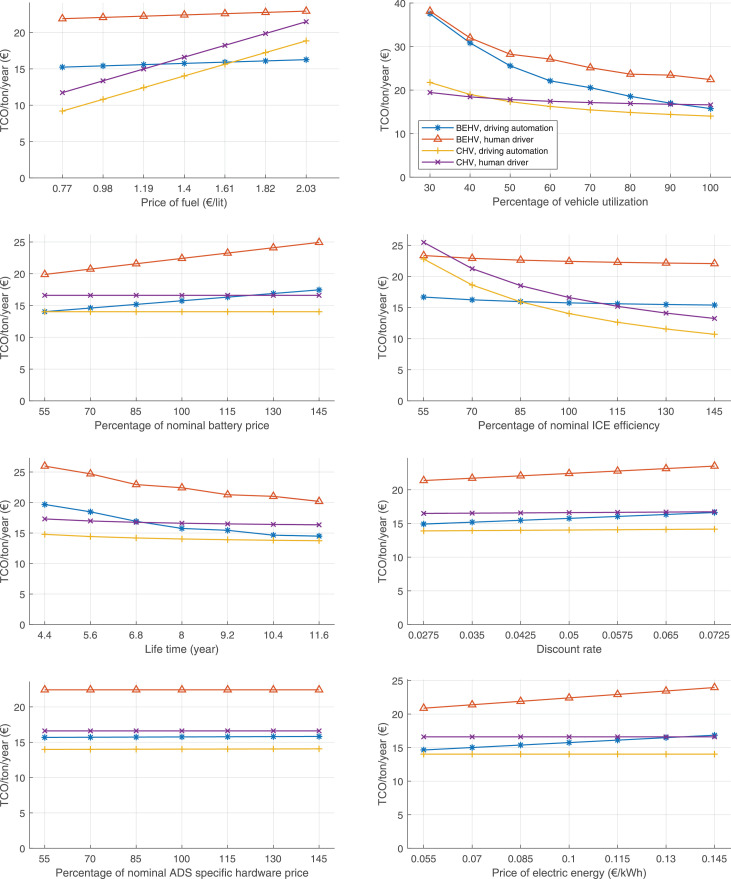
A-double on a flat road of 320 km length; sensitivity of TCO to different parameters are shown for the optimum speed of the transportation scenario. See Table 6 for the other vehicle sizes and road types.

**Fig. 233:**
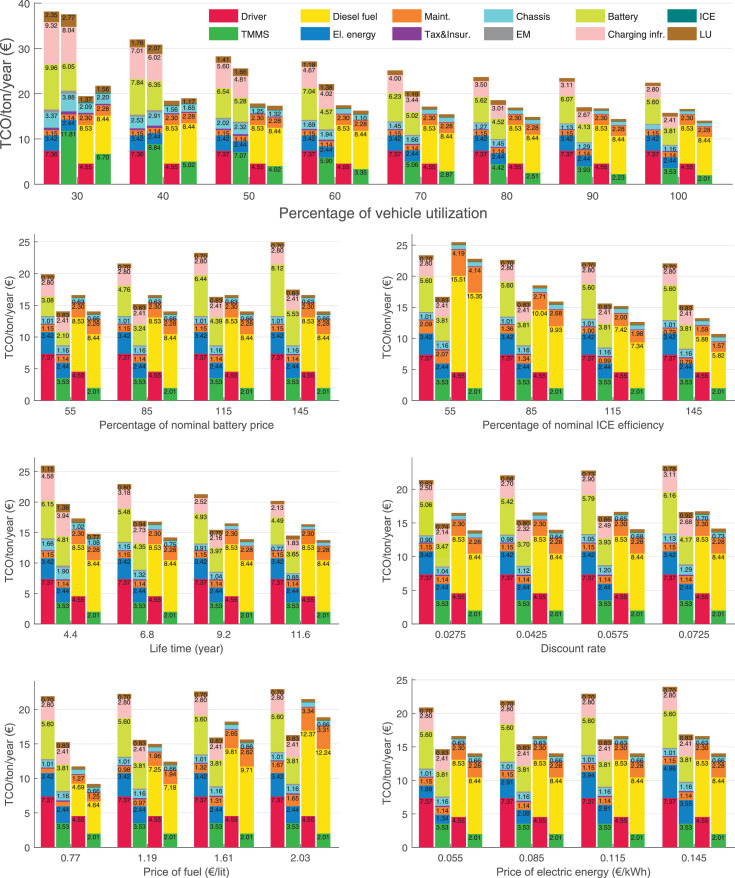
A-double on a flat road of 320 km length; sensitivity of the TCO components of different vehicles and driving systems are shown for the optimum speed of the transportation scenario. A group of four bars from left to right represent BEHV HD, BEHV ADS-V, CHV HD and CHV ADS-DV, respectively. See Table 6 for the other vehicle sizes and road types.

**Fig. 234:**
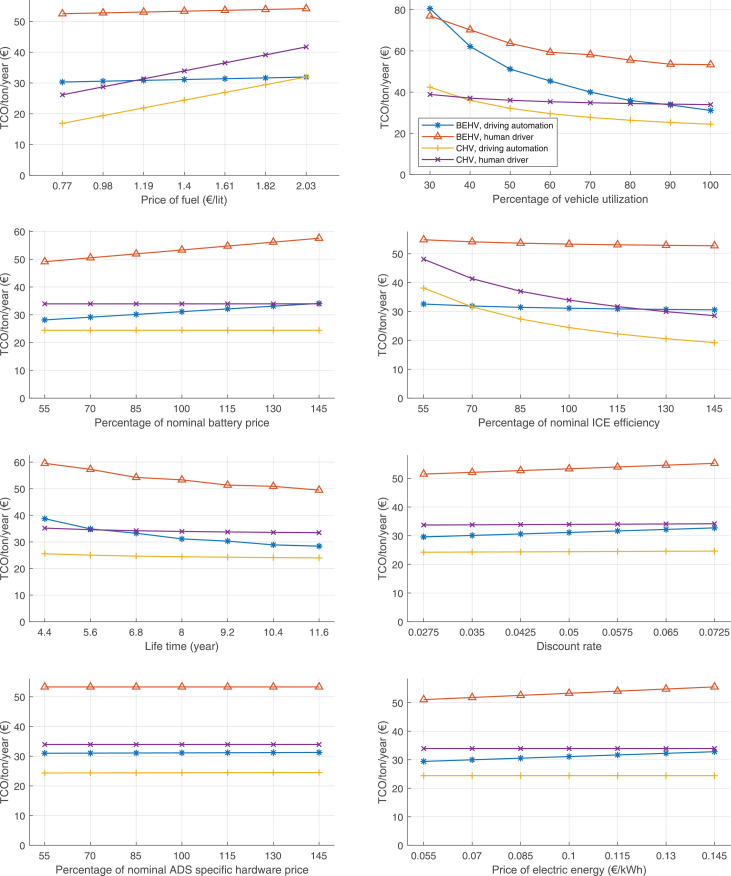
Rigid truck on a predominantly flat road of 320 km length; sensitivity of TCO to different parameters are shown for the optimum speed of the transportation scenario. See Table 6 for the other vehicle sizes and road types.

**Fig. 235:**
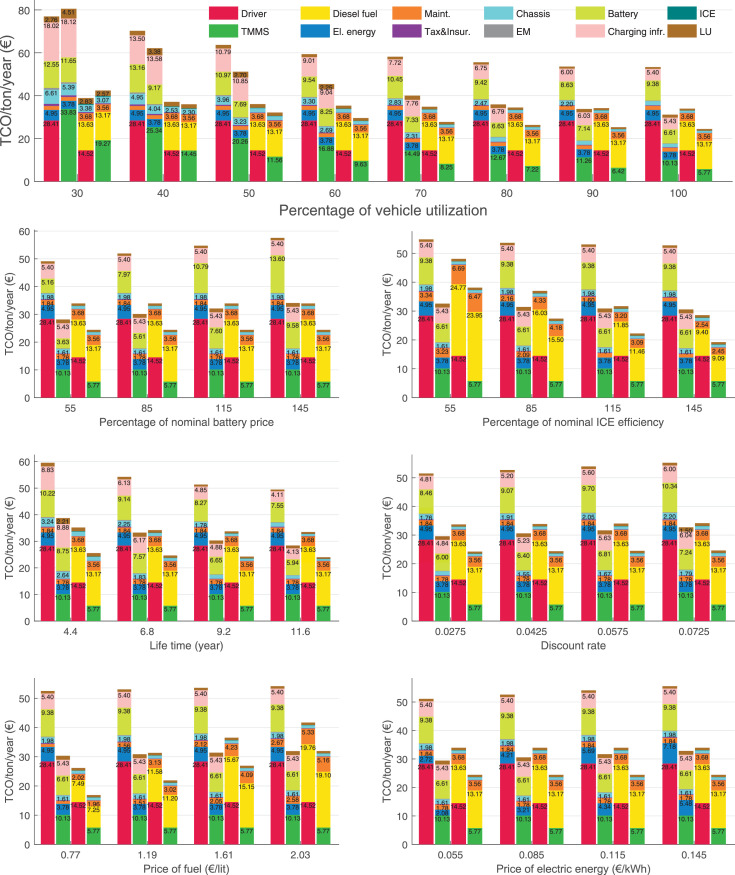
Rigid truck on a predominantly flat road of 320 km length; sensitivity of the TCO components of different vehicles and driving systems are shown for the optimum speed of the transportation scenario. A group of four bars from left to right represent BEHV HD, BEHV ADS-V, CHV HD and CHV ADS-DV, respectively. See Table 6 for the other vehicle sizes and road types.

**Fig. 236:**
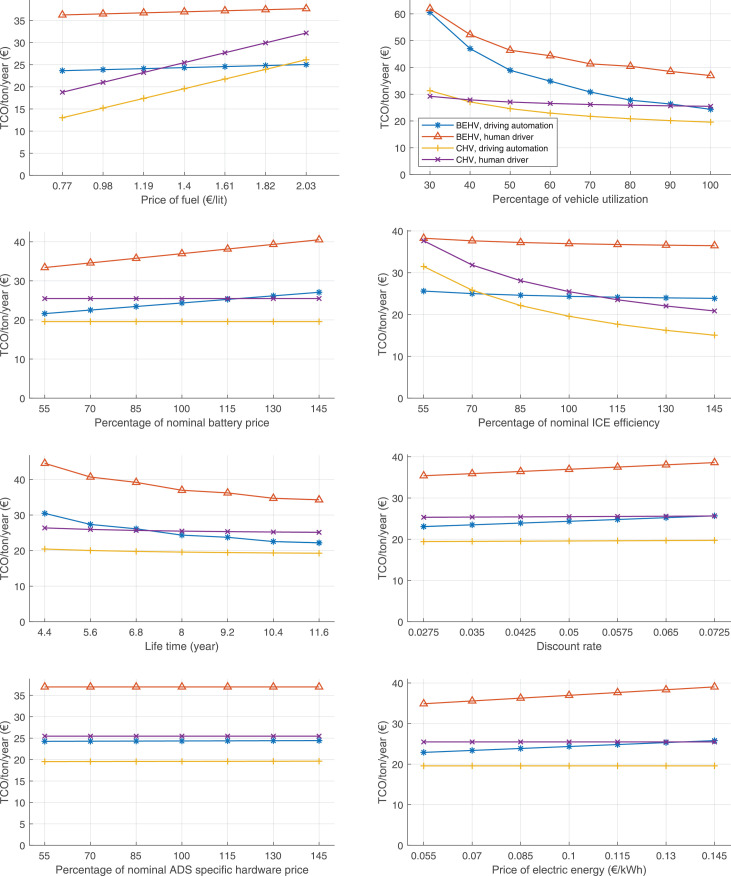
Tractor-semitrailer on a predominantly flat road of 320 km length; sensitivity of TCO to different parameters are shown for the optimum speed of the transportation scenario. See Table 6 for the other vehicle sizes and road types.

**Fig. 237:**
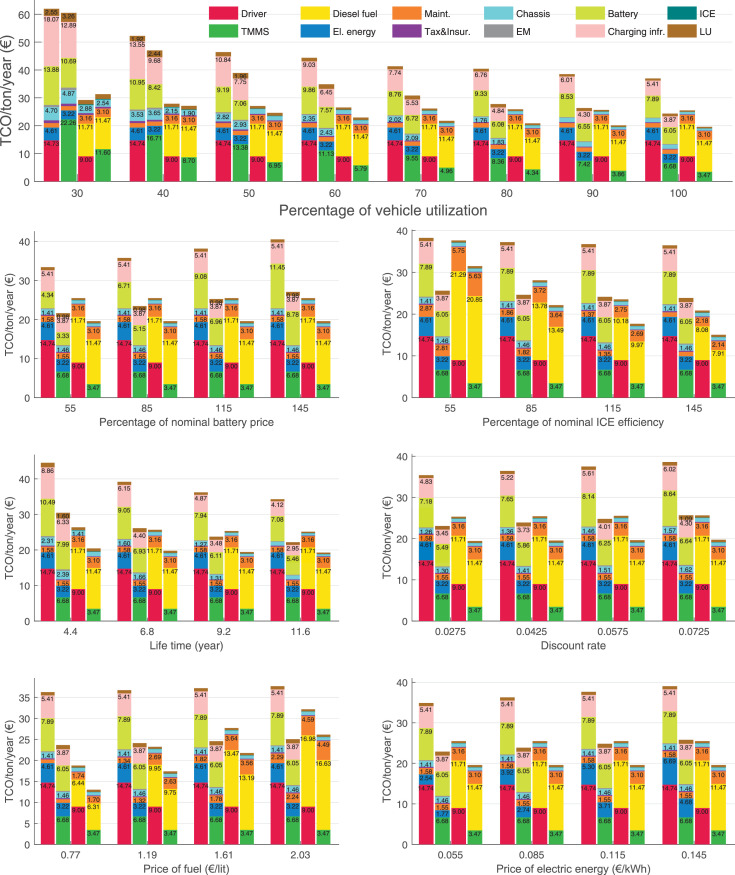
Tractor-semitrailer on a predominantly flat road of 320 km length; sensitivity of the TCO components of different vehicles and driving systems are shown for the optimum speed of the transportation scenario. A group of four bars from left to right represent BEHV HD, BEHV ADS-V, CHV HD and CHV ADS-DV, respectively. See Table 6 for the other vehicle sizes and road types.

**Fig. 238:**
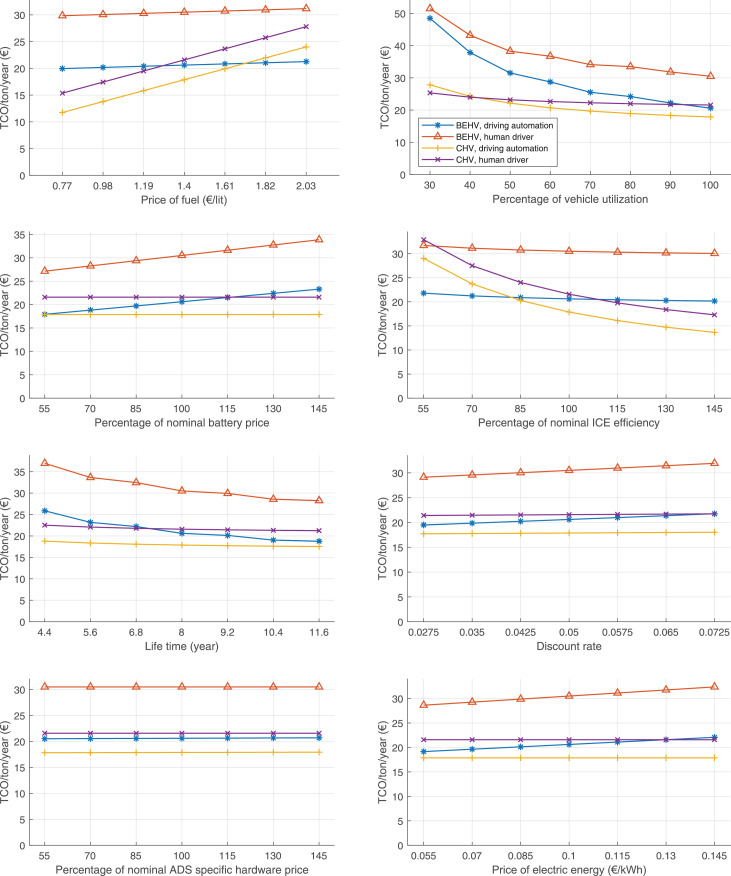
Nordic combination on a predominantly flat road of 320 km length; sensitivity of TCO to different parameters are shown for the optimum speed of the transportation scenario. See Table 6 for the other vehicle sizes and road types.

**Fig. 239:**
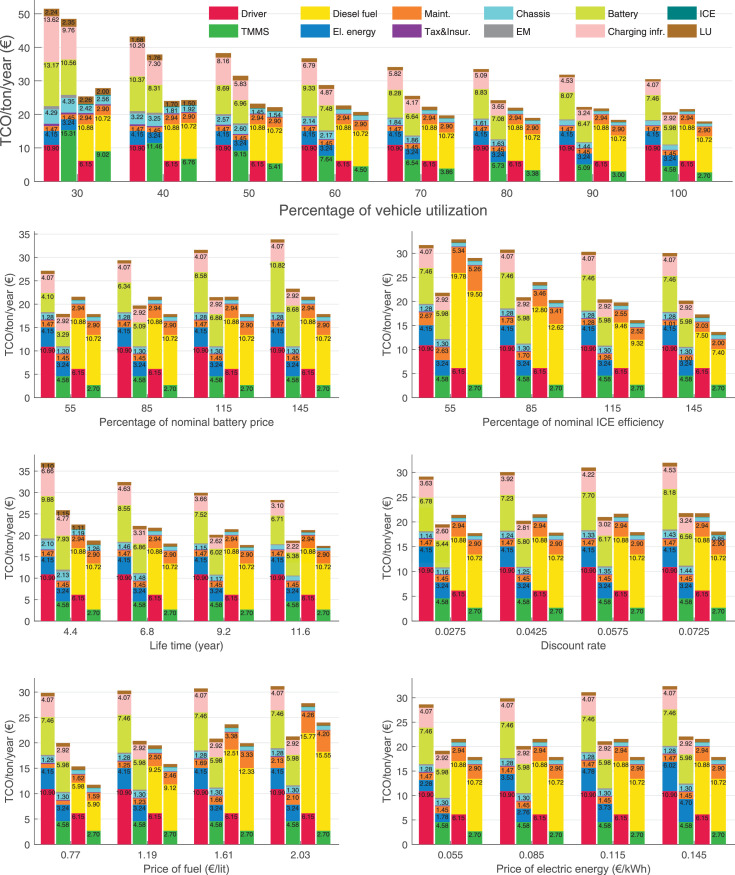
Nordic combination on a predominantly flat road of 320 km length; sensitivity of the TCO components of different vehicles and driving systems are shown for the optimum speed of the transportation scenario. A group of four bars from left to right represent BEHV HD, BEHV ADS-V, CHV HD and CHV ADS-DV, respectively. See Table 6 for the other vehicle sizes and road types.

**Fig. 240:**
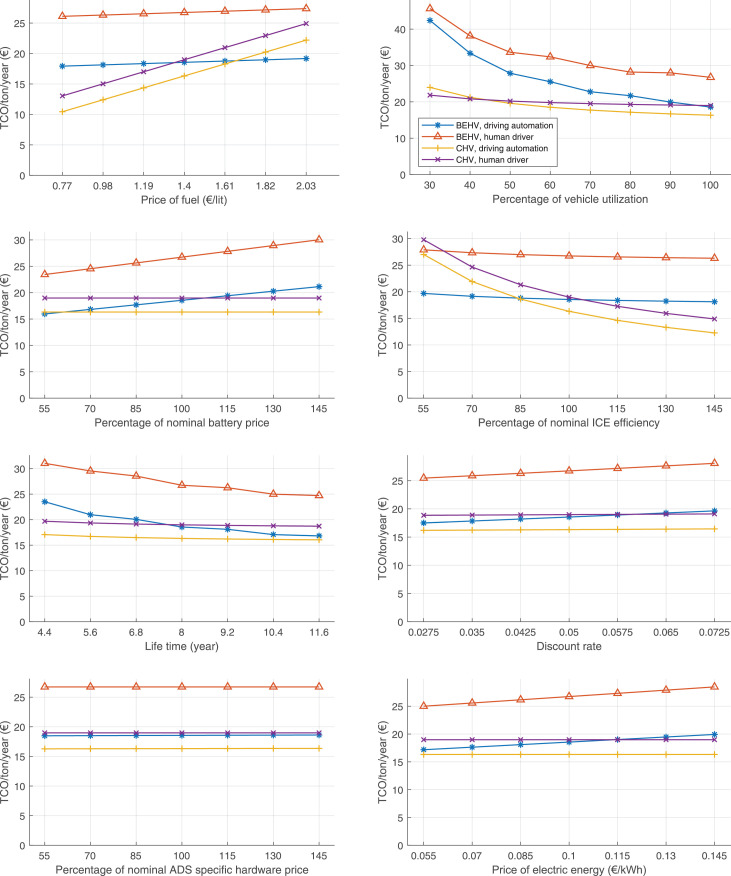
A-double on a predominantly flat road of 320 km length; sensitivity of TCO to different parameters are shown for the optimum speed of the transportation scenario. See Table 6 for the other vehicle sizes and road types.

**Fig. 241:**
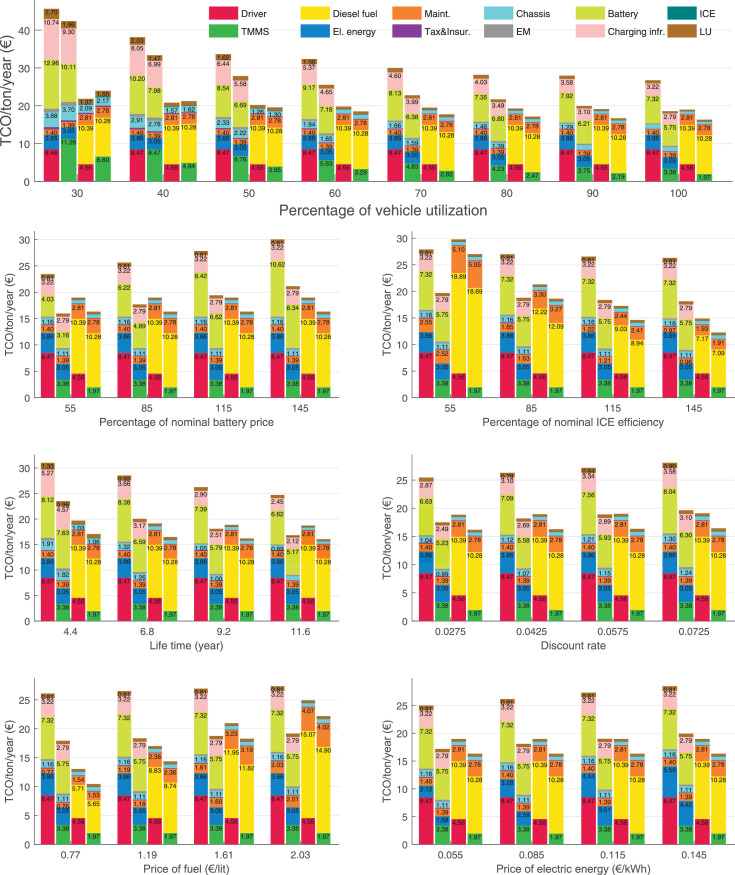
A-double on a predominantly flat road of 320 km length; sensitivity of the TCO components of different vehicles and driving systems are shown for the optimum speed of the transportation scenario. A group of four bars from left to right represent BEHV HD, BEHV ADS-V, CHV HD and CHV ADS-DV, respectively. See Table 6 for the other vehicle sizes and road types.

**Fig. 242:**
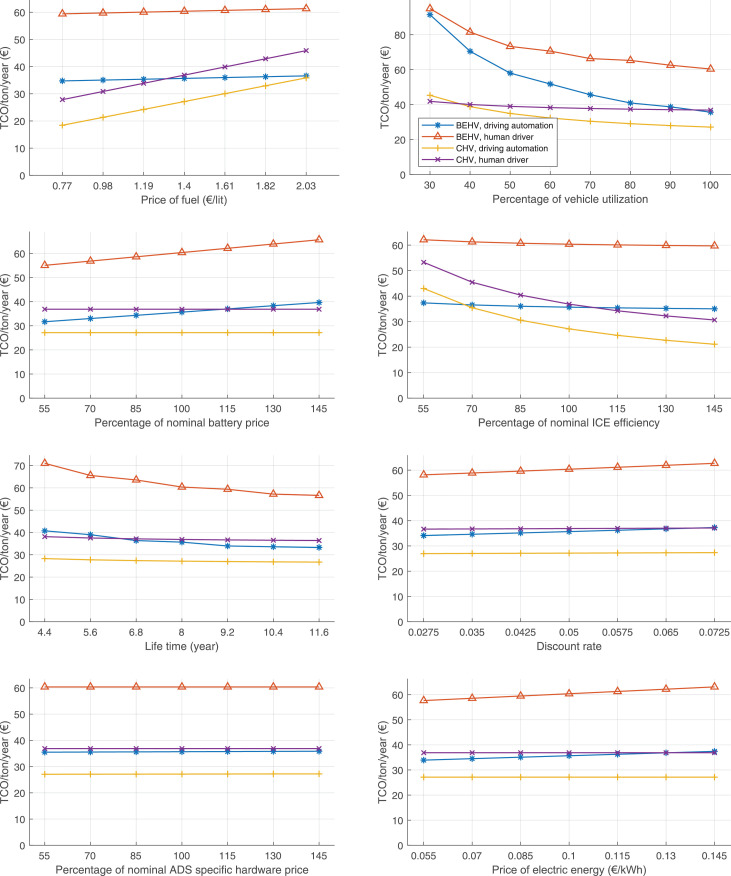
Rigid truck on a hilly road of 320 km length; sensitivity of TCO to different parameters are shown for the optimum speed of the transportation scenario. See Table 6 for the other vehicle sizes and road types.

**Fig. 243:**
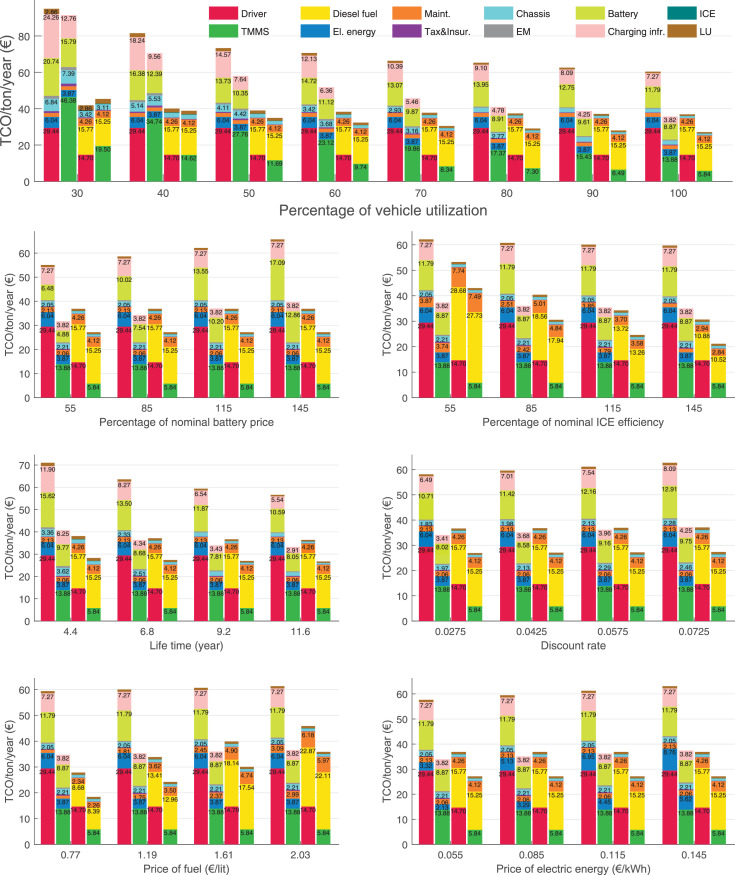
Rigid truck on a hilly road of 320 km length; sensitivity of the TCO components of different vehicles and driving systems are shown for the optimum speed of the transportation scenario. A group of four bars from left to right represent BEHV HD, BEHV ADS-V, CHV HD and CHV ADS-DV, respectively. See Table 6 for the other vehicle sizes and road types.

**Fig. 244:**
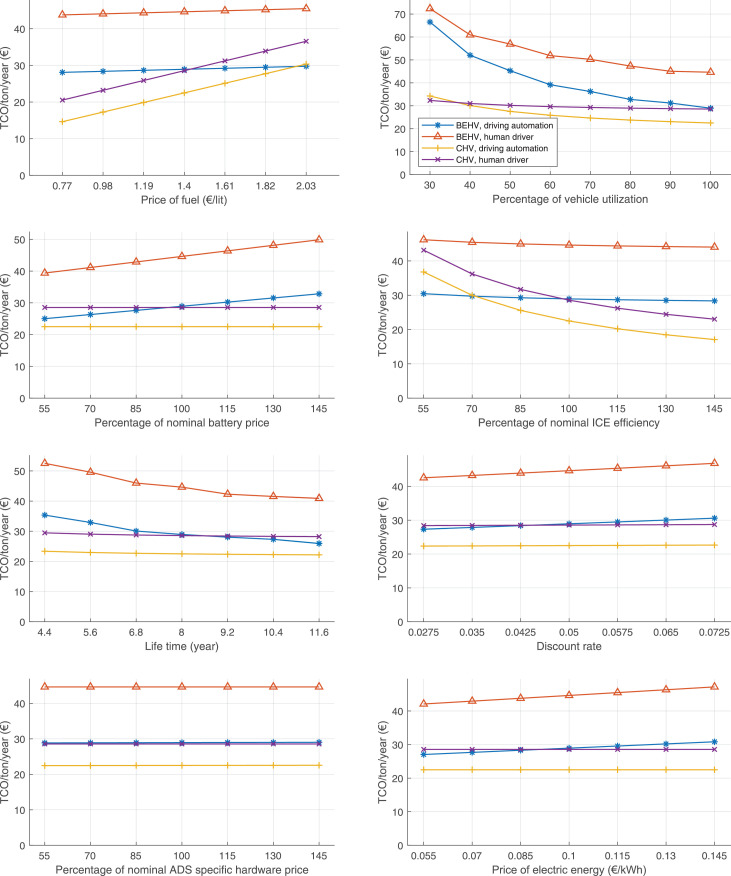
Tractor-semitrailer on a hilly road of 320 km length; sensitivity of TCO to different parameters are shown for the optimum speed of the transportation scenario. See Table 6 for the other vehicle sizes and road types.

**Fig. 245:**
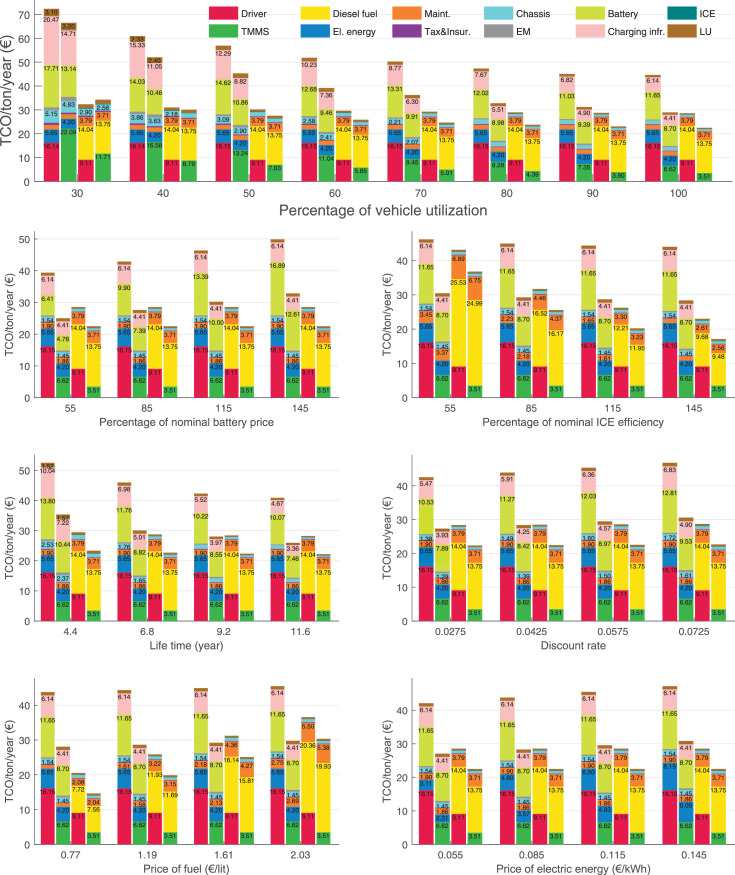
Tractor-semitrailer on a hilly road of 320 km length; sensitivity of the TCO components of different vehicles and driving systems are shown for the optimum speed of the transportation scenario. A group of four bars from left to right represent BEHV HD, BEHV ADS-V, CHV HD and CHV ADS-DV, respectively. See Table 6 for the other vehicle sizes and road types.

**Fig. 246:**
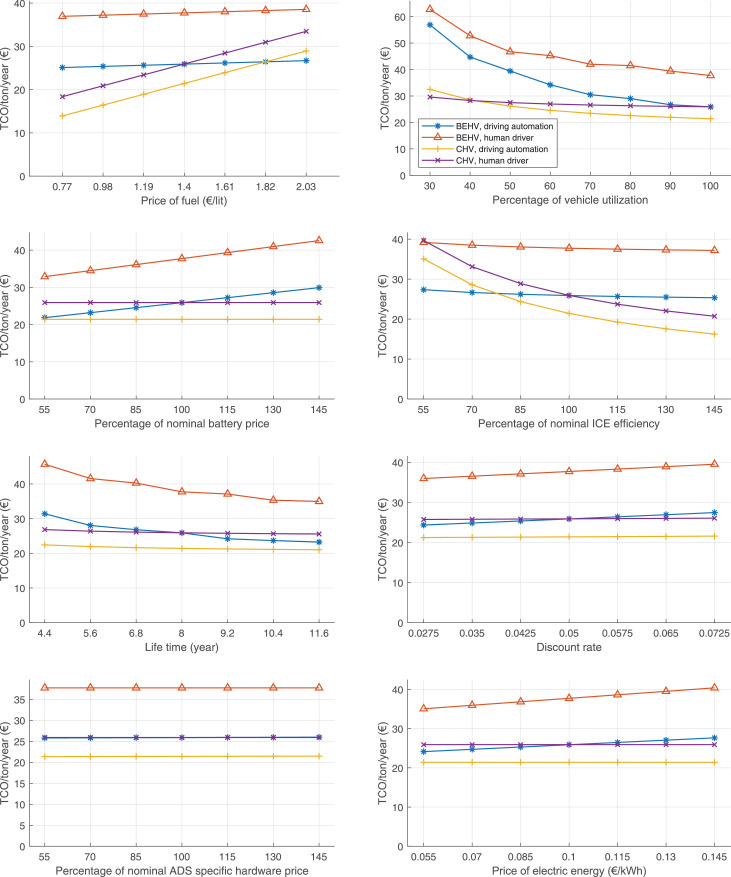
Nordic combination on a hilly road of 320 km length; sensitivity of TCO to different parameters are shown for the optimum speed of the transportation scenario. See Table 6 for the other vehicle sizes and road types.

**Fig. 247:**
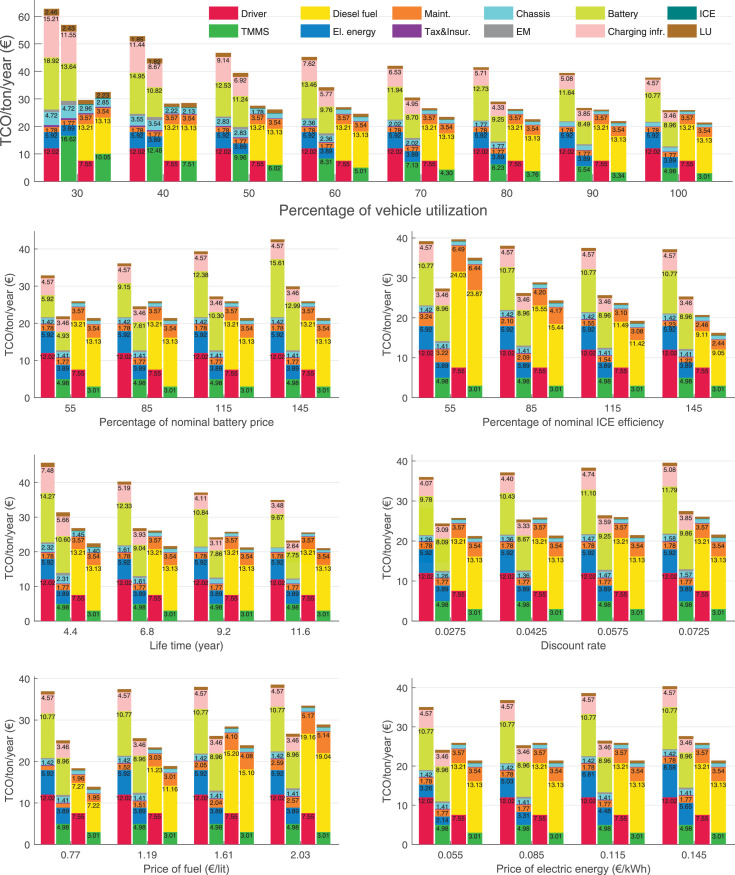
Nordic combination on a hilly road of 320 km length; sensitivity of the TCO components of different vehicles and driving systems are shown for the optimum speed of the transportation scenario. A group of four bars from left to right represent BEHV HD, BEHV ADS-V, CHV HD and CHV ADS-DV, respectively. See Table 6 for the other vehicle sizes and road types.

**Fig. 248:**
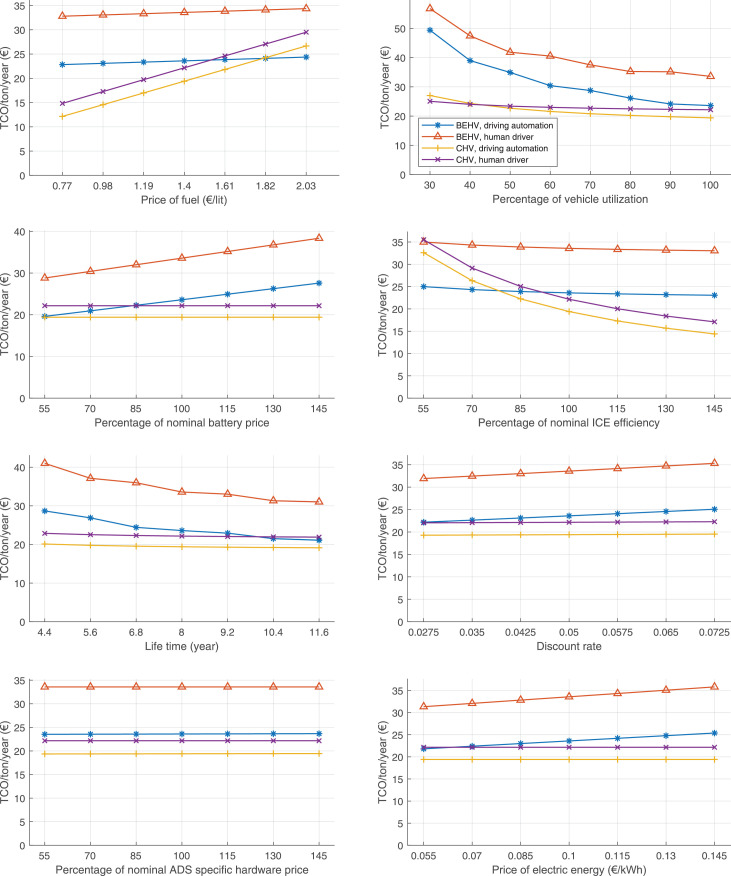
A-double on a hilly road of 320 km length; sensitivity of TCO to different parameters are shown for the optimum speed of the transportation scenario. See Table 6 for the other vehicle sizes and road types.

**Fig. 249:**
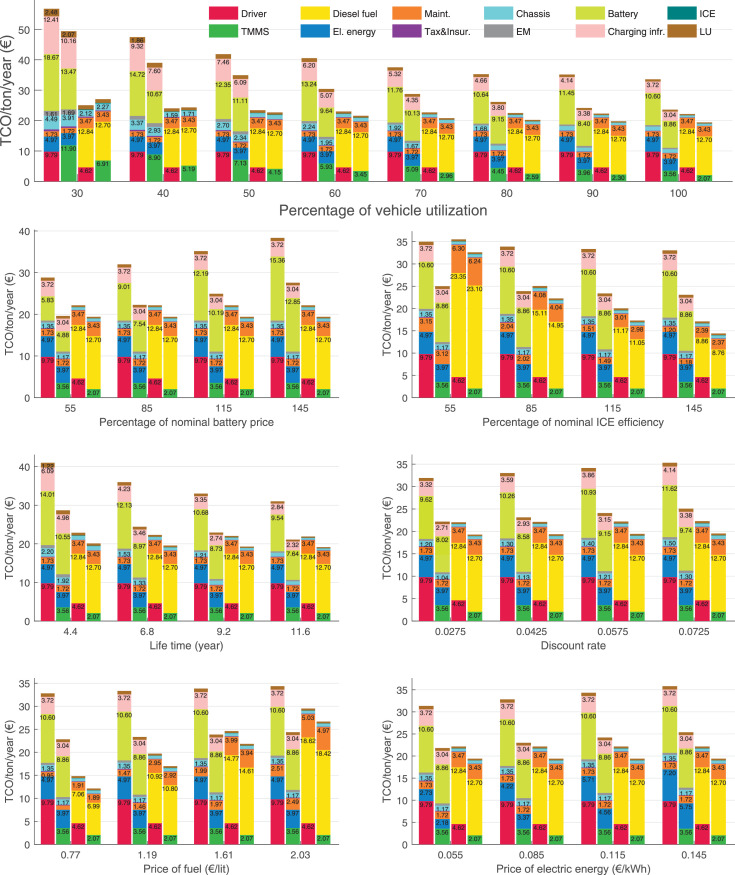
A-double on a hilly road of 320 km length; sensitivity of the TCO components of different vehicles and driving systems are shown for the optimum speed of the transportation scenario. A group of four bars from left to right represent BEHV HD, BEHV ADS-V, CHV HD and CHV ADS-DV, respectively. See Table 6 for the other vehicle sizes and road types.

**Fig. 250:**
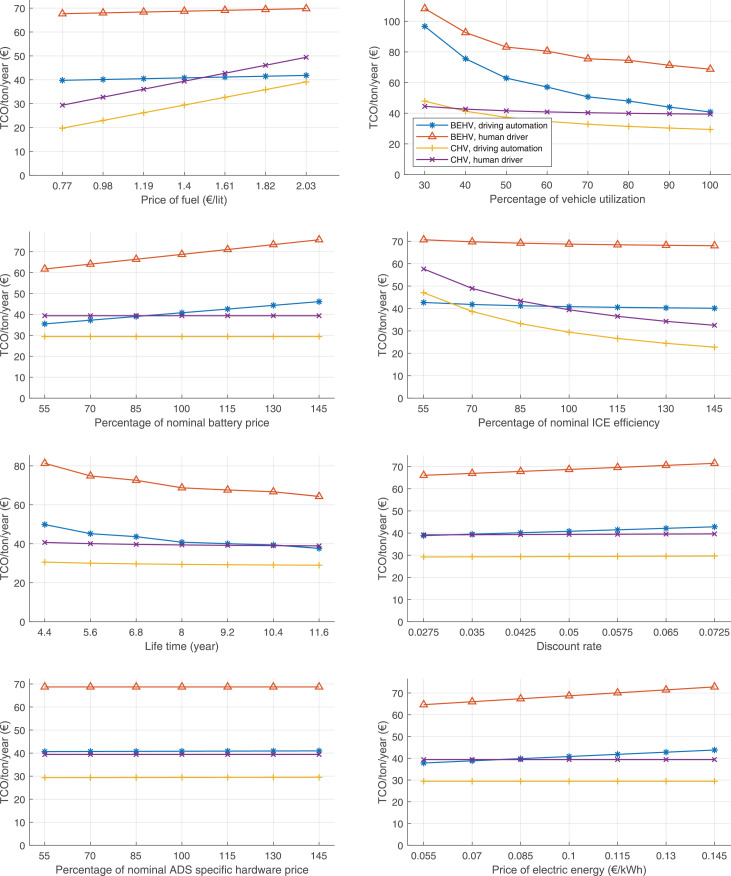
Rigid truck on a very hilly road of 320 km length; sensitivity of TCO to different parameters are shown for the optimum speed of the transportation scenario. See Table 6 for the other vehicle sizes and road types.

**Fig. 251:**
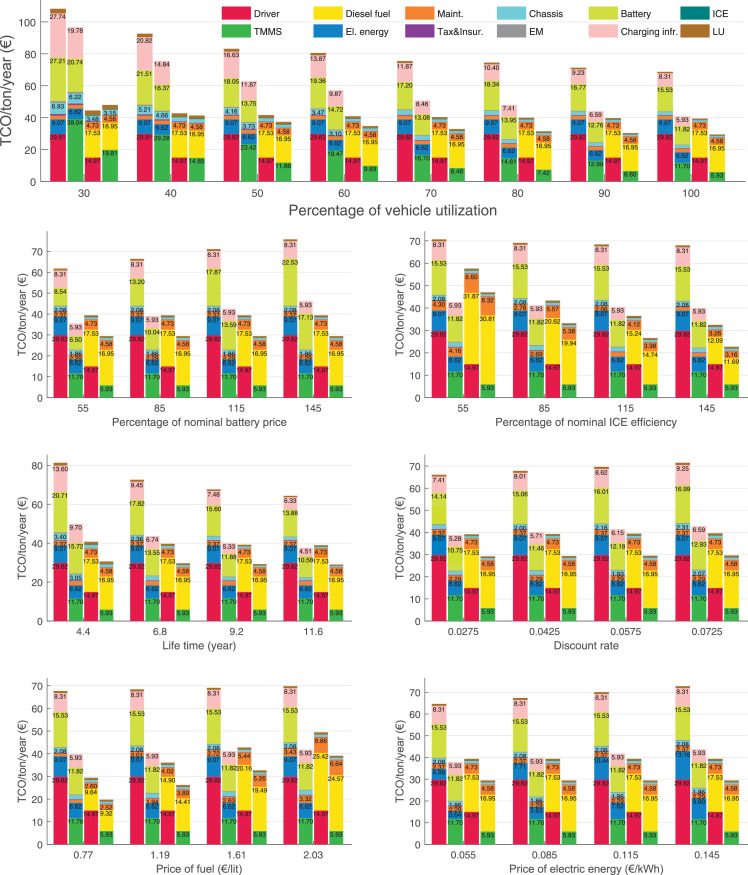
Rigid truck on a very hilly road of 320 km length; sensitivity of the TCO components of different vehicles and driving systems are shown for the optimum speed of the transportation scenario. A group of four bars from left to right represent BEHV HD, BEHV ADS-V, CHV HD and CHV ADS-DV, respectively. See Table 6 for the other vehicle sizes and road types.

**Fig. 252:**
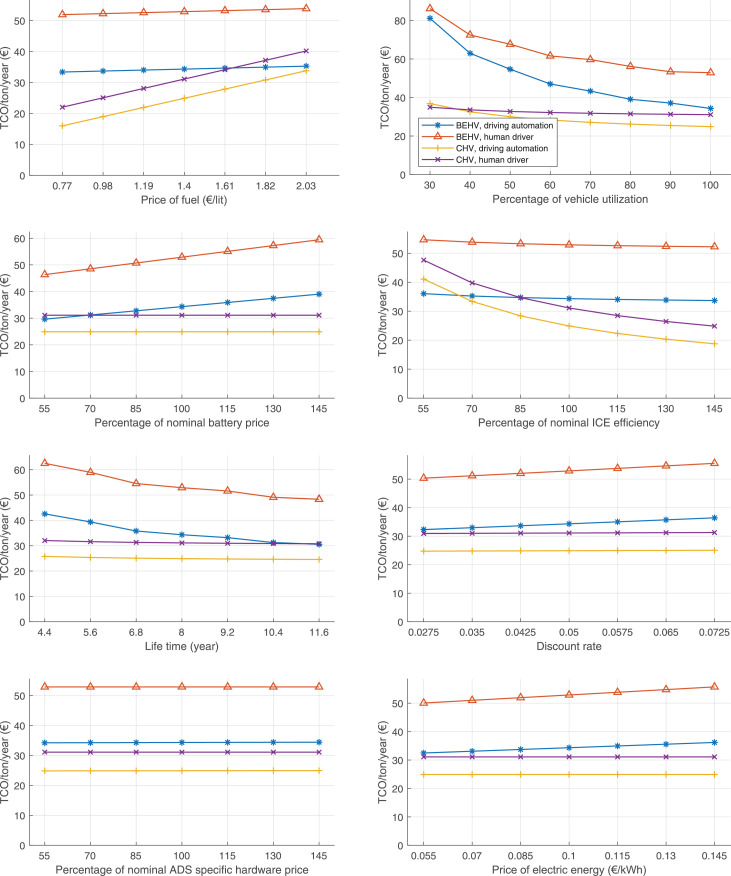
Tractor-semitrailer on a very hilly road of 320 km length; sensitivity of TCO to different parameters are shown for the optimum speed of the transportation scenario. See Table 6 for the other vehicle sizes and road types.

**Fig. 253:**
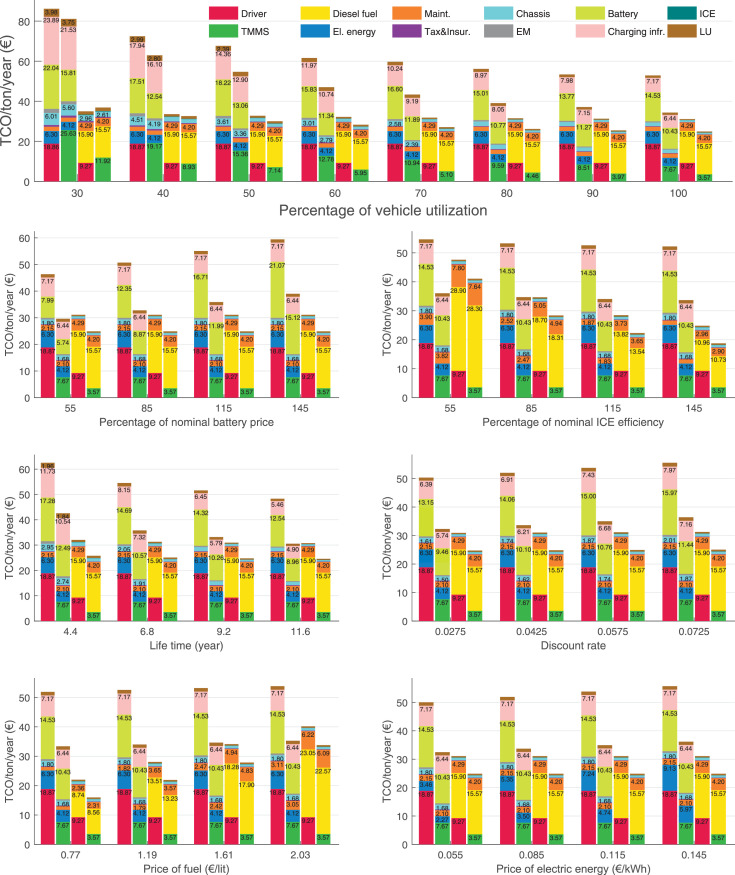
Tractor-semitrailer on a very hilly road of 320 km length; sensitivity of the TCO components of different vehicles and driving systems are shown for the optimum speed of the transportation scenario. A group of four bars from left to right represent BEHV HD, BEHV ADS-V, CHV HD and CHV ADS-DV, respectively. See Table 6 for the other vehicle sizes and road types.

**Fig. 254:**
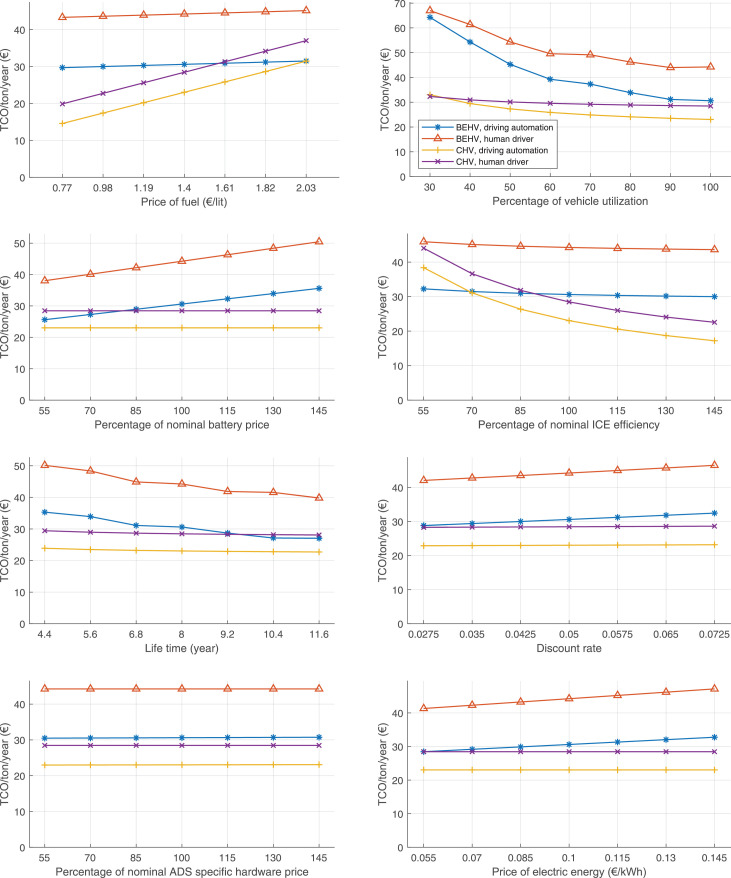
Nordic combination on a very hilly road of 320 km length; sensitivity of TCO to different parameters are shown for the optimum speed of the transportation scenario. See Table 6 for the other vehicle sizes and road types.

**Fig. 255:**
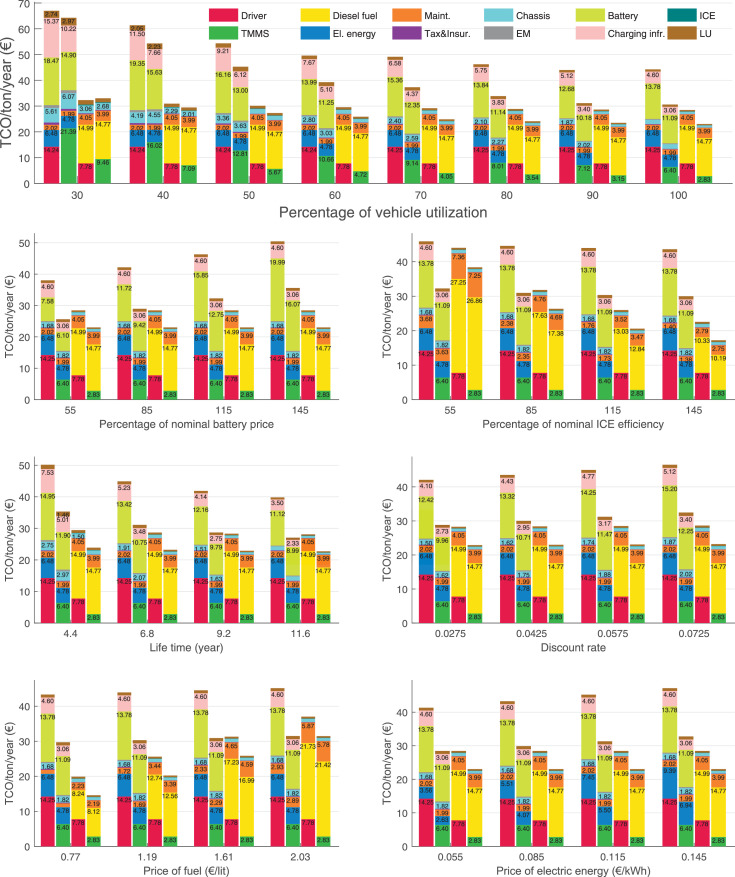
Nordic combination on a very hilly road of 320 km length; sensitivity of the TCO components of different vehicles and driving systems are shown for the optimum speed of the transportation scenario. A group of four bars from left to right represent BEHV HD, BEHV ADS-V, CHV HD and CHV ADS-DV, respectively. See Table 6 for the other vehicle sizes and road types.

**Fig. 256:**
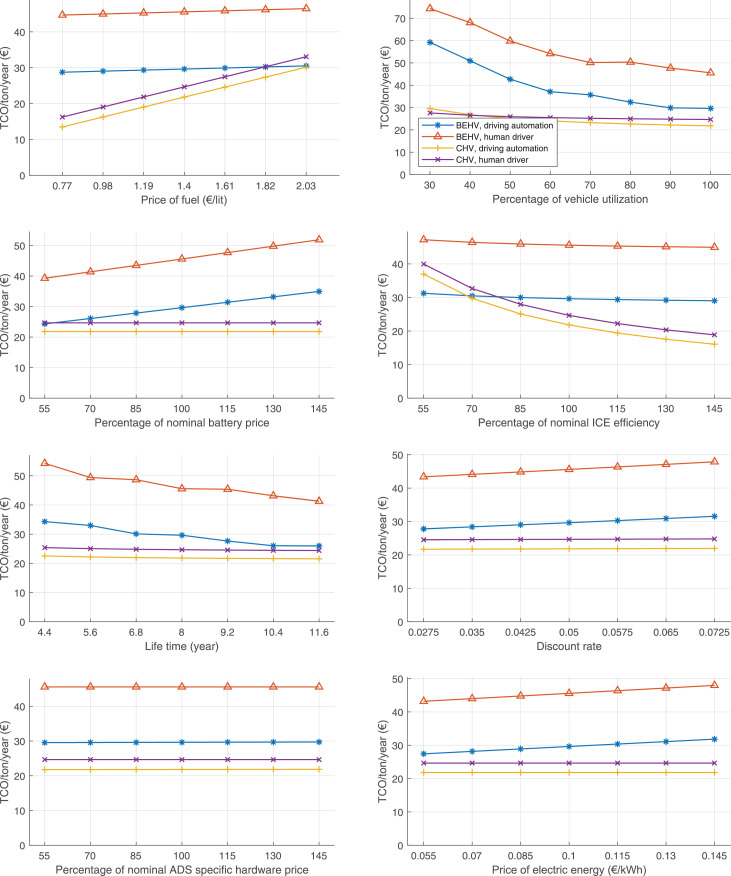
A-double on a very hilly road of 320 km length; sensitivity of TCO to different parameters are shown for the optimum speed of the transportation scenario. See Table 6 for the other vehicle sizes and road types.

**Fig. 257:**
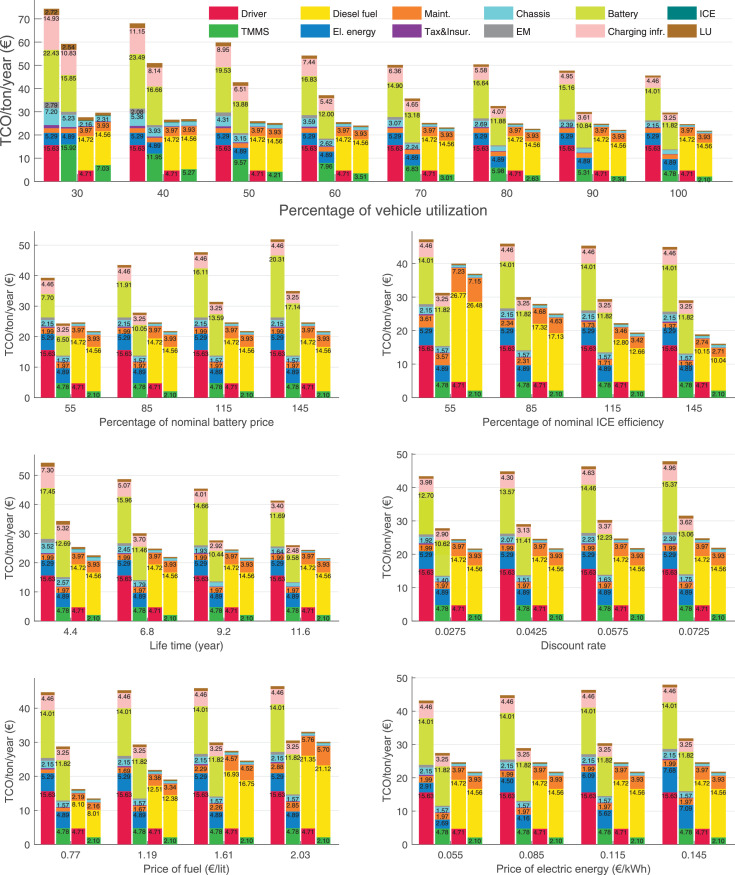
A-double on a very hilly road of 320 km length; sensitivity of the TCO components of different vehicles and driving systems are shown for the optimum speed of the transportation scenario. A group of four bars from left to right represent BEHV HD, BEHV ADS-V, CHV HD and CHV ADS-DV, respectively. See Table 6 for the other vehicle sizes and road types.

## Appendix A Supplementary materials

Supplementary Data S1 Supplementary Raw Research Data. This is open data under the CC BY license http://creativecommons.org/licenses/by/4.0/ Supplementary Data S1
